# Stereoselective additions to alkenylphosphonium salts for the synthesis of P-stereogenic compounds

**DOI:** 10.1039/d5sc09946c

**Published:** 2026-02-20

**Authors:** Xiao-Bing Chen, Damián Padín, Ben L. Feringa

**Affiliations:** a Stratingh Institute for Chemistry, University of Groningen Groningen 9747AG The Netherlands b.l.feringa@rug.nl

## Abstract

The stereoselective functionalisation of alkenyl P(v) compounds *via* conjugate additions represents an attractive approach to synthesise chiral organophosphorus compounds. However, asymmetric conjugate additions to alkenyl P(v) compounds are scarce and, in the presence of P-stereogenic centers, diastereoinduction is often low. Here, we report the use of BINOL-based alkenylphosphonium salts for the generation of two non-consecutive P- and C-stereogenic centers *via* addition of C-nucleophiles in a single operation. These alkenylphosphonium salts behave as activated alkenylphosphonamidate surrogates with increased reactivity and stereocontrol. This methodogy allows the versatile preparation of enantioenriched organophosphorus building-blocks in high yield and stereoselectivity.

## Abstract 2

BINOL-based alkenylphosphonium salts enable highly stereoselective C-nucleophile additions, delivering P- and C-stereogenic organophosphorus compounds in a single operation with high yield and stereocontrol.

## Graphical abstract

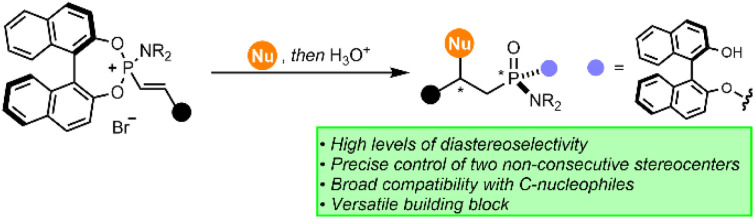

## Introduction

The stereoselective synthesis of P-stereogenic building blocks has become a major goal in organic synthesis due to their increasingly widespread use in ligand-design for asymmetric catalysis,^1–3^ medicinal chemistry and agrochemistry.^4,5^ Yet, routes to access chiral organophosphorus compounds are limited and less explored than C-stereogenic compounds. Recently, notable advancements in the field have been reported by using chiral auxiliaries,^6–15^ resolution of enantiomers, formation of diastereomeric complexes^16^ or catalytic asymmetric synthesis.^17–20^ Importantly, approaches that render multiple orthogonal functionalisation sites at P-chiral building blocks have gained particular attention, as they allow a straightforward and modular access to a myriad of chiral enantioenriched compounds from a relatively small number of precursors.^8,18,21–25^

Chiral alkenyl P(v) compounds (*e.g.* alkenylphosphonates, alkenyl phosphine oxides or alkenylphosphonamidates, among others) would represent a suitable platform for preparing P-stereogenic compounds. In principle, the combination of a P-stereocenter and a mildly electrophilic olefin would allow the functionalisation at the β-carbon *via* conjugate addition and, potentially, the formation of a new C-stereocenter.^26^ Not surprisingly, this motif has attracted considerable attention in recent years and several methods have been devised by our group^27^ and others using asymmetric hydrophosphonylation of alkynes,^28–35^ C_sp^2^_-P(iii)/P(v) cross-coupling reactions^27,36,37^ or desymmetrisation reactions.^38–42^

**Fig. 1:**
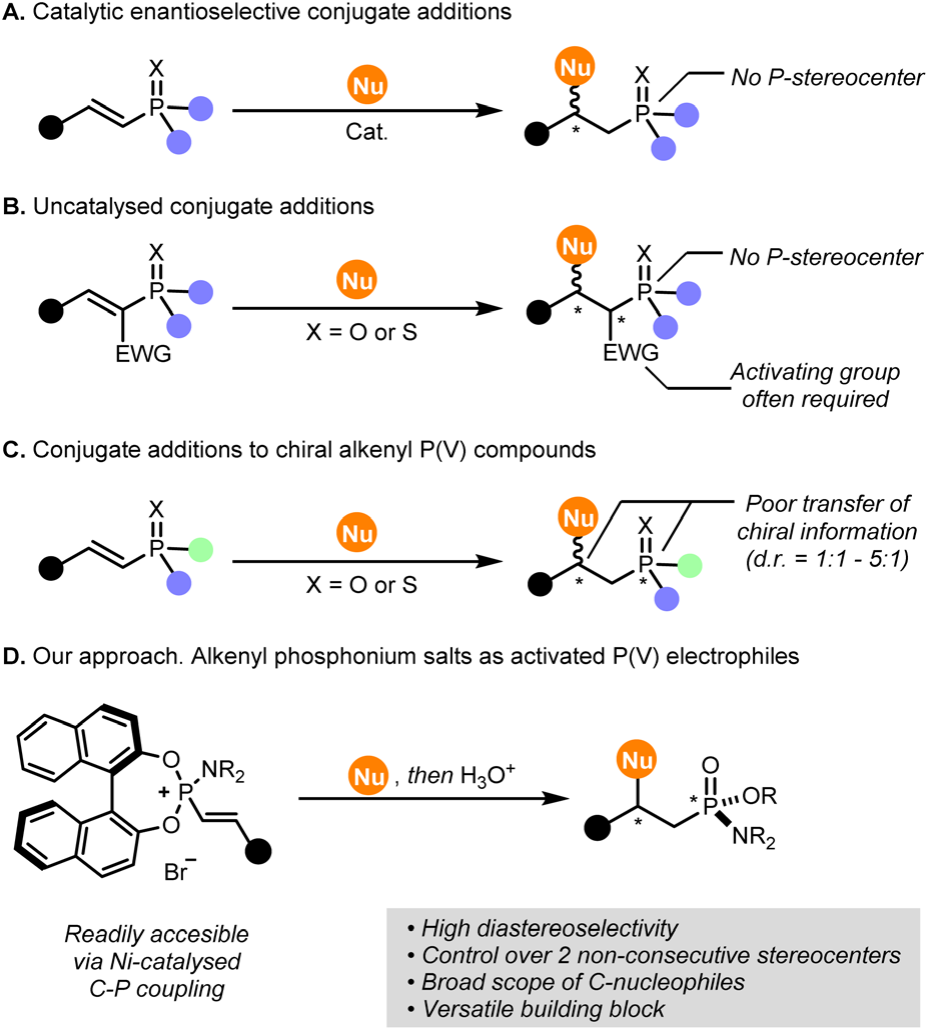
State-of-the-art and challenges in stereoselective conjugate additions using (chiral) alkenyl P(v) compounds as electrophiles.

However, stereoselective functionalisations of alkenyl P(v) compounds *via* conjugate additions are not trivial, and current methodologies suffer from certain limitations. First, our group^43^ and others^44–49^ have developed catalytic asymmetric conjugate additions to alkenyl P(v) compounds, however, the introduction of P-stereocenters was not explored using this approach (Fig. 1A). Second, alkenyl P(v) compounds are challenging Michael acceptors that often require the presence of activating groups (Fig. 1B).^50–53^ Third, conjugate additions to alkenyl P(v) compounds bearing a P-stereogenic centre show, in most cases, poor diastereoselectivity (from 1 : 1 to 5 : 1 d.r.), as a result of an inefficient transfer of chiral information from the P-stereocenter to the β position in the substrate (Fig. 1C).^28–30,33–35,42,54,55^

In our previous report on Ni(0)-catalysed alkenylation of BINOL-based phosphoramidites towards chiral-at-P alkenylphosphonamidates, we identified alkenylphosphonium salts as key intermediates.^27^ These intermediates proved to be reasonably stable under ambient conditions and, upon hydrolysis under acidic conditions, provided versatile P-stereogenic alkenylphosphonamidates with high optical purity. While we were unsuccessful to selectively functionalise the β position of the final alkenylphosphonamidates, preliminary studies suggested that the alkenylphosphonium intermediates could actually be more reactive and selective towards formal conjugate addition processes. In the literature, simple alkenylphosphonium salts, such as triphenylvinylphosphonium bromide, have been widely explored as electrophiles with a variety of nucleophiles.^56–60^ However, to the best of our knowledge, no stereoselective additions have been reported, so far. Moreover, none of the reported methodologies allow the access to P-stereogenic compounds in a single operation. Addressing this challenge, we report here the use of readily available BINOL-based alkenylphosphonium salts as activated alkenyl P(v) precursors in stereoselective additions with a wide range of C-nucleophiles (Fig. 1D). Remarkably, the final phosphonamidates obtained by this methodology feature two non-consecutive stereocenters, *i.e.*, a P-stereocenter and a C-stereocenter, controlled by a highly efficient axial-to-point chirality transfer promoted by the BINOL moiety. In addition, these chiral organophosphorus compounds present several functionalisation sites suitable for further derivatization downstream.

## Results and discussion

**Fig. 2:**
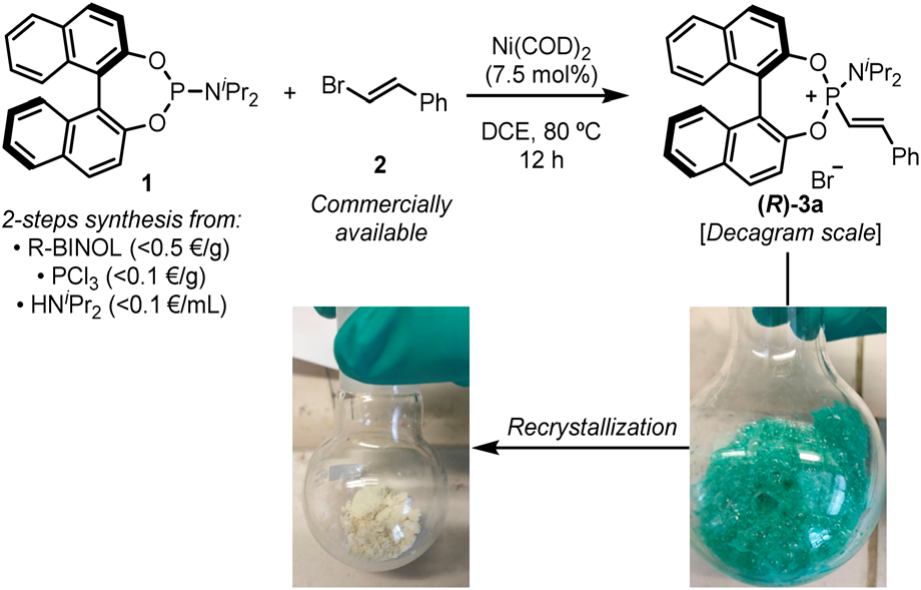
Practical preparation of chiral alkenylphosphonium salt (*R*)-3a through a Ni(0)-catalysed C–P coupling reaction.

Chiral alkenylphosphonium salts, such as (*R*)-3a, could be easily accessed in multi-gram scale and nearly quantitative yields from readily available BINOL-based phosphoramidites and β-bromostyrenes using a variation of our previously reported Ni(0)-catalysed C–P coupling reaction (Fig. 2).^27^ These phosphonium salts proved to be reasonably bench-stable, although partial hydrolysis in the presence of moisture or during purification by column chromatography was occasionally observed. Interestingly, although the crude reaction mixture after evaporation of the solvent provided a reasonably pure phosphonium salt, Ni(ii) contamination, which rendered the crude product a characteristic bright green colour, proved to be detrimental in subsequent manipulations of the product. Nonetheless, a simple recrystallization of the crude enabled the removal of any Ni(ii) impurities and provided the phosphonium salts as off-white solids.

For the development of an asymmetric addition to alkenylphosphonium salts, we chose phosphonium salt (*R*)-3a and a commercially available Grignard reagent, 4-methoxyphenylmagnesium bromide, as model substrates (Table 1). Encouraged by our previous experience on catalytic asymmetric 1,4-additions using organometallic reagents,^61–67^ we envisioned that the addition of Cu^I^/Cu^II^ salts should promote the addition reaction. Indeed, preliminary experiments using different copper salts as catalysts in THF at room temperature provided nearly quantitative conversions of (*R*)-3a, which, upon hydrolysis under acidic conditions, led to adduct 5 as a mixture of diastereoisomers (Table 1, entry 1). Importantly, while the addition of the Grignard reagent generates the C-stereocenter (referred to condition 1 in Table 1), the hydrolysis step generates the P-stereocenter (condition 2). Given the presence of the enantiopure BINOL auxiliary, four diastereoisomers can be observed in the ^31^P-NMR of the crude mixture (see SI for NMR monitoring of the reaction). Interestingly, this two-step, one-pot procedure using copper salts as catalysts led us to observe the predominant formation of one of the possible diastereoisomers, albeit in moderate stereoselectivity. Subsequent control experiments allowed us to discover that the use of a catalyst was, in fact, not necessary (entry 2). In an attempt to improve the diastereoselectivity, the reaction was conducted at low temperatures (entries 3 and 4), however, poor conversions and moderate stereoselectivities were observed. After extensive optimisation studies (see SI for a detailed optimisation table), we established that the pH of the hydrolysis step had a critical role in controlling the stereoselection. While in our previous Pd-catalysed arylation of phosphoramidites^12^ the addition of Cs_2_CO_3_ proved crucial for obtaining high yields and stereoselectivities, and in the Ni-catalysed alkenylation,^27^ addition of aqueous HCl led to the highest diastereoselectivities, in the current study, addition of neutral water produced better results than Cs_2_CO_3_ or HCl (compare entries 2, 5 and 6). Curiously, a remarkable diastereodivergency was observed when performing the hydrolysis step in water or in the presence of Cs_2_CO_3_ (entry 2 *vs.* entries 5 and 6). Finally, further screening of the reaction conditions allowed us to identify 1,4-dioxane as the optimal solvent for both, the Grignard addition and the hydrolysis steps (entry 7). Under these conditions, adduct (*R*,*R*_p_,*S*)-5 was obtained in high yield (87%) and excellent diastereoselectivity (88 : 0 : 4 : 8 d.r.). Notably, the diastereomeric purity of the final product could be often improved after purification of the crude compound by simple column chromatography. Thus, in practice, only two, out of four possible stereoisomers, were isolated after purification (*vide infra*).

**Table 1:** Screening of reaction conditions^a^

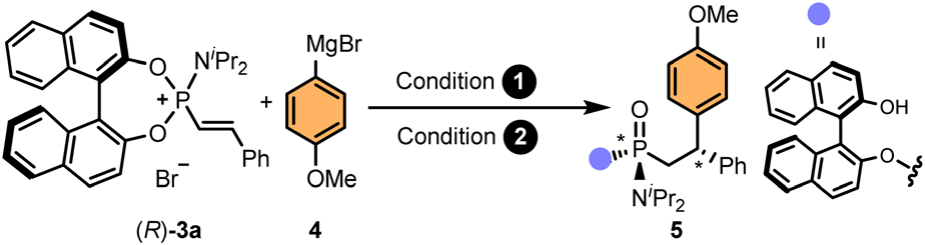			
Entry	Conditions	Conversion^b^ (%)	d.r.^b^
1	(1) Cu^I^/Cu^II^ salts, THF, r.t.	70–100	15 : 6 : 14 : 65–14 : 1 : 10 : 75
	(2) HCl_aq_		
2	(1) THF, r.t.	84	11 : 2 : 10 : 77
	(2) HCl_aq_		
3	(1) THF,-78 °C	35	21 : 3 : 10 : 66
	(2) HCl_aq_		
4	(1) THF, 0 °C	84	11 : 2 : 10 : 76
	(2) HCl_aq_		
5	(1) THF, r.t.	92	81 : 11 : 1 : 7
	(2) H_2_O		
6	(1) THF, r.t.	90	63 : 7 : 3 : 27
	(2) Cs_2_CO_3_ (2 equiv.)		
**7**	**(1) 1,4-Dioxane, r.t.**	**98 (87)^c^**	**88 : 0** : **4 : 8**
	**(2) H**_**2**_ **O**		

a Reactions performed with (*R*)-3a (0.1 mmol), 4-methoxyphenylmagnesium bromide (1.5 equiv.), under the indicated conditions. For the full optimisation table, see the SI.

b Determined by ^31^P-NMR and ^1^H-NMR (CDCl_3_) of the crude reaction mixture.

c Isolated yield in brackets.

With the optimised conditions in hand, we evaluated the generality of the methodology and its limitations. First, we analysed the effect of the phosphonium salt counterion on the yield and stereoselectivity (Table 2). Curiously, while phosphonium bromide (*R*)-3a-Br and iodide (*R*)-3a-I provided the desired phosphonamidate 5 in good yield and stereoselectivity, triflate (*R*)-3a-OTf led to a diminished yield, albeit improved stereoselectivity, and chloride (*R*)-3a-Cl only produced trace amounts of (*R*,*R*_p_,*S*)-5.

**Table 2:** Effect of the counterion^a^

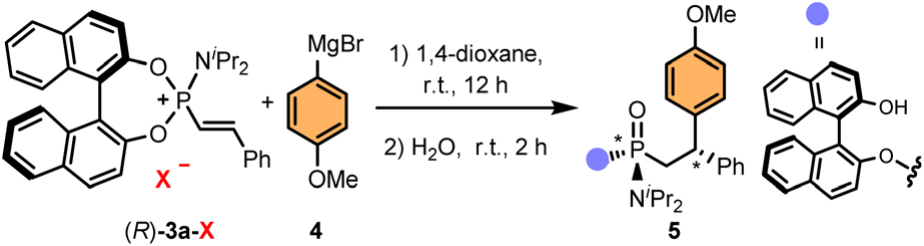		
X	Yield^b^	d.r.^c^
Cl	Trace	—
Br	87	88 : 0 : 4 : 8
I	90	85 : 8 : 1 : 6
OTf	40	94 : 0 : 0 : 6

a Conditions: (*R*)-3a-X (0.1 mmol), 4-methoxyphenylmagnesium bromide (1.5 equiv.) in 1,4-dioxane at room temperature for 12 h, then H_2_O was added and stirred for 2 h.

b Isolated yield.

c Diastereomeric ratio (d.r.) determined by ^31^P-NMR and ^1^H-NMR (CDCl_3_) of the crude reaction mixture after hydrolysis with H_2_O.

**Scheme 1:**
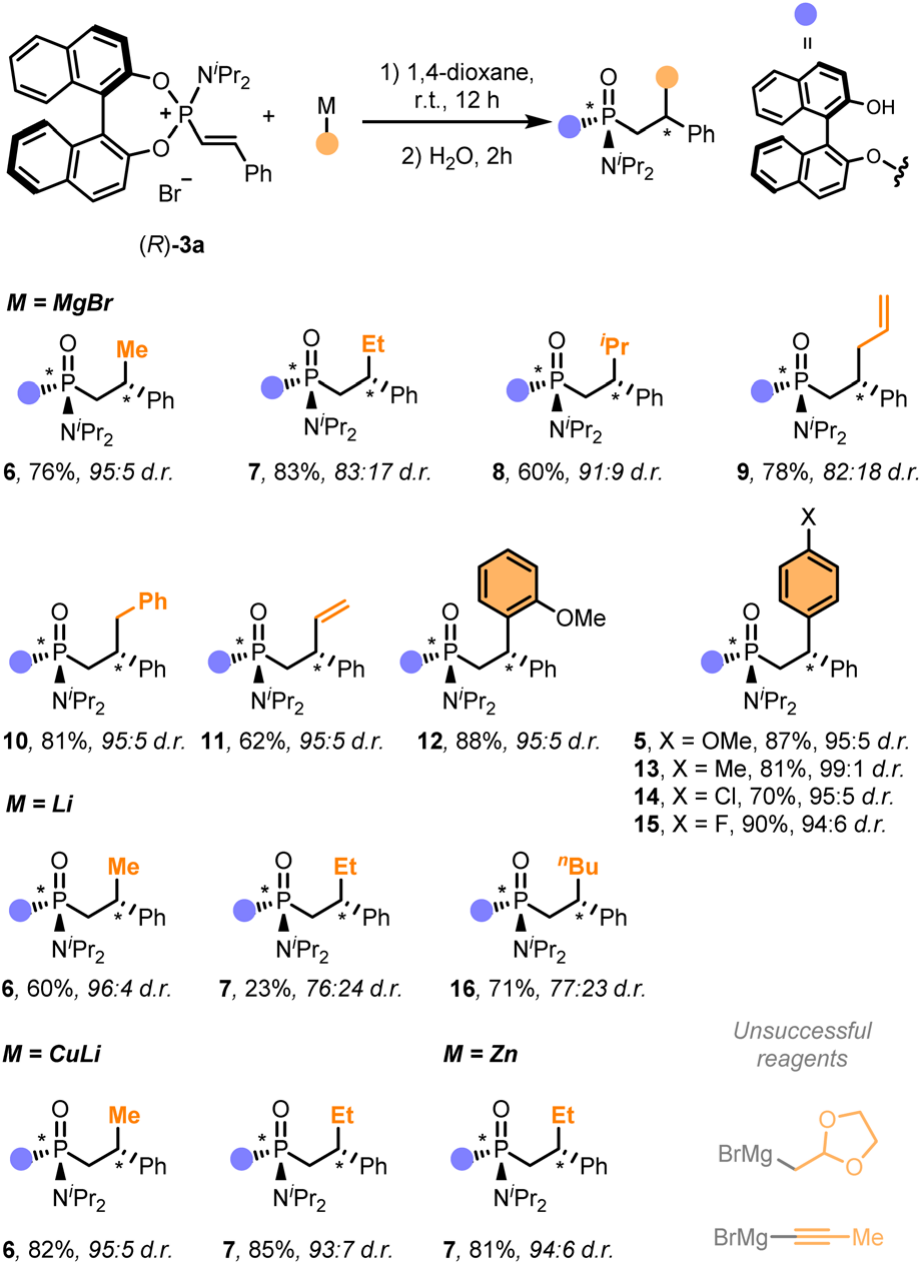
Scope of organometallic reagents. General conditions: (*R*)-3a (0.1 mmol), organometallic reagent (2.0 equiv.) in 1,4-dioxane (0.1 M) at room temperature for 18 h, then, H_2_O (1 mL) was added and stirred for 6 h. Diastereomeric ratio determined by ^31^P NMR (CDCl_3_). For the sake of clarity, the functionalities highlighted in orange indicate the moiety installed with the organometallic reagent.

We next examined the use of other commercially available organometallic reagents as nucleophiles (Scheme 1). The reaction tolerated well the use of a wide range of organomagnesium, organolithium, organocuprate and organozinc C-nucleophiles, which allowed the installation of alkyl (6–10, 16), vinyl (11) and aryl groups (5, 12–15) in good overall yields with moderate to excellent diastereoselectivities (up to >20 : 1 d.r.). Notably, arene-based nucleophiles proved to be excellent coupling partners for the synthesis of functionalised chiral diarylmethanes (5, 12–15). Curiously, while these C_sp^3^_- and C_sp^2^_-based nucleophiles cleanly reacted with alkenylphosphonium (*R*)-3a, 1-propynylmagnesium bromide, as a prototypic C_sp_-based nucleophile, failed to provide any identifiable product. A similar fate was observed with (1,3-dioxolan-2-ylmethyl)magnesium bromide.

**Scheme 2:**
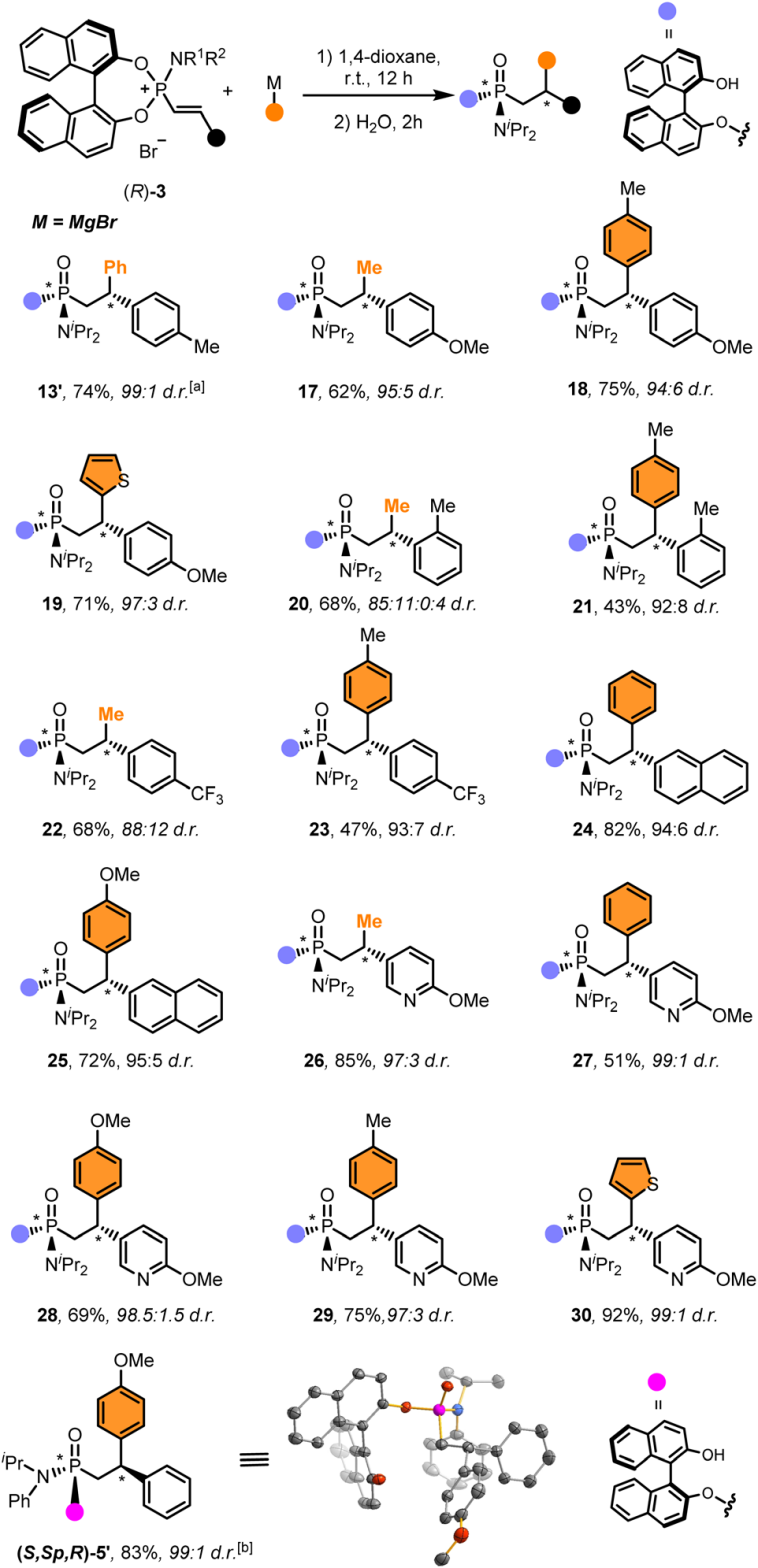
Scope of alkenylphosphonium salts. General conditions: (*R*)-3 (0.1 mmol), organometallic reagent (2.0 equiv.) in 1,4 dioxane (0.1 mL) at room temperature for 18 h, then, H_2_O (1 mL) was added and stirred for 6 h. Diastereomeric ratio determined by ^31^P NMR (CDCl_3_). For the sake of clarity, the functionalities highlighted in orange indicate the moiety installed with the organometallic reagent. ^*a*^ Compounds 13 and 13′ are epimers. (see SI for more details). ^*b*^ The (*S*,*S*_p_,*R*)-5′ was prepared following the same procedure starting from (*S*)-1b (see SI for more details).

The scope of electrophiles was subsequently explored using different alkenylphosphonium salts and Grignard reagents, which are readily available and often provide better diastereoselectivities and yields (Scheme 2).^68^ The addition reaction took place with a wide variety of alkenylphosphonium salts bearing different substitution patterns, including electron-donating groups (13, 17–21, 24, 25) as well as electron-poor substrates (22) at different positions. The reaction also tolerated well the introduction of one (26–29) or even two heteroaromatic rings (30). Remarkably, the resulting functionalised phosphonamidates were obtained in high overall yields (43–92%) and excellent stereoselectivities (>20 : 1 d.r. in most cases).

**Fig. 3:**
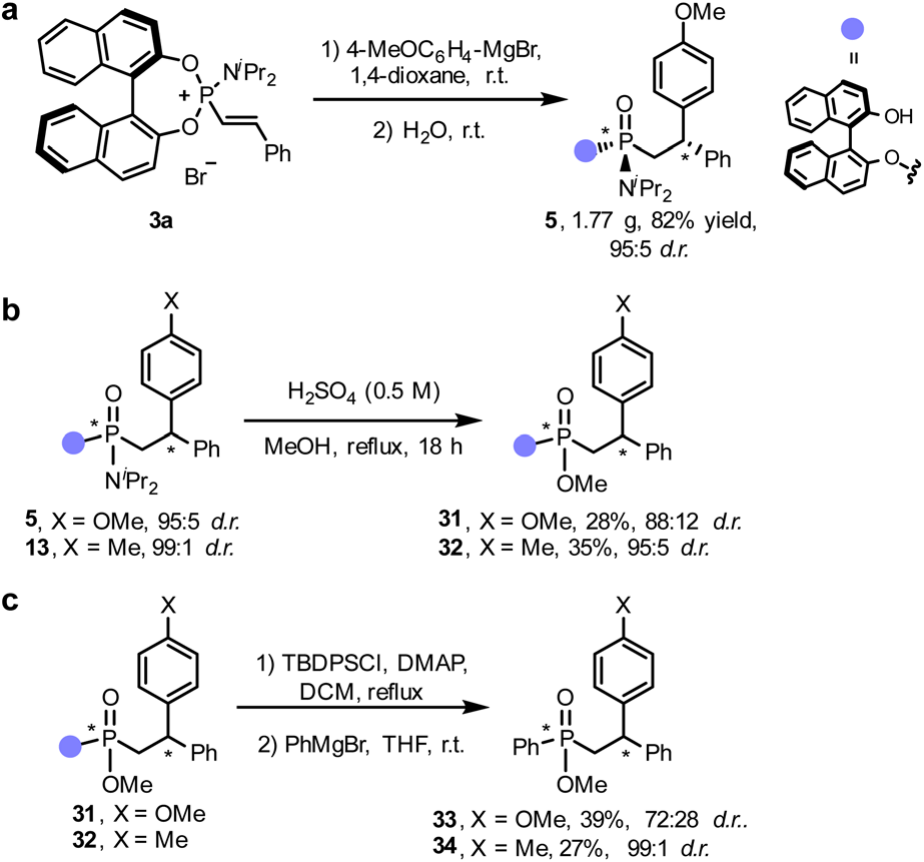
Gram-scale synthesis of 5 and synthetic manipulations of phosphonamidates.

The simplicity of this formal conjugate addition/hydrolysis approach to generate non-consecutive P- and C-stereocenters, together with the presence of multiple functionalisation sites in the final phosphonamidates led us to explore the scalability of the reaction and develop some synthetic applications. Gratifyingly, the reaction could be easily scaled up to without modifying the standard reaction conditions and compound (*R*,*R*_p_,*S*)-5 could be isolated in gram scale (1.77 g) and in excellent yield (82%) and diastereomeric purity (95 : 5 d.r., Fig. 3a). First, we demonstrated that the amine moiety of phosphonamidate (*R*,*R*_p_,*S*)-5 could be replaced by a methoxy group under acidic conditions,^27,69^ furnishing chiral phosphonate 31 in an unoptimized 28% yield and moderate diastereospecificity (88 : 12 d.r., Fig. 3b). Interestingly, upon protection of the BINOL moiety as silylether, the chiral auxiliary could be chemoselectively replaced with different arene motifs by addition of an aryl Grignard reagent. The stereospecificity in these nucleophilic substitutions was substrate-dependent, obtaining compound 33 with significant erosion of the diastereomeric purity and 34 in nearly perfect diastereospecificity (Fig. 3c).

**Fig. 4:**
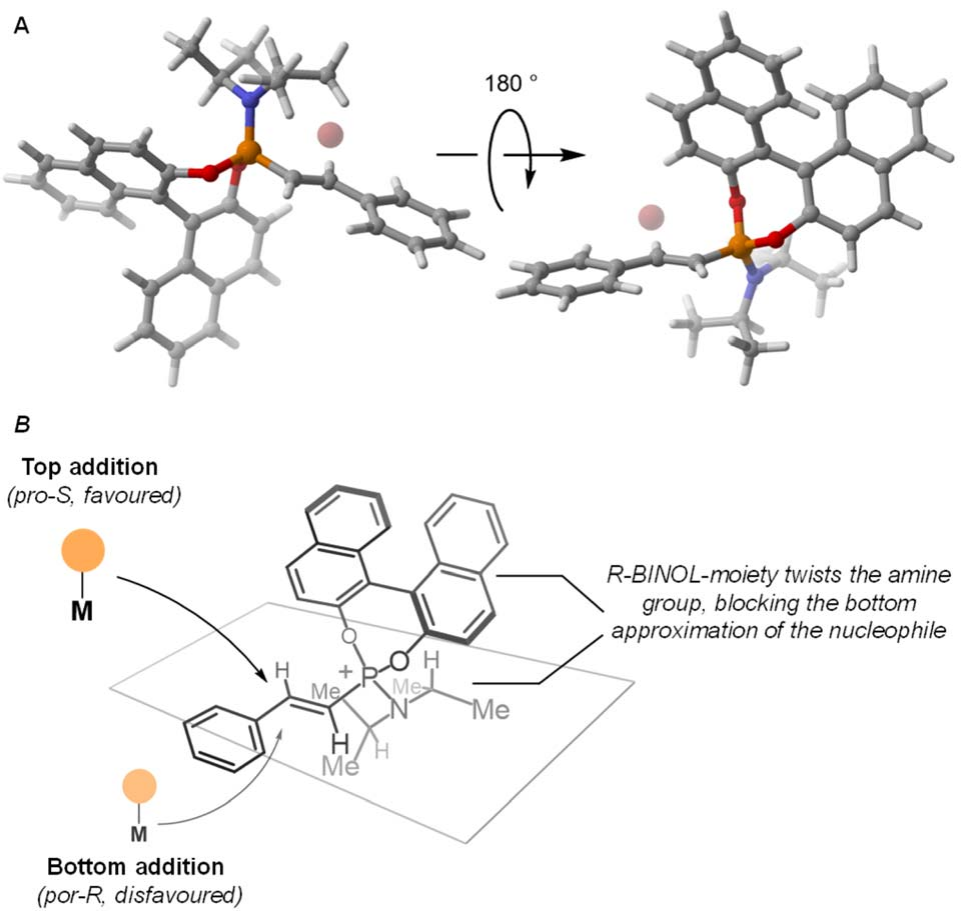
Stereochemical rationale.

The unusually high level of remote stereocontrol exerted by the BINOL auxiliary on the β-carbon led us to study in more detail the structure of the alkenylphosphonium salt. DFT-based modelling of alkenylphosphonium salt (*R*)-3a-Br (see SI for details) revealed a key synergistic role between the amine and the BINOL substituents at the phosphorus centre (Fig. 4a). The axially chiral BINOL group appears to induce a rotation of the P–N (^*i*^Pr)_2_ bond to minimize the steric repulsion between these two groups. As a consequence, the bulky iso-propyl groups of the amine fragment block one of the diastereotopic faces of the olefin, ultimately leaving only the pro-S face available for nucleophilic attack by the organometallic reagent (Fig. 4b).

## Conclusions

In summary, stereoselective additions to chiral alkenyl-P^V^ compounds have usually proved challenging to accomplish due to their low reactivity of and poor diastereoselectivity. Here, we have demonstrated that the combination of an alkenylphosphonium salt, acting as a more electrophilic alkenylphosphonamidate surrogate, and a BINOL moiety as chiral auxiliary, enabled the smooth and highly stereoselective nucleophilic addition of a wide range of C-nucleophiles to the β-carbon. This allowed us to access to functionalised P-stereogenic building-blocks featuring two non-consecutive stereocenters.

## Author contributions

Conceptualization: X.-B. C., D. P. and B. L. investigation and methodology: X.-B. C. and D. P. writing – original draft, review and editing: X.-B. C., D. P. and B. L. supervision: D. P. and B. L. funding acquisition: B. L.

## Conflicts of interest

There are no conflicts to declare.

## Supplementary Material

SC-017-D5SC09946C-s001

SC-017-D5SC09946C-s002

## Data Availability

CCDC 2526188 contains the supplementary crystallographic data for this paper.^70^ The data supporting this article have been included as part of the supplementary information (SI). Supplementary information: experimental procedures, characterisation of new compounds, computational details, crystallographic data, and extended discussion of the scope of the reaction. See DOI: https://doi.org/10.1039/d5sc09946c.
