# Supplementary material for: Stereoselective additions to alkenylphosphonium salts for the synthesis of P-stereogenic compounds
Source: Chem Sci. 2026 Feb 20;17(15):7575–81. doi: 10.1039/d5sc09946c (PMC12921701; doi:10.1039/d5sc09946c)

Supplementary Information (SI) for Chemical Science.
This journal is © The Royal Society of Chemistry 2026

Supporting Information

Stereoselective Additions to Alkenylphosphonium Salts for the Synthesis of P-stereogenic Compounds

Xiao-Bing Chen, Damián Padín, and Ben L. Feringa

Table of Contents

TABLE OF CONTENTS 2
1. GENERAL EXPERIMENTAL PROCEDURES 3
2. LIST OF STARTING MATERIALS 4
PHOSPHORAMIDITES 4
ALKENYL HALIDES 4
ALKENYLPHOSPHONIUM SALTS 4
ORGANOMETALLIC REAGENTS 4
3. OPTIMIZATION OF THE REACTION CONDITIONS 5
4. ^{31}P-NMR MONITORING OF THE REACTION PROGRESS 7
5. SYNTHESIS AND CHARACTERIZATION OF ALKENYLPHOSPHONIUM SALTS 8
6. SYNTHESIS AND CHARACTERIZATION OF PHOSPHONAMIDATES 13
7. X-RAY DIFFRACTION ANALYSIS AND DETERMINATION OF ABSOLUTE CONFIGURATION 29
8. DFT STRUCTURAL ANALYSIS OF ALKENYLPHOSPHONIUM SALT *R*-3A 31
9. DERIVATIZATION REACTIONS 34
10. CHALLENGING SUBSTRATES 39
11. REFERENCES 46
12. NMR SPECTRA 47

1. General experimental procedures

Unless otherwise specified, all reagents were obtained from commercial sources and used without further purification. Prior to use, cesium carbonate (Cs_2CO_3) was dried under a high vacuum at 70 °C overnight and stored under inert atmosphere. Dry solvents were obtained using a MBraun SPS 800 system and stored under N_2.

All reactions under N_2 atmosphere were carried out in oven-dried or heat gun-dried glassware with magnetic stirring. When specified, reactions were monitored by analytical thin layer chromatography on silica-coated aluminum plates (silica gel 60 F254 Merck) and components were visualized by UV light and $KMnO_4$ staining (1.5 g $KMnO_4$, 10 g K_2CO_3, 1.25 mL 10% NaOH, 200 mL H_2O). Flash column chromatography was performed on silica gel 60 (Merck, 230-400 mesh).

^{1}H-NMR, ^{13}C-NMR, ^{31}P-NMR and ^{19}F-NMR experiments were carried out using Varian AMX400 and Bruker Innova 600 MHz spectrometers. Chemical shift values are reported in ppm with the residual solvent resonances as the internal standards. Coupling constants (*J*) are given in Hertz (Hz). Multiplicities are reported as follows: s = singlet, d = doublet, t = triplet, q = quartet, p = pentet, m = multiplet or as a combination of them.

Reactions at temperatures higher than 25 ºC were carried out using an oil bath or heating block. High Resolution Mass spectrometry (HRMS) analysis was carried out using a LTQ Orbitrap XL (ESI+, ESI-). Optical rotations were determined with a SCHMIDT + HAENSCH Polartronic MH8 polarimeter at 589 nm and 20 °C. Specific rotation data are reported as follows: $[\alpha]_{\lambda}^{temp}$(concentration [in g/100 mL], solvent). Enantiomeric ratios were determined by HPLC analysis using a Shimadzu SPD M10AVP diode array detector using a Chiralpak columns with mixtures of HPLC-grade *n*-heptane and 2-propanol as the eluent and a column temperature of 40 °C.

2. List of starting materials

Phosphoramidites

Phosphoramidites (***R***)-**1a and** (***S***)-**1b** (Figure S 1) were prepared following our reported procedures.[1-2]

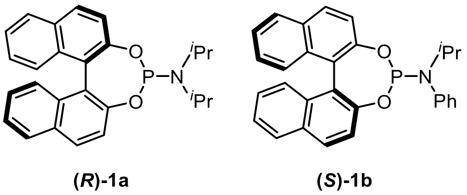

Figure S 1 List of phosphoramidites used in this study.

Alkenyl halides

Alkenyl halide **2a-Br** is commercially available and it was used as received.

Alkenyl halides **2a-Cl**, **2a-I**, **2a-I(Mes)OTf**, **2b**, **2c**, **2d**, **2e**, **2f**, **2g** were prepared following previously reported procedures.[3]

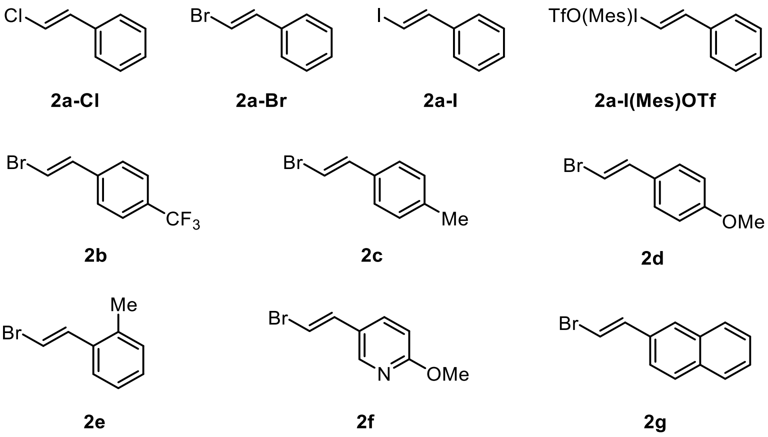

Figure S 2 List of alkenyl halides used in this study.

Alkenylphosphonium salts

The synthesis and characterization of alkenylphosphonium salts ***R*-3a** to ***R*-3g** and ***S*-3b** is presented in section 5.

Organometallic reagents

MeMgBr (3 M in Et_2O), EtMgBr (3 M in Et_2O), ^{i}PrMgBr (3 M in 2-MeTHF), VinylMgBr (1 M in THF), AllylMgBr (1 M in Et_2O), BenzylMgBr (0.9 M in THF), *p*-TolylMgBr (1 M in THF), 4-ChloroPhenylMgBr (1 M in Et_2O), 4-FluoroPhenylMgBr (1 M in THF), 4-MethoxyPhenylMgBr (1 M in THF), 2-MethoxyPhenylMgBr (1 M in THF), 2-ThienylMgBr (1 M in THF), 1-PropynylMgBr (0.5 M in THF), PhenylethynylMgBr (1 M in THF), (1,3-dioxolan-2-yl) MethylMgBr (0.5 M in THF), MeLi (1.6 M in Et_2O), EtLi (0.5 M in Benzene/Cyclohexane), ^{n}BuLi (1.6 M in Hexanes), Et_2Zn (1 M in Hexanes) were purchased from Sigma-Aldrich and TCI Chemicals. Me_2CuLi and Et_2CuLi were prepared following reported procedures.[4]

3. Optimization of the reaction conditions

General procedure A for the optimization of the reaction conditions (for Table S1): A dry vial was charged with phosphonium salt *R*-**3a** (0.10 mmol), which was prepared according to our established procedure,[2] the copper salt (10 mol%, if necessary), and a stirring bar. The vial was sealed with a septum, evacuated and backfilled with N_2 3 times. Afterwards, the solvent was added, followed by 4-methoxyphenylmagnesium bromide solution (0.15 mmol, 1.5 equiv.). The mixture was stirred at the indicated temperature for 18 h. Subsequently, an aliquot (ca. 0.1 mL) was taken and diluted with 0.5 mL of $CDCl_3$ to analyze the conversion of *R*-**3a** and distribution of products by ^{1}H- and ^{31}P-NMR. Then, the reaction mixture was hydrolyzed with H_2O, 1 M HCl (1 mL) or Cs_2CO_3 (32.6 mg in 1 mL H_2O) and stirred at room temperature overnight (see Table S1). The mixture was then extracted with DCM (3x), and the combined organic extracts were dried over Na_2SO_4, filtered, and concentrated under reduced pressure. When applicable, the crude material was purified by flash column chromatography on silica gel using hexane/AcOEt 3:1.

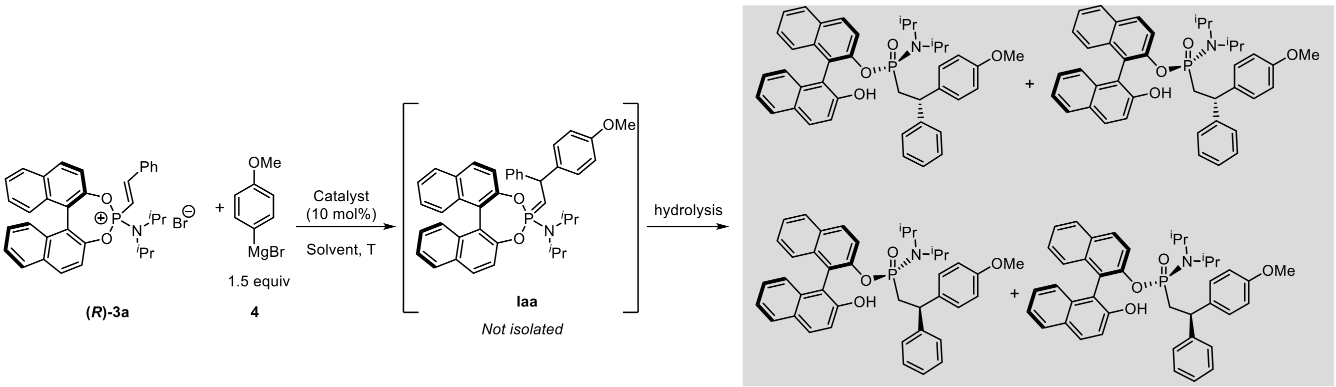

Table S1. Optimization of the reaction conditions.[a][b]

Entry	[Cu]	Solvent	Conc (M)	T (°C)	Hydrolysis conditions	Conversion of *R*-3a (%)	*d.r.*
1	-	THF	0.1	25	HCl	100	14:1:10:75
2[c]	-	THF	0.1	25	HCl	90	17:2:8:73
3	-	Toluene	0.1	25	HCl	100	52:9:5:34
4	-	1,4-dioxane	0.1	25	HCl	100	60:3:2:35
5	-	Et_2O	0.1	25	HCl	100	70:10:3:17
6	-	DMF	0.1	25	HCl	trace	-
7	-	DCE	0.1	25	HCl	100	35:5:6:54
8	-	2-MeTHF	0.1	25	HCl	100	51:6:6:37
9	-	MTBE	0.1	25	HCl	100	67:11:3:19
10	-	THF	0.1	0	HCl	83.5	11:2:10:76

11	-	THF	0.1	-78	HCl	35	21:3:10:66
12	-	THF	0.025	25	HCl	25	18:6:9:67
13	-	THF	0.05	25	HCl	45	15:3:9:73
14	-	THF	0.2	25	HCl	76	16:3:11:70
15	CuBr	THF	0.1	25	HCl	99	12:2:15:71
16	CuI	THF	0.1	25	HCl	99	12:3:16:69
17	$CuBr\cdot SMe_2$	THF	0.1	25	HCl	100	14:1:10:75
18	CuCN	THF	0.1	25	HCl	76	16:3:11:70
19	$Cu(OAc)_2$	THF	0.1	25	HCl	99	15:6:14:65
20	$CuCl_2$	THF	0.1	25	HCl	71	15:7:11:67
21	-	THF	0.1	25	Cs_2CO_3 (2 eq)	90	63:7:3:27
22	-	THF	0.1	25	H_2O	92	81:11:1:7
23	-	Et_2O	0.1	25	H_2O	95	78:11:2:9
24	-	1,4-dioxane	0.1	25	H_2O	98(87[d])	88:0:4:8

[a] All reactions were performed under N_2 atmosphere with (*R*)-**3a** (0.1 mmol) and **4** (1.5 equiv.) in the presence of the indicated metal complex (10 mol %), and solvent at the indicated temperature for 18 h. [b] Conversion and diastereomeric ratio (*d.r.*) were determined by ^{31}P-NMR and ^{1}H-NMR ($CDCl_3$) of the crude reaction mixture after hydrolysis (1 mL of H_2O/1 mL of 1 N $HCl_{aq.}$/Cs_2CO_3). [c] **4** was added by syringe pump (0.5 mL in 30 min) at 25°C. [d] Isolated yield.

4. ^{31}P-NMR monitoring of the reaction progress

The reaction progress could be conveniently monitored by ^{31}P-NMR (proton decoupled) by taking a small aliquot (ca. 100 µL) and diluting it with $CDCl_3$ (ca. 500 µL) (Figure S 3).

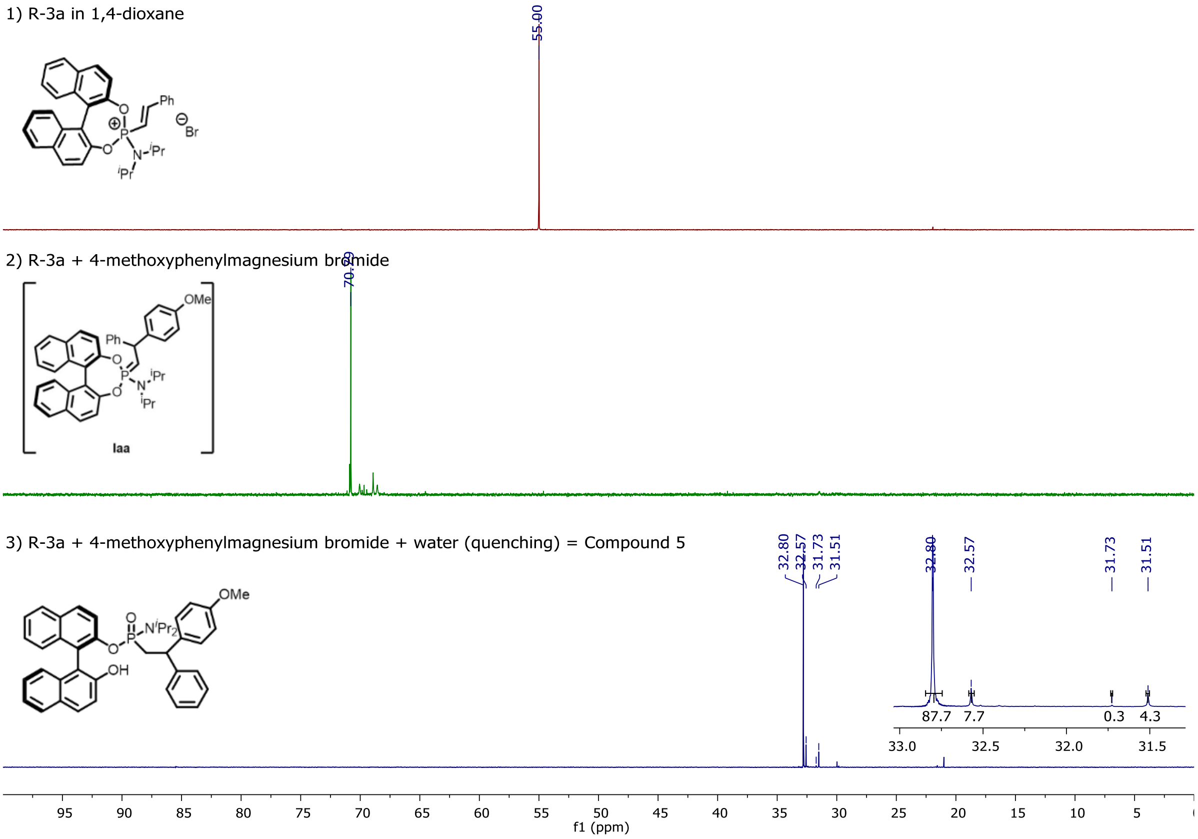

Figure S 3 ^{31}P-NMR spectra of the products at the different stages of the reaction. 1) Starting phosphonium salt *R*-**3a** before addition of the Grignard reagent. 2) After addition of the Grignard reagent (ylide intermediate **Iaa**). 3) After quenching the reaction with H_2O (hydrolysis), which corresponds to compound **5**.

5. Synthesis and characterization of alkenylphosphonium salts

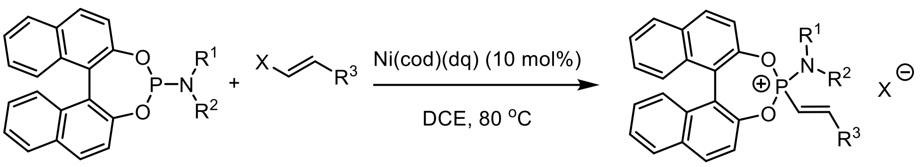

3

General procedure B for the preparation of alkenylphosphonium salts

For preparation of alkenylphosphonium salt **3**. A dry vial was charged with phosphonamidate (5 mmol, 1.0 equiv.), Ni(cod)(dq) (0.17 g, 0.5 mmol, 0.1 equiv.), and a stirring bar. Note: Both Ni(cod)(dq) and Ni(cod)$_2$ worked and gave similar results. The vial was sealed, evacuated, and backfilled with N_2 3 times. Next, DCE (30 mL) was added via syringe, followed by *trans*-β-halostyrene (5.5 mmol, 1.1 equiv.) via syringe. The mixture was stirred at 80 ºC for 18 h. Subsequently, the reaction mixture was allowed to cool to room temperature. The solution was concentrated under reduced pressure to afford **3** as a green solid. NOTE: The resulting solid might remain pale green, indicating possible contamination with Ni salts. The phosphonium salt proved to be reasonably stable under air, although water is known to hydrolyze it. Most of the Ni contamination can be removed by dissolving the green solid in DCM and quickly washing it with ice-cold water (30 mL, 3 times, to avoid hydrolysis). The organic solution was dried over Na_2SO_4, filtered, and concentrated under reduced pressure to afford a white solid. Minimal amounts of hydrolyzed product can be removed by washing this white solid with ethyl acetate, obtaining **3** as a white solid. NOTE: The NMR data and reactivity of the green and white solids were the same.

(*E*)-4-(diisopropylamino)-4-styryldinaphtho[2,1-*d*:1',2'-*f*][1,3,2]dioxaphosphepin-4-ium bromide (*R*-3a)

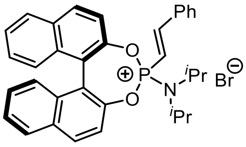

***R*-3a**

Following General Procedure B. Purification by recrystallization (DCM/EtOAc) afforded 2.2 g (3.65 mmol, 73%) of the title compound as white solid. Characterization data matched with those in previous report.[3]

(*E*)-4-(diisopropylamino)-4-(4-methylstyryl)dinaphtho[2,1-*d*:1',2'-*f*][1,3,2]dioxaphosphepin-4-ium bromide (*R*-3b)

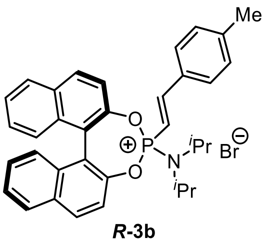

***R*-3b**

Following General Procedure B. Purification by recrystallization (DCM/EtOAc) afforded 2.3 g (3.75 mmol, 75%) of the title compound as a white solid.

Specific rotation: $[\alpha]_D^{20} = 481.7$ (c = 0.71, CH_2Cl_2).

^{1}H NMR (400 MHz, $CDCl_3$) δ 8.17 (d, *J* = 8.9 Hz, 1H), 8.03 (s, 3H), 7.93 (d, *J* = 8.3 Hz, 1H), 7.74 (q, *J* = 9.6, 8.7 Hz, 3H), 7.69 – 7.55 (m, 3H), 7.52 (t, *J* = 7.5 Hz, 1H), 7.43 – 7.37 (m, 2H), 7.33 (t, *J* = 7.6 Hz, 1H), 7.30 – 7.24 (m, 1H), 7.21 (d, *J* = 7.8 Hz, 2H), 4.00 – 3.78 (m, 2H), 2.34 (s, 3H), 1.51 (d, *J* = 6.7 Hz, 6H), 1.08 (d, *J* = 6.8 Hz, 6H).

^{13}C NMR (101 MHz, $CDCl_3$) δ 160.2 (d, *J* = 4.6 Hz), 144.8 (d, *J* = 8.5 Hz), 144.3, 144.2 (d, *J* = 10.8 Hz), 132.7, 132.4 (d, *J* = 1.5 Hz), 132.28 (d, *J* = 1.4 Hz), 132.25 (d, *J* = 1.5 Hz), 132.1 (d, *J* = 1.4 Hz), 131.9 (d, *J* = 1.7 Hz), 130.39 (d, *J* = 24.9 Hz), 130.35, 130.1, 128.9 (d, *J* = 8.5 Hz), 127.9, 127.5, 127.1, 127.0, 126.9, 126.8, 121.8 (d, *J* = 2.6 Hz), 120.885 (d, *J* = 1.9 Hz), 120.882 (d, *J* = 7.6 Hz), 102.2 (d, *J* = 177.5 Hz), 49.5 (d, *J* = 5.4 Hz), 22.9 (d, *J* = 2.1 Hz), 22.5 (d, *J* = 1.6 Hz), 21.8.
^{31}P-NMR (162 MHz, $CDCl_3$) δ 55.58.
HRMS (ESI+, m/z) calculated for $C_{35}H_{35}NO_2P$ $[M]^+$: 532.2400 found 532.2393.

(*E*)-4-(diisopropylamino)-4-(4-methoxystyryl)dinaphtho[2,1-*d*:1',2'-*f*][1,3,2]dioxaphosphepin-4-ium bromide (*R*-3c)

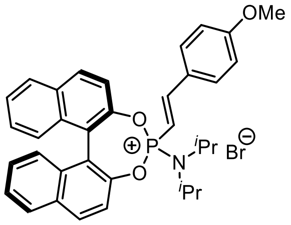

***R*-3c**

Following General Procedure B. Purification by recrystallization (DCM/EtOAc) afforded 2.5 g (4.0 mmol, 80%) of the title compound as a yellow solid.
Specific rotation: $[\alpha]_D^{20} = 53.6$ (c = 0.47, CH_2Cl_2).
^{1}H NMR (400 MHz, $CDCl_3$) δ 8.16 (d, *J* = 8.9 Hz, 1H), 8.07 – 7.97 (m, 2H), 7.98 – 7.88 (m, 2H), 7.84 (d, *J* = 8.2 Hz, 2H), 7.68 – 7.45 (m, 5H), 7.44 – 7.29 (m, 3H), 6.90 (d, *J* = 8.3 Hz, 2H), 3.91 – 3.81 (m, 2H), 3.79 (s, 3H), 1.49 (d, *J* = 6.7 Hz, 6H), 1.05 (d, *J* = 6.8 Hz, 6H).
^{13}C NMR (101 MHz, $CDCl_3$)δ 163.8, 159.8 (d, *J* = 5.2 Hz), 144.8 (d, *J* = 8.5 Hz), 144.3 (d, *J* = 10.7 Hz), 132.6, 132.4 (d, *J* = 46.0 Hz), 132.4 (d, *J* = 1.6 Hz), 132.3 (d, *J* = 1.5 Hz), 132.1 (d, *J* = 1.3 Hz), 131.9 (d, *J* = 1.6 Hz), 128.9 (d, *J* = 9.6 Hz), 127.8, 127.5, 127.1, 127.0, 126.9, 126.8, 126.0 (d, *J* = 25.4 Hz), 121.8 (d, *J* = 2.6 Hz), 120.94 (d, *J* = 2.6 Hz), 120.85 (d, *J* = 3.2 Hz), 119.8 (d, *J* = 2.8 Hz), 114.8, 99.5 (d, *J* = 179.2 Hz), 55.7, 49.4 (d, *J* = 5.5 Hz), 22.8 (d, *J* = 2.2 Hz), 22.5 (d, *J* = 1.6 Hz).
^{31}P-NMR (162 MHz, $CDCl_3$) δ 56.36.
HRMS (ESI+, m/z) calculated for $C_{35}H_{35}NO_3P$ $[M]^+$: 548.2349 found 548.2340.

(*E*)-4-(diisopropylamino)-4-(4-(trifluoromethyl)styryl)dinaphtho[2,1-*d*:1',2'-*f*][1,3,2]dioxaphosphepin-4-ium bromide (*R*-3d)

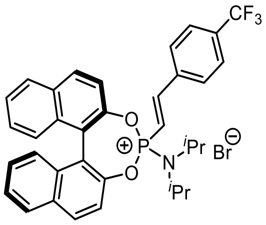

***R*-3d**

Following General Procedure B. Purification by recrystallization (DCM/EtOAc) afforded 2.1 g (3.15 mmol, 63%) of the title compound as a yellow solid.
Specific rotation: $[\alpha]_D^{20} = 55.8$ (c = 0.52, CH_2Cl_2).
^{1}H NMR (400 MHz, $CDCl_3$) δ 8.16 (d, *J* = 8.8 Hz, 1H), 8.08 – 7.96 (m, 4H), 7.87 (d, *J* = 7.7 Hz, 2H), 7.83 – 7.69 (m, 2H), 7.58 (d, *J* = 8.5 Hz, 3H), 7.50 (d, *J* = 7.6 Hz, 2H), 7.39 (d, *J* = 7.6 Hz, 2H), 7.32 (t, *J* = 7.5 Hz, 2H), 4.04 – 3.67 (m, 2H), 1.46 (d, *J* = 6.5 Hz, 6H), 1.01 (d, *J* = 6.7 Hz, 6H).

^{13}C NMR (151 MHz, $CDCl_3$) δ 158.0 (d, *J* = 4.2 Hz), 152.8 (d, *J* = 36.6 Hz), 144.8 (d, *J* = 8.6 Hz), 144.0 (d, *J* = 10.8 Hz), 133.9, 132.6 (d, *J* = 59.3 Hz), 132.5 (d, *J* = 1.4 Hz), 132.25 (d, *J* = 1.6 Hz), 132.18 (d, *J* = 1.3 Hz), 131.8 (d, *J* = 1.5 Hz), 130.6, 128.9 (d, *J* = 14.6 Hz), 127.8 (d, *J* = 25.1 Hz), 127.5, 127.0 (d, *J* = 28.3 Hz), 126.1 (d, *J* = 4.1 Hz), 125.5 (d, *J* = 3.7 Hz), 124.7 (d, *J* = 3.4 Hz), 123.3 (d, *J* = 17.5 Hz), 121.9 (d, *J* = 2.6 Hz), 120.8 (dd, *J* = 5.5, 2.9 Hz), 119.7 (d, *J* = 2.6 Hz), 118.7 (d, *J* = 79.8 Hz), 114.8, 112.5, 108.2 (d, *J* = 173.8 Hz), 49.6 (d, *J* = 5.5 Hz), 46.9 (d, *J* = 140.5 Hz), 22.7 (d, *J* = 42.0 Hz), 22.6 (d, *J* = 99.1 Hz), 18.8 (d, *J* = 3.0 Hz). Due to C−P and C-F coupling and the complexity of the spectrum, some doublets cannot be assigned and they are listed as singlets.
^{31}P-NMR (162 MHz, $CDCl_3$) δ 53.91.
^{19}F NMR (376 MHz, $CDCl_3$) δ -63.19.
HRMS (ESI+, m/z) calculated for $C_{35}H_{32}F_3NO_2P$ $[M]^+$: 586.2117 found 586.2106.

(*E*)-4-(diisopropylamino)-4-(2-methylstyryl)dinaphtho[2,1-*d*:1',2'-*f*][1,3,2]dioxaphosphepin-4-ium bromide (*R*-3e)

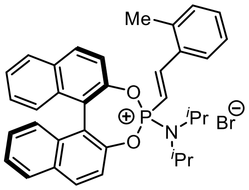

***R*-3e**

Following General Procedure B. Purification by recrystallization (DCM/EtOAc) afforded 1.4 g (2.35 mmol, 47%) of the title compound as a yellow solid.
Specific rotation: $[\alpha]_D^{20}$ = -34.5 (c = 0.11, CH_2Cl_2).
^{1}H NMR (400 MHz, $CDCl_3$) δ 8.17 (d, *J* = 9.1 Hz, 1H), 8.01 (t, 2H), 7.93 (d, *J* = 8.8 Hz, 2H), 7.80 – 7.73 (m, 1H), 7.66 (d, *J* = 8.9 Hz, 1H), 7.59 (t, *J* = 6.9 Hz, 1H), 7.39 (t, *J* = 8.5 Hz, 2H), 7.35 – 7.29 (m, 3H), 7.21 (dd, *J* = 12.9, 7.9 Hz, 1H), 7.11 (dd, *J* = 16.8, 7.9 Hz, 2H), 3.97 – 3.81 (m, 2H), 2.08 (s, 3H), 1.46 (d, *J* = 6.7 Hz, 6H), 1.08 (d, *J* = 6.7 Hz, 6H).
^{13}C NMR (101 MHz, $CDCl_3$) δ 157.5 (d, *J* = 4.9 Hz), 153.0, 144.6 (d, *J* = 8.5 Hz), 144.2 (d, *J* = 10.9 Hz), 138.7, 133.1, 132.7, 132.4, 132.2 (dd, *J* = 2.9, 1.4 Hz), 131.9 (d, *J* = 1.6 Hz), 131.7, 131.5, 131.0 (d, *J* = 1.7 Hz), 130.6, 128.9, 128.6, 128.2, 127.9, 127.6, 127.4, 127.04 (d, *J* = 4.0 Hz), 126.9 (d, *J* = 5.7 Hz), 124.1 (d, *J* = 123.5 Hz), 121.6 (d, *J* = 2.6 Hz), 121.0 (d, *J* = 2.6 Hz), 120.6 (d, *J* = 3.1 Hz), 119.8 (d, *J* = 2.8 Hz), 118.3, 112.3, 104.3 (d, *J* = 177.0 Hz), 49.5 (d, *J* = 5.3 Hz), 22.8 (d, *J* = 2.1 Hz), 22.5 (d, *J* = 1.6 Hz), 19.4, 18.9 (d, *J* = 2.0 Hz).
^{31}P-NMR (162 MHz, $CDCl_3$) δ 54.63.
HRMS (ESI+, m/z) calculated for $C_{35}H_{35}NO_2P[M]^+$: 532.2400 found 532.2397.

(*E*)-4-(diisopropylamino)-4-(2-(naphthalen-2-yl)vinyl)dinaphtho[2,1-*d*:1',2'-*f*][1,3,2]dioxaphosphepin-4-ium bromide (*R*-3f)

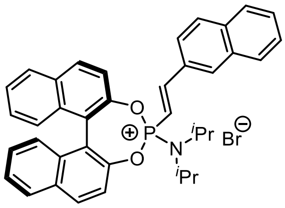

***R*-3f**

Following General Procedure B. Purification by recrystallization (DCM/EtOAc) afforded 2.5 g (3.85 mmol, 77%) of the title compound as a yellow solid.
Specific rotation: $[\alpha]_D^{20}$ = 74.2 (c = 0.66, CH_2Cl_2).

^{1}H NMR (400 MHz, $CDCl_3$) δ 8.32 – 8.10 (m, 3H), 8.02 (dd, *J* = 15.4, 8.2 Hz, 4H), 7.95 – 7.83 (m, 4H), 7.78 (d, *J* = 8.2 Hz, 1H), 7.67 – 7.56 (m, 2H), 7.55 – 7.37 (m, 5H), 7.34 (t, *J* = 7.7 Hz, 1H), 7.29 (s, 1H), 4.01 – 3.80 (m, 2H), 1.54 (d, *J* = 6.5 Hz, 6H), 1.09 (d, *J* = 6.7 Hz, 6H).

^{13}C NMR (101 MHz, $CDCl_3$) δ 160.2 (d, *J* = 4.6 Hz), 144.8 (d, *J* = 8.5 Hz), 144.2 (d, *J* = 10.7 Hz), 135.5, 133.4, 132.8 (d, *J* = 1.2 Hz), 132.5 (d, *J* = 45.8 Hz), 132.4 (d, *J* = 1.5 Hz), 132.3 (d, *J* = 1.5 Hz), 132.1 (d, *J* = 1.3 Hz), 131.9 (d, *J* = 1.5 Hz), 130.7, 130.5, 129.5, 129.4, 129.0, 128.9, 128.7,J 127.9, 127.8, 127.5, 127.1 (d, *J* = 4.8 Hz), 126.9 (d, *J* = 4.5 Hz), 126.8, 124.5, 121.8 (d, *J* = 2.6 Hz), 120.9 (d, *J* = 2.6 Hz), 120.8 (d, *J* = 3.3 Hz), 119.8 (d, *J* = 2.8 Hz), 103.8 (d, *J* = 176.1 Hz), 49.5 (d, *J* = 5.3 Hz), 22.9 (d, *J* = 2.0 Hz), 22.5 (d, *J* = 1.6 Hz).

^{31}P-NMR (162 MHz, $CDCl_3$) δ 55.51.

HRMS (ESI+, m/z) calculated for $C_{38}H_{35}NO_2P[M]^+$: 568.2400 found 568.2393.

(*E*)-4-(diisopropylamino)-4-(2-(6-methoxypyridin-3-yl)vinyl)dinaphtho[2,1-*d*:1',2'-*f*][1,3,2]dioxaphosphepin-4-ium bromide (*R*-3g)

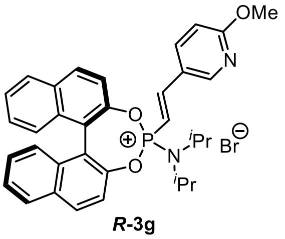

***R*-3g**

Following General Procedure B. Purification by recrystallization (DCM/EtOAc) afforded 2.6 g (4.10 mmol, 82%) of the title compound as a yellow solid.

Specific rotation: $[\alpha]_D^{20}$ = -6.9 (c = 0.52, CH_2Cl_2).

^{1}H NMR (400 MHz, $CDCl_3$) δ 9.00 (s, 1H), 8.27 (dd, *J* = 24.3, 17.1 Hz, 1H), 8.16 (d, *J* = 8.9 Hz, 1H), 8.11 – 7.98 (m, 4H), 7.92 (d, *J* = 8.2 Hz, 1H), 7.65 – 7.44 (m, 4H), 7.40 (d, *J* = 3.9 Hz, 2H), 7.37 – 7.30 (m, 1H), 7.30 – 7.21 (m, 1H), 6.88 (s, 1H), 3.91 (s, 3H), 3.89 – 3.78 (m, 2H), 1.56 (d, *J* = 6.7 Hz, 6H), 1.06 (d, *J* = 6.7 Hz, 6H).

^{13}C NMR (101 MHz, $CDCl_3$) δ 156.5, 151.0, 144.8 (d, *J* = 8.5 Hz), 144.2, 144.1, 139.3, 132.41 (d, *J* = 1.5 Hz), 132.40 (d, *J* = 48.9 Hz), 132.2 (d, *J* = 1.6 Hz), 132.1 (d, *J* = 1.3 Hz), 131.8 (d, *J* = 1.6 Hz), 128.9, 128.8, 127.8, 127.4, 127.1, 127.0, 126.8, 126.7, 121.8 (d, *J* = 2.6 Hz), 121.1 (d, *J* = 3.1 Hz), 120.9 (d, *J* = 2.6 Hz), 119.7 (d, *J* = 2.7 Hz), 112.6, 103.0 (d, *J* = 175.5 Hz), 54.1, 49.5 (d, *J* = 5.4 Hz), 22.9 (d, *J* = 2.2 Hz), 22.5 (d, *J* = 1.6 Hz).

^{31}P-NMR (162 MHz, $CDCl_3$) δ 55.57.

HRMS (ESI+, m/z) calculated for $C_{34}H_{34}N_2O_3P$ $[M]^+$: 549.2302 found 549.2295.

(*E*)-4-(diisopropylamino)-4-styryldinaphtho[2,1-*d*:1',2'-*f*][1,3,2]dioxaphosphepin-4-ium iodide (*R*-3a-I)

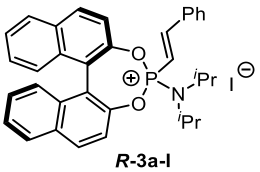

***R*-3a-I**

Following General Procedure B by using *trans*-β-iodostyrene. Purification by recrystallization (DCM/EtOAc) afforded 2.8 g (4.4 mmol, 88%) of the title compound as a white solid.

Specific rotation: $[\alpha]_D^{20}$ = 9.0 (c = 0.42, CH_2Cl_2).

^{1}H NMR (400 MHz, $CDCl_3$) δ 8.21 (d, *J* = 8.9 Hz, 1H), 8.06 (t, *J* = 9.4 Hz, 2H), 7.98 – 7.89 (m, 2H), 7.83 – 7.77 (m, 3H), 7.66 (dd, *J* = 8.9, 1.1 Hz, 1H), 7.60 (ddd, *J* = 8.1, 6.2, 1.8 Hz, 1H), 7.57 – 7.50 (m, 1H), 7.48 – 7.43 (m, 2H), 7.42 – 7.39 (m, 2H), 7.38 – 7.30 (m, 2H), 7.27 (d, *J* = 10.2 Hz, 2H), 3.98 – 3.81 (m, 2H), 1.50 (d, *J* = 6.7 Hz, 6H), 1.08 (d, *J* = 6.7 Hz, 6H).

^{13}C NMR (151 MHz, $CDCl_3$) δ 160.7 (d, *J* = 4.5 Hz), 144.6 (d, *J* = 8.6 Hz), 144.1 (d, *J* = 10.8 Hz), 133.4, 133.0, 132.8 (d, *J* = 24.6 Hz), 132.5, 132.2 (d, *J* = 6.5 Hz), 132.0, 130.3, 129.5, 129.0, 129.0, 128.0, 127.6, 127.13, 127.06, 127.0, 126.9, 121.8 (d, *J* = 2.6 Hz), 121.0 (d, *J* = 2.6 Hz), 120.6 (d, *J* = 3.2 Hz), 119.9 (d, *J* = 2.7 Hz), 103.4 (d, *J* = 178.8 Hz), 49.6 (d, *J* = 5.3 Hz), 23.0 (d, *J* = 1.8 Hz), 22.6.

^{31}P-NMR (162 MHz, $CDCl_3$) δ 54.56.

HRMS (ESI+, m/z) calculated for $C_{37}H_{31}NO_2P$ $[M]^+$: 518.2243, found 518.2234.

(*E*)-4-(isopropyl(phenyl)amino)-4-styryldinaphtho[2,1-*d*:1',2'-*f*][1,3,2]dioxaphosphepin-4-ium iodide (*S*-3a'-I)

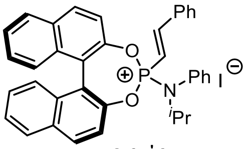

***S*-3a'-I**

Following General Procedure B by using *trans*-β-iodostyrene. Purification by recrystallization (DCM/EtOAc) afforded 1.3 g (1.8 mmol, 37%) of the title compound as a white solid.

Specific rotation: $[\alpha]_D^{20} = 23.3$ (c = 0.3, CH_2Cl_2).

^{1}H NMR (400 MHz, $CDCl_3$) δ 8.30 (d, *J* = 8.9 Hz, 1H), 8.16 – 8.00 (m, 3H), 7.95 (d, *J* = 8.3 Hz, 1H), 7.88 – 7.78 (m, 1H), 7.70 – 7.60 (m, 2H), 7.57 (d, *J* = 8.2 Hz, 2H), 7.53 (d, *J* = 8.0 Hz, 2H), 7.48 – 7.43 (m, 3H), 7.40 (d, *J* = 8.6 Hz, 3H), 6.94 (dd, *J* = 25.7, 17.3 Hz, 1H), 4.11 (hept, *J* = 8.5, 6.6 Hz, 1H), 1.35 (d, *J* = 6.6 Hz, 3H), 0.97 (d, *J* = 6.6 Hz, 3H).

^{13}C NMR (151 MHz, $CDCl_3$) δ 160.82 (d, *J* = 4.5 Hz), 145.09 (d, *J* = 8.7 Hz), 144.30 (d, *J* = 10.8 Hz), 133.44, 133.04, 132.82, 132.61, 132.51, 132.44, 132.36, 132.33, 132.25, 132.23, 131.90, 131.39 (d, *J* = 5.7 Hz), 130.44 (d, *J* = 1.8 Hz), 130.26 (d, *J* = 2.2 Hz), 129.76 (d, *J* = 102.3 Hz), 129.05 (d, *J* = 4.8 Hz), 128.03, 127.66, 127.28 (d, *J* = 4.0 Hz), 126.98 (d, *J* = 6.7 Hz), 121.45 (d, *J* = 2.5 Hz), 120.78 (d, *J* = 3.4 Hz), 120.69 (d, *J* = 2.3 Hz), 119.59 (d, *J* = 2.8 Hz), 101.20 (d, *J* = 182.6 Hz), 53.61 (d, *J* = 3.1 Hz), 22.58 (d, *J* = 3.3 Hz), 22.05 (d, *J* = 2.2 Hz).

^{31}P-NMR (162 MHz, $CDCl_3$) δ 47.82.

HRMS (ESI+, m/z) calculated for $C_{37}H_{31}NO_2P$ $[M]^+$: 552.2087, found 552.2075

6. Synthesis and characterization of phosphonamidates

General procedure C for conjugate addition of organometallic reagents to alkenylphosphonium salts followed by hydrolysis: A dry vial was charged with phosphonium salt **3** (0.1 mmol) and a stirring bar. The vial was sealed with a septum, evacuated and backfilled with N_2 3 times. Next, 1,4-dioxane (0.9 mL) was added, followed by the organometallic reagent (0.2 mmol, 2.0 eq). The mixture was stirred at room temperature for 18 h. Then, the reaction mixture was hydrolyzed with H_2O (1 mL) and stirred at room temperature for 8-12 h. The reaction mixture was then extracted with DCM (3 x 10 mL), and combined organic extracts were dried over Na_2SO_4, filtered, and concentrated under reduced pressure. When applicable, the crude material was purified by flash column chromatography on silica gel using hexane/AcOEt 3:1.

(*R*)-2'-hydroxy-[1,1'-binaphthalen]-2-yl (*R*)-*N, N*-diisopropyl-*P*-((*S*)-2-(4-methoxyphenyl)-2-phenylethyl)phosphonamidate (5)

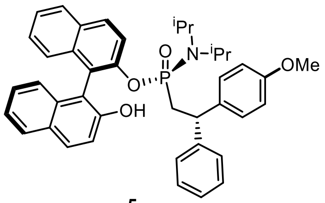

5

Following General Procedure C on 0.1 mmol scale using *R*-**3a** and 4-methoxyphenylmagnesium bromide solution (1 M in THF). Purification by flash chromatography (3:1 hexanes/EtOAc) afforded 56.0 mg (87%) of the title compound as a a white solid.

Diastereomeric purity: 88:0:4:8 *d.r.* (crude, ^{31}P-NMR in $CDCl_3$), 95:5 *d.r.* (after purification, ^{31}P-NMR in $CDCl_3$).

Specific rotation: $[\alpha]_D^{20} = 74.9$ (c = 1.91, CH_2Cl_2).

^{1}H NMR (400 MHz, $CDCl_3$) δ 7.92 (t, *J* = 9.0 Hz, 2H), 7.83 (dd, *J* = 11.7, 9.2 Hz, 3H), 7.50 – 7.39 (m, 1H), 7.37 – 7.27 (m, 4H), 7.25 – 7.18 (m, 3H), 7.12 (d, *J* = 8.2 Hz, 3H), 7.08 (d, *J* = 8.4 Hz, 1H), 6.80 (d, *J* = 8.7 Hz, 2H), 6.68 (d, *J* = 8.7 Hz, 2H), 4.17 – 3.94 (m, 1H), 3.69 (s, 3H), 3.22 – 2.89 (m, 2H), 2.27 (td, *J* = 15.3, 7.4 Hz, 1H), 1.99 (td, *J* = 16.8, 15.8, 6.3 Hz, 1H), 1.02 (d, *J* = 6.7 Hz, 6H), 0.96 (d, *J* = 6.8 Hz, 6H).

^{13}C NMR (101 MHz, $CDCl_3$) δ 157.9, 152.3, 148.1 (d, *J* = 9.2 Hz), 144.8 (d, *J* = 8.5 Hz), 137.2 (d, *J* = 10.3 Hz), 134.0, 133.9, 131.2, 130.3, 130.1, 129.4, 128.6, 128.5, 128.24, 128.17, 127.9, 127.2, 126.6, 126.3, 125.7, 125.5, 125.0, 123.6, 122.1 (d, *J* = 5.9 Hz), 121.4 (d, *J* = 2.7 Hz), 119.7, 116.7, 113.7, 55.2, 46.3 (d, *J* = 4.6 Hz), 44.2 (d, *J* = 2.3 Hz), 34.5 (d, *J* = 128.1 Hz), 22.9 (d, *J* = 1.5 Hz), 22.4 (d, *J* = 2.0 Hz).

^{31}P-NMR (162 MHz, $CDCl_3$) δ 33.16.

HRMS (ESI+, m/z) calculated for $C_{41}H_{42}NO_4PNa$ $[M+Na]^+$: 666.2744 found 666.2746.

(*S*)-2'-hydroxy-[1,1'-binaphthalen]-2-yl (*S*)-N-isopropyl-*P*-((*R*)-2-(4-methoxyphenyl)-2-phenylethyl)-N-phenylphosphonamidate (*S,Sp,R*)-5'

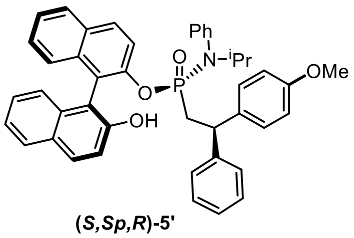

(*S,Sp,R*)-5'

Following General Procedure C on 0.1 mmol scale using ***S*-3a'-I** and 4-methoxyphenylmagnesium bromide solution (1 M in THF). Purification by flash chromatography (3:1 hexanes/EtOAc) afforded 56.2 mg (83%) of the title compound as a a white solid.

Diastereomeric purity: 94:0:0:6 *d.r.* (crude, ^{31}P-NMR in $CDCl_3$), 99:1 *d.r.* (after purification, ^{31}P-NMR in $CDCl_3$).

Specific rotation: $[\alpha]_D^{20} = -189.0$ (c = 1.38, CH_2Cl_2).

^{1}H NMR (600 MHz, $CDCl_3$) δ 7.98 (d, *J* = 9.0 Hz, 1H), 7.91 (d, *J* = 8.2 Hz, 1H), 7.81 (t, *J* = 8.1 Hz, 2H), 7.75 (d, *J* = 8.9 Hz, 1H), 7.43 (t, *J* = 7.4 Hz, 1H), 7.33 – 7.27 (m, 3H), 7.25 – 7.20 (m, 2H), 7.19 – 7.10 (m, 4H), 7.07 (d, *J* = 8.5 Hz, 1H), 7.02 (t, *J* = 7.6 Hz, 2H), 6.97 (d, *J* = 7.5 Hz, 2H), 6.79 (d, *J* = 8.2 Hz, 2H), 6.68 (d, *J* = 8.2 Hz, 2H), 6.52 (d, *J* = 7.7 Hz, 2H), 4.22 – 4.13 (m, 1H), 4.13 – 4.06 (m, 1H), 3.72 (s, 3H), 2.10 – 1.96 (m, 1H), 1.82 – 1.72 (m, 1H), 1.58 (s, 1H), 0.81 (d, *J* = 6.7 Hz, 3H), 0.76 (d, *J* = 6.8 Hz, 3H).

^{13}C NMR (151 MHz, $CDCl_3$) δ 158.1, 152.2, 148.7 (d, *J* = 9.3 Hz), 144.6 (d, *J* = 7.6 Hz), 137.0 (d, *J* = 4.2 Hz), 136.5, 136.4, 133.8, 131.34, 131.32, 130.7, 130.3, 129.5, 128.8, 128.5, 128.3, 128.2, 127.9, 127.6, 127.4, 127.1, 126.7, 126.4, 125.6, 124.3 (d, *J* = 187.1 Hz), 122.0 (d, *J* = 5.6 Hz), 121.0 (d, *J* = 2.3 Hz), 119.7, 116.9, 114.3, 113.8, 113.7, 55.3, 48.4 (d, *J* = 3.3 Hz), 44.0, 32.4 (d, *J* = 129.7 Hz), 23.0 (d, *J* = 4.1 Hz), 21.9.

^{31}P NMR (243 MHz, $CDCl_3$) δ 31.49.

HRMS (ESI+, m/z) calculated for $C_{44}H_{40}NO_4PH$ $[M+H]^+$: 678.2768 found 678.2762.

(*R*)-2'-hydroxy-[1,1'-binaphthalen]-2-yl (*R*)-*N*, *N*-diisopropyl-*P*-((*R*)-2-phenylpropyl)phosphonamidite (6)

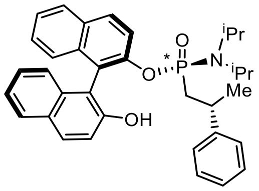

6

Following General Procedure C on 0.1 mmol scale using *R*-**3a** and MeMgBr (3 M in Et_2O), or MeLi (1.6 M in hexane) or $CuLiMe_2$. Purification by flash chromatography (3:1 hexanes/EtOAc) afforded 45.2 mg (76%) of the title compound as a white solid.

Diastereomeric purity: 85:4:0:11 *d.r.* (crude, ^{31}P-NMR in $CDCl_3$), 95:5 *d.r.* (after purification, ^{31}P-NMR in $CDCl_3$).

Specific rotation: $[\alpha]_D^{20} = 63.7$ (c = 1.19, CH_2Cl_2).

^{1}H NMR (400 MHz, $CDCl_3$) δ 7.99 (d, *J* = 8.9 Hz, 1H), 7.94 (dd, *J* = 8.5, 5.3 Hz, 2H), 7.86 (d, *J* = 8.0 Hz, 1H), 7.74 (dd, *J* = 9.0, 1.1 Hz, 1H), 7.49 – 7.41 (m, 2H), 7.34 – 7.27 (m, 3H), 7.25 – 7.18 (m, 3H), 7.18 – 7.10 (m, 1H), 7.05 (dd, *J* = 8.4, 1.1 Hz, 1H), 6.99 – 6.92 (m, 2H), 3.30 – 3.06 (m, 2H), 2.92 – 2.64 (m, 1H), 1.78 – 1.65 (m, 1H), 1.31 – 1.22 (m, 1H), 1.16 (d, *J* = 6.7 Hz, 6H), 1.10 (d, *J* = 6.8 Hz, 6H), 1.06 (d, *J* = 6.9 Hz, 3H).

^{13}C NMR (151 MHz, $CDCl_3$) δ 152.5, 147.9 (d, *J* = 9.9 Hz), 147.8 (d, *J* = 2.9 Hz), 133.90, 133.85, 131.3, 130.1, 130.0, 129.4, 128.5, 128.1, 127.9, 127.1, 126.43, 126.38, 126.1, 125.6, 125.5, 124.8, 123.6, 123.14 (d, *J* = 5.2 Hz), 121.4 (d, *J* = 2.6 Hz), 120.5, 117.9, 46.1 (d, *J* = 4.6 Hz), 35.6 (d, *J* = 127.2 Hz), 33.9 (d, *J* = 2.1 Hz), 22.9 (d, *J* = 3.2 Hz), 22.8 (d, *J* = 75.2 Hz).

^{31}P-NMR (162 MHz, $CDCl_3$) δ 35.78.

HRMS (ESI+, m/z) calculated for $C_{35}H_{39}NO_3P$ $[M+H]^+$: 552.2662, found 552.2656.

(*R*)- 2'-hydroxy-[1,1'-binaphthalen]-2-yl (*R*)-*N*, *N*-diisopropyl-*P*-((*R*)-2-phenylbutyl)phosphonamidate (7)

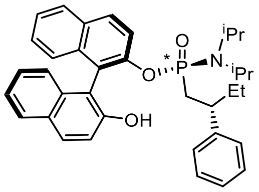

7

Following General Procedure C on 0.1 mmol scale using *R*-**3a** and EtMgBr (3 M in Et_2O), or EtLi (0.5 M in benzene/cyclohexane) or $CuLiEt_2$. Purification by flash chromatography (3:1 hexanes/EtOAc) afforded 48.0 mg (85%) of the title compound as a white solid.

Diastereomeric purity: 82:9:0:9 *d.r.* (crude, ^{31}P-NMR in $CDCl_3$), 93:7 *d.r.* (after purification, ^{31}P-NMR in $CDCl_3$).

Specific rotation: $[\alpha]_D^{20} = 56.3$ (c = 0.98, CH_2Cl_2).

^{1}H NMR (400 MHz, $CDCl_3$) δ 7.98 (t, 2H), 7.93 (d, *J* = 7.9 Hz, 1H), 7.87 (d, *J* = 8.1 Hz, 1H), 7.75 (dd, *J* = 8.9, 0.9 Hz, 1H), 7.48 – 7.39 (m, 2H), 7.34 – 7.26 (m, 3H), 7.25 – 7.18 (m, 3H), 7.16 – 7.10 (m, 1H), 7.05 (dd, *J* = 8.4, 1.1 Hz, 1H), 6.94 – 6.89 (m, 2H), 3.28 – 2.96 (m, 2H), 2.79 – 2.35 (m, 1H), 2.03 – 1.81 (m, 1H), 1.64 (td, *J* = 16.0, 3.7 Hz, 1H), 1.30 – 1.19 (m, 2H), 1.12 (d, *J* = 6.7 Hz, 6H), 1.04 (d, *J* = 6.8 Hz, 6H), 0.52 (t, *J* = 7.3 Hz, 3H).

^{13}C NMR (151 MHz, $CDCl_3$) δ 152.6, 148.2 (d, *J* = 9.6 Hz), 145.5 (d, *J* = 14.8 Hz), 133.9, 131.3, 130.2, 130.0, 129.5, 128.4, 128.2, 128.0, 127.5, 127.32, 127.29, 126.6, 126.3, 125.7, 125.5, 124.9, 123.7, 122.9 (d, *J* = 5.4 Hz), 121.5 (d, *J* = 2.6 Hz), 120.3, 117.7, 46.2 (d, *J* = 4.5 Hz), 41.3 (d, *J* = 1.6 Hz), 34.3 (d, *J* = 127.6 Hz), 29.9 (d, *J* = 4.6 Hz), 22.8 (d, *J* = 87.9 Hz), 11.8.

^{31}P-NMR (162 MHz, $CDCl_3$) δ 35.71.

HRMS (ESI+, m/z) calculated for $C_{36}H_{40}NO_3PNa$ $[M+Na]^+$: 588.2639, found 588.2638.

(*R*)-2'-hydroxy-[1,1'-binaphthalen]-2-yl (*R*)-*N*, *N*-diisopropyl-*P*-((*R*)-3-methyl-2-phenylbutyl)phosphonamidate (8)

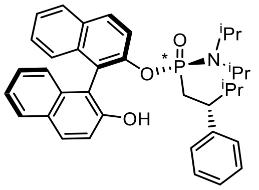

8

Following General Procedure C on 0.1 mmol scale using *R*-**3a** and ^{i}PrMgBr (3 M in 2-MeTHF). Purification by flash chromatography (3:1 hexanes/EtOAc) afforded 35.0 mg (60%) of the title compound as a white solid.

Diastereomeric purity: 89:8:0:3 *d.r.* (crude, ^{31}P-NMR in $CDCl_3$), 91:9 *d.r.* (after purification, ^{31}P-NMR in $CDCl_3$).

Specific rotation: $[\alpha]_D^{20} = 66.5$ (c = 0.86, CH_2Cl_2).

^{1}H NMR (400 MHz, $CDCl_3$) δ 7.98 (d, *J* = 8.9 Hz, 1H), 7.93 (dd, *J* = 8.5, 2.8 Hz, 2H), 7.87 (d, *J* = 8.5 Hz, 1H), 7.82 (d, 1H), 7.49 – 7.41 (m, 2H), 7.35 – 7.27 (m, 2H), 7.26 – 7.22 (m, 2H), 7.22 – 7.16 (m, 2H), 7.14 (d, *J* = 7.2 Hz, 1H), 7.07 (d, *J* = 8.4 Hz, 1H), 6.94 (d, *J* = 6.8 Hz, 2H), 3.11 – 2.94 (m, 2H), 2.66 (dq, *J* = 17.7, 6.0 Hz, 1H), 1.85 (td, *J* = 7.0, 5.4 Hz, 1H), 1.72 (dd, *J* = 16.1, 6.0 Hz, 1H), 1.67 – 1.58 (m, 1H), 1.05 (d, *J* = 6.7 Hz, 6H), 0.92 (d, *J* = 6.7 Hz, 6H), 0.61 (d, *J* = 6.8 Hz, 3H), 0.49 (d, *J* = 6.8 Hz, 3H).

^{13}C NMR (101 MHz, $CDCl_3$) δ 152.3, 148.2 (d, *J* = 9.3 Hz), 142.7 (d, *J* = 9.6 Hz), 133.9 (d, *J* = 2.2 Hz), 131.3, 130.3, 130.1, 129.5, 129.0, 128.3, 128.1, 127.8, 127.3, 126.6, 126.2, 125.7, 125.5, 125.0, 123.7, 122.4 (d, *J* = 5.8 Hz), 121.5 (d, *J* = 2.6 Hz), 119.9, 118.0, 117.1, 46.2 (d, *J* = 4.6 Hz), 45.1 (d, *J* = 2.1 Hz), 32.9 (d, *J* = 8.3 Hz), 31.1 (d, *J* = 129.4 Hz), 23.0 (d, *J* = 1.4 Hz), 22.3 (d, *J* = 1.7 Hz), 21.1, 17.9.

^{31}P-NMR (162 MHz, $CDCl_3$) δ 35.95.

HRMS (ESI+, m/z) calculated for $C_{37}H_{42}NO_3PNa$ $[M+Na]^+$: 602.2795, found 602.2791.

(*R*)-2'-hydroxy-[1,1'-binaphthalen]-2-yl (*R*)-*N*, *N*-diisopropyl-*P*-((*R*)-2-phenylpent-4-en-1-yl)phosphonamidate (9)

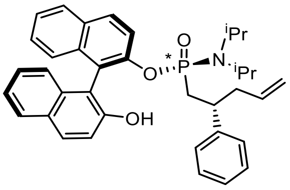

9

Following General Procedure C on 0.1 mmol scale using *R*-**3a** and allylmagnesium bromide (1 M in Et_2O). Purification by flash chromatography (3:1 hexanes/EtOAc) afforded 45.4 mg (78%) of the title compound as a white solid.

Diastereomeric purity: 79:4:0:17 *d.r.* (crude, ^{31}P-NMR in $CDCl_3$), 82:18 *d.r.* (after purification, ^{31}P-NMR in $CDCl_3$).

Specific rotation: $[\alpha]_D^{20} = 59.2$ (c = 0.96, CH_2Cl_2).

^{1}H NMR (400 MHz, $CDCl_3$) δ 7.98 (t, 2H), 7.95 – 7.90 (m, 2H), 7.87 (d, *J* = 7.9 Hz, 1H), 7.78 (dd, *J* = 8.9, 0.8 Hz, 1H), 7.47 – 7.40 (m, 2H), 7.34 – 7.29 (m, 2H), 7.25 – 7.18 (m, 3H), 7.18 – 7.12 (m, 1H), 7.11 – 7.05 (m, 1H), 6.94 (d, *J* = 7.0 Hz, 2H), 6.75 – 6.60 (m, 1H), 5.36 – 5.20 (m, 1H), 4.84 – 4.71 (m, 2H), 3.18 – 3.00 (m, 2H), 2.87 – 2.71 (m, 1H), 2.57 (ddd, *J* = 12.8, 6.7, 4.5 Hz, 1H), 2.13 – 1.98 (m, 1H), 1.64 (td, *J* = 15.9, 4.1 Hz, 1H), 1.36 (ddd, *J* = 17.7, 15.6, 9.1 Hz, 1H), 1.10 (d, *J* = 6.7 Hz, 6H), 1.04 (d, *J* = 6.8 Hz, 6H).

^{13}C NMR (101 MHz, $CDCl_3$) δ 152.4, 148.1 (d, *J* = 9.3 Hz), 145.0 (d, *J* = 14.0 Hz), 136.2, 133.9, 131.3, 130.3, 129.5, 128.4, 128.3, 128.1, 127.5, 127.4, 126.6, 126.4, 125.7, 125.6, 124.9, 123.7, 122.6 (d, *J* = 5.5 Hz), 121.5 (d, *J* = 2.6 Hz), 120.1, 117.5, 116.5, 46.2 (d, *J* = 4.6 Hz), 41.1 (d, *J* = 5.3 Hz), 39.6 (d, *J* = 1.8 Hz), 33.4 (d, *J* = 128.0 Hz), 23.0 (d, *J* = 1.4 Hz), 22.5 (d, *J* = 1.8 Hz).

^{31}P-NMR (162 MHz, $CDCl_3$) δ 35.03.

HRMS (ESI+, m/z) calculated for $C_{37}H_{41}NO_3P$ $[M+H]^+$: 578.2819, found 578.2810.

(*R*)-2'-hydroxy-[1,1'-binaphthalen]-2-yl (*R*)-*P*-((*R*)-2,3-diphenylpropyl)-*N*, *N*-diisopropylphosphonamidate (10)

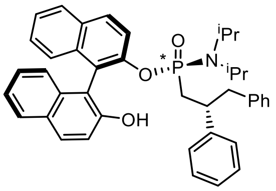

10

Following General Procedure C on 0.1 mmol scale using *R*-**3a** and benzylmagnesium bromide (0,9 M in THF). Purification by flash chromatography (3:1 hexanes/EtOAc) afforded 51.1 mg (81%) of the title compound as a white solid.

Diastereomeric purity: 85:6:0:9 *d.r.* (crude, ^{31}P-NMR in $CDCl_3$), 95:5 *d.r.* (after purification, ^{31}P-NMR in $CDCl_3$).

Specific rotation: $[\alpha]_D^{20} = 49.3$ (c = 0.92, CH_2Cl_2).

^{1}H NMR (400 MHz, $CDCl_3$) δ 8.01 – 7.93 (m, 3H), 7.92 – 7.85 (m, 1H), 7.82 (dd, *J* = 9.0, 1.1 Hz, 1H), 7.47 (ddd, *J* = 8.2, 6.5, 1.6 Hz, 1H), 7.41 – 7.34 (m, 3H), 7.35 – 7.28 (m, 3H), 7.25 – 7.21 (m, 1H), 7.11 (dd, *J* = 10.0, 7.4 Hz, 4H), 7.03 – 6.95 (m, 2H), 6.80 (d, *J* = 6.5 Hz, 2H), 6.75 – 6.70 (m, 1H), 3.21 (dd, *J* = 13.4, 4.8 Hz, 1H), 3.13 – 3.04 (m, 1H), 3.04 – 2.93 (m, 2H), 2.55 (dd, *J* = 13.4, 9.7 Hz, 1H), 1.70 (td, *J* = 15.9, 4.2 Hz, 1H), 1.44 (ddd, *J* = 17.7, 15.6, 8.9 Hz, 1H), 1.04 (d, *J* = 6.7 Hz, 6H), 1.01 (d, *J* = 6.8 Hz, 6H).

^{13}C NMR (101 MHz, $CDCl_3$) δ 152.9, 152.2, 148.3 (d, *J* = 8.9 Hz), 144.6 (d, *J* = 13.3 Hz), 139.8, 133.9, 133.8, 131.3, 130.5, 130.1, 129.5, 129.4, 128.7, 128.21, 128.20 (d, *J* = 28.1 Hz), 127.8 (d, *J* = 6.4 Hz), 127.4, 127.1, 126.5 (d, *J* = 30.6 Hz), 125.8, 125.6, 124.4 (d, *J* = 123.8 Hz), 124.3 (d, *J* = 45.2 Hz), 122.2 (d, *J* = 5.6 Hz), 121.5 (d, *J* = 2.5 Hz), 119.7, 118.0, 117.1, 65.4, 46.2 (d, *J* = 4.4 Hz), 43.8 (d, *J* = 5.5 Hz), 41.6 (d, *J* = 1.5 Hz), 32.6 (d, *J* = 127.8 Hz), 22.9 (d, *J* = 1.6 Hz), 22.6 (d, *J* = 2.0 Hz).

^{31}P-NMR (162 MHz, $CDCl_3$) δ 34.68.

HRMS (ESI+, m/z) calculated for $C_{41}H_{43}NO_3P$ $[M+H]^+$: 628.2975 found 628.2975.

(*R*)-2'-hydroxy-[1,1'-binaphthalen]-2-yl (*R*)-*N*, *N*-diisopropyl-*P*-((*R*)-2-(2-methoxyphenyl)-2-phenylethyl)phosphonamidate (11)

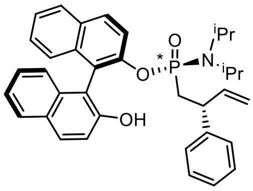

11

Following General Procedure C on 0.1 mmol scale using *R*-**3a** and vinylmagnesium bromide (1 M in THF). Purification by flash chromatography (3:1 hexanes/EtOAc) afforded 35.1 mg (62%) of the title compound as a white solid.

Diastereomeric purity: 79:6:1:13 *d.r.* (crude, ^{31}P-NMR in $CDCl_3$), 95:5 *d.r.* (after purification, ^{31}P-NMR in $CDCl_3$).

Specific rotation: $[\alpha]_D^{20} = 6.2$ (c = 0.42, CH_2Cl_2).

^{1}H NMR (400 MHz, $CDCl_3$) δ 7.99 (d, *J* = 9.0 Hz, 1H), 7.93 (dd, *J* = 8.4, 5.3 Hz, 2H), 7.84 (t, *J* = 9.9 Hz, 2H), 7.48 – 7.42 (m, 1H), 7.40 (d, *J* = 8.9 Hz, 1H), 7.34 – 7.27 (m, 3H), 7.23 (d, *J* = 7.5 Hz, 3H), 7.19 – 7.12 (m, 1H), 7.07 (d, *J* = 8.4 Hz, 1H), 6.99 (d, *J* = 7.1 Hz, 2H), 6.55 (s, 1H), 5.78 (ddd, *J* = 16.9, 10.3, 6.6 Hz, 1H), 4.85 (d, *J* = 10.2 Hz, 1H), 4.63 (d, *J* = 17.1 Hz, 1H), 3.57 – 3.34 (m, 1H), 3.11 (dp, *J* = 18.1, 6.7 Hz, 2H), 1.77 (td, *J* = 15.6, 4.4 Hz, 1H), 1.56 (ddd, *J* = 17.4, 15.5, 9.3 Hz, 1H), 1.09 (d, *J* = 6.7 Hz, 6H), 1.04 (d, *J* = 6.7 Hz, 6H).

^{13}C NMR (101 MHz, $CDCl_3$) δ 152.4, 148.1 (d, *J* = 9.4 Hz), 144.0 (d, *J* = 14.0 Hz), 141.3 (d, *J* = 5.8 Hz), 133.94, 133.90, 131.3, 130.3, 129.5, 128.6, 128.3, 128.1, 127.7, 127.4, 126.6, 125.7, 125.6, 125.0, 123.7, 122.4 (d, *J* = 5.5 Hz), 121.4 (d, *J* = 2.6 Hz), 120.0, 117.3, 114.6, 46.3 (d, *J* = 4.6 Hz), 43.0 (d, *J* = 2.2 Hz), 33.5 (d, *J* = 128.4 Hz), 23.0 (d, *J* = 1.5 Hz), 22.6 (d, *J* = 1.8 Hz).

^{31}P-NMR (162 MHz, $CDCl_3$) δ 33.94.

HRMS (ESI+, m/z) calculated for $C_{36}H_{38}NO_3PNa$ $[M+Na]^+$: 586.2482, found 586.2478.

(*R*)-2'-hydroxy-[1,1'-binaphthalen]-2-yl (*R*)-*N*, *N*-diisopropyl-*P*-((*S*)-2-(2-methoxyphenyl)-2-phenylethyl)phosphonamidite (12)

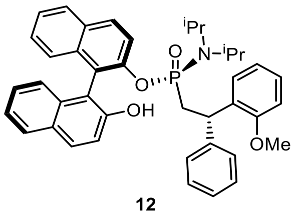

12

Following General Procedure C on 0.1 mmol scale using *R*-**3a** and 2-methoxyphenylmagnesium bromide. Purification by flash chromatography (3:1 hexanes/EtOAc) afforded 56.8 mg (88%) of the title compound as a white solid.

Diastereomeric purity: 5:86:0:9 *d.r.* (crude, ^{31}P-NMR in $CDCl_3$), 95:5 *d.r.* (after purification, ^{31}P-NMR in $CDCl_3$).

Specific rotation: $[\alpha]_D^{20} = 114.8$ (c = 1.98, CH_2Cl_2).

^{1}H NMR (400 MHz, $CDCl_3$) δ 7.93 (dd, *J* = 13.2, 8.5 Hz, 2H), 7.86 – 7.79 (m, 3H), 7.43 (ddd, *J* = 8.1, 6.6, 1.4 Hz, 1H), 7.34 – 7.27 (m, 3H), 7.26 – 7.21 (m, 2H), 7.21 – 7.16 (m, 1H), 7.16 – 7.08 (m, 5H), 7.05 (d, *J* = 8.5 Hz, 1H), 6.95 (dd, *J* = 7.6, 1.6 Hz, 1H), 6.86 (td, *J* = 7.5, 1.0 Hz, 1H), 6.70 (dd, *J* = 8.2, 1.2 Hz, 1H), 6.36 (s, 1H), 4.72 – 4.37 (m, 1H), 3.53 (s, 3H), 3.10 – 2.91 (m, 2H), 2.36 – 2.14 (m, 1H), 2.12 – 1.93 (m, 1H), 0.95 (d, *J* = 1.4 Hz, 6H), 0.94 (d, *J* = 1.5 Hz, 6H).

^{13}C NMR (101 MHz, $CDCl_3$) 156.2, 152.0, 148.4 (d, *J* = 8.9 Hz), 144.0 (d, *J* = 9.4 Hz), 133.8, 133.7, 131.1, 130.12, 130.12, 129.9, 129.3, 128.2, 128.1, 128.00, 127.97, 127.4, 127.1, 126.5, 126.0, 125.5, 125.3, 124.9, 123.5, 121.5 (d, *J* = 2.5 Hz), 120.5, 119.4, 116.7, 110.7, 55.2, 46.1 (d, *J* = 4.3 Hz), 38.5, 32.5 (d, *J* = 127.8 Hz), 22.7, 22.2 (d, *J* = 2.2 Hz).

^{31}P-NMR (162 MHz, $CDCl_3$) δ 33.46.

HRMS (ESI+, m/z) calculated for $C_{41}H_{43}NO_4P$ $[M+H]^+$: 644.2924 found 644.2913.

(*R*)-2'-hydroxy-[1,1'-binaphthalen]-2-yl (*R*)-*N*, *N*-diisopropyl-*P*-((*S*)-2-phenyl-2-(p-tolyl)ethyl)phosphonamidate (13)

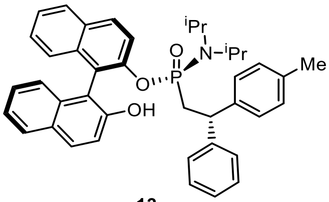

13

Following General Procedure C on 0.1 mmol scale using *R*-**3a** and *p*-tolylmagnesium bromide (1 M in THF). Purification by flash chromatography (3:1 hexanes/EtOAc) afforded 51.0 mg (81%) of the title compound as a white solid.

Diastereomeric purity: 94:5:0:1 *d.r.* (crude, ^{31}P-NMR in $CDCl_3$), 99:1 *d.r.* (after purification, ^{31}P-NMR in $CDCl_3$).

Specific rotation: $[\alpha]_D^{20} = 55.9$ (c = 0.54, CH_2Cl_2).

^{1}H NMR (400 MHz, $CDCl_3$) δ 7.95 (t, *J* = 4.5 Hz, 1H), 7.93 – 7.88 (m, 1H), 7.88 – 7.81 (m, 3H), 7.44 (ddd, *J* = 8.1, 6.6, 1.4 Hz, 1H), 7.38 – 7.28 (m, 4H), 7.26 – 7.18 (m, 3H), 7.11 (dd, *J* = 17.6, 8.0 Hz, 4H), 6.96 (d, *J* = 7.9 Hz, 2H), 6.78 (d, *J* = 8.0 Hz, 2H), 4.08 – 3.93 (m, 1H), 3.18 – 2.91 (m, 2H), 2.35 – 2.26 (m, 1H), 2.25 (s, 3H), 2.08 – 1.93 (m, 1H), 1.02 (d, *J* = 6.7 Hz, 6H), 0.97 (d, *J* = 6.7 Hz, 6H).

^{13}C NMR (101 MHz, $CDCl_3$) δ 152.8, 152.1, 148.1 (d, *J* = 9.0 Hz), 144.5 (d, *J* = 8.2 Hz), 141.9 (d, *J* = 10.5 Hz), 135.6, 133.8 (d, *J* = 13.6 Hz), 131.2, 131.1, 130.2, 130.1, 129.4, 129.0, 128.4, 128.1 (d, *J* = 9.9 Hz), 127.8, 127.4, 127.2, 126.4 (d, *J* = 33.4 Hz), 125.5 (d, *J* = 13.1 Hz), 124.22 (d, *J* = 200.5 Hz), 124.15 (d, *J* = 90.1 Hz), 121.8 (d, *J* = 5.7 Hz), 121.3 (d, *J* = 2.7 Hz), 119.5, 117.9, 116.6, 111.5, 46.2 (d, *J* = 4.6 Hz), 44.5 (d, *J* = 2.4 Hz), 34.3 (d, *J* = 128.2 Hz), 22.8, 22.4, 21.0.

^{31}P-NMR (162 MHz, $CDCl_3$) δ 33.17.

HRMS (ESI+, m/z) calculated for $C_{41}H_{43}NO_3P$ $[M+H]^+$: 628.2975 found 628.2969.

(*R*)-2'-hydroxy-[1,1'-binaphthalen]-2-yl (*R*)-*N*, *N*-diisopropyl-P-((*R*)-2-phenyl-2-(p-tolyl)ethyl)phosphonamidite (13')

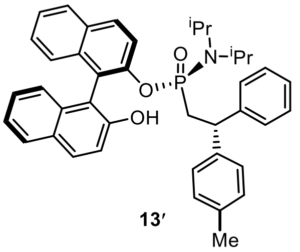

13′

Following General Procedure C on 0.1 mmol scale using *R*-**3b** and phenylmagnesium bromide (1 M in THF). Purification by flash chromatography (3:1 hexanes/EtOAc) afforded 46.6 mg (74%) of the title compound as a white solid.

Diastereomeric purity: 95:0:5:0 *d.r.* (crude, ^{31}P-NMR in $CDCl_3$), 99:1 *d.r.* (after purification, ^{31}P-NMR in $CDCl_3$).

Specific rotation: $[\alpha]_D^{20} = 176.6$ (c = 0.77, CH_2Cl_2).

^{1}H NMR (600 MHz, $CDCl_3$) δ 7.97 (d, *J* = 9.0 Hz, 1H), 7.94 (d, *J* = 8.2 Hz, 1H), 7.90 – 7.82 (m, 3H), 7.46 (t, *J* = 7.5 Hz, 1H), 7.40 – 7.30 (m, 3H), 7.30 – 7.21 (m, 3H), 7.21 – 7.13 (m, 2H), 7.11 (t, *J* = 8.2 Hz, 2H), 7.05 (t, *J* = 5.5 Hz, 3H), 6.89 (d, *J* = 7.5 Hz, 2H), 4.04 (dd, *J* = 15.0, 7.4 Hz, 1H), 3.41 – 2.72 (m, 2H), 2.38 – 2.30 (m, 1H), 2.29 (s, 3H), 2.00 (ddd, *J* = 18.1, 15.6, 6.0 Hz, 1H), 1.07 (d, *J* = 6.7 Hz, 6H), 1.01 (d, *J* = 6.9 Hz, 6H).

^{13}C NMR (151 MHz, $CDCl_3$) δ 152.3, 148.1 (d, *J* = 8.9 Hz), 145.3 (d, *J* = 10.4 Hz), 141.3 (d, *J* = 8.1 Hz), 135.9, 133.9 (d, *J* = 6.6 Hz), 131.2, 130.3, 130.1, 129.4, 128.760 (d, *J* = 89.0 Hz), 128.762 (d, *J* = 123.4 Hz), 128.23, 128.19, 127.8, 127.7 (d, *J* = 60.5 Hz), 127.6, 127.3, 126.6, 126.2, 125.6, 125.5, 125.0, 123.6, 122.0 (d, *J* = 5.9 Hz), 121.3 (d, *J* = 2.7 Hz), 120.0, 116.8, 46.3, 44.6 (d, *J* = 2.5 Hz), 34.5 (d, *J* = 128.4 Hz), 23.0, 22.4 (d, *J* = 1.8 Hz), 21.1.

^{31}P-NMR (243 MHz, $CDCl_3$) δ 33.22.

(*R*)-2'-hydroxy-[1,1'-binaphthalen]-2-yl (*R*)-*P*-((*R*)-2-(4-chlorophenyl)-2-phenylethyl)-*N*, *N*-diisopropylphosphonamidate (14)

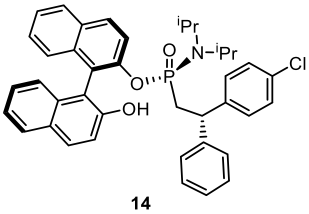

14

Following General Procedure C on 0.1 mmol scale using *R*-**3a** and 4-chlorophenylmagnesium bromide (1 M in Et_2O). Purification by flash chromatography (3:1 hexanes/EtOAc) afforded 45.0 mg (70%) of the title compound as a white solid.

Diastereomeric purity: 89:5:0:6 *d.r.* (crude, ^{31}P-NMR in $CDCl_3$), 95:5 *d.r.* (after purification, ^{31}P-NMR in $CDCl_3$).

Specific rotation: $[\alpha]_D^{20} = 75.9$ (c = 1.53, CH_2Cl_2).

^{1}H NMR (400 MHz, $CDCl_3$) δ 7.93 (dd, *J* = 12.3, 8.6 Hz, 2H), 7.86 – 7.80 (m, 2H), 7.77 (dd, *J* = 9.0, 1.1 Hz, 1H), 7.44 (ddd, *J* = 8.1, 6.7, 1.3 Hz, 1H), 7.40 – 7.27 (m, 4H), 7.25 – 7.19 (m, 3H), 7.14 (d, *J* = 7.3 Hz, 1H), 7.12 – 7.03 (m, 5H), 6.78 (d, *J* = 8.5 Hz, 2H), 6.42 (s, 1H), 3.98 (dt, *J* = 15.4, 6.6 Hz, 1H), 3.20 – 2.93 (m, 2H), 2.23 (td, *J* = 15.4, 6.9 Hz, 1H), 1.92 (ddd, *J* = 18.0, 15.6, 6.5 Hz, 1H), 1.05 (d, *J* = 6.7 Hz, 6H), 0.97 (d, *J* = 6.7 Hz, 6H).

^{13}C NMR (101 MHz, $CDCl_3$) δ 152.9, 152.2, 147.9 (d, *J* = 9.2 Hz), 144.0 (d, *J* = 8.9 Hz), 143.3 (d, *J* = 9.6 Hz), 133.9, 133.8, 132.0, 131.4, 131.3, 130.4, 130.2, 129.4, 129.1, 128.6, 128.5, 128.2 (d, *J* = 9.2 Hz), 127.9, 127.4, 126.7, 125.6 (d, *J* = 4.4 Hz), 124.4 (d, *J* = 121.2 Hz), 124.3 (d, *J* = 38.3 Hz), 122.2 (d, *J* = 5.8 Hz), 121.3 (d, *J* = 2.6 Hz), 119.7, 118.0, 116.9, 46.3 (d, *J* = 4.5 Hz), 44.4 (d, *J* = 2.1 Hz), 34.9, 33.6, 23.0 (d, *J* = 1.5 Hz), 22.5 (d, *J* = 1.9 Hz).

^{31}P-NMR (162 MHz, $CDCl_3$) δ 32.83.

HRMS (ESI+, m/z) calculated for $C_{40}H_{39}ClNO_3PNa$ $[M+Na]^+$: 670.2248 found 670.2245.

(*R*)-2'-hydroxy-[1,1'-binaphthalen]-2-yl (*R*)-*P*-((*R*)-2-(4-fluorophenyl)-2-phenylethyl)-*N*, *N*-diisopropylphosphonamidate (15)

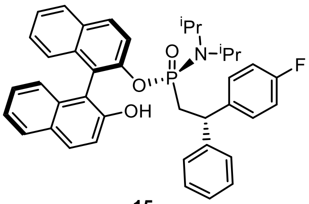

15

Following General Procedure C on 0.1 mmol scale using *R*-**3a** and 4-fluorophenylmagnesium bromide (1 M in THF). Purification by flash chromatography (3:1 hexanes/EtOAc) afforded 57.0 mg (90%) of the title compound as a white solid.

Diastereomeric purity: 88:8:0:4 *d.r.* (crude, ^{31}P-NMR in $CDCl_3$), 94:6 *d.r.* (after purification, ^{31}P-NMR in $CDCl_3$).

Specific rotation: $[\alpha]_D^{20} = 69.4$ (c = 1.26, CH_2Cl_2).

^{1}H NMR (400 MHz, $CDCl_3$) δ 7.93 (dd, *J* = 14.9, 8.5 Hz, 2H), 7.86 – 7.74 (m, 3H), 7.44 (ddd, *J* = 8.1, 6.7, 1.3 Hz, 1H), 7.36 – 7.28 (m, 3H), 7.26 – 7.20 (m, 4H), 7.17 – 7.11 (m, 1H), 7.07 (ddd, *J* = 8.5, 3.9, 1.4 Hz, 3H), 6.87 – 6.78 (m, 4H), 6.47 (s, 1H), 4.07 – 3.93 (m, 1H), 3.14 – 2.97 (m, 2H), 2.24 (td, *J* = 15.5, 6.9 Hz, 1H), 1.93 (ddd, *J* = 18.1, 15.6, 6.5 Hz, 1H), 1.05 (d, *J* = 6.7 Hz, 6H), 0.97 (d, *J* = 6.8 Hz, 6H).

^{13}C NMR (101 MHz, $CDCl_3$) δ 161.4 (d, *J* = 244.3 Hz), 152.3, 148.0 (d, *J* = 9.2 Hz), 144.4 (d, *J* = 9.0 Hz), 140.6 (d, *J* = 3.2 Hz), 140.5 (d, *J* = 3.3 Hz), 133.93, 133.85, 131.3, 130.3, 130.2, 129.4, 129.2, 129.1, 128.6, 128.3, 128.2, 127.8, 127.4, 126.6 (d, *J* = 6.9 Hz), 125.6, 125.0, 123.7, 122.3 (d, *J* = 5.8 Hz), 121.4 (d, *J* = 2.7 Hz), 119.8, 116.9, 115.1 (d, *J* = 21.2 Hz), 46.3 (d, *J* = 4.5 Hz), 44.2 (d, *J* = 2.2 Hz), 34.4 (d, *J* = 128.5 Hz), 22.9 (d, *J* = 1.5 Hz), 22.5 (d, *J* = 1.9 Hz).
^{31}P-NMR (162 MHz, $CDCl_3$) δ 33.05.
^{19}F NMR (376 MHz, $CDCl_3$) δ -117.17.
HRMS (ESI+, m/z) calculated for $C_{40}H_{40}FNO_3P$ $[M+H]^+$: 632.2724 found 632.2720.

(*R*)-2'-hydroxy-[1,1'-binaphthalen]-2-yl (*R*)-*N, N*-diisopropyl-*P*-((*R*)-2-phenylhexyl)phosphonamidate (16)

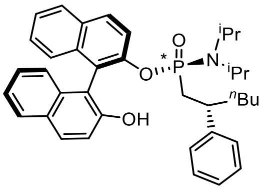

16

Following General Procedure C on 0.1 mmol scale using *R*-**3a** and ^{n}BuLi (1.6 M in hexanes). Purification by flash chromatography (3:1 hexanes/EtOAc) afforded 42.2 mg (71%) of the title compound as a white solid.
Diastereomeric purity: 73:25:0:2 *d.r.* (crude, ^{31}P-NMR in $CDCl_3$), 77:23 *d.r.* (after purification, ^{31}P-NMR in $CDCl_3$).
Specific rotation: $[\alpha]_D^{20} = 55.7$ (c = 0.83, CH_2Cl_2).
^{1}H NMR (400 MHz, $CDCl_3$) δ 7.98 (d, *J* = 9.0 Hz, 1H), 7.93 (dd, *J* = 8.6, 5.0 Hz, 2H), 7.87 (d, *J* = 8.6 Hz, 1H), 7.76 (d, *J* = 9.0 Hz, 1H), 7.48 – 7.38 (m, 2H), 7.30 (dd, *J* = 15.0, 8.1 Hz, 3H), 7.20 (d, *J* = 7.7 Hz, 2H), 7.18 – 7.08 (m, 2H), 7.09 – 7.02 (m, 1H), 6.94 (d, *J* = 7.2 Hz, 2H), 3.18 – 3.01 (m, 2H), 2.76 – 2.60 (m, 1H), 1.95 – 1.77 (m, 1H), 1.76 – 1.55 (m, 2H), 1.38 – 1.16 (m, 5H), 1.11 (dd, *J* = 8.0, 6.7 Hz, 6H), 1.03 (d, *J* = 6.7 Hz, 6H), 0.77 (t, *J* = 7.5 Hz, 3H).
^{13}C NMR (151 MHz, $CDCl_3$) δ 152.5, 148.2 (d, *J* = 9.3 Hz), 145.9 (d, *J* = 14.2 Hz), 133.9 (d, *J* = 3.1 Hz), 131.3, 130.2, 130.0, 129.5, 128.4, 128.3, 128.2, 128.0, 127.4, 127.3, 126.6, 126.3, 125.7, 125.5, 124.9, 123.7, 122.9 (d, *J* = 5.2 Hz), 121.5 (d, *J* = 2.5 Hz), 120.3, 117.6, 46.2 (d, *J* = 4.6 Hz), 39.7, 36.9 (d, *J* = 5.0 Hz), 34.5 (d, *J* = 127.5 Hz), 29.5, 22.8 (d, *J* = 56.6 Hz), 22.5, 14.2.
^{31}P-NMR (162 MHz, $CDCl_3$) δ 35.60.
HRMS (ESI+, m/z) calculated for $C_{38}H_{44}NO_3PNa$ $[M+Na]^+$: 616.2951, found 611.2946.

(*R*)-2'-hydroxy-[1,1'-binaphthalen]-2-yl (*R*)-*N, N*-diisopropyl-*P*-((*R*)-2-(4-methoxyphenyl)propyl)phosphonamidate (17)

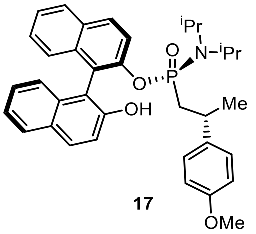

17

Following General Procedure C on 0.1 mmol scale using *R*-**3c** and MeMgBr (3 M in Et_2O). Purification by flash chromatography (3:1 hexanes/EtOAc) afforded 36.1 mg (62%) of the title compound as a white solid.
Diastereomeric purity: 77:5:0:18 *d.r.* (crude, ^{31}P-NMR in $CDCl_3$), 95:5 *d.r.* (after purification, ^{31}P-NMR in $CDCl_3$).
Specific rotation: $[\alpha]_D^{20} = 63.7$ (c = 1.19, CH_2Cl_2).
^{1}H NMR (400 MHz, $CDCl_3$) δ 7.99 (d, *J* = 9.0 Hz, 1H), 7.93 (dd, *J* = 8.5, 5.1 Hz, 2H), 7.88 – 7.84 (m, 1H), 7.74 (dd, *J* = 9.0, 1.1 Hz, 1H), 7.49 – 7.38 (m, 2H), 7.34 – 7.26 (m, 3H), 7.21 (d, 1H), 7.05 (d, *J* = 8.3 Hz, 1H), 6.97 (s, 1H), 6.88 (d, *J* = 8.7 Hz,

2H), 6.76 (d, J = 8.7 Hz, 2H), 3.76 (s, 3H), 3.31 – 3.06 (m, 2H), 2.89 – 2.56 (m, 1H), 1.65 (td, J = 16.1, 2.9 Hz, 2H), 1.16 (d, J = 6.6 Hz, 6H), 1.10 (d, J = 6.8 Hz, 6H), 1.03 (d, J = 6.9 Hz, 3H).

^{13}C NMR (101 MHz, $CDCl_3$) δ 158.0, 152.7, 148.0 (d, J = 9.6 Hz), 140.3 (d, J = 16.9 Hz), 134.03, 133.97, 131.4, 130.2, 130.1, 129.5, 128.2, 128.1, 127.4, 127.3, 126.6, 125.8, 125.6, 124.9, 123.7, 123.2 (d, J = 5.2 Hz), 121.5 (d, J = 2.5 Hz), 120.6, 118.0, 113.9, 55.4, 46.2 (d, J = 4.6 Hz), 36.0 (d, J = 126.7 Hz), 33.2 (d, J = 2.2 Hz), 23.3 (d, J = 3.4 Hz), 23.1 (d, J = 1.5 Hz), 22.6 (d, J = 1.6 Hz).

^{31}P-NMR (162 MHz, $CDCl_3$) δ 35.84.

HRMS (ESI+, m/z) calculated for $C_{36}H_{40}NO_4PNa$ $[M+Na]^+$: 604.2587 found 604.2593.

(*R*)-2'-hydroxy-[1,1'-binaphthalen]-2-yl (*R*)-*N, N*-diisopropyl-*P*-((*R*)-2-(4-methoxyphenyl)-2-(p-tolyl)ethyl)phosphonamidate (18)

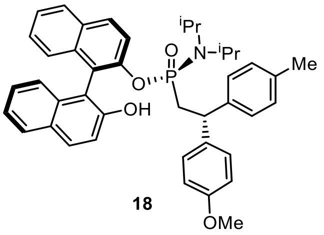

18

Following General Procedure C on 0.1 mmol scale using *R*-**3b** and 4-methylphenylmagnesium bromide (1 M in THF). Purification by flash chromatography (3:1 hexanes/EtOAc) afforded 48.2 mg (75%) of the title compound as a white solid.

Diastereomeric purity: 8:87:0:5 *d.r.* (crude, ^{31}P-NMR in $CDCl_3$), 94:6 *d.r.* (after purification, ^{31}P-NMR in $CDCl_3$).

Specific rotation: $[\alpha]_D^{20} = 37.6$ (c = 0.93, CH_2Cl_2).

^{1}H NMR (400 MHz, $CDCl_3$) δ 7.92 (dd, J = 15.2, 8.6 Hz, 2H), 7.83 (t, J = 9.1 Hz, 3H), 7.43 (t, J = 7.3 Hz, 1H), 7.37 – 7.28 (m, 3H), 7.22 (t, J = 8.0 Hz, 2H), 7.04 (dd, J = 15.3, 8.4 Hz, 3H), 6.94 (d, J = 7.8 Hz, 2H), 6.74 (d, J = 8.3 Hz, 2H), 6.69 (d, J = 7.8 Hz, 2H), 6.32 (s, 1H), 4.14 – 3.82 (m, 1H), 3.73 (d, J = 1.2 Hz, 3H), 3.23 – 2.89 (m, 2H), 2.24 (s, 3H), 2.22 – 2.14 (m, 1H), 2.00 – 1.86 (m, 1H), 1.03 (d, J = 6.7 Hz, 6H), 0.97 (d, J = 6.7 Hz, 6H).

^{13}C NMR (151 MHz, $CDCl_3$) δ 158.1, 152.3, 148.2 (d, J = 9.3 Hz), 142.6 (d, J = 11.0 Hz), 136.7 (d, J = 7.8 Hz), 135.6, 134.0, 133.9, 131.3, 130.3, 130.2, 129.5, 129.1, 128.9, 128.3, 128.2, 127.4, 127.3, 126.6, 125.6, 125.5, 125.0, 123.7, 121.9 (d, J = 5.6 Hz), 121.34 (d, J = 2.7 Hz), 119.7, 116.8, 113.8, 55.3, 46.3 (d, J = 4.5 Hz), 43.8 (d, J = 2.3 Hz), 34.8 (d, J = 127.9 Hz), 23.0, 22.5, 21.1.

^{31}P-NMR (162 MHz, $CDCl_3$) δ 33.16.

HRMS (ESI+, m/z) calculated for $C_{42}H_{44}NO_4PNa$ $[M+Na]^+$: 680.2900 found 680.2897.

(*R*)-2'-hydroxy-[1,1'-binaphthalen]-2-yl (*R*)-*N, N*-diisopropyl-*P*-((*R*)-2-(4-methoxyphenyl)-2-(thiophen-2-yl)ethyl)phosphonamidate (19)

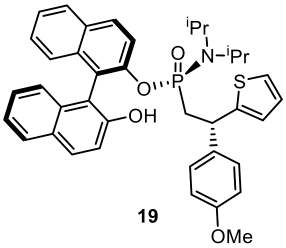

19

Following General Procedure C on 0.1 mmol scale using *R*-**3c** and 2-thienylmagnesium bromide (1 M in THF). Purification by flash chromatography (3:1 hexanes/EtOAc) afforded 45.7 mg (71%) of the title compound as a white solid.

Diastereomeric purity: 2:2:91:5 *d.r.* (crude, ^{31}P-NMR in $CDCl_3$), 97:3 *d.r.* (after purification, ^{31}P-NMR in $CDCl_3$).

Specific rotation: $[\alpha]_D^{20} = 83.5$ (c = 0.57, CH_2Cl_2).

^{1}H NMR (400 MHz, $CDCl_3$) δ 7.97 (d, *J* = 9.0 Hz, 1H), 7.92 (d, *J* = 8.1 Hz, 1H), 7.88 – 7.78 (m, 3H), 7.48 – 7.39 (m, 1H), 7.36 – 7.28 (m, 3H), 7.25 – 7.17 (m, 2H), 7.06 (t, *J* = 9.1 Hz, 3H), 7.01 (dd, *J* = 5.1, 1.2 Hz, 1H), 6.82 – 6.72 (m, 3H), 6.47 (dd, *J* = 3.5, 1.1 Hz, 1H), 6.23 (s, 1H), 4.30 – 4.12 (m, 1H), 3.75 (s, 3H), 3.03 (dd, *J* = 19.8, 6.8 Hz, 2H), 2.40 – 2.13 (m, 1H), 2.10 – 1.93 (m, 1H), 1.01 (d, *J* = 6.7 Hz, 6H), 0.94 (d, *J* = 6.7 Hz, 6H).

^{13}C NMR (101 MHz, $CDCl_3$) δ 158.5, 152.1, 150.0 (d, *J* = 13.2 Hz), 148.2 (d, *J* = 9.0 Hz), 136.5 (d, *J* = 7.1 Hz), 133.9, 131.3, 130.4, 130.3, 129.5, 128.9, 128.3, 128.2, 127.4, 126.60, 126.59, 125.62, 125.60, 124.9, 123.9, 123.8, 123.7, 121.8 (d, *J* = 6.2 Hz), 121.3 (d, *J* = 2.9 Hz), 119.5, 116.6, 113.9, 113.0, 55.3, 46.3 (d, *J* = 4.5 Hz), 40.0 (d, *J* = 2.3 Hz), 36.1 (d, *J* = 128.2 Hz), 22.9, 22.4 (d, *J* = 1.9 Hz).

^{31}P-NMR (162 MHz, $CDCl_3$) δ 31.85.

HRMS (ESI+, m/z) calculated for $C_{39}H_{40}NO_4PSH$ $[M+H]^+$: 650.2488 found 650.2489.

(*R*)-2'-hydroxy-[1,1'-binaphthalen]-2-yl (*R*)-*N*, *N*-diisopropyl-*P*-((*R*)-2-(o-tolyl)propyl)phosphonamidate (20)

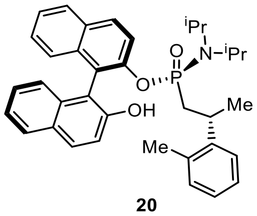

20

Following General Procedure C on 0.1 mmol scale using *R*-**3e** and methylmagnesium bromide (3 M in Et_2O). Purification by flash chromatography(3:1 hexanes/EtOAc) afforded 47.6 mg (68%) of the title compound as a white solid.

Diastereomeric purity: 82:6:0:12 *d.r.* (crude, ^{31}P-NMR in $CDCl_3$), 85:11:0:4 *d.r.* (after purification, ^{31}P-NMR in $CDCl_3$).

Specific rotation: $[\alpha]_D^{20} = 39.2$ (c = 1.49, CH_2Cl_2).

^{1}H NMR (400 MHz, $CDCl_3$) δ 8.02 – 7.94 (m, 2H), 7.97 – 7.88 (m, 2H), 7.78 (dd, *J* = 9.0, 1.1 Hz, 1H), 7.49 – 7.40 (m, 1H), 7.42 – 7.35 (m, 2H), 7.36 – 7.26 (m, 3H), 7.16 (dd, *J* = 8.4, 1.2 Hz, 1H), 7.10 – 7.03 (m, 2H), 6.82 – 6.76 (m, 1H), 3.21 – 3.13 (m, 2H), 3.13 – 3.06 (m, 1H), 2.27 (s, 3H), 1.70 – 1.56 (m, 2H), 1.15 (d, *J* = 6.6 Hz, 6H), 1.07 (d, *J* = 6.7 Hz, 6H), 1.01 (d, *J* = 6.8 Hz, 3H).

^{13}C NMR (151 MHz, $CDCl_3$) δ 152.9, 152.6, 148.1 (d, *J* = 9.4 Hz), 146.1 (d, *J* = 16.8 Hz), 142.5, 134.3 (d, *J* = 94.9 Hz), 133.6, 131.5, 131.0 (d, *J* = 126.6 Hz), 130.4 (d, *J* = 28.9 Hz), 129.9 (d, *J* = 79.7 Hz), 129.5 (d, *J* = 15.7 Hz), 128.2 (d, *J* = 26.2 Hz), 128.1 (d, *J* = 145.0 Hz), 127.0 (d, *J* = 117.1 Hz), 126.2 (d, *J* = 61.3 Hz), 125.7 (d, *J* = 23.2 Hz), 125.4, 124.3 (d, *J* = 184.2 Hz), 124.3 (d, *J* = 38.2 Hz), 122.9 (d, *J* = 5.4 Hz), 121.5 (d, *J* = 2.6 Hz), 120.4, 117.9, 114.7, 111.1, 46.2 (d, *J* = 4.6 Hz), 35.1 (d, *J* = 127.5 Hz), 29.2, 23.1, 22.6, 19.5.

^{31}P-NMR (162 MHz, $CDCl_3$) δ 35.95.

HRMS (ESI+, m/z) calculated for $C_{36}H_{40}NO_3PNa$ $[M+Na]^+$: 588.2638 found 588.2643.

(*R*)-2'-hydroxy-[1,1'-binaphthalen]-2-yl (*R*)-*N*, *N*-diisopropyl-*P*-((*R*)-2-(o-tolyl)-2-(*p*-tolyl)ethyl)phosphonamidate (21)

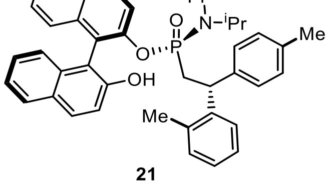

21

Following General Procedure C on 0.1 mmol scale using *R*-**3e** and *p*-tolylmagnesium bromide (1 M in THF). Purification by flash chromatography (3:1 hexanes/EtOAc) afforded 26.9 mg (43%) of the title compound as a white solid.

Diastereomeric purity: 8:86:0:6 *d.r.* (crude, ^{31}P-NMR in $CDCl_3$), 92:8 *d.r.* (after purification, ^{31}P-NMR in $CDCl_3$).

Specific rotation: $[\alpha]_D^{20} = 38.9$ (c = 1.11, CH_2Cl_2).

^{1}H NMR (400 MHz, $CDCl_3$) δ 7.98 (d, *J* = 8.9 Hz, 1H), 7.90 (dd, *J* = 7.5, 1.4 Hz, 2H), 7.86 – 7.81 (m, 2H), 7.41 – 7.35 (m, 2H), 7.34 – 7.28 (m, 3H), 7.24 – 7.21 (m, 1H), 7.18 – 7.14 (m, 2H), 7.08 – 7.02 (m, 3H), 6.94 (d, *J* = 7.9 Hz, 2H), 6.82 (d, *J* = 8.1 Hz, 2H), 5.93 (s, 1H), 3.07 – 2.84 (m, 2H), 2.30 (dd, *J* = 15.1, 7.2 Hz, 1H), 2.23 (d, *J* = 1.7 Hz, 6H), 1.95 – 1.80 (m, 1H), 0.95 (d, *J* = 6.7 Hz, 6H), 0.92 (d, *J* = 6.7 Hz, 6H).

^{13}C NMR (101 MHz, $CDCl_3$) δ 152.9, 151.9, 148.5 (d, *J* = 8.9 Hz), 142.1 (d, *J* = 8.1 Hz), 141.4 (d, *J* = 10.2 Hz), 136.2, 135.6, 133.8 (d, *J* = 7.8 Hz), 133.6, 131.5, 131.2, 130.7, 130.5, 129.9 (d, *J* = 58.2 Hz), 129.4, 128.8 (d, *J* = 45.0 Hz), 128.2 (d, *J* = 17.7 Hz), 127.7 (d, *J* = 28.9 Hz), 127.2 (d, *J* = 42.9 Hz), 126.5 (d, *J* = 21.8 Hz), 125.52, 125.51 (d, *J* = 105.1 Hz), 124.3 (d, *J* = 28.1 Hz), 123.7, 121.4 (d, *J* = 2.8 Hz), 121.1 (d, *J* = 6.1 Hz), 119.2, 117.9, 116.3, 111.1, 46.3 (d, *J* = 4.3 Hz), 40.2 (d, *J* = 1.9 Hz), 34.4 (d, *J* = 128.2 Hz), 22.8 (d, *J* = 1.5 Hz), 22.4 (d, *J* = 2.0 Hz), 21.1, 20.1.

^{31}P-NMR (162 MHz, $CDCl_3$) δ 33.15.

HRMS (ESI+, m/z) calculated for $C_{42}H_{44}NO_3PNa$ $[M+Na]^+$: 664.2951 found 664.2955.

(*R*)-2'-hydroxy-[1,1'-binaphthalen]-2-yl (*R*)-*N*, *N*-diisopropyl-*P*-((*R*)-2-(4-(trifluoromethyl)phenyl)propyl)phosphonamidate (22)

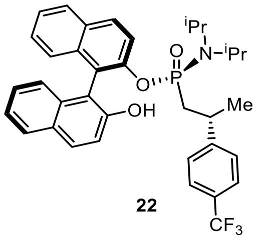

22

Following General Procedure C on 0.1 mmol scale using *R*-**3d** and methylmagnesium bromide (3 M in Et_2O). Purification by flash chromatography (3:1 hexanes/EtOAc) afforded 42.1 mg (68%) of the title compound as a white solid.

Diastereomeric purity: 67:13:2:18 *d.r.* (crude, ^{31}P-NMR in $CDCl_3$), 88:12 *d.r.* (after purification, ^{31}P-NMR in $CDCl_3$).

Specific rotation: $[\alpha]_D^{20} = 42.4$ (c = 1.43, CH_2Cl_2).

^{1}H NMR (400 MHz, $CDCl_3$) δ 7.98 (t, *J* = 9.1 Hz, 2H), 7.93 (t, *J* = 7.6 Hz, 2H), 7.89 – 7.85 (m, 1H), 7.75 (dd, *J* = 8.9, 1.1 Hz, 1H), 7.49 – 7.44 (m, 2H), 7.41 – 7.36 (m, 2H), 7.33 – 7.27 (m, 2H), 7.25 – 7.19 (m, 1H), 7.16 (d, *J* = 8.4 Hz, 1H), 7.11 – 7.03 (m, 2H), 6.80 (s, 1H), 3.31 – 3.04 (m, 2H), 2.93 – 2.69 (m, 1H), 1.63 (td, *J* = 15.9, 3.3 Hz, 2H), 1.16 (d, *J* = 6.7 Hz, 6H), 1.07 (d, *J* = 6.7 Hz, 6H).

^{13}C NMR (151 MHz, $CDCl_3$) δ 152.6 (d, *J* = 56.4 Hz), 147.7 (d, *J* = 9.6 Hz), 133.8 (d, *J* = 12.4 Hz), 133.5, 131.3, 130.2, 130.0, 129.4 (d, *J* = 12.2 Hz), 128.1 (d, *J* = 31.5 Hz), 127.5 (d, *J* = 280.8 Hz), 127.4 (d, *J* = 15.9 Hz), 126.8, 125.6 (d, *J* = 2.0 Hz), 125.5 (q, *J* = 3.7 Hz), 124.3, 124.2 (d, *J* = 169.3 Hz), 124.0, 122.8 (d, *J* = 5.4 Hz), 121.2 (d, *J* = 2.6 Hz), 120.1, 117.8, 117.5, 114.6, 111.2, 46.1 (d, *J* = 4.7 Hz), 35.4 (d, *J* = 128.1 Hz), 33.9 (d, *J* = 2.2 Hz), 23.0, 22.5.

^{31}P-NMR (162 MHz, $CDCl_3$) δ 34.58.

^{19}F NMR (376 MHz, Chloroform-*d*) δ -62.36.

HRMS (ESI+, m/z) calculated for $C_{36}H_{37}F_3NO_3PNa$ $[M+H]^+$: 642.2355 found 642.2354.

(*R*)-2'-hydroxy-[1,1'-binaphthalen]-2-yl (*R*)-*N*, *N*-diisopropyl-*P*-((*R*)-2-(*p*-tolyl)-2-(4-(trifluoromethyl)phenyl)ethyl)phosphonamidate (23)

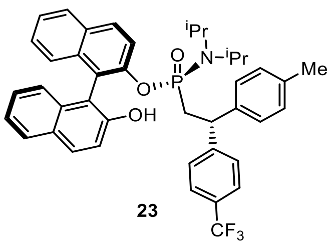

23

Following General Procedure C on 0.1 mmol scale using *R*-**3d** and *p*-tolylmagnesium bromide (1 M in THF). Purification by flash chromatography (3:1 hexanes/EtOAc) afforded 32.2 mg (47%) of the title compound as a white solid.

Diastereomeric purity: 8:86.5:0:5.5 *d.r.* (crude, ^{31}P-NMR in $CDCl_3$), 93:7 *d.r.* (after purification, ^{31}P-NMR in $CDCl_3$).

Specific rotation: $[\alpha]_D^{20} = 23.4$ (c = 1.05, CH_2Cl_2).

^{1}H NMR (400 MHz, $CDCl_3$) δ 8.00 – 7.93 (m, 2H), 7.93 – 7.88 (m, 1H), 7.87 – 7.82 (m, 2H), 7.46 (t, *J* = 7.2 Hz, 2H), 7.41 – 7.35 (m, 2H), 7.35 – 7.30 (m, 2H), 7.26 – 7.20 (m, 3H), 7.16 (dd, *J* = 8.3, 1.2 Hz, 1H), 7.07 (d, *J* = 8.4 Hz, 1H), 6.95 (d, *J* = 8.0 Hz, 2H), 6.64 (d, *J* = 8.1 Hz, 1H), 3.95 (tp, *J* = 14.0, 8.4, 5.2 Hz, 1H), 3.26 – 2.96 (m, 2H), 2.32 – 2.26 (m, 1H), 2.25 (s, 3H), 2.00 (td, *J* = 18.2, 15.5, 5.2 Hz, 1H), 1.05 (d, *J* = 6.6 Hz, 6H), 0.98 (d, *J* = 6.7 Hz, 6H).

^{13}C NMR (101 MHz, $CDCl_3$) δ 152.9, 152.2, 148.3 (d, *J* = 7.6 Hz), 148.0 (d, *J* = 9.2 Hz), 141.4 (d, *J* = 11.9 Hz), 136.2, 133.9 (d, *J* = 5.3 Hz), 133.6, 130.9 (d, *J* = 95.7 Hz), 130.4 (d, *J* = 174.5 Hz), 130.3, 129.4, 129.3, 128.5, 128.3 (d, *J* = 3.6 Hz), 127.9 (d, *J* = 96.9 Hz), 127.5 (d, *J* = 7.4 Hz), 125.6 (d, *J* = 6.2 Hz), 125.4 (q, *J* = 3.8 Hz), 125.2 (d, *J* = 294.1 Hz), 124.9, 124.3 (d, *J* = 37.4 Hz), 121.6 (d, *J* = 5.9 Hz), 121.1 (d, *J* = 2.9 Hz), 119.5, 118.0, 116.5, 111.3, 46.3 (d, *J* = 4.6 Hz), 44.6 (d, *J* = 2.9 Hz), 34.6 (d, *J* = 128.3 Hz), 23.0, 22.4 (d, *J* = 1.7 Hz), 21.1.

^{31}P-NMR (162 MHz, $CDCl_3$) δ 31.88.

^{19}F NMR (376 MHz, Chloroform-*d*) δ -62.37.

HRMS (ESI+, m/z) calculated for $C_{42}H_{41}F_3NO_3PNa$ $[M+H]^+$: 718.2668 found 718.2660.

(*R*)-2'-hydroxy-[1,1'-binaphthalen]-2-yl (*R*)-*N*, *N*-diisopropyl-*P*-((*R*)-2-(naphthalen-2-yl)-2-phenylethyl)phosphonamidate (24)

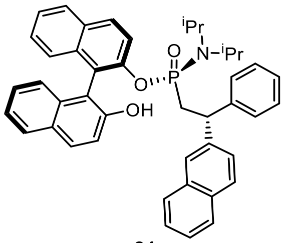

24

Following General Procedure C on 0.1 mmol scale using *R*-**3f** and phenylmagnesium bromide (1 M in THF). Purification by flash chromatography (3:1 hexanes/EtOAc) afforded 54.4 mg (82%) of the title compound as a white solid.

Diastereomeric purity: 6:89:0:5 *d.r.* (crude, ^{31}P-NMR in $CDCl_3$), 94:6 *d.r.* (after purification, ^{31}P-NMR in $CDCl_3$).

Specific rotation: $[\alpha]_D^{20} = 108.9$ (c = 1.28, CH_2Cl_2).

^{1}H NMR (400 MHz, $CDCl_3$) δ 7.96 (d, *J* = 9.0 Hz, 1H), 7.92 (d, *J* = 8.2 Hz, 1H), 7.88 – 7.81 (m, 3H), 7.80 – 7.72 (m, 2H), 7.70 (d, *J* = 8.5 Hz, 1H), 7.62 (s, 1H), 7.47 – 7.38 (m, 3H), 7.37 – 7.27 (m, 4H), 7.21 (dd, *J* = 8.5, 1.9 Hz, 2H), 7.16 (dd, *J* = 8.1, 6.3 Hz, 2H), 7.13 – 7.08 (m, 2H), 6.91 (d, *J* = 6.8 Hz, 2H), 6.49 (s, 1H), 4.55 – 4.00 (m, 1H), 3.43 – 2.88 (m, 2H), 2.43 (td, *J* = 15.3, 7.5 Hz, 1H), 2.27 – 1.95 (m, 1H), 1.03 (d, *J* = 6.6 Hz, 6H), 0.95 (d, *J* = 6.7 Hz, 6H).

^{13}C NMR (101 MHz, $CDCl_3$) δ 152.3, 148.1 (d, *J* = 9.2 Hz), 144.8 (d, *J* = 10.3 Hz), 141.7 (d, *J* = 8.2 Hz), 134.0, 133.9, 133.5, 132.3, 131.3, 130.3, 130.2, 129.5, 128.4, 128.3, 128.23, 128.17, 127.9, 127.8, 127.6, 127.4, 126.7, 126.6, 126.3, 126.2, 126.0,

125.6, 125.6, 125.0, 123.7, 122.2 (d, *J* = 5.7 Hz), 121.4 (d, *J* = 2.6 Hz), 120.0, 116.9, 46.3 (d, *J* = 4.5 Hz), 45.1, 34.2 (d, *J* = 128.6 Hz), 22.9 (d, *J* = 1.5 Hz), 22.5 (d, *J* = 1.9 Hz).

^{31}P-NMR (162 MHz, $CDCl_3$) δ 33.11.

HRMS (ESI+, m/z) calculated for $C_{44}H_{42}NO_3PNa$ $[M+Na]^+$: 686.2795 found 686.2798.

(*R*)-2'-hydroxy-[1,1'-binaphthalen]-2-yl (*R*)-*N*, *N*-diisopropyl-*P*-((*R*)-2-(4-methoxyphenyl)-2-(naphthalen-2-yl)ethyl)phosphonamidate (25)

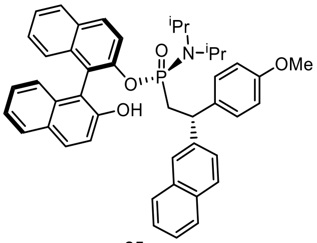

25

Following General Procedure C on 0.1 mmol scale using *R*-**3f** and 4-methoxyphenylmagnesium bromide (1 M in THF). Purification by flash chromatography (3:1 hexanes/EtOAc) afforded 49.9 mg (72%) of the title compound as a white solid.

Diastereomeric purity: 6:89:0:5 *d.r.* (crude, ^{31}P-NMR in $CDCl_3$), 95:5 *d.r.* (after purification, ^{31}P-NMR in $CDCl_3$).

Specific rotation: $[\alpha]_D^{20} = 65.2$ (c = 1.2, CH_2Cl_2).

^{1}H NMR (400 MHz, $CDCl_3$) δ 7.93 (dd, *J* = 14.3, 8.6 Hz, 2H), 7.87 – 7.79 (m, 3H), 7.79 – 7.72 (m, 2H), 7.69 (d, *J* = 8.5 Hz, 1H), 7.58 (s, 1H), 7.47 – 7.38 (m, 3H), 7.37 – 7.28 (m, 5H), 7.26 – 7.21 (m, 1H), 7.18 (dd, *J* = 8.5, 1.8 Hz, 1H), 7.14 – 7.05 (m, 1H), 6.82 (d, *J* = 8.7 Hz, 2H), 6.68 (d, *J* = 8.7 Hz, 2H), 6.49 (s, 1H), 4.28 – 4.02 (m, 1H), 3.71 (s, 3H), 3.05 (dp, *J* = 19.9, 6.7 Hz, 2H), 2.37 (td, *J* = 15.4, 7.1 Hz, 1H), 2.19 – 1.96 (m, 1H), 1.02 (d, *J* = 6.7 Hz, 6H), 0.95 (d, *J* = 6.7 Hz, 6H).

^{13}C NMR (101 MHz, $CDCl_3$) δ 158.0, 152.3, 148.1 (d, *J* = 9.2 Hz), 142.2 (d, *J* = 8.9 Hz), 136.9 (d, *J* = 9.8 Hz), 134.0, 133.9, 133.5, 132.3, 131.3, 130.3, 130.2, 129.5, 128.8, 128.3, 128.22, 128.16, 127.9, 127.6, 127.3, 126.7, 126.6, 126.0, 125.9, 125.62, 125.56, 125.5, 125.0, 123.7, 122.2 (d, *J* = 5.7 Hz), 121.5 (d, *J* = 2.6 Hz), 119.8, 116.9, 113.7, 55.3, 46.3 (d, *J* = 4.5 Hz), 44.2 (d, *J* = 2.3 Hz), 34.3 (d, *J* = 128.3 Hz), 23.0 (d, *J* = 1.5 Hz), 22.5 (d, *J* = 1.9 Hz).

^{31}P-NMR (162 MHz, $CDCl_3$) δ 33.27.

HRMS (ESI+, m/z) calculated for $C_{45}H_{44}NO_4PNa$ $[M+Na]^+$: 716.2900 found 716.2894.

(*R*)-2'-hydroxy-[1,1'-binaphthalen]-2-yl (*R*)-*N*, *N*-diisopropyl-*P*-((*R*)-2-(6-methoxypyridin-3-yl)propyl)phosphonamidate (26)

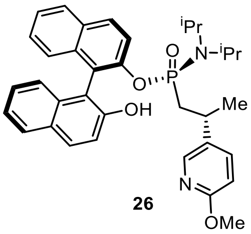

26

Following General Procedure C on 0.1 mmol scale using *R*-**3g** and methylmagnesium bromide (3 M in Et_2O). Purification by flash chromatography (3:1 hexanes/EtOAc) afforded 49.6 mg (85%) of the title compound as a white solid.

Diastereomeric purity: 78:3:3:16 *d.r.* (crude, ^{31}P-NMR in $CDCl_3$), 97:3 *d.r.* (after purification, ^{31}P-NMR in $CDCl_3$).

Specific rotation: $[\alpha]_D^{20} = 65.9$ (c = 1.38, CH_2Cl_2).

^{1}H NMR (400 MHz, $CDCl_3$) δ 7.98 (d, *J* = 9.0 Hz, 1H), 7.92 (d, *J* = 8.8 Hz, 2H), 7.85 (d, *J* = 8.1 Hz, 1H), 7.80 (d, *J* = 2.5 Hz, 1H), 7.75 (dd, *J* = 9.0, 1.1 Hz, 1H), 7.48 – 7.37 (m, 2H), 7.35 – 7.27 (m, 3H), 7.20 (ddd, *J* = 15.8, 8.6, 2.0 Hz, 2H), 7.05 (dd,

J = 8.4, 1.2 Hz, 1H), 6.95 (s, 1H), 6.62 (d, J = 8.7 Hz, 1H), 3.87 (s, 3H), 3.27 – 3.05 (m, 2H), 2.84 – 2.61 (m, 1H), 1.60 (td, J = 16.0, 3.4 Hz, 1H), 1.37 – 1.26 (m, 1H), 1.13 (d, J = 6.6 Hz, 6H), 1.08 (d, J = 6.7 Hz, 6H), 1.02 (d, J = 6.9 Hz, 3H).

^{13}C NMR (101 MHz, $CDCl_3$) δ 162.9, 152.6, 147.8 (d, J = 9.5 Hz), 144.5, 137.1, 135.9, 135.7, 134.0, 133.9, 131.4, 130.2, 130.1, 129.4, 128.2, 128.1, 127.3, 126.6, 125.7 (d, J = 11.5 Hz), 124.9, 123.7, 123.0 (d, J = 5.4 Hz), 121.4 (d, J = 2.5 Hz), 120.2, 117.6, 110.8, 53.5, 46.2 (d, J = 4.7 Hz), 35.8 (d, J = 127.4 Hz), 30.9 (d, J = 2.2 Hz), 23.1, 22.6 (d, J = 1.7 Hz).

^{31}P-NMR (162 MHz, $CDCl_3$) δ 34.58.

HRMS (ESI+, m/z) calculated for $C_{35}H_{40}N_2O_4P$ $[M+H]^+$: 583.2720 found 583.2721.

(*R*)-2'-hydroxy-[1,1'-binaphthalen]-2-yl (*R*)-*N*, *N*-diisopropyl-*P*-((*R*)-2-(6-methoxypyridin-3-yl)-2-phenylethyl)phosphonamidate (27)

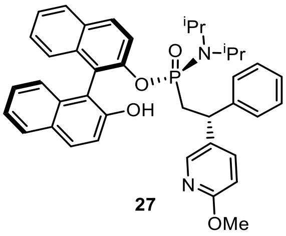

27

Following General Procedure C on 0.1 mmol scale using *R*-**3g** and phenylmagnesium bromide (1 M in THF). Purification by flash chromatography (3:1 hexanes/EtOAc) afforded 32.9 mg (51%) of the title compound as a white solid.

Diastereomeric purity: 7:0:9:84 *d.r.* (crude, ^{31}P-NMR in $CDCl_3$), 99:1 *d.r.* (after purification, ^{31}P-NMR in $CDCl_3$).

Specific rotation: $[\alpha]_D^{20} = 52.5$ (c = 1.26, CH_2Cl_2).

^{1}H NMR (400 MHz, $CDCl_3$) δ 8.02 – 7.88 (m, 3H), 7.82 (td, J = 9.0, 2.0 Hz, 3H), 7.43 (ddd, J = 8.2, 6.7, 1.3 Hz, 1H), 7.38 – 7.27 (m, 4H), 7.25 – 7.19 (m, 2H), 7.18 – 7.04 (m, 4H), 6.78 – 6.69 (m, 2H), 6.61 (d, J = 8.6 Hz, 1H), 6.49 (s, 1H), 3.94 – 3.86 (m, 1H), 3.85 (s, 3H), 3.30 – 2.93 (m, 2H), 2.36 – 2.12 (m, 1H), 2.08 – 1.90 (m, 0H), 1.05 (d, J = 6.7 Hz, 6H), 1.00 (d, J = 6.8 Hz, 6H).

^{13}C NMR (101 MHz, $CDCl_3$) δ 162.9, 152.3, 147.9 (d, J = 9.1 Hz), 145.9, 144.8 (d, J = 12.2 Hz), 138.6, 133.92, 133.90, 132.22, 132.16, 131.3, 130.3, 130.2, 129.4, 128.6, 128.3, 127.44, 127.36, 126.6, 126.5, 125.7, 125.6, 124.9, 123.7, 121.8 (d, J = 6.1 Hz), 121.1 (d, J = 3.0 Hz), 119.6, 116.6, 110.6, 53.5, 46.3 (d, J = 4.6 Hz), 42.0 (d, J = 3.0 Hz), 34.8 (d, J = 128.3 Hz), 23.0 (d, J = 1.8 Hz), 22.5 (d, J = 1.7 Hz).

^{31}P-NMR (162 MHz, $CDCl_3$) δ 31.66.

HRMS (ESI+, m/z) calculated for $C_{40}H_{42}N_2O_4P$ $[M+H]^+$: 645.2877 found 645.2865.

(*R*)-2'-hydroxy-[1,1'-binaphthalen]-2-yl (*R*)-*N*, *N*-diisopropyl-*P*-((*R*)-2-(4-methoxyphenyl)-2-(6-methoxypyridin-3-yl)ethyl)phosphonamidate (28)

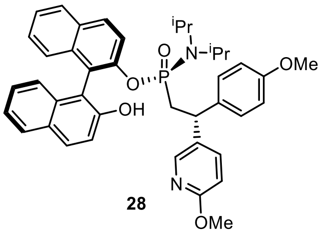

28

Following General Procedure C on 0.1 mmol scale using *R*-**3g** and 4-methoxyphenylmagnesium bromide (1 M in THF). Purification by flash chromatography (3:1 hexanes/EtOAc) afforded 46.6 mg (69%) of the title compound as a white solid.

Diastereomeric purity: 2:0:87:11 *d.r.* (crude, ^{31}P-NMR in $CDCl_3$), 98.5:1.5 *d.r.* (after purification, ^{31}P-NMR in $CDCl_3$).

Specific rotation: $[\alpha]_D^{20} = 64.2$ (c = 1.42, CH_2Cl_2).

^{1}H NMR (400 MHz, $CDCl_3$) δ 7.97 – 7.88 (m, 3H), 7.87 – 7.76 (m, 3H), 7.51 – 7.38 (m, 1H), 7.37 – 7.27 (m, 4H), 7.25 – 7.19 (m, 3H), 7.07 (d, *J* = 8.2 Hz, 1H), 6.67 (s, 3H), 6.61 (d, *J* = 8.6 Hz, 1H), 6.56 (s, 1H), 3.85 (s, 3H), 3.71 (s, 3H), 3.07 (dp, *J* = 20.1, 6.7 Hz, 2H), 2.29 – 2.11 (m, 1H), 1.98 (ddd, *J* = 17.9, 15.5, 5.4 Hz, 1H), 1.36 – 1.23 (m, 1H), 1.04 (d, *J* = 6.7 Hz, 6H), 1.00 (d, *J* = 6.8 Hz, 6H).

^{13}C NMR (101 MHz, $CDCl_3$) δ 162.8, 158.1, 152.3, 147.9 (d, *J* = 9.2 Hz), 145.8, 138.6, 136.9 (d, *J* = 11.8 Hz), 134.0, 133.9, 132.7 (d, *J* = 7.0 Hz), 131.2, 130.3, 130.2, 129.4, 128.4, 128.27, 128.25, 127.3, 126.6, 125.7, 125.6, 124.9, 123.7, 121.9 (d, *J* = 6.2 Hz), 121.1 (d, *J* = 2.9 Hz), 119.6, 116.6, 113.9, 110.6, 55.3, 53.5, 46.3 (d, *J* = 4.7 Hz), 41.2, 34.9 (d, *J* = 127.8 Hz), 23.0, 22.5 (d, *J* = 1.7 Hz).

^{31}P-NMR (162 MHz, $CDCl_3$) δ 31.80.

HRMS (ESI+, m/z) calculated for $C_{41}H_{44}N_2O_5P$ $[M+H]^+$: 675.2982 found 675.2971.

(*R*)-2'-hydroxy-[1,1'-binaphthalen]-2-yl (*R*)-*N*, *N*-diisopropyl-*P*-((*R*)-2-(6-methoxypyridin-3-yl)-2-(p-tolyl)ethyl)phosphonamidate (29)

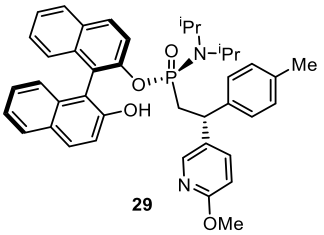

29

Following General Procedure C on 0.1 mmol scale using *R*-**3g** and *p*-tolylmagnesium bromide (1 M in THF). Purification by flash chromatography (3:1 hexanes/EtOAc) afforded 49.4 mg (75%) of the title compound as a white solid.

Diastereomeric purity: 5:0:90:5 *d.r.* (crude, ^{31}P-NMR in $CDCl_3$), 97:3 *d.r.* (after purification, ^{31}P-NMR in $CDCl_3$).

Specific rotation: $[\alpha]_D^{20} = 62.7$ (c = 1.33, CH_2Cl_2).

^{1}H NMR (400 MHz, $CDCl_3$) δ 8.00 – 7.88 (m, 3H), 7.87 – 7.80 (m, 3H), 7.48 – 7.39 (m, 1H), 7.38 – 7.27 (m, 4H), 7.26 – 7.15 (m, 2H), 7.07 (d, *J* = 8.4 Hz, 1H), 6.95 (d, *J* = 7.9 Hz, 2H), 6.65 (d, *J* = 8.1 Hz, 2H), 6.60 (d, *J* = 8.6 Hz, 1H), 6.48 (s, 1H), 3.84 (s, 3H), 3.84 – 3.78 (m, 1H), 3.27 – 2.92 (m, 2H), 2.25 (s, 3H), 2.23 – 2.13 (m, 1H), 2.05 – 1.92 (m, 1H), 1.04 (d, *J* = 6.6 Hz, 6H), 1.00 (d, *J* = 6.7 Hz, 6H).

^{13}C NMR (101 MHz, $CDCl_3$) δ 162.8, 152.3, 148.0 (d, *J* = 9.0 Hz), 145.8, 141.8 (d, *J* = 11.9 Hz), 138.6, 136.0, 133.93, 133.88, 132.5, 132.4, 131.2, 130.3, 130.2, 129.4, 129.2, 128.3, 127.34, 127.29, 126.6, 125.64, 125.56, 124.9, 123.7, 121.7 (d, *J* = 6.0 Hz), 121.1 (d, *J* = 2.8 Hz), 119.6, 116.5, 110.6, 53.5, 46.3 (d, *J* = 4.7 Hz), 41.6 (d, *J* = 3.0 Hz), 34.8 (d, *J* = 127.9 Hz), 23.0, 22.5 (d, *J* = 1.7 Hz), 21.1.

^{31}P-NMR (162 MHz, $CDCl_3$) δ 31.65.

HRMS (ESI+, m/z) calculated for $C_{41}H_{44}N_2O_4P$ $[M+H]^+$: 659.3033 found 659.3026.

(*R*)-2'-hydroxy-[1,1'-binaphthalen]-2-yl (*R*)-*N*, *N*-diisopropyl-P-((*R*)-2-(6-methoxypyridin-3-yl)-2-(thiophen-2-yl)ethyl)phosphonamidate (30)

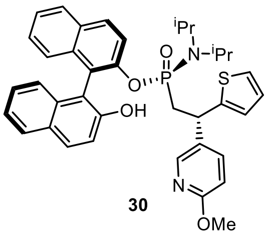

30

Following General Procedure C on 0.1 mmol scale using *R*-**3g** and 2-thienylmagnesium bromide (1 M in THF). Purification by flash chromatography (3:1 hexanes/EtOAc) afforded 60.1 mg (92%) of the title compound as a white solid.

Diastereomeric purity: 92:0:0:8 *d.r.* (crude, ^{31}P-NMR in $CDCl_3$), 99:1 *d.r.* (after purification, ^{31}P-NMR in $CDCl_3$).

Specific rotation: $[\alpha]_D^{20} = 57.9$ (c = 1.56, CH_2Cl_2).

^{1}H NMR (400 MHz, $CDCl_3$) δ 8.01 (d, *J* = 2.5 Hz, 1H), 7.93 (dd, *J* = 14.4, 8.6 Hz, 2H), 7.86 – 7.79 (m, 3H), 7.44 (ddd, *J* = 8.1, 6.6, 1.4 Hz, 1H), 7.38 (dd, *J* = 8.6, 2.6 Hz, 1H), 7.36 – 7.28 (m, 3H), 7.27 (d, *J* = 0.9 Hz, 1H), 7.25 – 7.19 (m, 1H), 7.11 – 7.00 (m, 2H), 6.78 (dd, *J* = 5.1, 3.5 Hz, 1H), 6.63 (d, *J* = 8.6 Hz, 1H), 6.44 (d, *J* = 3.5 Hz, 1H), 6.33 (s, 1H), 4.27 – 4.05 (m, 1H), 3.85 (s, 3H), 3.28 – 2.92 (m, 2H), 2.32 – 2.01 (m, 2H), 1.01 (d, *J* = 6.7 Hz, 6H), 0.96 (d, *J* = 6.8 Hz, 6H).

^{13}C NMR (101 MHz, $CDCl_3$) δ 163.1, 152.1, 149.1 (d, *J* = 14.5 Hz), 148.0 (d, *J* = 9.1 Hz), 145.9, 138.3, 133.9, 132.31, 132.25, 131.2, 130.4, 130.3, 129.4, 128.33, 128.27, 127.4, 126.7, 126.6, 125.63, 125.62, 124.9, 124.14, 124.06, 123.7, 121.2 (d, *J* = 6.2 Hz), 121.0 (d, *J* = 3.0 Hz), 119.3, 116.3, 110.8, 53.5, 46.3 (d, *J* = 4.6 Hz), 37.8 (d, *J* = 2.6 Hz), 36.1 (d, *J* = 128.6 Hz), 23.0 (d, *J* = 1.8 Hz), 22.4 (d, *J* = 1.8 Hz).

^{31}P-NMR (162 MHz, $CDCl_3$) δ 30.25.

HRMS (ESI+, m/z) calculated for $C_{38}H_{40}N_2O_4PS$ $[M+H]^+$: 651.2441 found 651.2433.

7. X-ray diffraction analysis and determination of absolute configuration

Single crystals of (*S*,S*p*,*R*)-**5**' were obtained by slow diffusion of heptane into a solution of (*S*,S*p*,*R*)-**5**' in DCM. A suitable crystal was selected and analyzed on a Bruker-AXS D8 Venture diffractometer, and the Bruker APEX4 software suite was used for data collection and processing. A suitable crystal was selected and mounted on a cryoloop and placed in the cold nitrogen stream (100 K) of a Bruker-AXS D8 Venture diffractometer with a Cu Kα (λ = 1.54178 Å) source. Data collection and processing was done using the Bruker APEX4 software suite.[5]The structure was solved using SHELXT[6] and refinement was performed with SHELXL[6] using the OLEX2 software package.[7] A multi-scan absorption correction was applied, based on the intensities of symmetry-related reflections measured at different angular settings (SADABS-2016/2).[8] Contributions from disordered solvent were removed using the PLATON/SQUEEZE routine.[9] Hydrogen atoms were generated by geometrical considerations and refined using a riding model. No A- or B-level alerts were raised by CheckCIF for the fully refined structure. CCDC 2526188 contains the supplementary crystallographic data for this paper. These data can be obtained free of charge from The Cambridge Crystallographic Data Centre via www.ccdc.cam.ac.uk/data_request/cif. The crystal structure allowed us to determine the absolute configuration of the stereogenic centers, being (*S*,S*p*,*R*). By analogy, we assign the opposite (*R*,*Rp*,*S*) configuration to other phosphonamidate derived from *R*-BINOL.

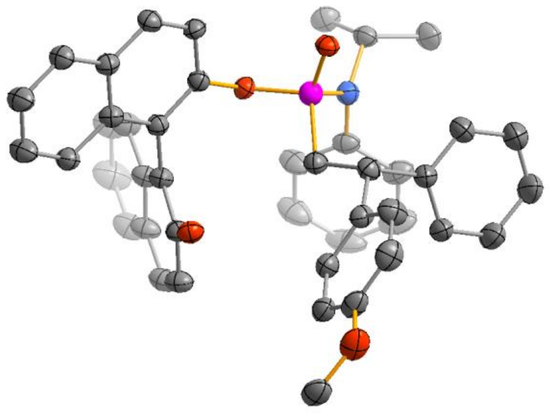

Crystal data and structure refinement for (*S*,S*p*,*R*)-**5**'

Empirical formula	$C_{44}H_{40}NO_4P$
Formula weight	677.74
Temperature/K	100
Crystal system	tetragonal
Space group	$P4_3$
a/Å	12.7645(3)
b/Å	12.7645(3)
c/Å	25.9354(10)
α/°	90
β/°	90
γ/°	90
Volume/Å^3	4225.7(3)

Z	4
ρ_{calc}g/cm^3	1.065
μ/mm^{-1}	0.876
F(000)	1432.0
Crystal size/mm^3	0.443 × 0.068 × 0.053
Radiation	CuKα (λ = 1.54178)
2Θ range for data collection/°	6.924 to 136.486
Index ranges	-15 ≤ h ≤ 15, -15 ≤ k ≤ 15, -31 ≤ l ≤ 30
Reflections collected	154703
Independent reflections	7740 [R_{int} = 0.1166, R_{sigma} = 0.0394]
Data/restraints/parameters	7740/1/455
Goodness-of-fit on F^2	1.078
Final R indexes [I>=2σ (I)]	R_1 = 0.0547, wR_2 = 0.1423
Final R indexes [all data]	R_1 = 0.0588, wR_2 = 0.1462
Largest diff. peak/hole / e Å^{-3}	0.21/-0.50
Flack parameter	0.084(11)

8. DFT structural analysis of alkenylphosphonium salt *R*-3a

Electronic structure calculations of alkenylphosphonium salt ***R*-3a** were performed using the Gaussian 09 software package.[10] The geometries of all minima were optimized at the ωb97XD level[11-12] within the self-consistent reaction field (SCRF) in the gas phase. The 6-31+G(d,p) basis set[13-14] was employed for all atoms. Frequency calculations were performed at the same level to evaluate the zero-point vibrational energy and thermal corrections at 298 K and to confirm the nature of the stationary points. 3D representation were generated using CYLview.[15]

Cartesian coordinates in Å for *R*-3a . 6-31G(d,p)-optimized geometries

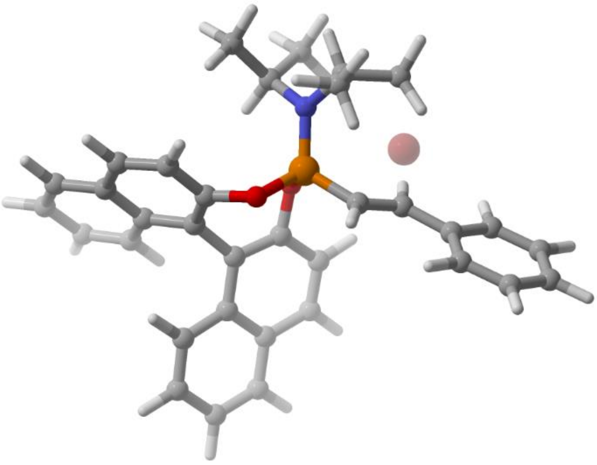

Figure S 4 DFT optimized structure of ***R*-3a.**

Electronic energy (6-31+G(d,p) = -4433.448726 Hartree

Zero-point Energy Correction = 0.594271 Hartree

Thermal correction to Energy = 0.629337 Hartree

Thermal correction to Enthalpy = 0.630282 Hartree

Thermal correction to Free Energy = 0.526287 Hartree

Charge = 0 Multiplicity = 1

P	0.53514	-0.97015	-0.63474
O	-0.0825	-0.38369	0.7123
O	-0.62957	-0.72702	-1.74117
N	0.83235	-2.51469	-0.26575
C	4.92834	1.46563	0.05111
H	4.73675	1.08889	1.0545
C	-0.32903	3.1771	1.35273
H	0.21039	3.96073	1.87621
C	2.84301	0.38512	-0.57691
H	2.82894	0.10744	0.48736
C	3.99806	1.18843	-0.96138
C	2.00158	-3.29077	-0.77568
H	1.78761	-4.30607	-0.43722
C	1.83638	-0.04742	-1.35987
H	1.7145	0.18735	-2.41309
C	-1.95861	4.85802	0.55645

H	-1.39259	5.62461	1.07812
C	4.22401	1.66978	-2.25975
H	3.5092	1.46659	-3.05206
C	-1.50911	3.5132	0.63439
C	0.14522	1.8944	1.36809
H	1.04149	1.58186	1.90246
C	-1.79899	1.13511	0.05792
C	-5.86631	0.15779	1.2659
H	-6.34328	0.7257	2.05813
C	-0.60234	0.90998	0.69609
C	5.36344	2.4109	-2.53941
H	5.53531	2.78372	-3.54411
C	-0.07337	-3.21691	0.70064
H	-0.85064	-2.49888	0.96734
C	-1.9683	-0.88391	-1.39451
C	6.0725	2.20412	-0.2353
H	6.79086	2.41205	0.55082
C	-5.96927	-1.61579	-0.36089
H	-6.5005	-2.42514	-0.85393
C	6.28856	2.67821	-1.52648
C	-2.24887	2.49444	-0.0329
C	-2.65777	-1.94203	-2.01904
H	-2.12723	-2.55577	-2.73862
C	-3.97234	-2.15674	-1.71116
H	-4.52289	-2.96058	-2.1907
C	-4.57277	0.44519	0.91068
H	-4.0375	1.23419	1.42564
C	0.6772	-3.57364	1.98015
H	1.43673	-4.34289	1.80185
H	-0.03034	-3.9716	2.71386
H	1.16762	-2.68894	2.40005
C	-3.91688	-0.28726	-0.11623
C	-4.62572	-1.35434	-0.73958
C	-3.38837	2.87283	-0.7927
H	-3.9425	2.11438	-1.33453
C	-6.58113	-0.87478	0.61629
H	-7.60613	-1.08618	0.90297
C	-0.76269	-4.41092	0.04148
H	-1.28729	-4.10899	-0.86919
H	-1.49835	-4.82545	0.73672
H	-0.06159	-5.21415	-0.20642
C	-2.56257	-0.01088	-0.50907

C	-3.07391	5.18952	-0.16901
H	-3.40393	6.22188	-0.22405
C	-3.78809	4.18379	-0.85992
H	-4.65793	4.45118	-1.45149
C	3.33975	-2.8809	-0.15485
H	3.79088	-2.04044	-0.6876
H	4.03598	-3.723	-0.22376
H	3.22817	-2.5935	0.89439
C	2.03802	-3.33099	-2.30228
H	1.08049	-3.66837	-2.71034
H	2.81899	-4.02344	-2.6299
H	2.27611	-2.34967	-2.7251
H	7.17942	3.2572	-1.75017
Br	2.73596	-0.41519	2.73697

9. Derivatization reactions

Gram scale for the synthesis of 5

A dry vial was charged with *R*-**3a** (2.0 g, 3.35 mmol 1.0 equiv). The vial was sealed with a septum and evacuated and backfilled with N_2 three times. Next, anhydrous 1,4-Dioxane (10 mL) was added, followed by slow addition of 4-methoxyphenylmagnesium bromide (6.7 mL, 6.7 mmol, 2.0 equiv, 1 M) and the reaction mixture was stirred at room temperature for 18 hours. Then, the reaction mixture was hydrolyzed with H_2O and stirred at room temperature for 8-12 h. The reaction mixture was extracted with DCM (3x), and combined organic extracts were dried over Na_2SO_4, filtered, and concentrated under reduced pressure. The crude material was purified by flash column chromatography on silica gel using hexane/EtOAc 2:1 to afford product **5** as a white solid (1.77 g, 2.75 mmol, 82%, 95:5 *d.r.* after purification).

General procedure C for the removal displacement of amino group and protection of OH group

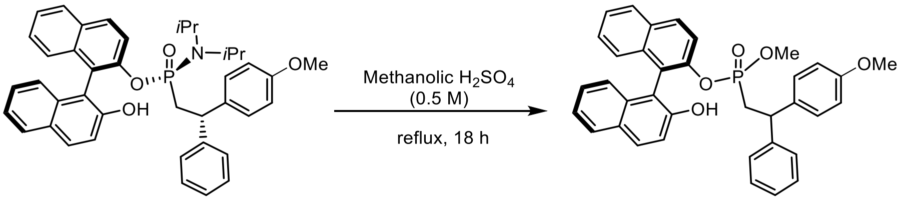

5 **31**

A flame-dried three-necked flask was charged with **5** (1.0 g, 1.55 mmol). After three vacuum-N_2 cycles, 15 ml of 1 M anhydrous methanolic H_2SO_4 was added. The reaction mixture was heated at reflux for 48 h. Next, 30 mL of aq. $NaHCO_3$ were slowly added and the mixture was stirred for additional 30 min. The methanol was evaporated and the aqueous phase was extracted with EtOAc (3 x 20mL). Combined organic layers were washed with brine (100 mL) and dried over Na_2SO_4. All volatiles were removed under reduced pressure to afford the crude product. Purification by flash chromatography using ether as eluent afforded **31** as a white solid (88:12 *d.r.*, 0.16 g, 0.28 mmol, 28%).

(*R*)-2'-hydroxy-[1,1'-binaphthalen]-2-yl methyl (2-(4-methoxyphenyl)-2-phenylethyl)phosphonate (31)

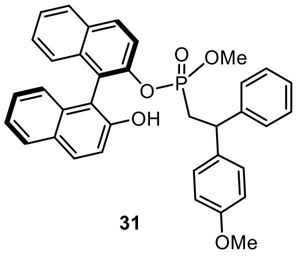

31

Diastereomeric purity: 88:12 *d.r.* (crude, ^{31}P-NMR in $CDCl_3$), 88:12 *d.r.* (after purification, ^{31}P-NMR in $CDCl_3$).

Specific rotation: $[\alpha]_D^{20} = 56.0$ (c = 0.1, CH_2Cl_2).

^{1}H NMR (600 MHz, $CDCl_3$) δ 7.95 (d, *J* = 9.0 Hz, 1H), 7.91 (t, *J* = 7.9 Hz, 2H), 7.84 (d, *J* = 8.1 Hz, 1H), 7.46 (t, *J* = 7.5 Hz, 1H), 7.38 (dd, *J* = 13.2, 9.0 Hz, 2H), 7.35 – 7.29 (m, 2H), 7.26 – 7.18 (m, 4H), 7.14 (d, *J* = 7.2 Hz, 1H), 7.06 – 7.00 (m, 3H), 6.97 (d, *J* = 8.6 Hz, 2H), 6.75 (d, *J* = 8.6 Hz, 2H), 4.12 (t, *J* = 6.9 Hz, 1H), 3.74 (s, 3H), 2.82 (s, 3H), 2.54 – 2.31 (m, 2H), 1.59 (s, 1H).

^{13}C NMR (151 MHz, , $CDCl_3$) δ 158.35, 152.40, 147.32 (d, *J* = 8.3 Hz), 144.06 (d, *J* = 12.5 Hz), 135.80 (d, *J* = 8.8 Hz), 133.84 (d, *J* = 4.9 Hz), 131.66, 130.99, 130.43, 129.30, 128.77, 128.63, 128.29, 128.21, 127.64, 127.44, 126.95, 126.62, 126.04,

125.82, 124.29 (d, J = 141.2 Hz), 122.65 (d, J = 4.9 Hz), 120.54 (d, J = 2.2 Hz), 119.56, 116.10, 113.96, 55.37, 52.17 (d, J = 7.5 Hz), 44.40 (d, J = 3.2 Hz), 33.10 (d, J = 140.0 Hz).

^{31}P-NMR (243 MHz, $CDCl_3$) δ 29.12.

HRMS (ESI+, m/z) calculated for $C_{36}H_{31}O_5P$ $[M+H]^+$: 575.1982 found 575.1983.

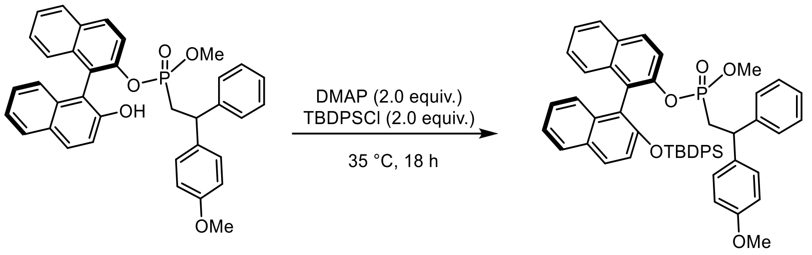

31 **31’**

A flame-dried Schlenk-tube was charged with **28** (0.1 g, 0.17 mmol, 1.00 equiv.) and DMAP (41.5 mg, 0.34 mmol, 2.00 equiv.). After three vacuum-N_2-cycles, dichloromethane (1 mL) was added, followed by TBDPSCl (93.5 mg, 0.34 mmol, 2.00 equiv.). The reaction mixture was heated to 35 °C for 18 h. When the reaction was finished, H_2O (12 mL) and EtOAc (2 mL) were added. The aqueous phase was extracted with EtOAc (3 x 2 mL) and the combined organic phases were washed with brine (5 mL) and dried over Na_2SO_4. All volatiles were removed under reduced pressure to afford the crude product. Purification by flash chromatography using ether as eluent to afford desired product **31’** (80:20 *d.r.*, 56.9 mg, 0.07 mmol, 42%) as a white solid.

(*R*)-2'-((*tert*-butyldiphenylsilyl)oxy)-[1,1'-binaphthalen]-2-yl methyl (2-(4-methoxyphenyl)-2-phenylethyl)phosphonate (31’)

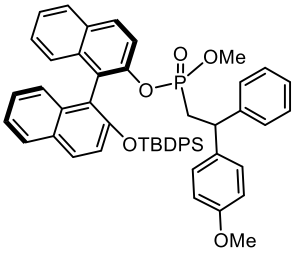

31’

Diastereomeric purity: 80:20 *d.r.* (crude, ^{31}P-NMR in $CDCl_3$), 80:20 *d.r.* (after purification, ^{31}P-NMR in $CDCl_3$).

Specific rotation: $[\alpha]_D^{20}$ = -60.7 (c = 0.27, CH_2Cl_2).

^{1}H NMR (401 MHz, $CDCl_3$) δ 7.94 (dd, J = 12.0, 8.6 Hz, 2H), 7.80 (dd, J = 8.9, 0.9 Hz, 1H), 7.73 (d, J = 8.0 Hz, 1H), 7.67 (d, J = 6.6 Hz, 2H), 7.58 – 7.47 (m, 3H), 7.46 – 7.26 (m, 10H), 7.26 – 7.22 (m, 2H), 7.18 – 7.04 (m, 3H), 6.85 (d, J = 9.0 Hz, 1H), 6.77 – 6.70 (m, 3H), 6.67 (d, J = 8.7 Hz, 2H), 3.94 – 3.86 (m, 1H), 3.73 (s, 3H), 3.10 (d, J = 11.3 Hz, 3H), 2.00 – 1.74 (m, 2H), 0.48 (s, 9H).

^{13}C NMR (151 MHz, $CDCl_3$) δ 158.14, 151.23, 146.92 (d, J = 7.9 Hz), 144.34 (d, J = 13.9 Hz), 135.81 (d, J = 7.7 Hz), 135.52, 135.49, 134.27, 134.06, 133.04, 132.49, 131.27, 130.00, 129.60, 129.28, 128.98, 128.73, 128.40, 128.21, 128.16, 127.89, 127.83, 127.62, 127.31, 126.79 (d, J = 26.2 Hz), 126.30 (d, J = 2.3 Hz), 125.38 (d, J = 51.6 Hz), 123.85, 120.55, 120.28 (d, J = 140.0 Hz), 113.76, 113.72, 55.33, 52.20 (d, J = 7.1 Hz), 44.03 (d, J = 2.8 Hz), 32.04 (d, J = 138.7 Hz), 25.65, 19.00.

^{31}P-NMR (162 MHz $CDCl_3$) δ 27.47.

NOTE: HRMS of this compound was unsuccessful.

(*R*)-2'-hydroxy-[1,1'-binaphthalen]-2-yl methyl (2-phenyl-2-(p-tolyl)ethyl)phosphonate (32)

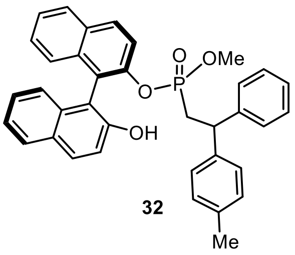

32

Following the same procedure C for displacement of the amino group, compound **13** was used as the starting material on a 2 mmol scale, affording the product in 35% yield (0.39 g, 0.7 mmol).

Diastereomeric purity: 95:5 *d.r.* (crude, ^{31}P-NMR in $CDCl_3$), 95:5 *d.r.* (after purification, ^{31}P-NMR in $CDCl_3$).

^{1}H NMR (400 MHz, Chloroform-*d*) δ 7.98 – 7.90 (m, 3H), 7.88 (d, *J* = 8.0 Hz, 1H), 7.51 – 7.40 (m, 3H), 7.39 – 7.34 (m, 1H), 7.34 – 7.28 (m, 3H), 7.28 – 7.24 (m, 1H), 7.22 (d, *J* = 7.7 Hz, 2H), 7.16 (d, *J* = 7.2 Hz, 1H), 7.09 (d, *J* = 8.6 Hz, 1H), 7.05 (d, *J* = 6.6 Hz, 3H), 6.96 (d, *J* = 8.0 Hz, 2H), 6.50 (s, 0H), 4.31 – 4.06 (m, 1H), 2.88 (d, *J* = 11.4 Hz, 3H), 2.48 – 2.36 (m, 2H), 2.30 (s, 3H).

^{31}P NMR (162 MHz, $CDCl_3$) δ 28.92.

(*R*)-2'-((*tert*-butyldiphenylsilyl)oxy)-[1,1'-binaphthalen]-2-yl methyl (2-phenyl-2-(p-tolyl)ethyl)phosphonate (32’)

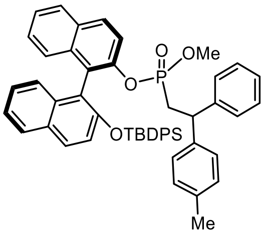

32'

Following same procedure **C** for protection of OH group using **32** as starting material on a scale of 0.6 mmol (0.17 g, 0.24 mmol, 40%).

Diastereomeric purity: 95:5 *d.r.* (crude, ^{31}P-NMR in $CDCl_3$), 99:1 *d.r.* (after purification, ^{31}P-NMR in $CDCl_3$).

Specific rotation: $[\alpha]_D^{20}$ = -48.8 (c = 2.18, CH_2Cl_2).

^{1}H NMR (400 MHz, $CDCl_3$) δ 7.97 (d, *J* = 9.0 Hz, 1H), 7.93 (d, *J* = 8.1 Hz, 1H), 7.83 (d, *J* = 9.0 Hz, 1H), 7.75 (d, *J* = 7.9 Hz, 1H), 7.69 (d, *J* = 7.2 Hz, 2H), 7.60 – 7.50 (m, 3H), 7.48 – 7.28 (m, 10H), 7.27 – 7.24 (m, 2H), 7.17 – 7.06 (m, 3H), 6.96 (d, *J* = 7.9 Hz, 2H), 6.87 (d, *J* = 8.9 Hz, 1H), 6.76 (d, *J* = 6.9 Hz, 2H), 6.72 (d, *J* = 8.0 Hz, 2H), 4.01 – 3.88 (m, 1H), 3.12 (dd, *J* = 11.3, 0.7 Hz, 3H), 2.25 (s, 3H), 2.08 – 1.79 (m, 2H), 0.50 (s, 9H).

^{31}P NMR (243 MHz, $CDCl_3$) δ 27.56.

^{13}C NMR (101 MHz, $CDCl_3$) δ 151.19, 146.91 (d, *J* = 7.6 Hz), 144.19 (d, *J* = 13.0 Hz), 135.90, 135.49, 135.46, 134.25, 134.03, 133.00, 132.47, 131.23, 129.97 (d, *J* = 2.2 Hz), 129.56, 129.25, 129.02, 128.96, 128.37, 128.16 (d, *J* = 3.5 Hz), 127.86, 127.80, 127.54, 127.34, 126.76 (d, *J* = 19.4 Hz), 126.27 (d, *J* = 2.4 Hz), 125.35 (d, *J* = 35.8 Hz), 123.84, 120.74, 120.51, 119.80, 52.15 (d, *J* = 7.1 Hz), 44.42 (d, *J* = 2.9 Hz), 31.93 (d, *J* = 138.7 Hz), 25.63, 21.07, 18.97.

HRMS (ESI+, m/z) calculated for $C_{52}H_{49}O_4PSi$ $[M+Na]^+$: 819.3030 found 819.3017.

General procedure D for the removal of BINOL auxiliary

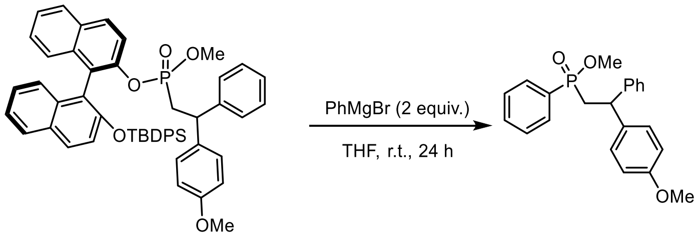

31' **33**

A flame-dried Schlenk tube was charged with **31'** (60.0 mg, 0.07 mmol, 0.1 equiv.). After three vacuum-nitrogen cycles, anhydrous THF(0.4 mL) was added. Subsequently, the phenylmagnesium bromide (0.1 mmol, 1.5 equiv., 1M) was added dropwise and the reaction mixture was stirred at 0 °C for 3 hours . After full conversion was observed by TLC sat. aq. NH_4Cl-sol. (2 mL) was added and the aqueous phase was extracted with ethyl acetate (3 x 1.5 mL). The combined organic phases were dried over Na_2SO_4 and all volatiles were removed under reduced pressure. The crude product was purified by flash chromatography with dichloromethane/methanol as eluent to get **33** (25.4 mg, 0.069 mmol, 92%).

Methyl (2-(4-methoxyphenyl)-2-phenylethyl)(phenyl)phosphinate (33)

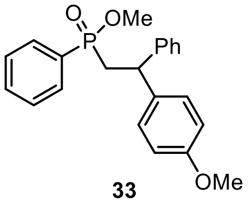

33

Diastereomeric purity: 72:28 *d.r.* (crude, ^{31}P-NMR in $CDCl_3$), 72:28 *d.r.* (after purification, ^{31}P-NMR in $CDCl_3$).

Specific rotation: $[\alpha]_D^{20} = 5.0$ (c = 0.04, CH_2Cl_2).

^{1}H NMR (600 MHz, $CDCl_3$) δ 7.54 – 7.47 (m, 2H), 7.45 – 7.40 (m, 1H), 7.33 – 7.29 (m, 2H), 7.23 (d, *J* = 2.1 Hz, 2H), 7.15 (d, *J* = 8.7 Hz, 1H), 7.09 (d, *J* = 5.7 Hz, 2H), 7.01 (d, *J* = 8.6 Hz, 2H), 6.78 (d, *J* = 8.7 Hz, 1H), 6.60 (d, *J* = 8.7 Hz, 1H), 4.53 – 4.36 (m, 1H), 3.70 (s, 3H), 3.46 (d, *J* = 11.1 Hz, 3H), 2.76 – 2.68 (m, 2H).

^{13}C NMR (151 MHz, $CDCl_3$) δ 158.17, 132.03 (d, *J* = 2.5 Hz), 131.93 (d, *J* = 2.8 Hz), 131.67 (d, *J* = 3.2 Hz), 131.61 (d, *J* = 3.2 Hz), 128.94, 128.62, 128.49, 128.43 (d, *J* = 8.5 Hz), 127.55, 127.14 (d, *J* = 187.9 Hz), 113.85, 55.34 (d, *J* = 8.2 Hz), 51.00 (d, *J* = 6.5 Hz), 44.23, 36.44 (d, *J* = 99.5 Hz).

^{31}P-NMR (162 MHz, $CDCl_3$) δ 44.20, 44.14.

HRMS (ESI+, m/z) calculated for $C_{22}H_{23}O_3P$ $[M+H]^+$: 367.1458 found 367.1459.

Methyl phenyl(2-phenyl-2-(p-tolyl)ethyl)phosphinate (34)

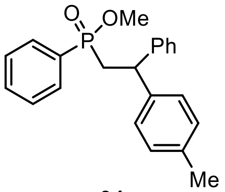

34

Following the same procedure D using 29' as starting material on a 0.06 mmol scale, **31** (14.0mg, 0.04 mmol, 67%).

Diastereomeric purity: 95:5 *d.r.* (crude, ^{31}P-NMR in $CDCl_3$), 99:1 *d.r.* (after purification, ^{31}P-NMR in $CDCl_3$).

Specific rotation: $[\alpha]_D^{20} = 1.8$ (c = 0.22, CH_2Cl_2).

^{1}**H NMR** (600 MHz, $CDCl_3$) δ 7.56 (t, *J* = 6.6 Hz, 1H), 7.51 (d, *J* = 7.3 Hz, 2H), 7.43 (q, *J* = 6.1, 4.8 Hz, 1H), 7.31 (t, *J* = 7.6 Hz, 2H), 7.24 (d, *J* = 4.3 Hz, 3H), 7.16 – 7.08 (m, 2H), 6.99 (d, *J* = 7.8 Hz, 2H), 6.88 (d, *J* = 7.7 Hz, 1H), 4.45 (t, *J* = 7.6 Hz, 1H), 3.45 (s, 3H), 2.92 – 2.59 (m, 2H), 2.20 (s, 3H).

^{31}**P NMR** (243 MHz, $CDCl_3$) δ 44.20, 29.59.

^{13}**C NMR** (151 MHz, $CDCl_3$) δ 144.48 (d, J = 9.9 Hz), 140.54 (d, J = 7.7 Hz), 136.05, 131.85, 131.66 (d, J = 9.8 Hz), 129.25 (d, J = 16.5 Hz), 128.88 (d, J = 81.8 Hz), 128.47, 128.39, 127.71 (d, J = 20.4 Hz), 127.53, 126.53, 50.99 (d, J = 6.0 Hz), 44.63, 36.37 (d, J = 99.5 Hz), 29.86.

HRMS (ESI+, m/z) calculated for $C_{22}H_{23}O_2PH$ $[M+H]^+$: 351.1508 found 351.1513.

10. Challenging substrates

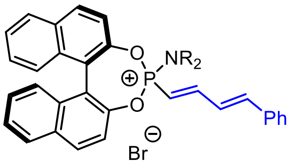

I

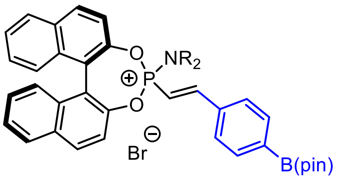

II

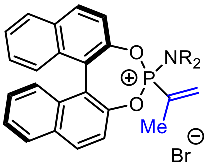

III

We attempted the synthesis of additional alkenylphosphonium salts **I–III** following General Procedure B for the preparation. Purification of phosphonium salts **I** and **II** was attempted but both compounds were found to contain minor impurities that were difficult to remove. Nevertheless, **^{31}P NMR spectra was consistent with the formation of the desired alkenyl phosphonium salts**, despite the purification challenges. Alkenylphosphonium salt **III** was not bench-stable under ambient conditions and underwent rapid hydrolysis to give the corresponding alkenylphosphonamidate previously described by us.[3] ^{1}H-NMR and ^{31}P-NMR spectra is provided below for the partially purified compounds.

^{1}H-NMR in $CDCl_3$

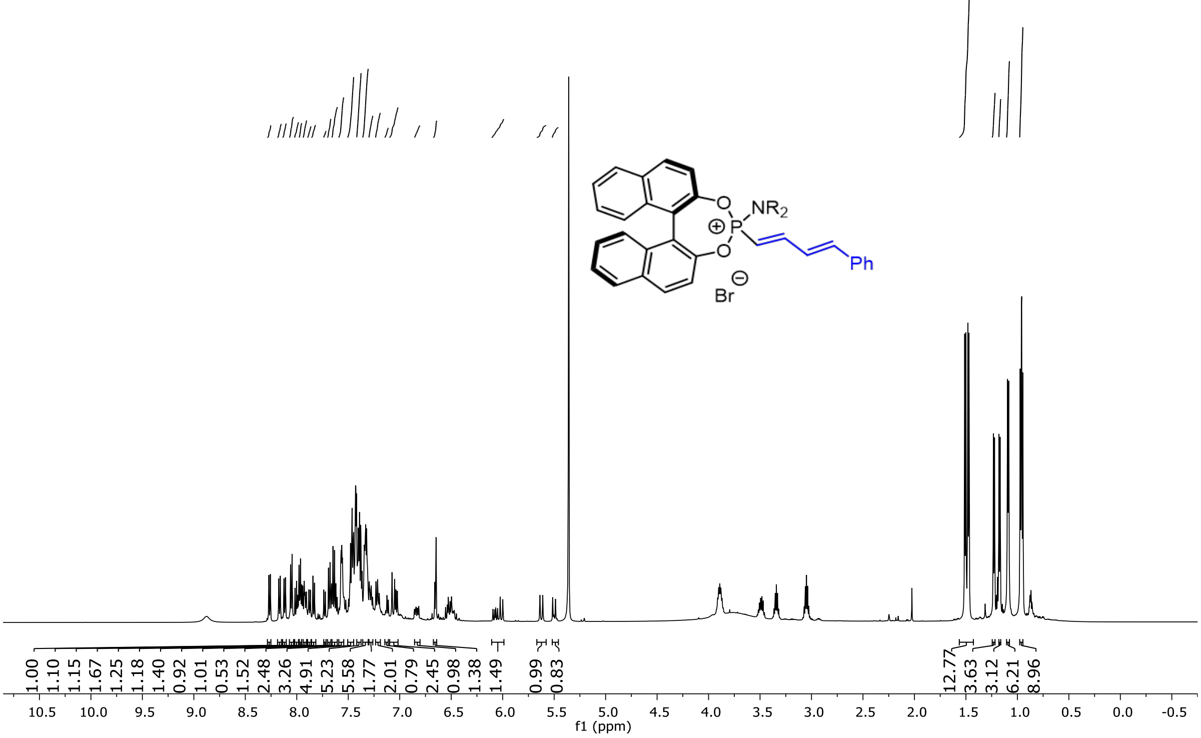

^{31}P-NMR in $CDCl_3$

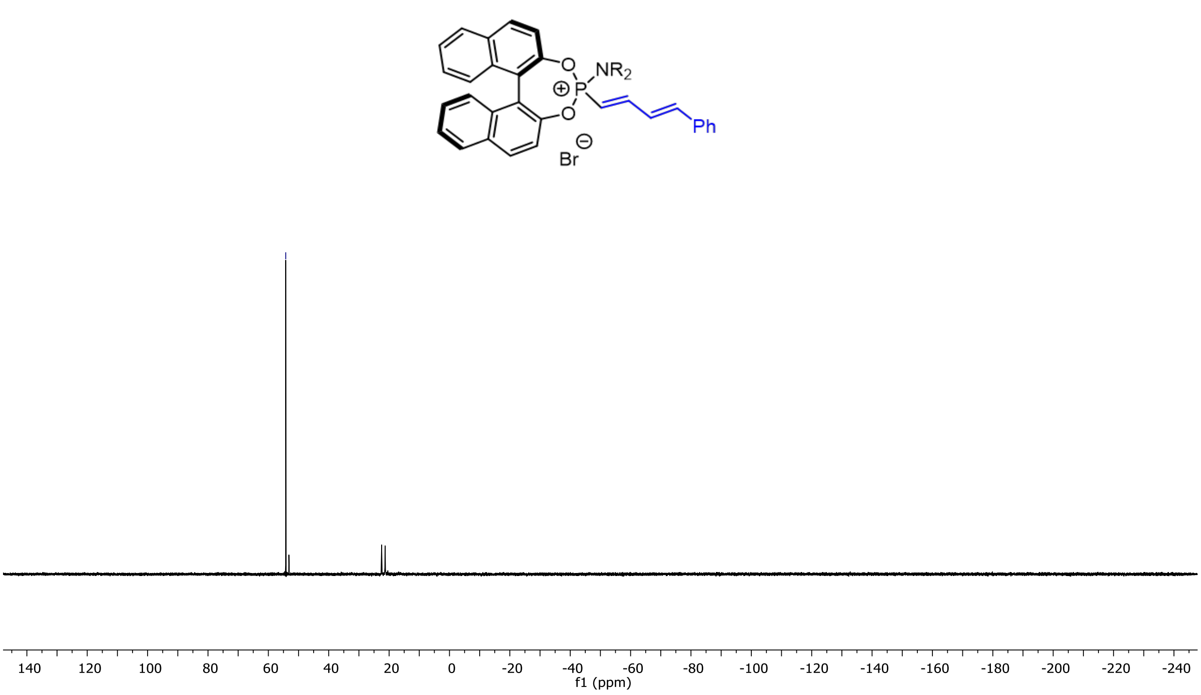

^{1}H-NMR in $CDCl_3$

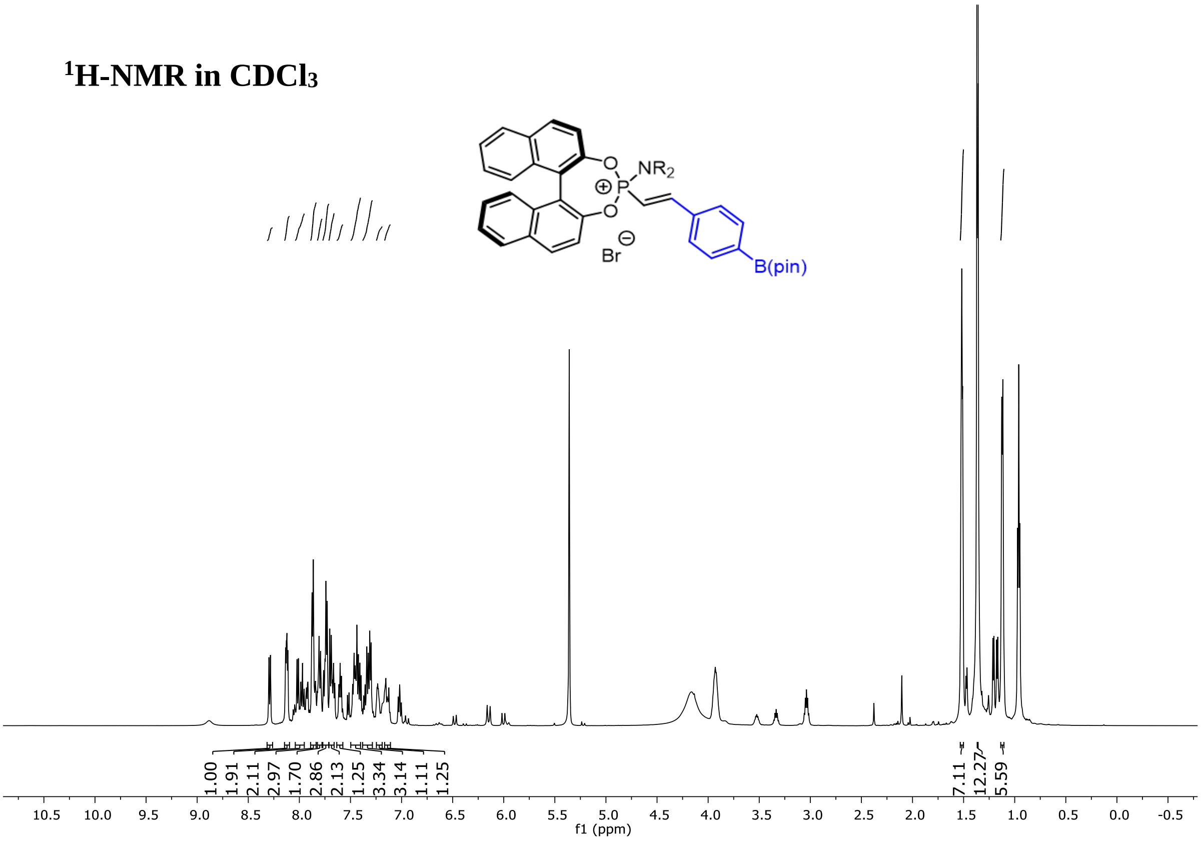

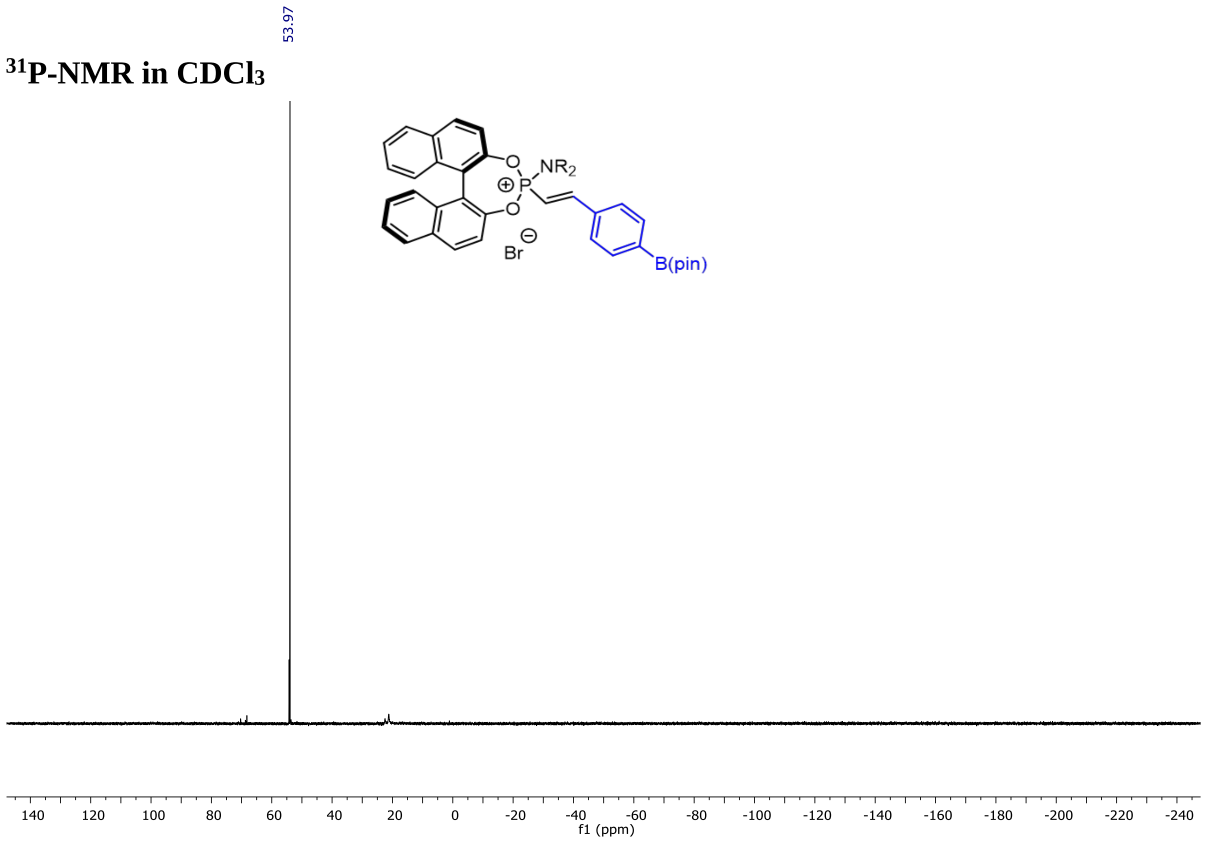

Alkenylphosphonium salt **I** was subjected to the general conditions (General Procedure C) with different Grignard reagents (Figure S 5 and Figure S 6). Analysis of the isolated products showed that, even after multiple purification attempts, the corresponding expected products **IV** and **V** contained variable amounts of unidentified impurities visible in the **^{1}H NMR** spectra, whereas their corresponding **^{31}P NMR** spectra were relatively clean. Regarding product **IV**, the **^{1}H NMR** data was consistent with the preferential formation of a **1,4-addition** product (addition at the δ carbon rather than β). However, due to the presence of impurities and the complexity of the NMR data, we could not assign all the NMR signals. ^{1}H-NMR and ^{31}P-NMR spectra is provided below for the partially purified compounds.

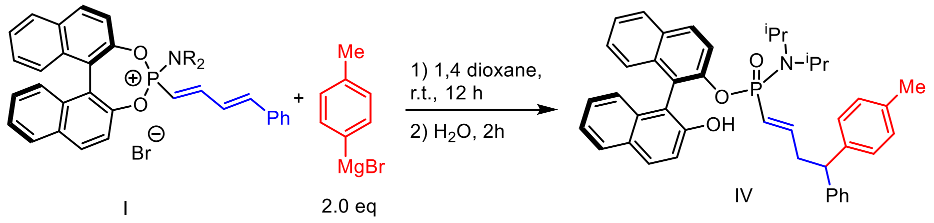

Figure S 5

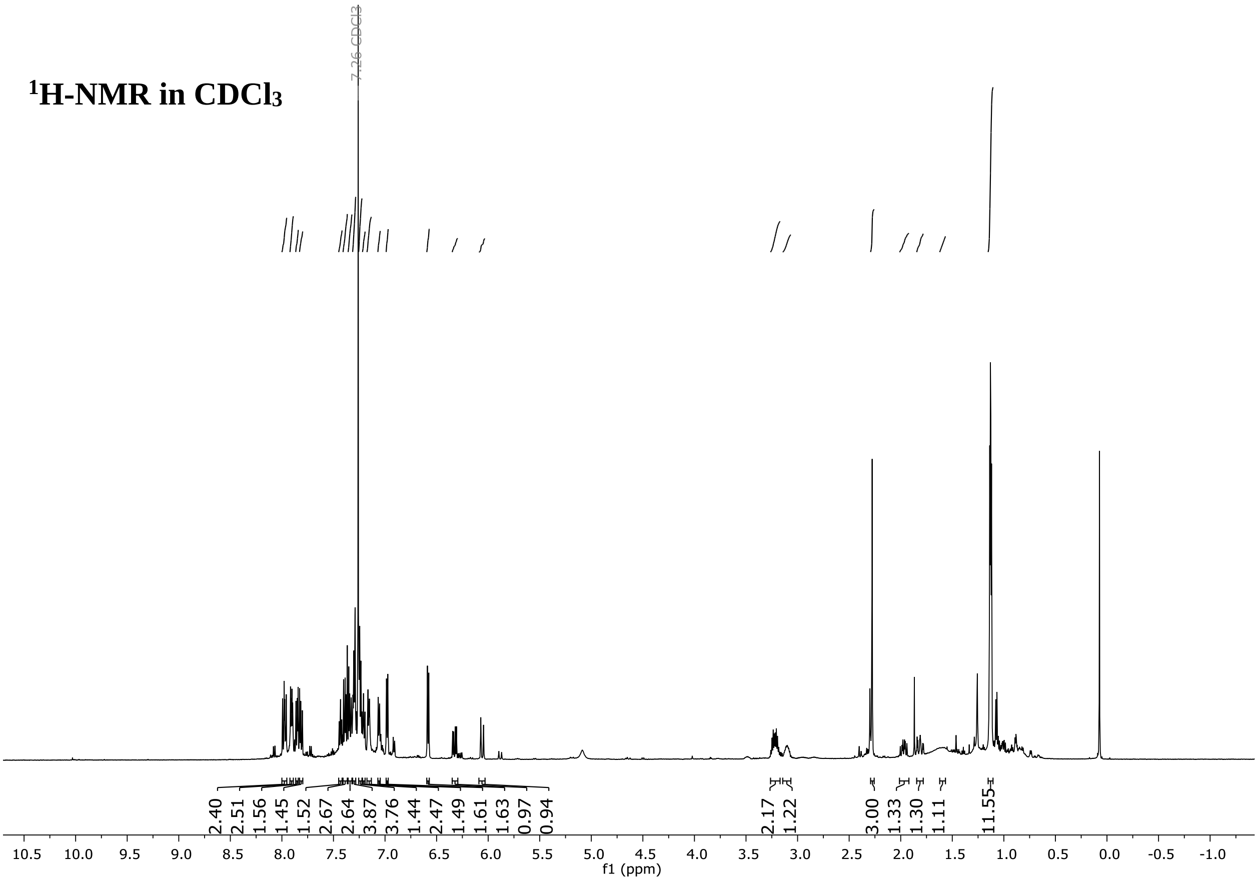

^{1}H-NMR in CDCl$_3$
7.26 CDCl3
2.40
2.51
1.56
1.45
1.52
2.67
2.64
3.87
3.76
1.44
2.47
1.49
1.61
1.63
0.97
0.94
2.17
1.22
3.00
1.33
1.30
1.11
11.55
10.5 10.0 9.5 9.0 8.5 8.0 7.5 7.0 6.5 6.0 5.5 5.0 4.5 4.0 3.5 3.0 2.5 2.0 1.5 1.0 0.5 0.0 -0.5 -1.0
f1 (ppm)

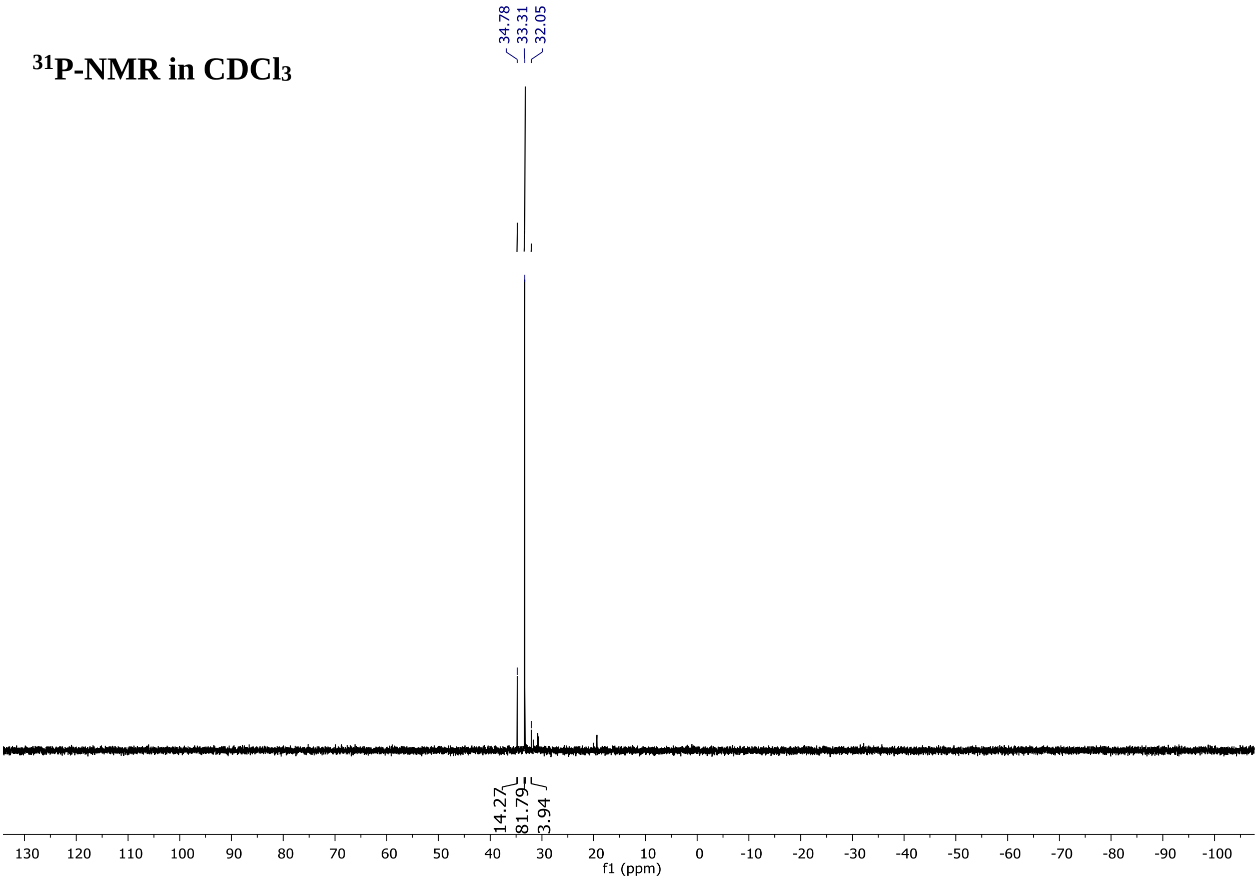

^{31}P-NMR in CDCl$_3$
34.78
33.31
32.05
14.27
81.79
3.94
130 120 110 100 90 80 70 60 50 40 30 20 10 0 -10 -20 -30 -40 -50 -60 -70 -80 -90 -100
f1 (ppm)

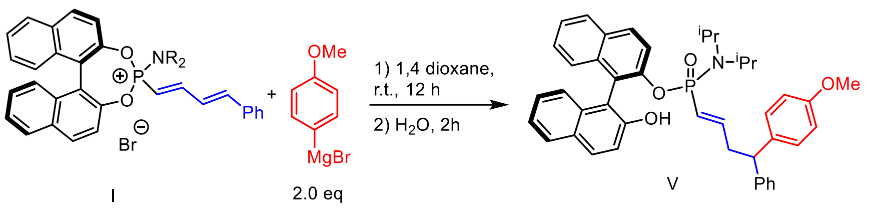

NR2
Br
Ph
OMe
MgBr
1) 1,4 dioxane, r.t., 12 h
2) H2O, 2h
iPr
N-iPr
OH
OMe
Ph
I
2.0 eq
V

Figure S 6

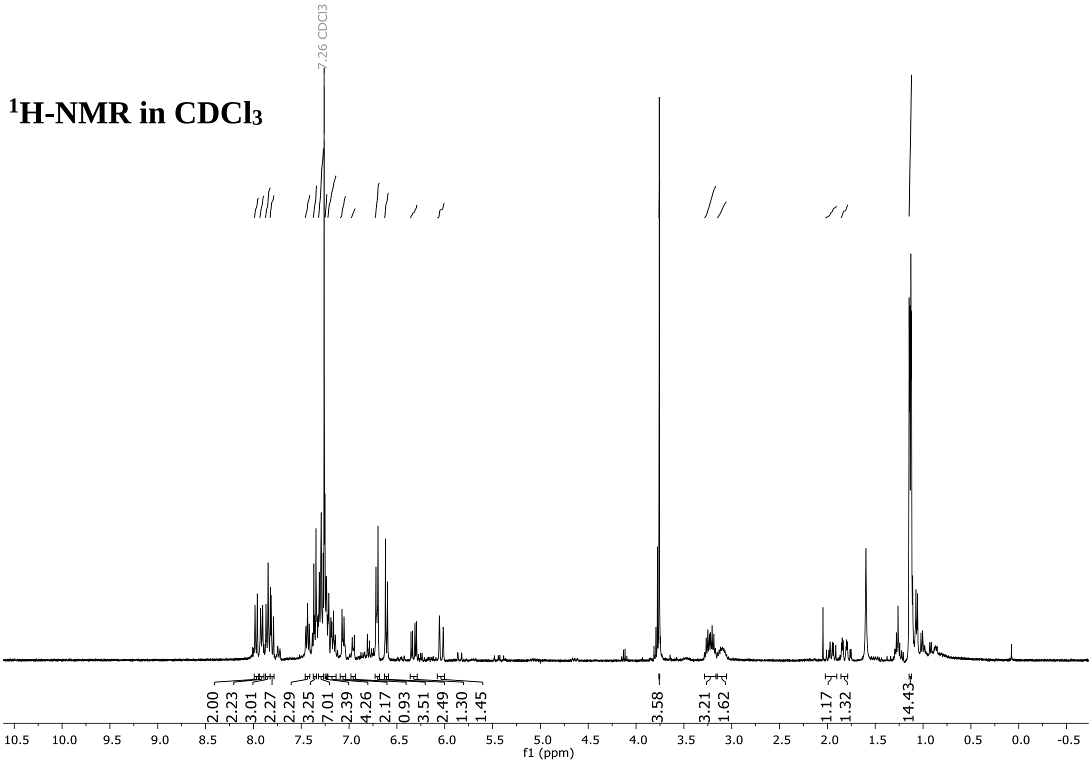

1H-NMR in CDCl3
7.26 CDCl3
f1 (ppm)

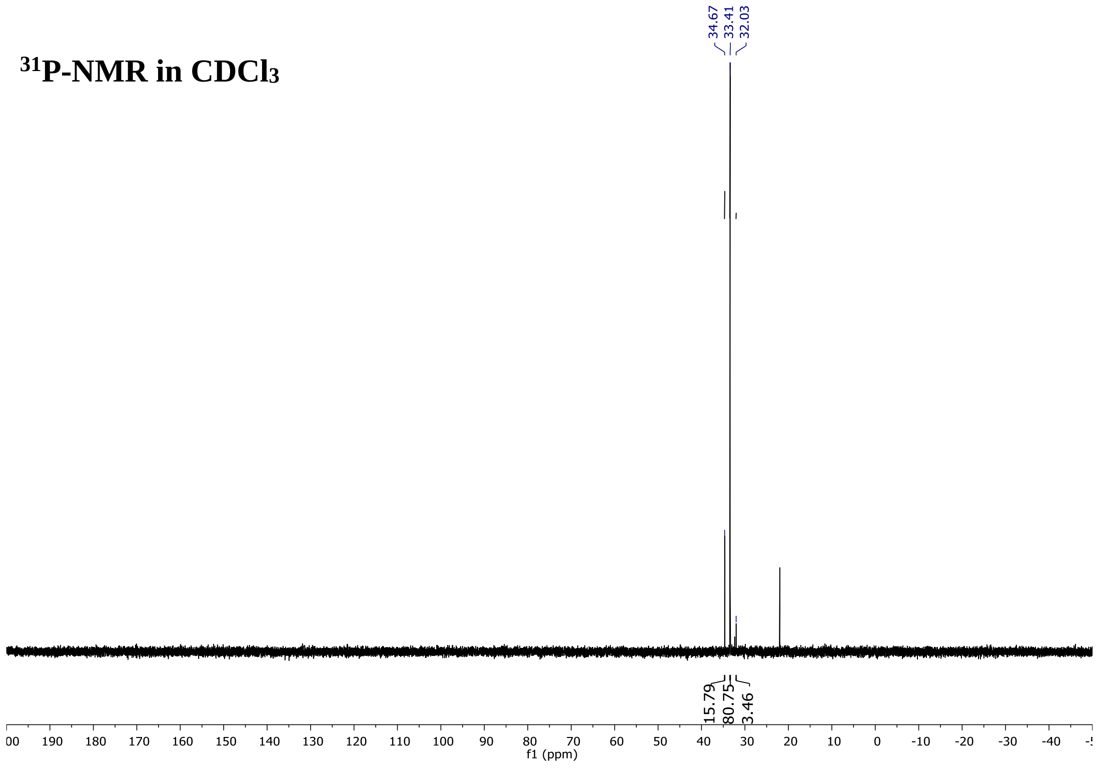

31P-NMR in CDCl3
34.67
33.41
32.03
15.79
80.75
3.46
f1 (ppm)

Similar results were obtained when examining the reactivity of alkenylphosphonium salt **II** (Figure S 7). Despite multiple purification attempts, complete removal of minor impurities was not possible and characterization data proved to be too complicated to unequivocal assign of the signals.

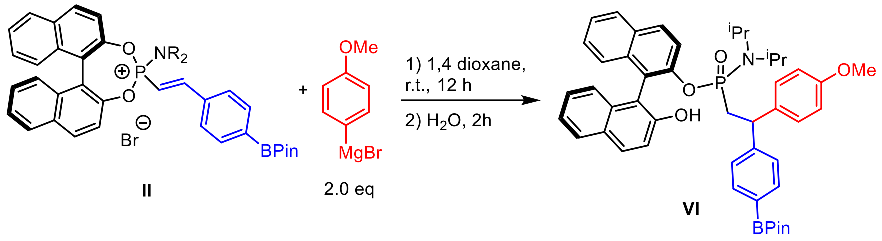

Figure S 7

^{1}H-NMR in CDCl3

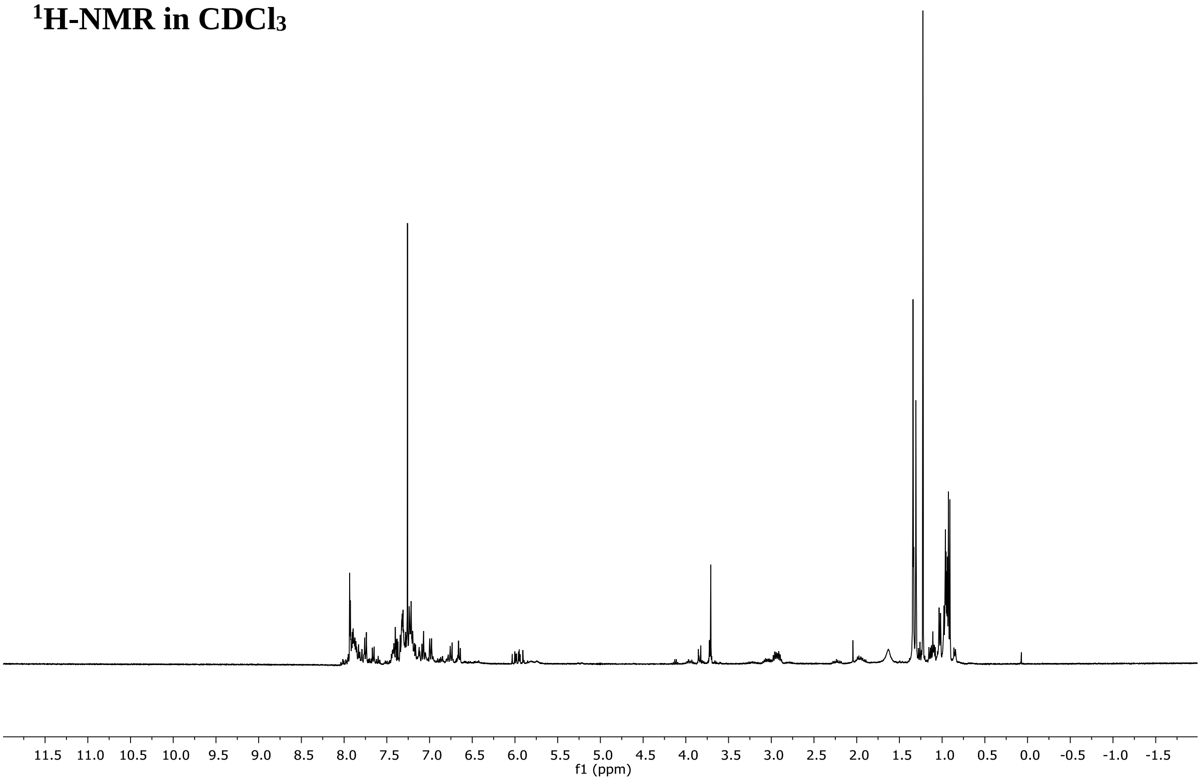

^{31}P-NMR in $CDCl_3$

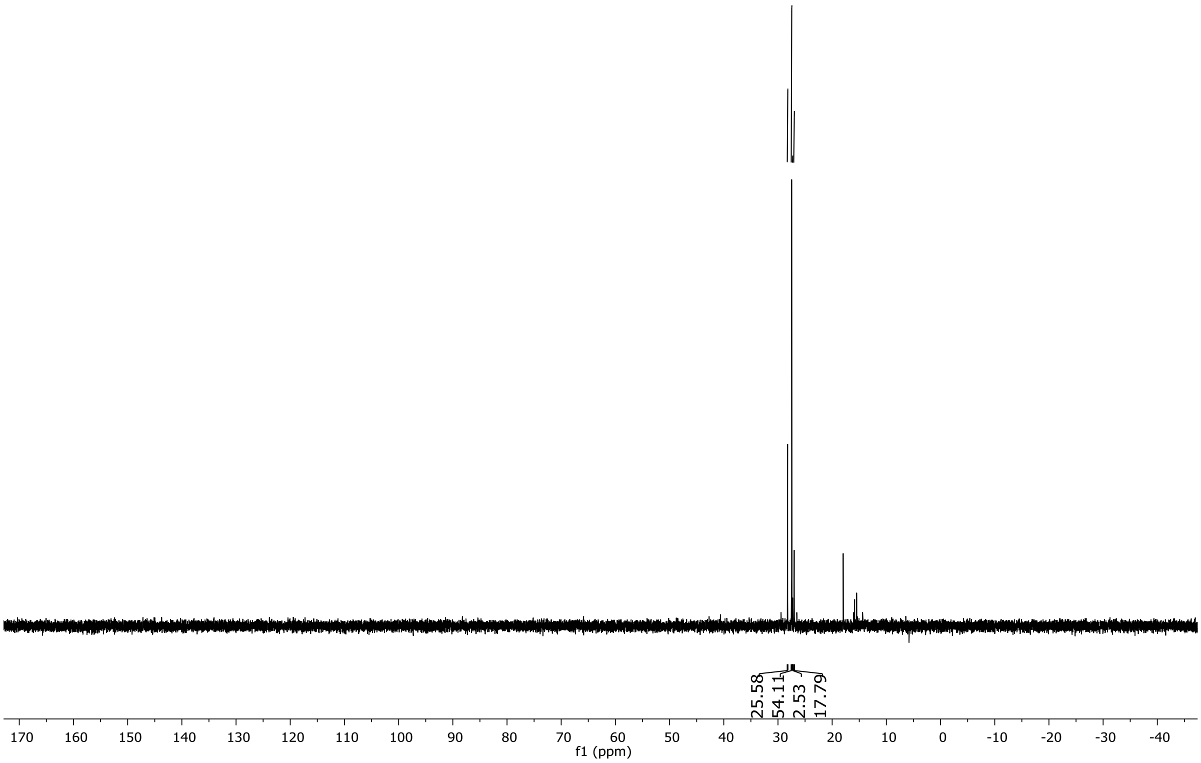

11. References

1. A. Duursma, J.-G. Boiteau, L. Lefort, J. A. F. Boogers, A. H. M. de Vries, J. G. de Vries, A. J. Minnaard and B. L. Feringa, *J. Org. Chem.*, 2004, **69**, 8045-8052.
2. R. Ardkhean, P. M. C. Roth, R. M. Maksymowicz, A. Curran, Q. Peng, R. S. Paton and S. P. Fletcher, *ACS Catal.*, 2017, **7**, 6729-6737.
3. X.-B. Chen, D. Padín, C. N. Stindt and B. L. Feringa, *Angew. Chem. Int. Ed.*, 2023, **62**, e202307450.
4. Y. Minko, M. Pasco, L. Lercher and I. Marek, *Nat. Protoc.*, 2013, **8**, 749-754.
5. APEX4: Bruker, APEX4, SAINT and SADABS. Bruker AXS Inc., Madison, Wisconsin, USA.
6. G. Sheldrick, *Acta Cryst. A*, 2015, **71**, 3-8.
7. O. V. Dolomanov, L. J. Bourhis, R. J. Gildea, J. A. K. Howard and H. Puschmann, *J. Appl. Crystallogr.*, 2009, **42**, 339-341.
8. L. Krause, R. Herbst-Irmer, G. M. Sheldrick and D. Stalke, *J. Appl. Crystallogr.*, 2015, **48**, 3-10.
9. A. Spek, *Acta Crystallogr., Sect. C: Struct. Chem.*, 2015, **71**, 9-18.
10. Frisch, M. J.; Trucks, G. W.; Schlegel, H. B.; Scuseria, G. E.; Robb, M. A.; Cheeseman, J. R.; Scalmani, G.; Barone, V.; Mennucci, B.; Petersson, G. A.; Nakatsuji, H.; Caricato, M.; Li, X.; Hratchian, H. P.; Izmaylov, A. F.; Bloino, J.; Zheng, G.; Sonnenberg, J. L.; Hada, M.; Ehara, M.; Toyota, K.; Fukuda, R.; Hasegawa, J.; Ishida, M.; Nakajima, T.; Honda, Y.; Kitao, O.; Nakai, H.; Vreven, T.; Montgomery, J. A., Jr.; Peralta, J. E.; Ogliaro, F.; Bearpark, M.; Heyd, J. J.; Brothers, E.; Kudin, K. N.; Staroverov, V. N.; Kobayashi, R.; Normand, J.; Raghavachari, K.; Rendell, A.; Burant, J. C.; Iyengar, S. S.; Tomasi, J.; Cossi, M.; Rega, N.; Millam, N. J.; Klene, M.; Knox, J. E.; Cross, J. B.; Bakken, V.; Adamo, C.; Jaramillo, J.; Gomperts, R.; Stratmann, R. E.; Yazyev, O.; Austin, A. J.; Cammi, R.; Pomelli, C.; Ochterski, J. W.; Martin, R. L.; Morokuma, K.; Zakrzewski, V. G.; Voth, G. A.; Salvador, P.; Dannenberg, J. J.; Dapprich, S.; Daniels, A. D.; Farkas, Ö.; Foresman, J. B.; Ortiz, J. V.; Cioslowski, J.; Fox, D. J. Gaussian 09, Revision D.01; Gaussian, Inc., Wallingford, CT, 2013.
11. J.-D. Chai and M. Head-Gordon, *J. Chem. Phys.*, 2008, **128**, 084106.
12. J.-D. Chai and M. Head-Gordon, *Phys. Chem. Chem. Phys.*, 2008, **10**, 6615-6620.
13. T. Clark, J. Chandrasekhar, G. W. Spitznagel and P. V. R. Schleyer, *J. Comput. Chem.*, 1983, **4**, 294-301.
14. M. J. Frisch, J. A. Pople and J. S. Binkley, *J. Chem. Phys.*, 1984, **80**, 3265-3269.
15. Legault, C. Y. CYLview, 1.0b; Université de Sherbrooke: Quebec, Canada, 2009; http://www.cylview.org.

12. NMR spectra

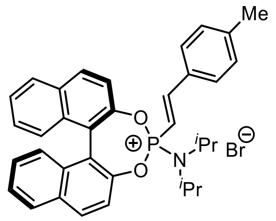

***R*-3b**

^{1}H-NMR

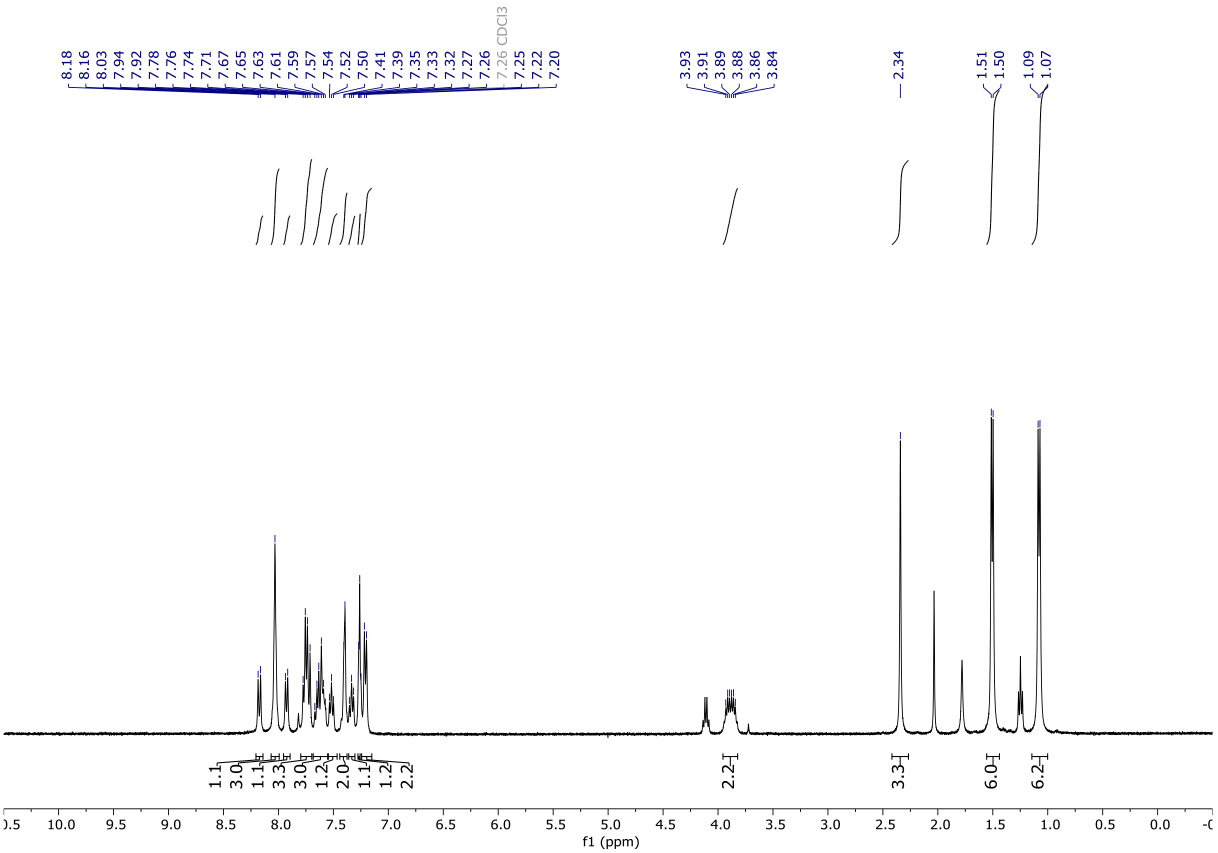

8.18
8.16
8.03
7.94
7.92
7.78
7.76
7.74
7.71
7.67
7.65
7.63
7.61
7.59
7.57
7.54
7.52
7.50
7.41
7.39
7.35
7.33
7.32
7.27
7.26 CDCl3
7.25
7.22
7.20
3.93
3.91
3.89
3.88
3.86
3.84
2.34
1.51
1.50
1.09
1.07
1.1
3.0
1.1
3.3
3.0
1.2
2.0
1.1
1.2
2.2
2.2
3.3
6.0
6.2
f1 (ppm)

^{31}P-NMR

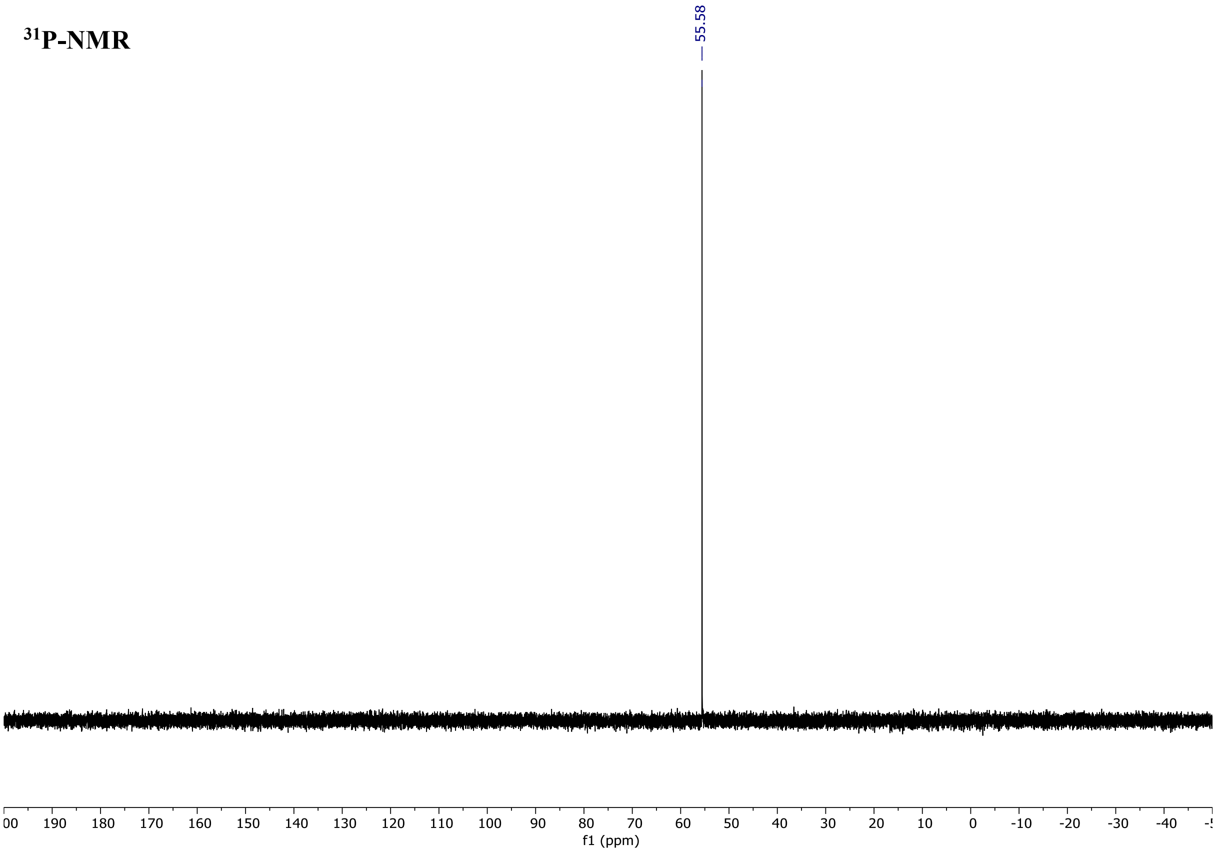

^{13}C-NMR

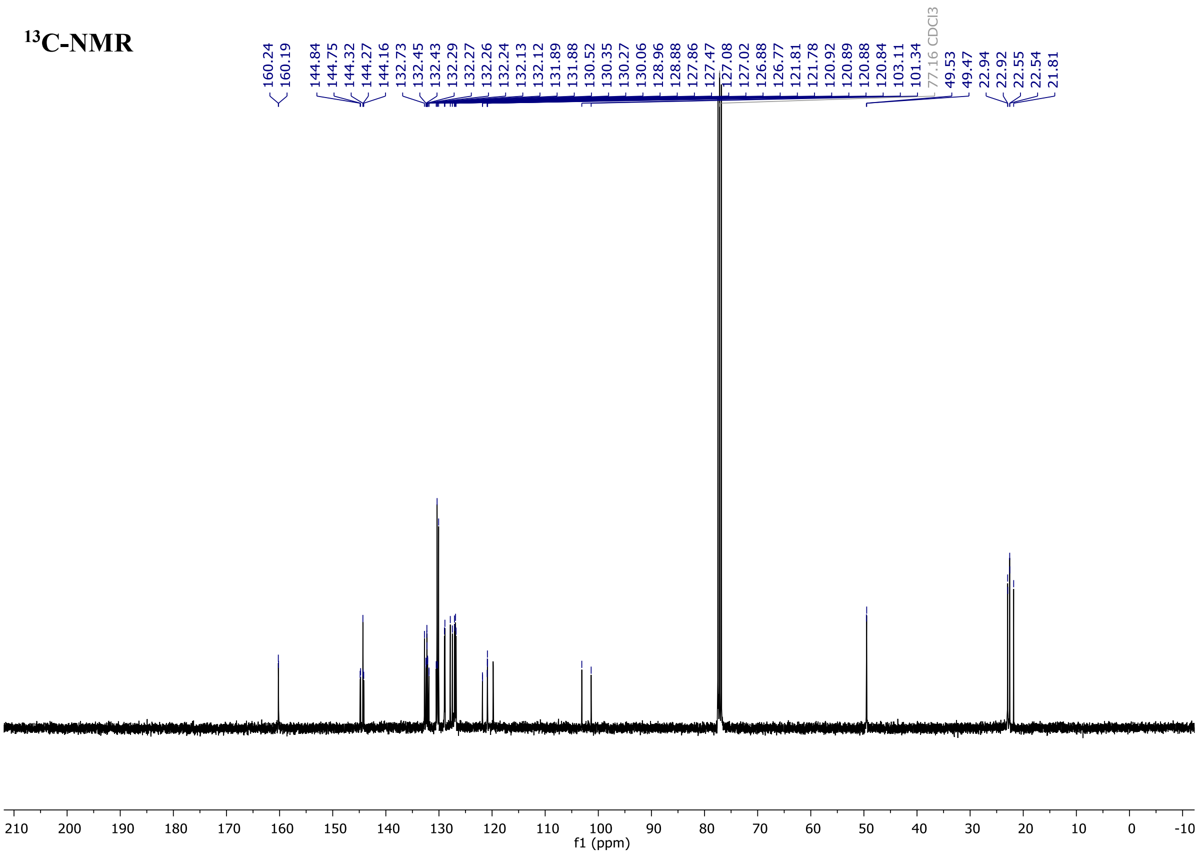

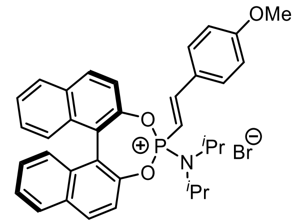
OMe
O
P
O
N
iPr
iPr
Br

***R*-3c**

^{1}H-NMR

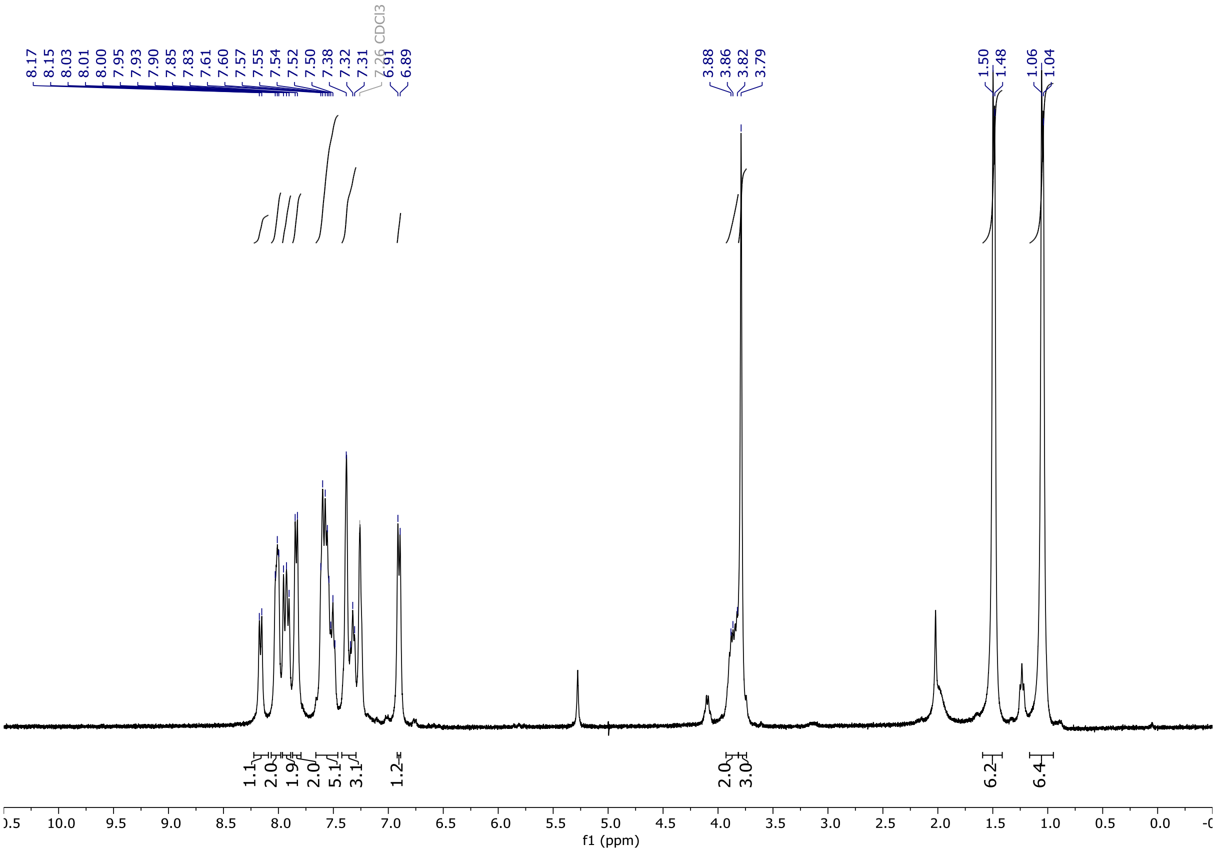
8.17
8.15
8.03
8.01
8.00
7.95
7.93
7.90
7.85
7.83
7.61
7.60
7.57
7.55
7.54
7.52
7.50
7.38
7.32
7.31
7.26 CDCl3
6.91
6.89
3.88
3.86
3.82
3.79
1.50
1.48
1.06
1.04
1.1
2.0
1.9
2.0
5.1
3.1
1.2
2.0
3.0
6.2
6.4
10.0
9.5
9.0
8.5
8.0
7.5
7.0
6.5
6.0
5.5
5.0
4.5
4.0
3.5
3.0
2.5
2.0
1.5
1.0
0.5
0.0
f1 (ppm)

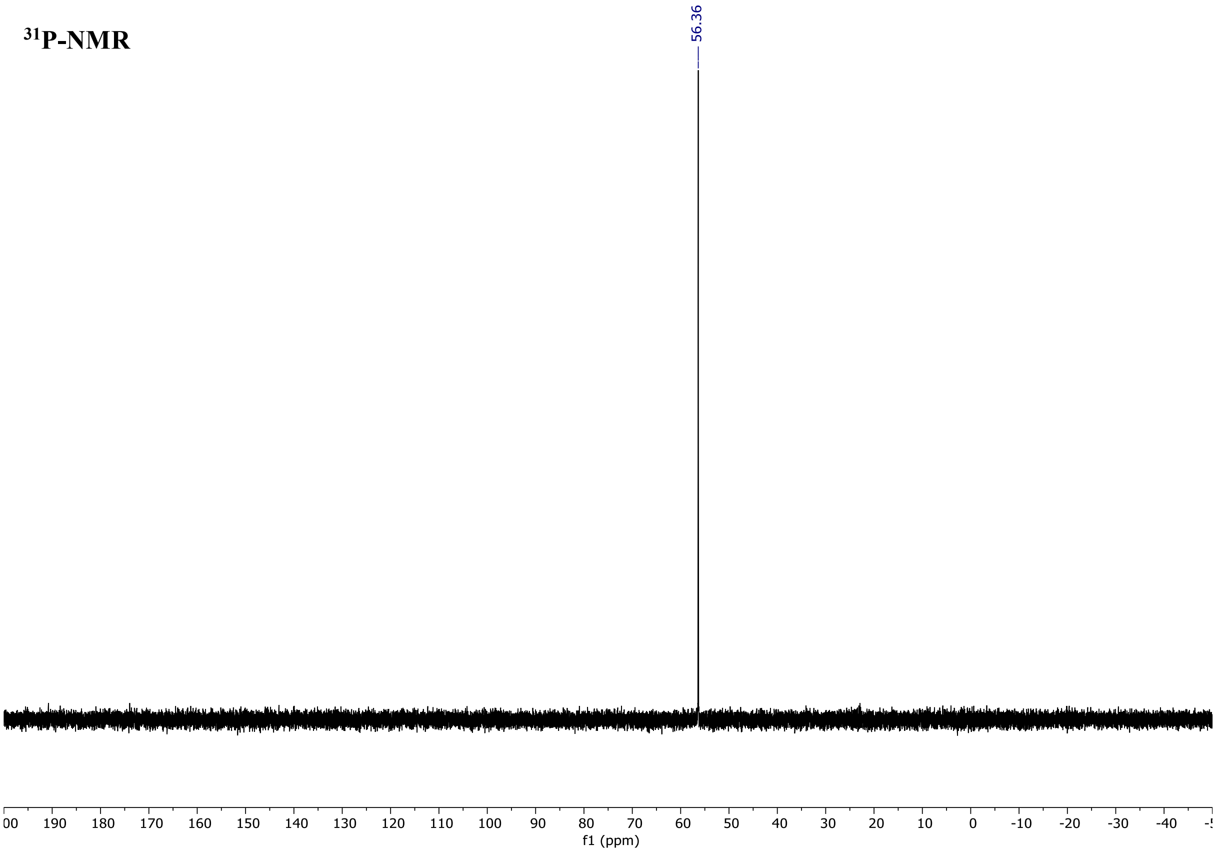

31P-NMR
56.36
f1 (ppm)

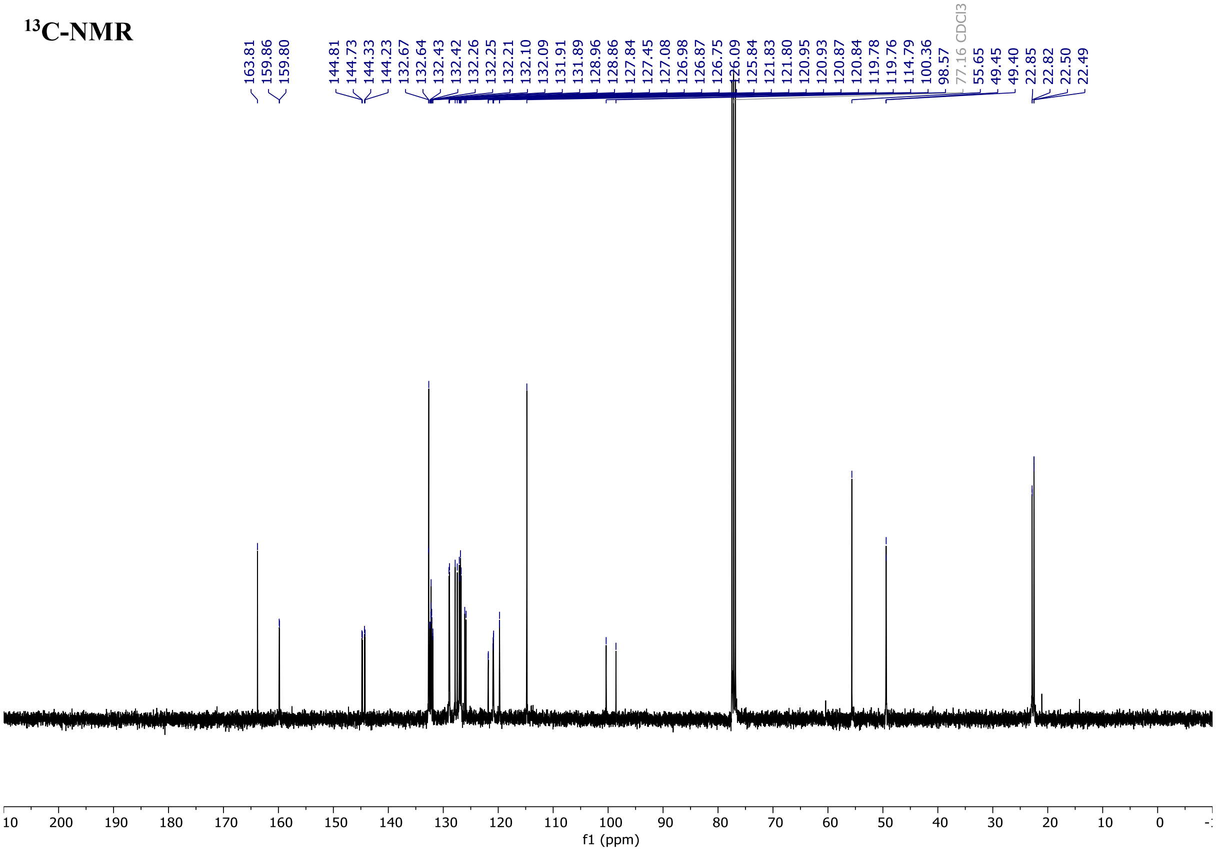

13C-NMR
163.81
159.86
159.80
144.81
144.73
144.33
144.23
132.67
132.64
132.43
132.42
132.26
132.25
132.21
132.10
132.09
131.91
131.89
128.96
128.86
127.84
127.45
127.08
126.98
126.87
126.75
126.09
125.84
121.83
121.80
120.95
120.93
120.87
120.84
119.78
119.76
114.79
100.36
98.57
77.16 CDCl3
55.65
49.45
49.40
22.85
22.82
22.50
22.49
f1 (ppm)

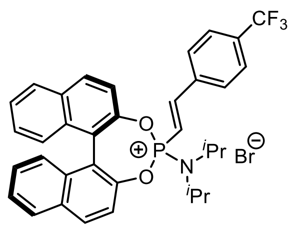
CF3
O
P
O
N
iPr
iPr
Br

***R*-3d**

^{1}H-NMR

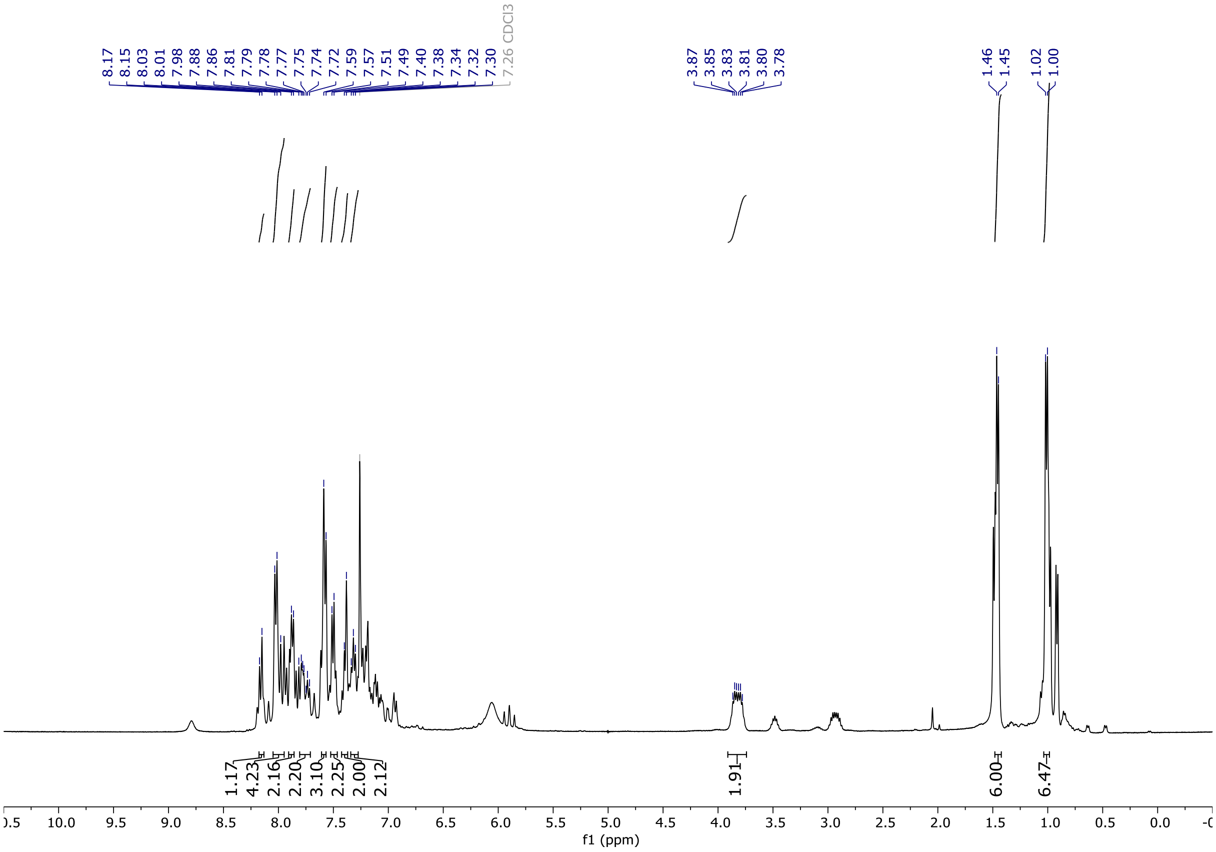
8.17
8.15
8.03
8.01
7.98
7.88
7.86
7.81
7.79
7.78
7.77
7.75
7.74
7.72
7.59
7.57
7.51
7.49
7.40
7.38
7.34
7.32
7.30
7.26 CDCl3
3.87
3.85
3.83
3.81
3.80
3.78
1.46
1.45
1.02
1.00
1.17
4.23
2.16
2.20
3.10
2.25
2.00
2.12
1.91
6.00
6.47
f1 (ppm)

^{31}P-NMR

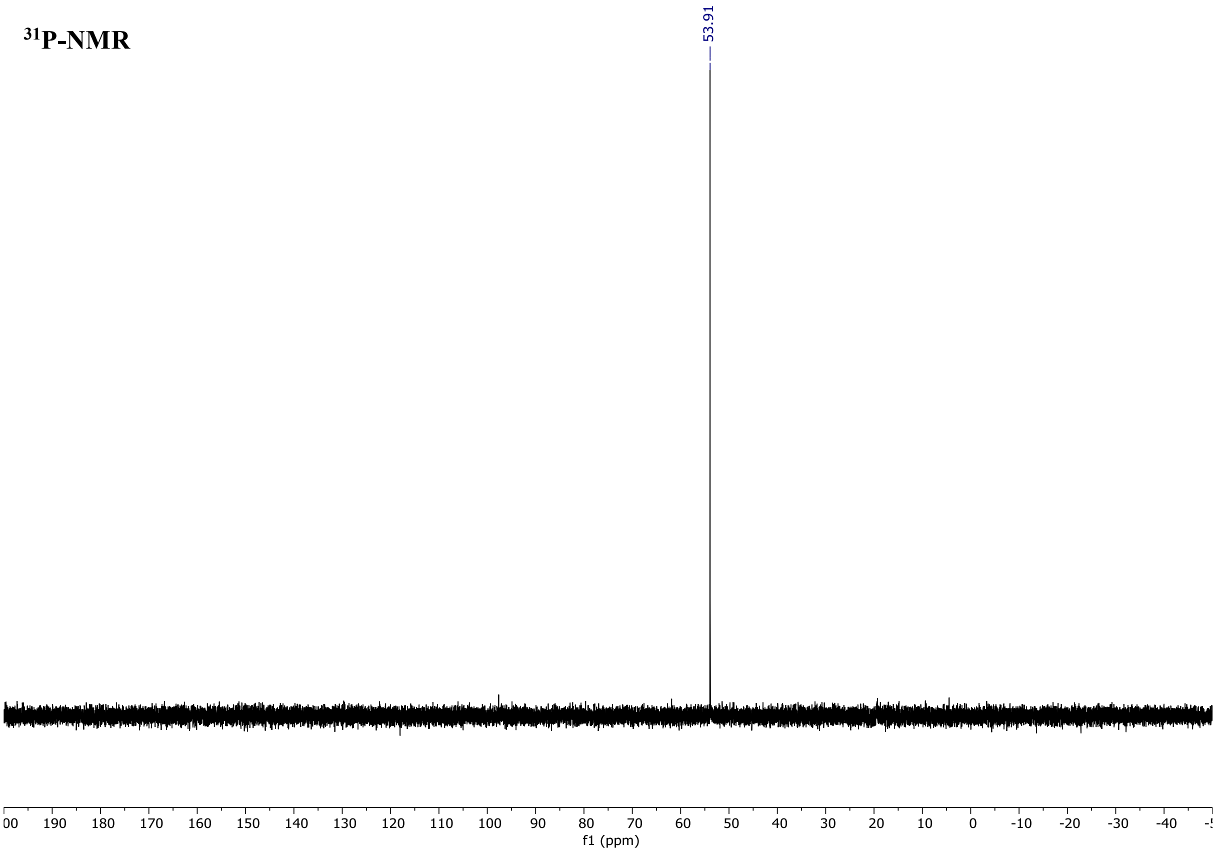

^{19}F-NMR

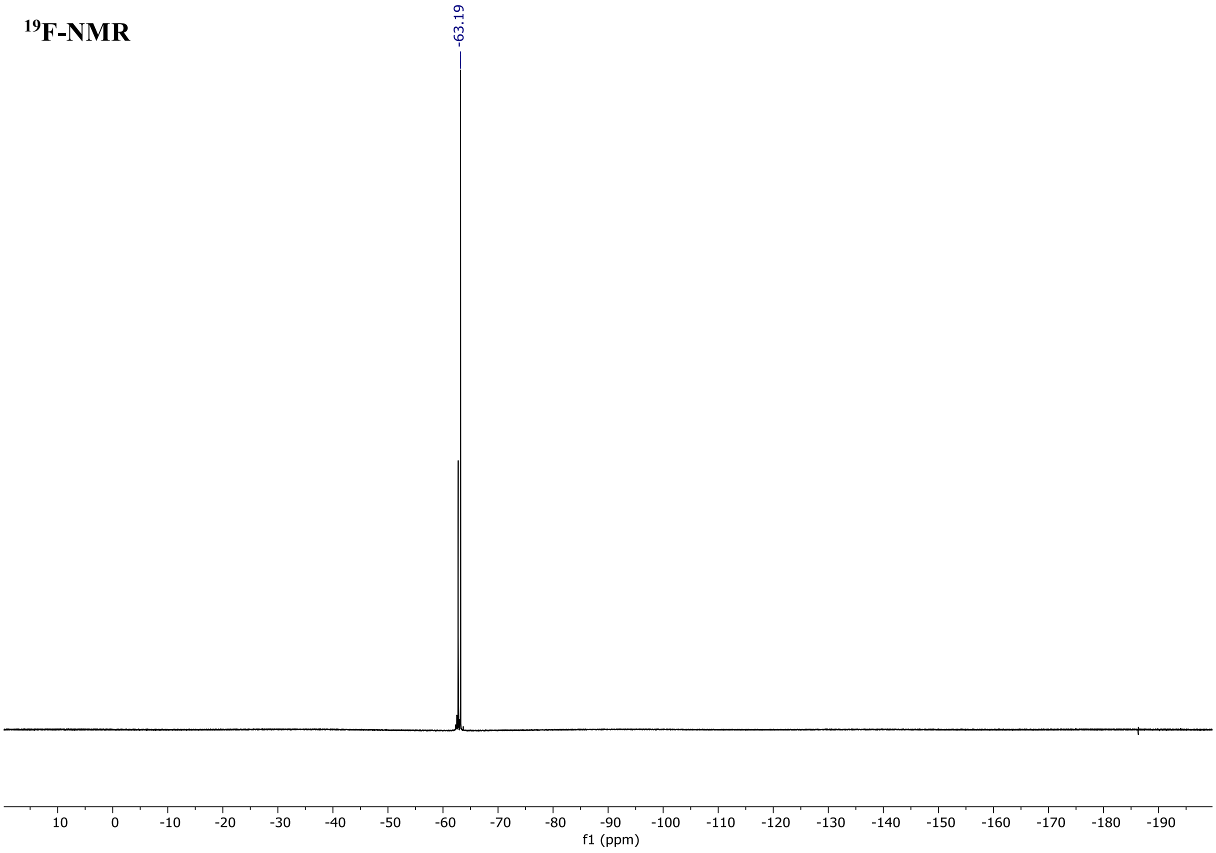

^{13}C-NMR

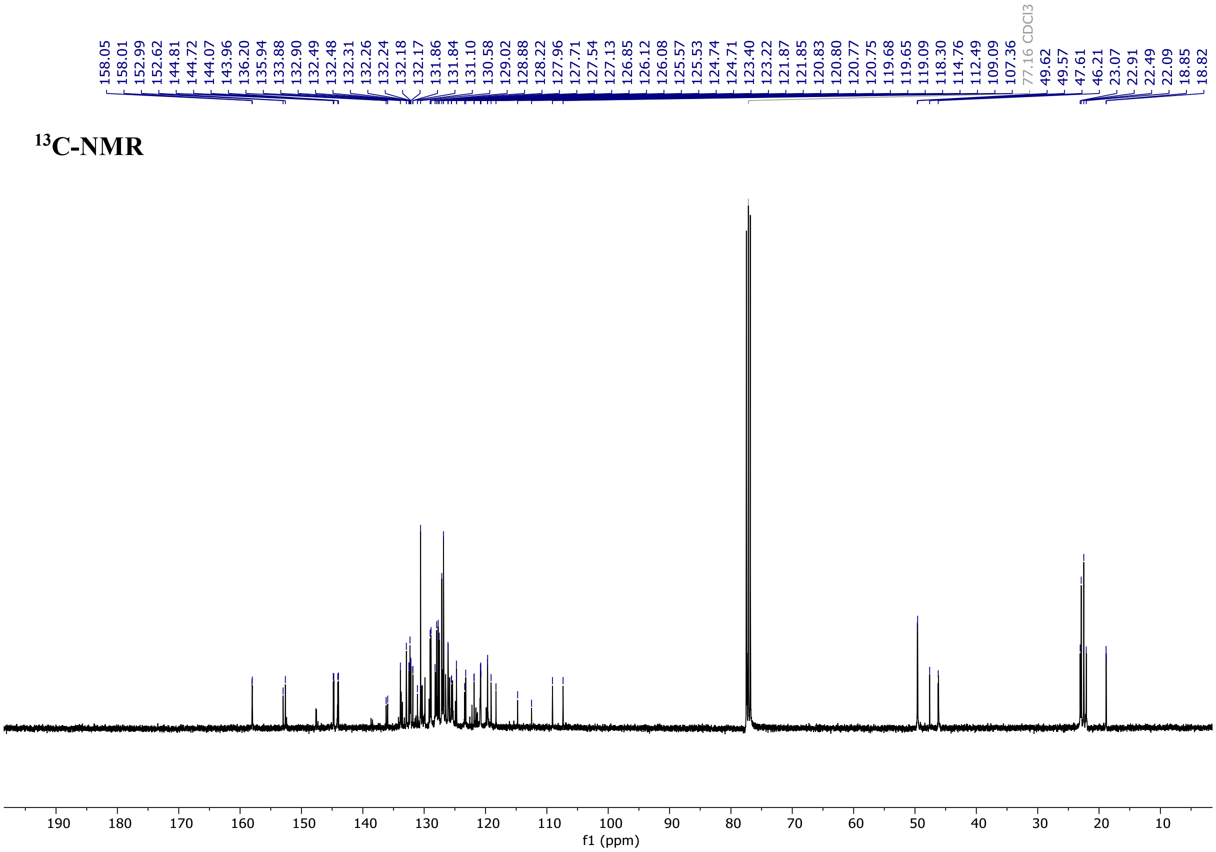
158.05
158.01
152.99
152.62
144.81
144.72
144.07
143.96
136.20
135.94
133.88
132.90
132.49
132.48
132.31
132.26
132.24
132.18
132.17
131.86
131.84
131.10
130.58
129.02
128.88
128.22
127.96
127.71
127.54
127.13
126.85
126.12
126.08
125.57
125.53
124.74
124.71
123.40
123.22
121.87
121.85
120.83
120.80
120.77
120.75
119.68
119.65
119.09
118.30
114.76
112.49
109.09
107.36
77.16 CDCl3
49.62
49.57
47.61
46.21
23.07
22.91
22.49
22.09
18.85
18.82
f1 (ppm)

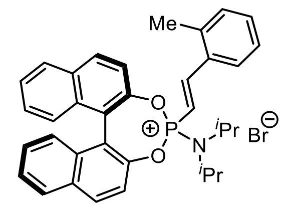
Me
O
P
O
N
iPr
iPr
Br

***R*-3e**

^{1}H-NMR

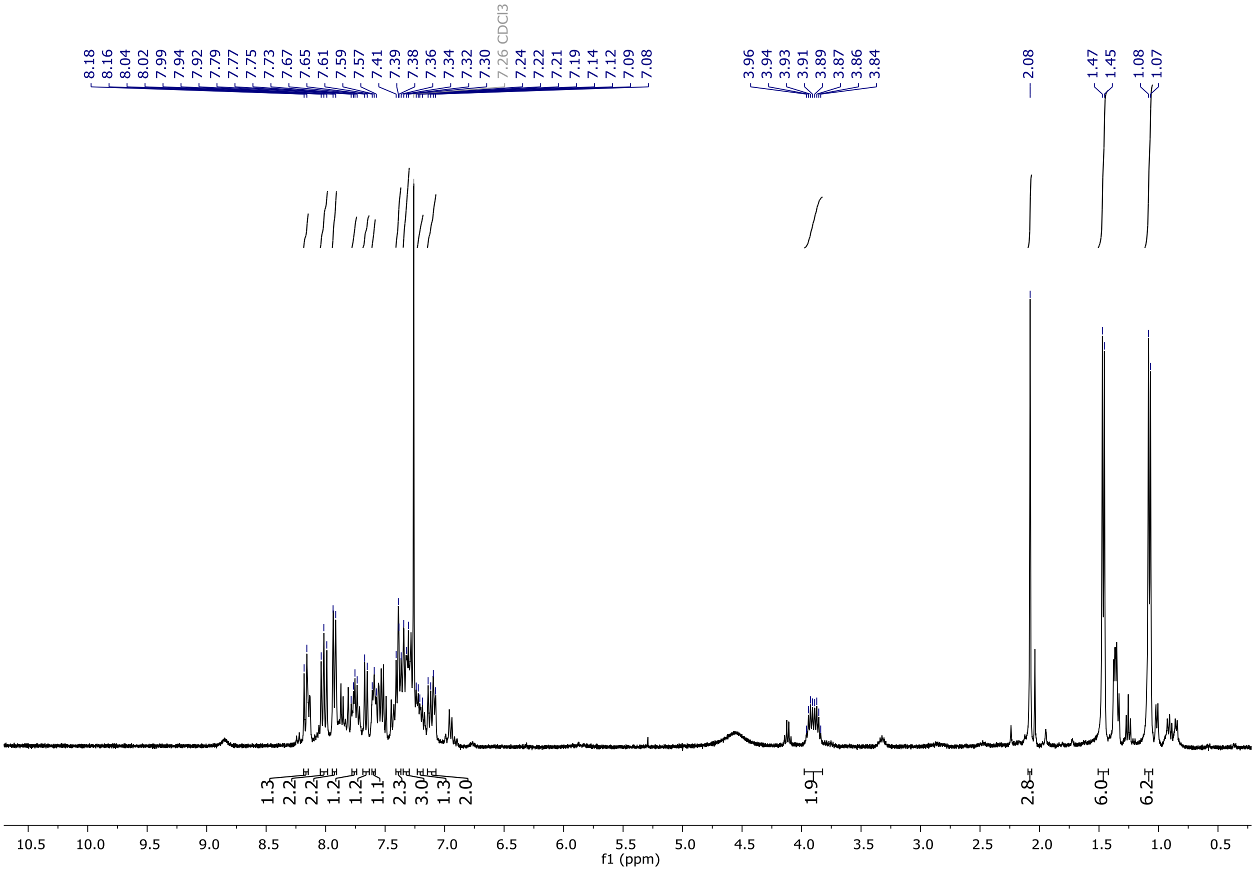
8.18
8.16
8.04
8.02
7.99
7.94
7.92
7.79
7.77
7.75
7.73
7.67
7.65
7.61
7.59
7.57
7.41
7.39
7.38
7.36
7.34
7.32
7.30
7.26 CDCl3
7.24
7.22
7.21
7.19
7.14
7.12
7.09
7.08
3.96
3.94
3.93
3.91
3.89
3.87
3.86
3.84
2.08
1.47
1.45
1.08
1.07
1.3
2.2
2.2
1.2
1.2
1.1
2.3
3.0
1.3
2.0
1.9
2.8
6.0
6.2
10.5
10.0
9.5
9.0
8.5
8.0
7.5
7.0
6.5
6.0
5.5
5.0
4.5
4.0
3.5
3.0
2.5
2.0
1.5
1.0
0.5
f1 (ppm)

^{31}P-NMR

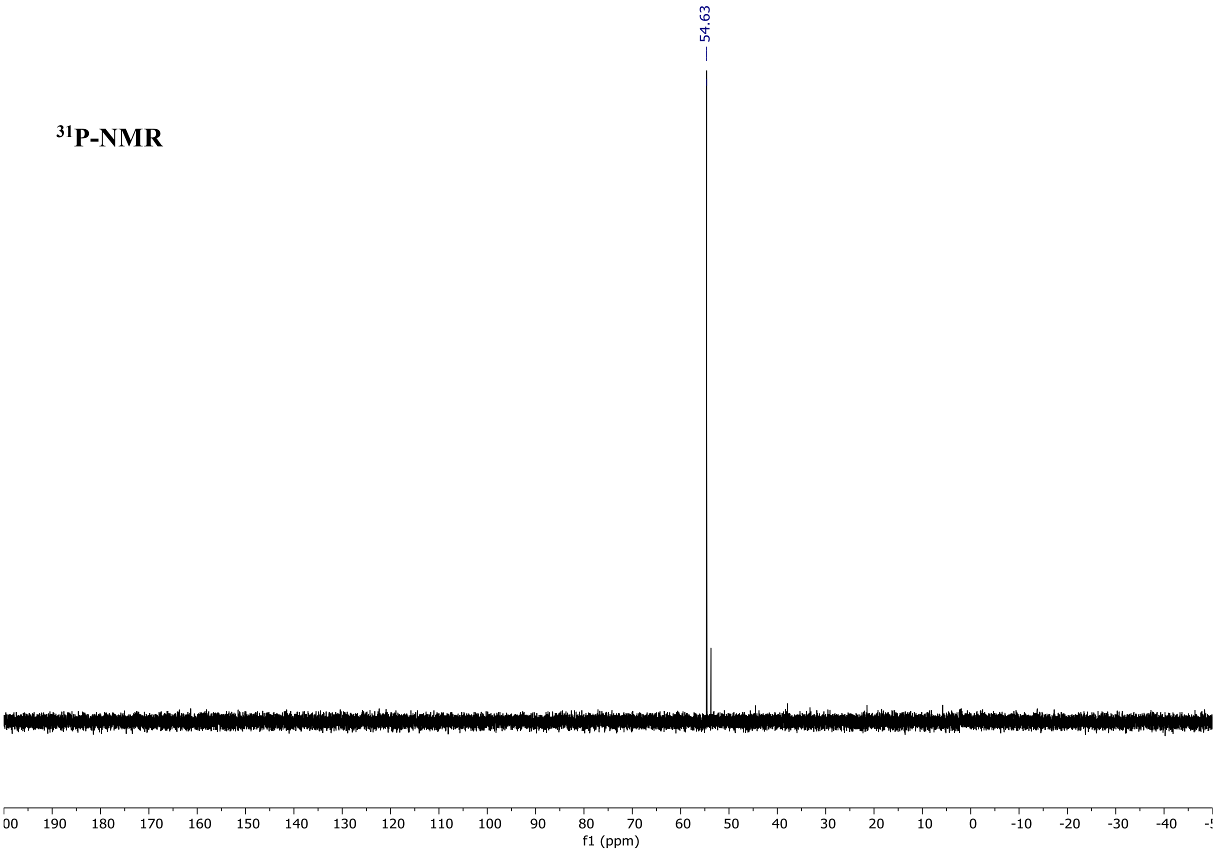

^{13}C-NMR

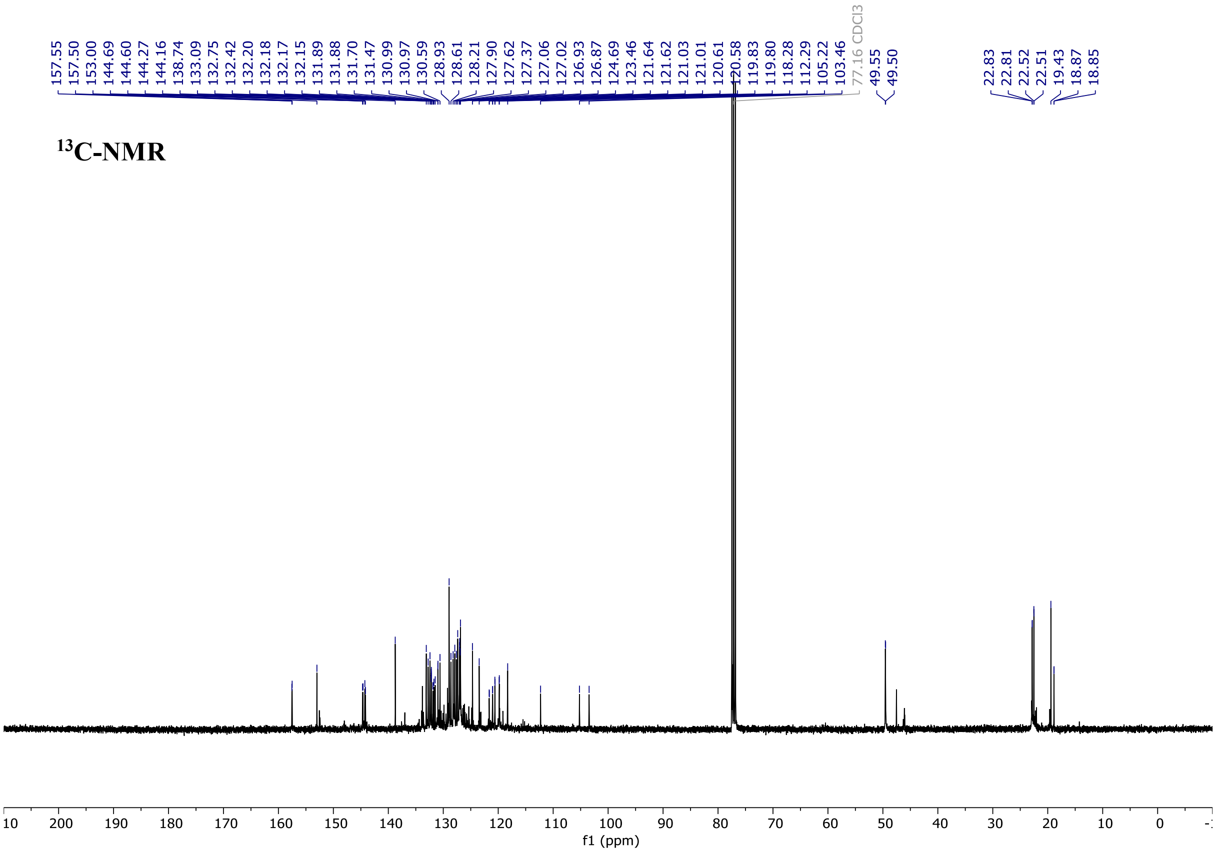

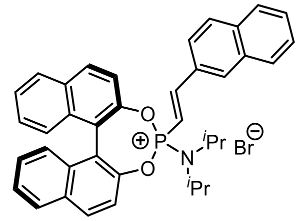

O
P
N
iPr
Br
iPr
O

***R*-3f**

^{1}H-NMR

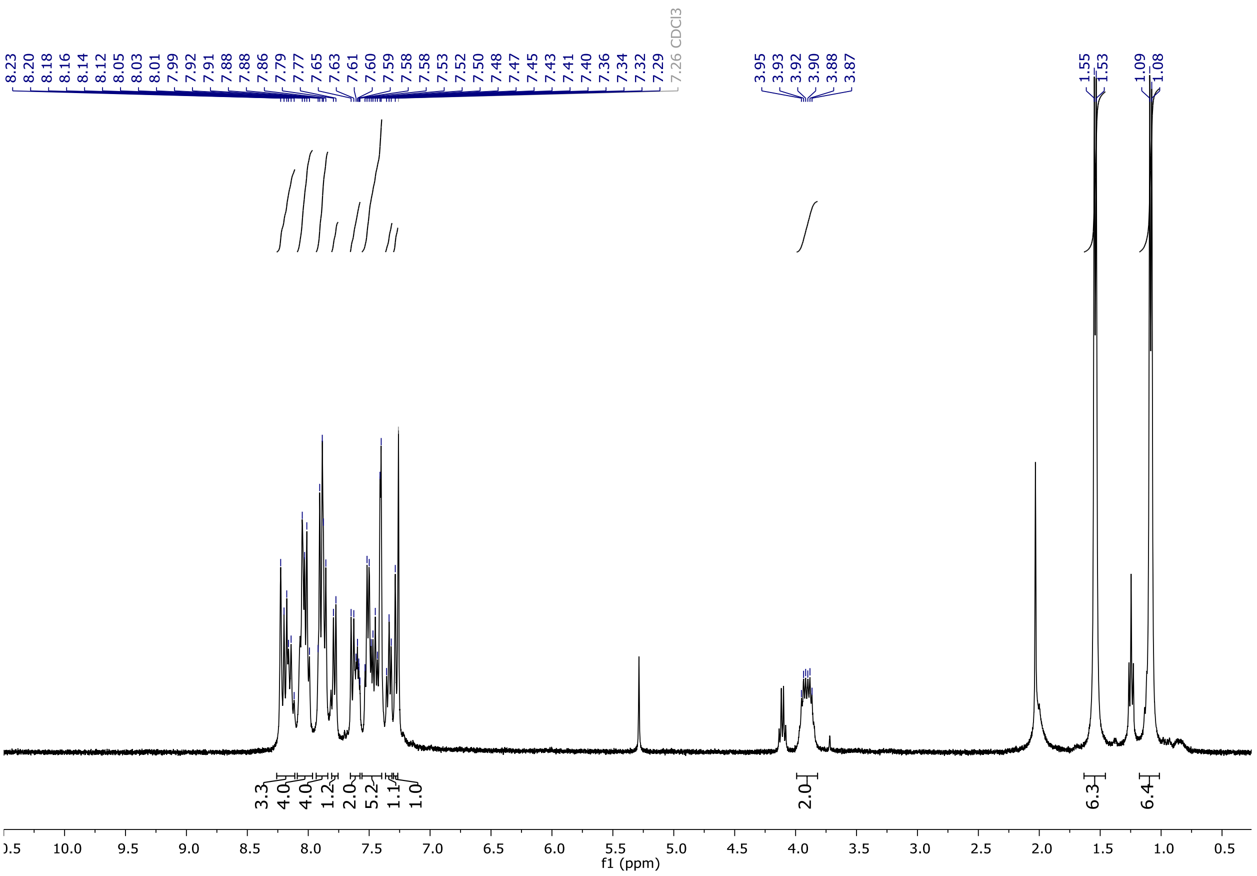

8.23
8.20
8.18
8.16
8.14
8.12
8.05
8.03
8.01
7.99
7.92
7.91
7.88
7.86
7.79
7.77
7.65
7.63
7.61
7.60
7.59
7.58
7.58
7.53
7.52
7.50
7.48
7.47
7.45
7.43
7.41
7.40
7.36
7.34
7.32
7.29
7.26 CDCl3
3.95
3.93
3.92
3.90
3.88
3.87
1.55
1.53
1.09
1.08
3.3
4.0
4.0
1.2
2.0
5.2
1.1
1.0
2.0
6.3
6.4
f1 (ppm)

^{31}P-NMR

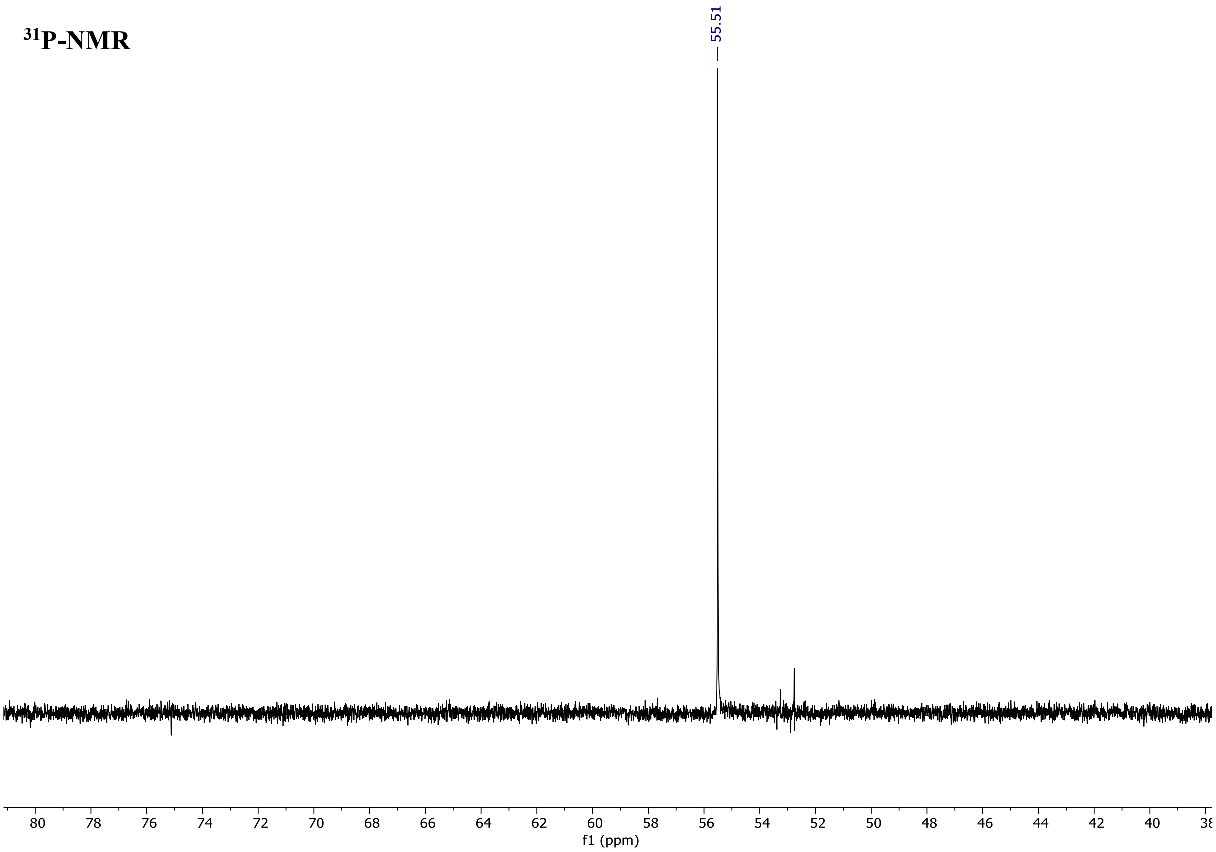

^{13}C-NMR

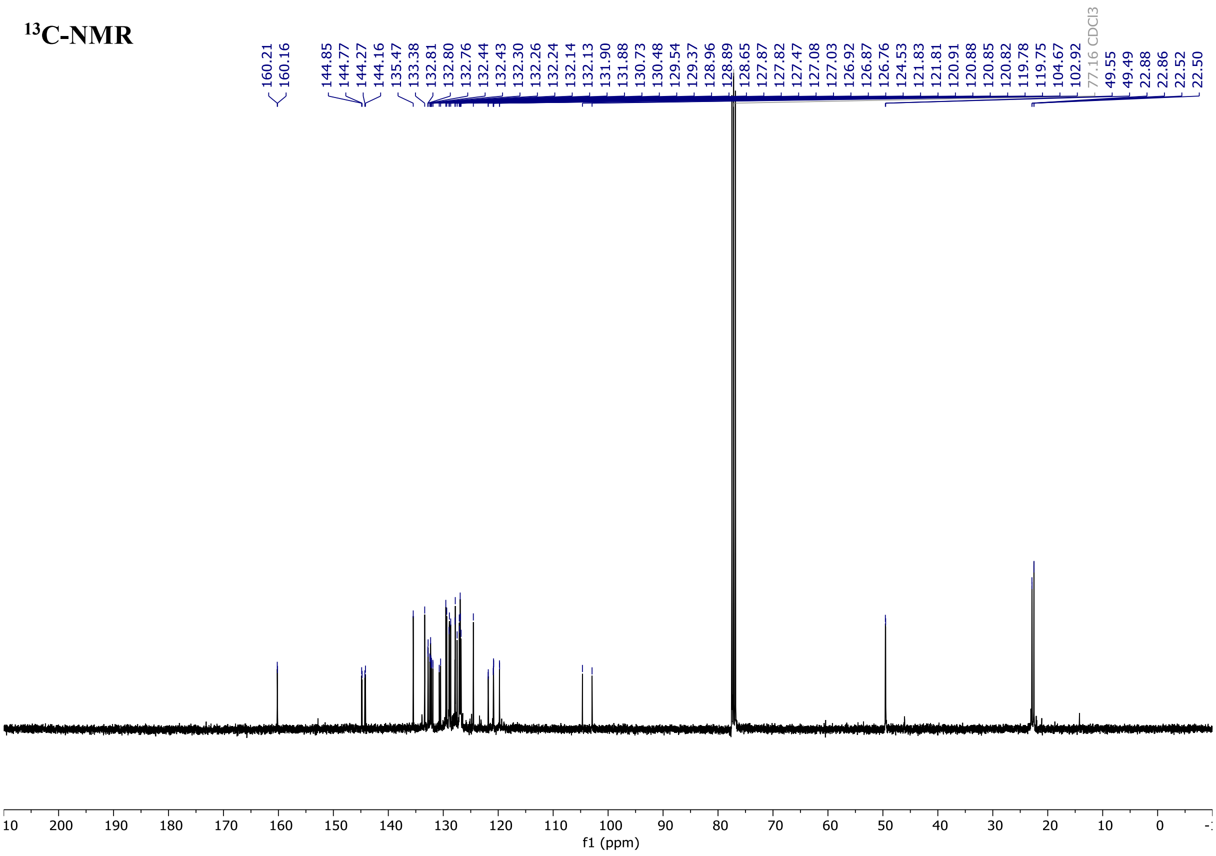

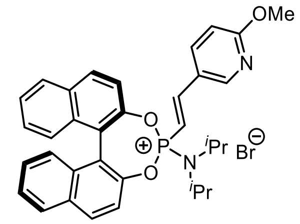

***R*-3g**

^{1}H-NMR

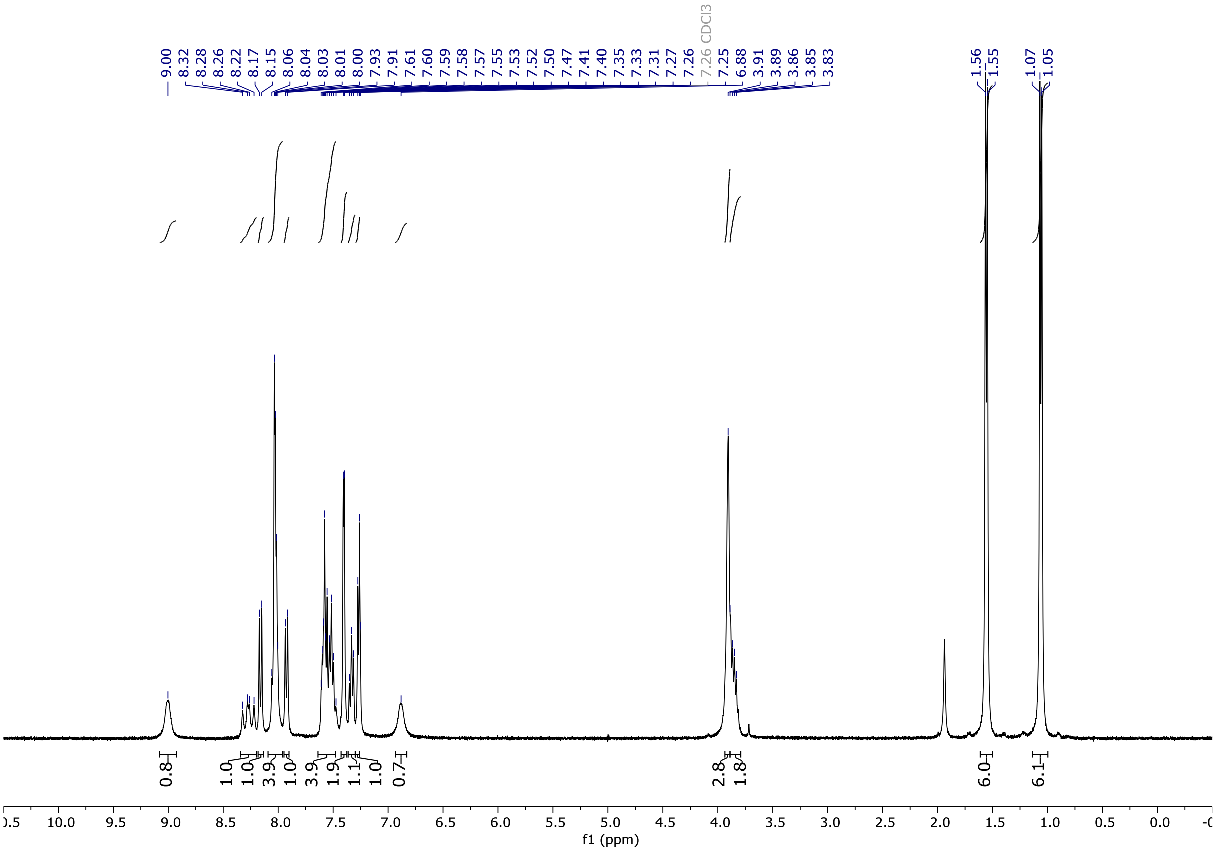

9.00
8.32
8.28
8.26
8.22
8.17
8.15
8.06
8.04
8.03
8.01
8.00
7.93
7.91
7.61
7.60
7.59
7.58
7.57
7.55
7.53
7.52
7.50
7.47
7.41
7.40
7.35
7.33
7.31
7.27
7.26
7.26 CDCl3
7.25
6.88
3.91
3.89
3.86
3.85
3.83
1.56
1.55
1.07
1.05
0.8
1.0
1.0
3.9
1.0
3.9
1.9
1.1
1.0
0.7
2.8
1.8
6.0
6.1
f1 (ppm)

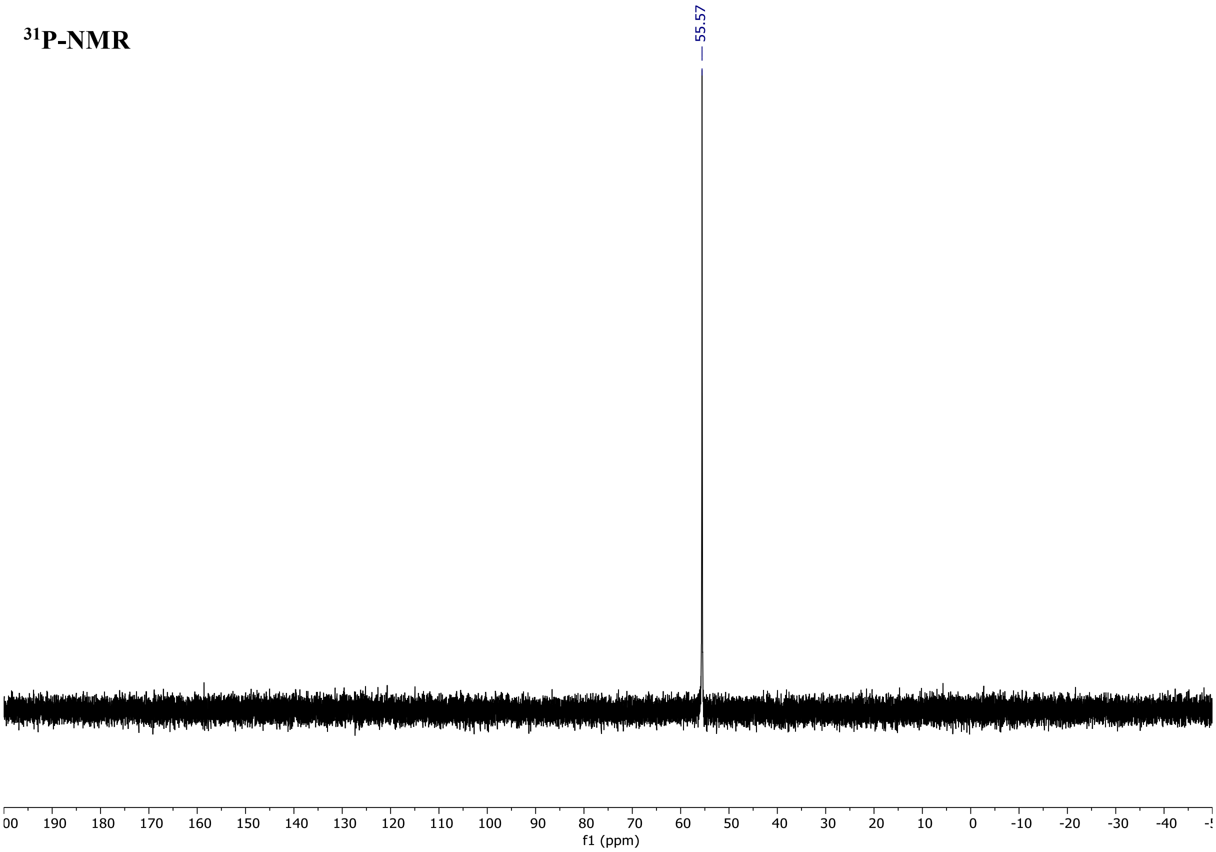
31P-NMR
55.57
f1 (ppm)

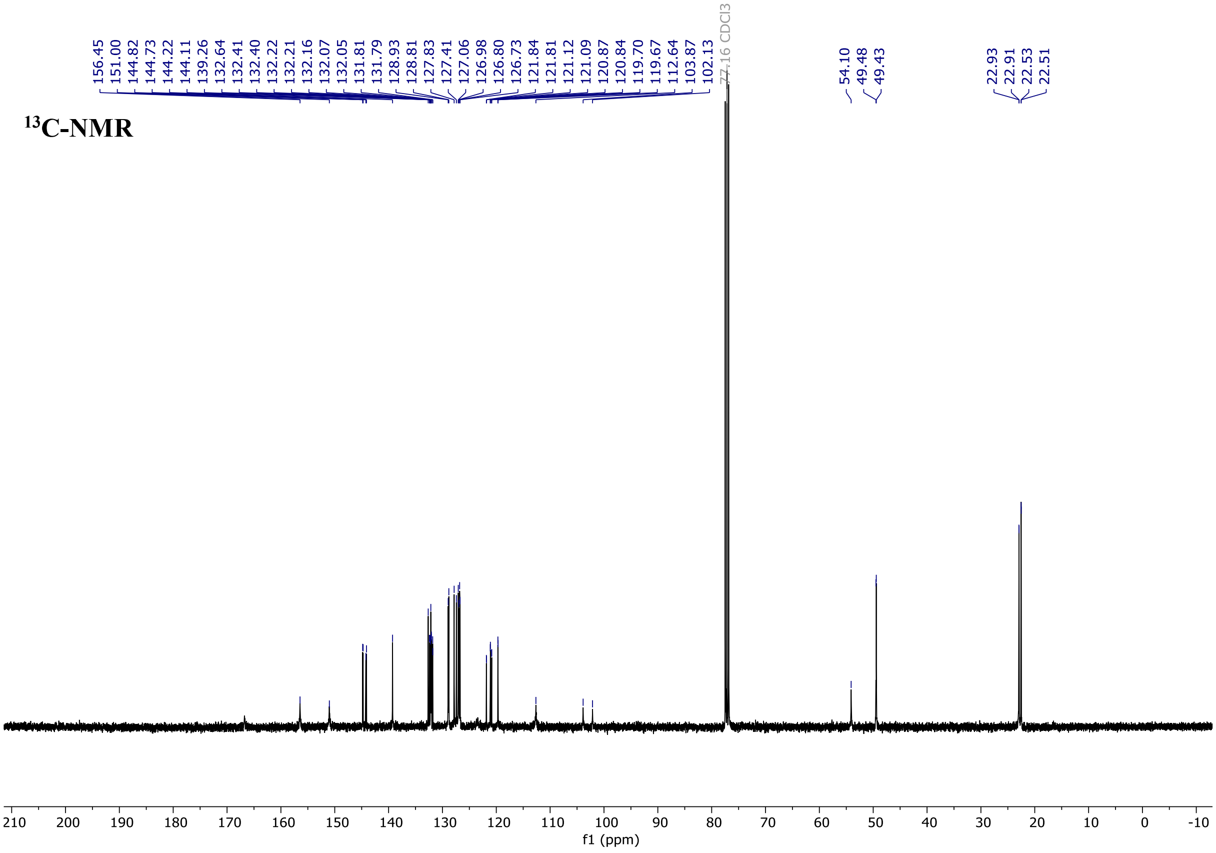
13C-NMR
156.45
151.00
144.82
144.73
144.22
144.11
139.26
132.64
132.41
132.40
132.22
132.21
132.16
132.07
132.05
131.81
131.79
128.93
128.81
127.83
127.41
127.06
126.98
126.80
126.73
121.84
121.81
121.12
121.09
120.87
120.84
119.70
119.67
112.64
103.87
102.13
77.16 CDCl3
54.10
49.48
49.43
22.93
22.91
22.53
22.51
f1 (ppm)

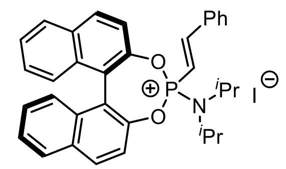

***R*-3a-I**

^{1}H-NMR

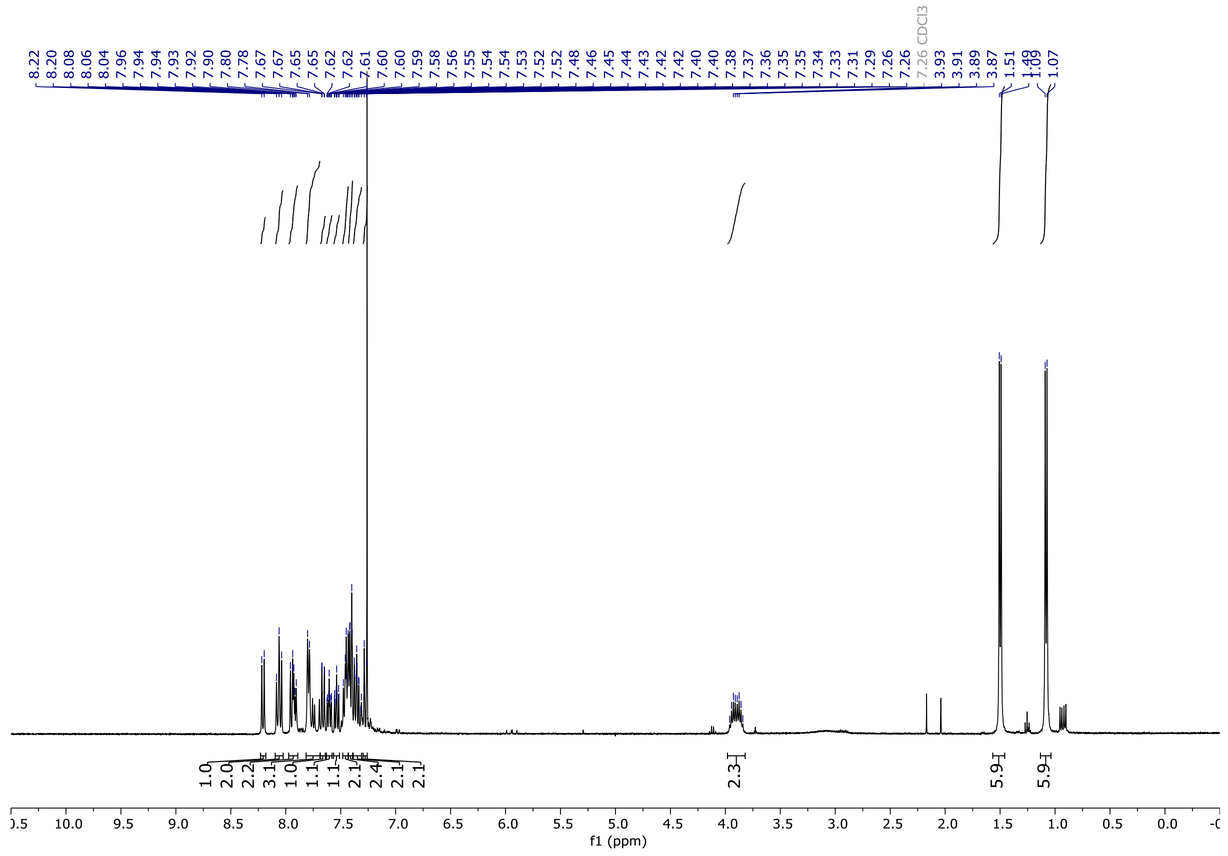

^{31}P-NMR

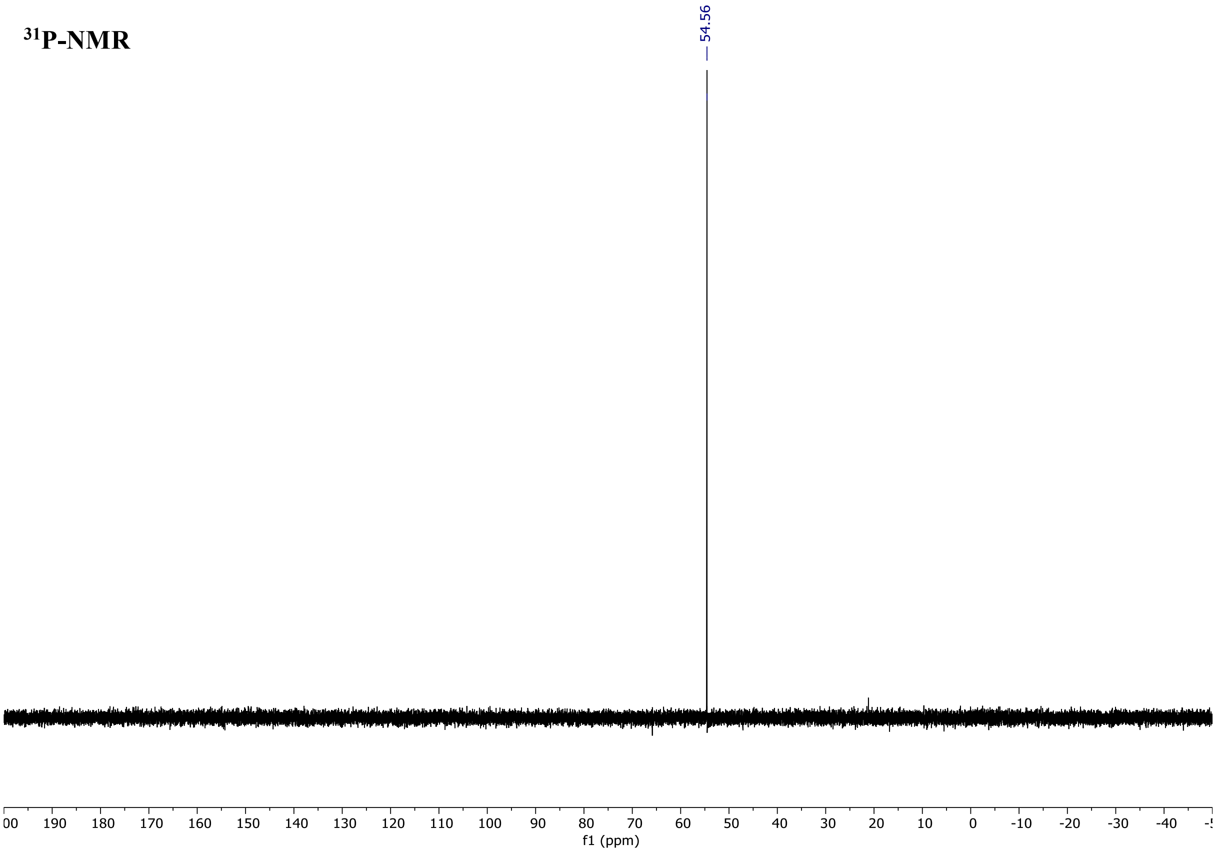

^{13}C-NMR

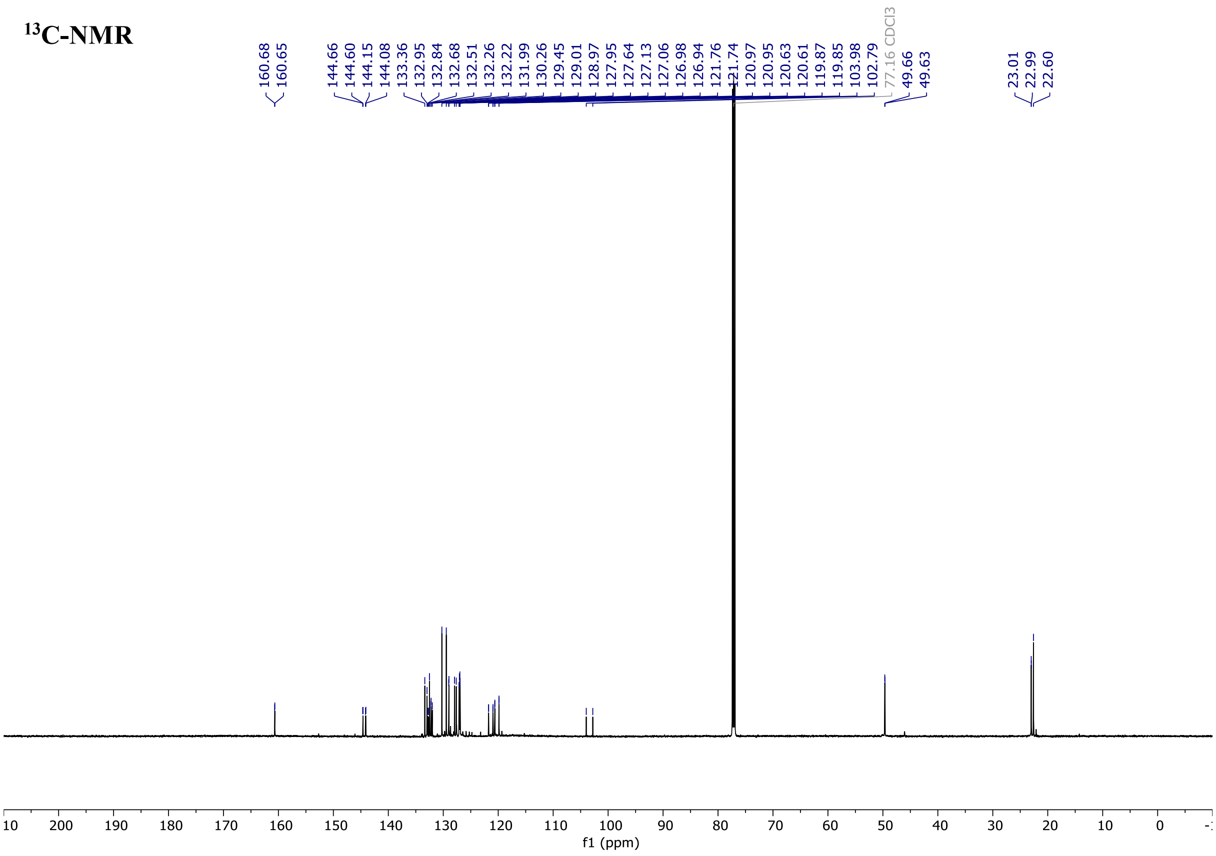

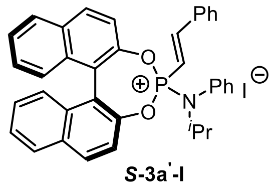

***S*-3a'-I**

^{1}H-NMR

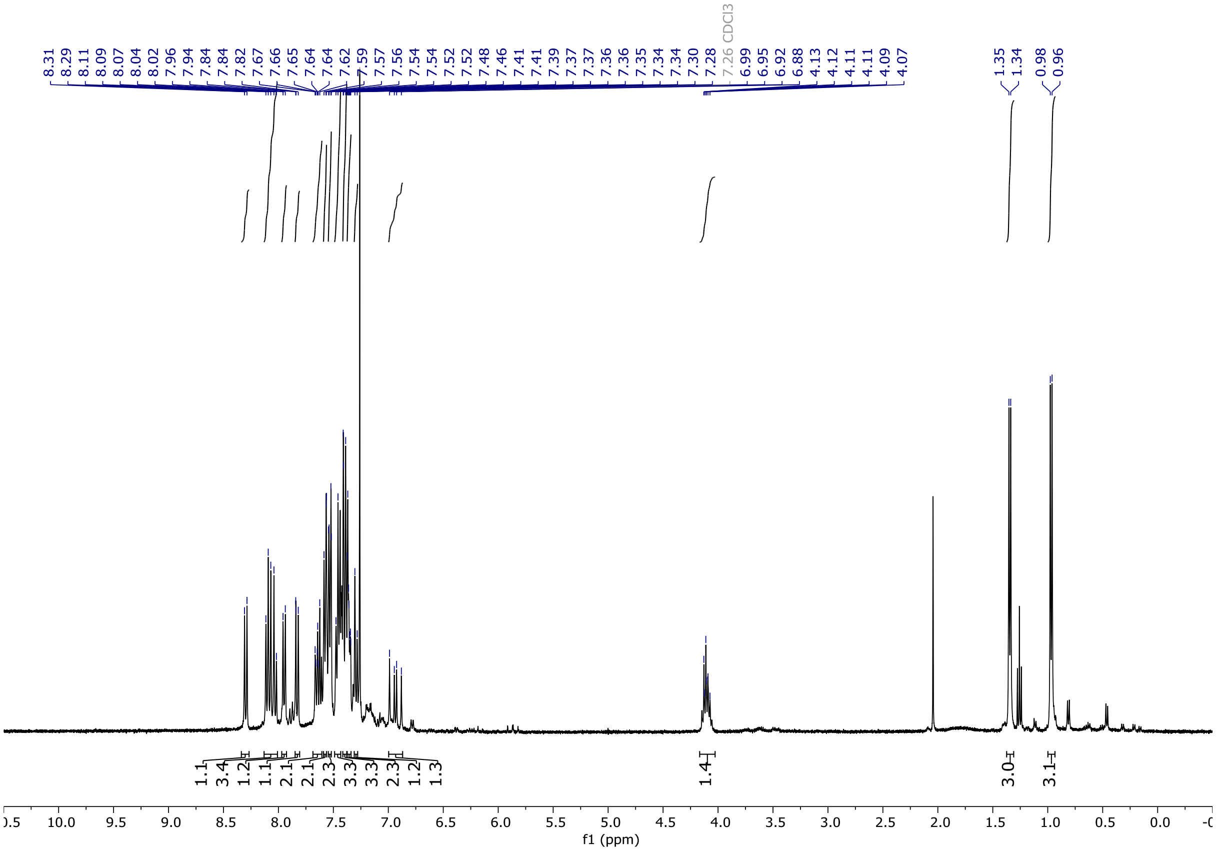

^{31}P-NMR

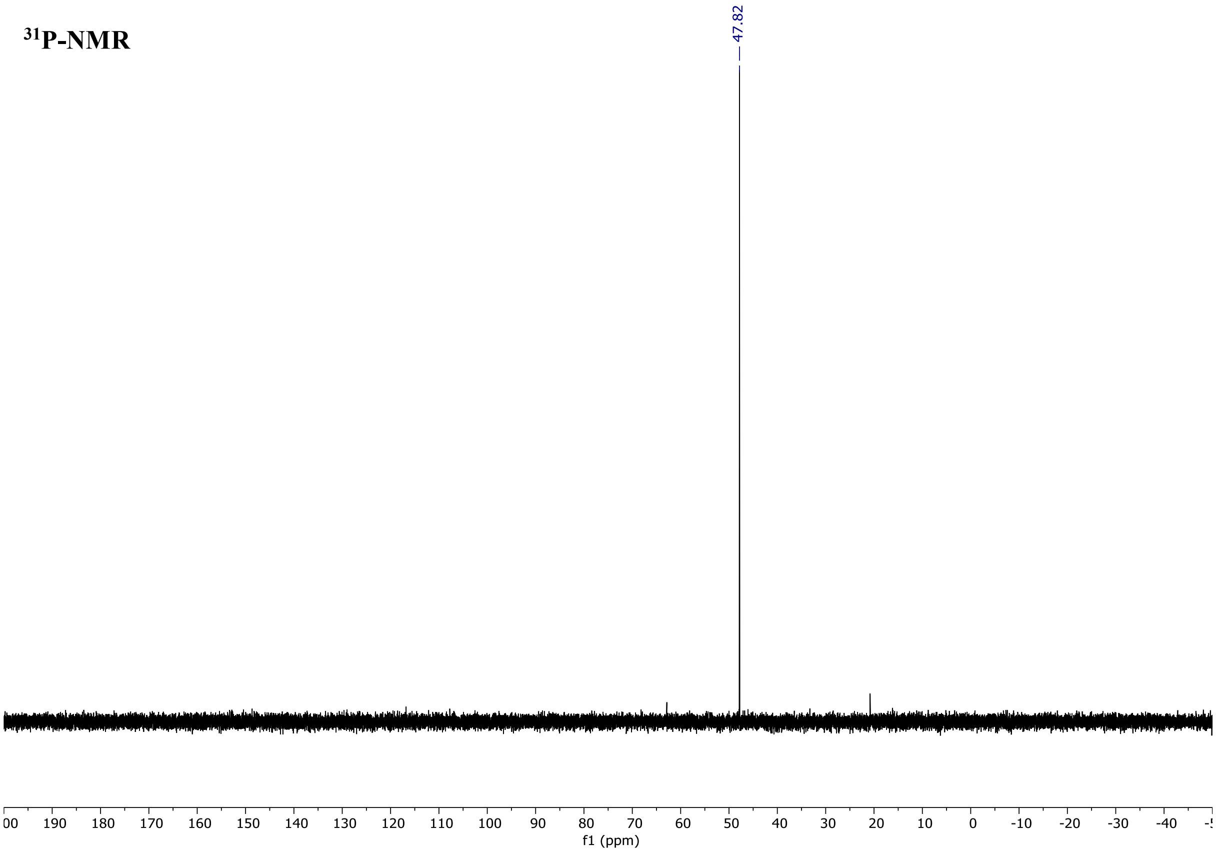

^{13}C-NMR

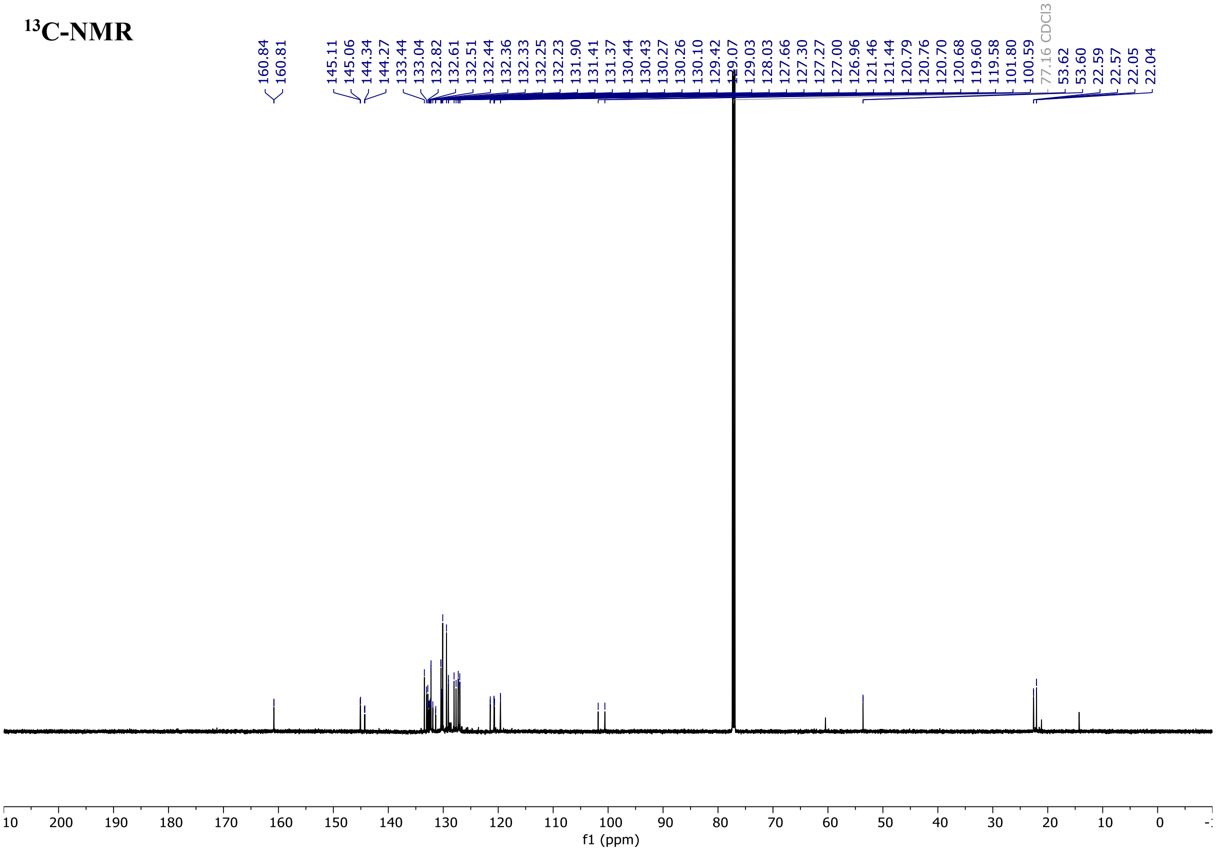

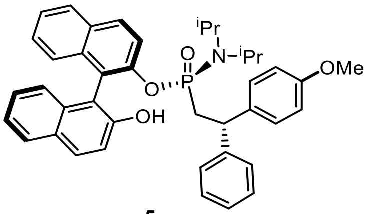

5

^{1}H-NMR

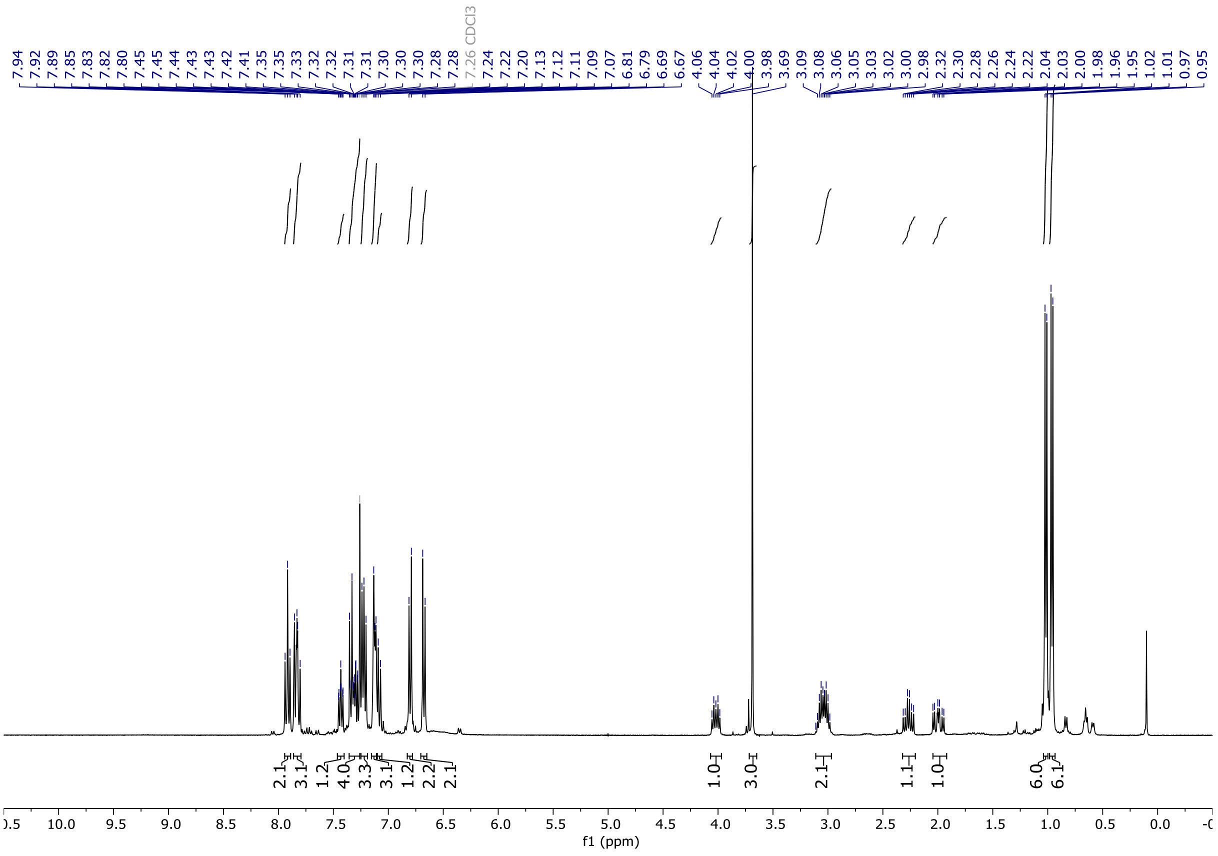

^{31}P-NMR

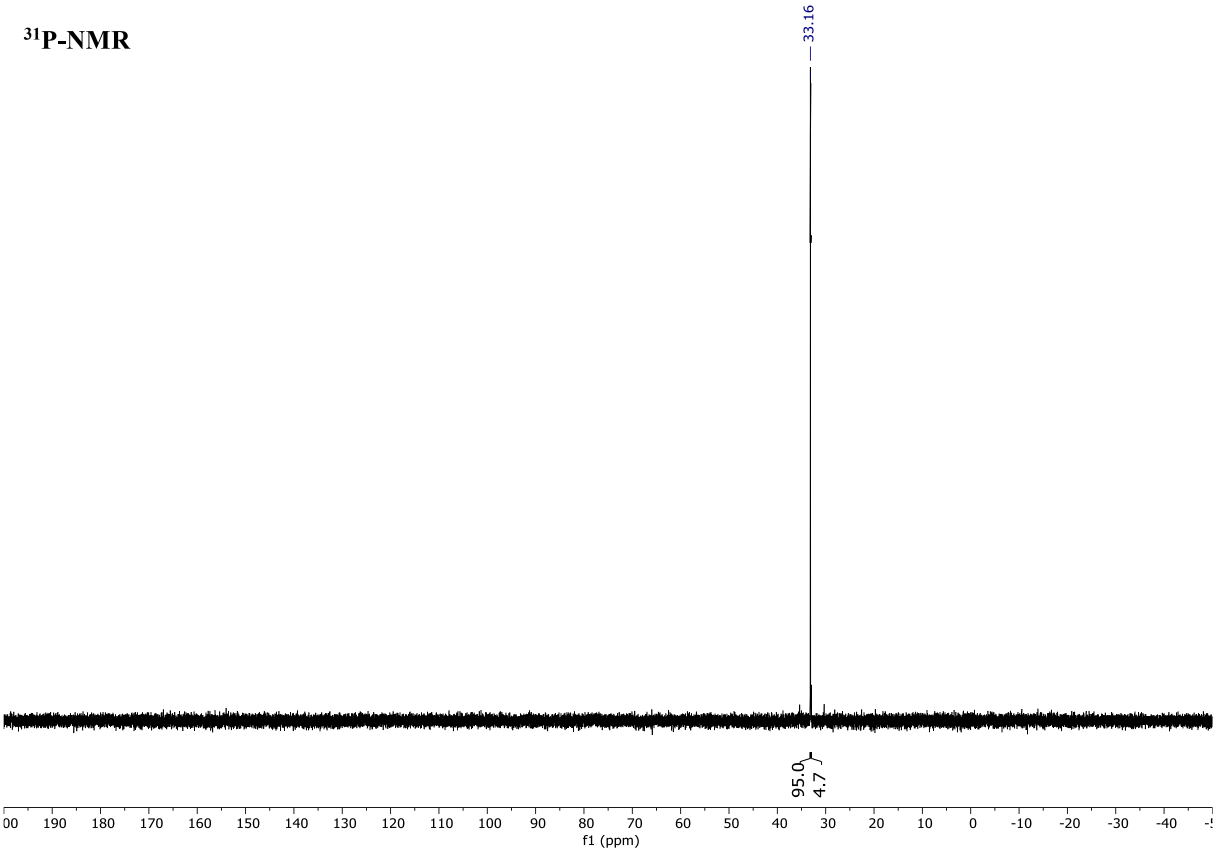

^{13}C-NMR

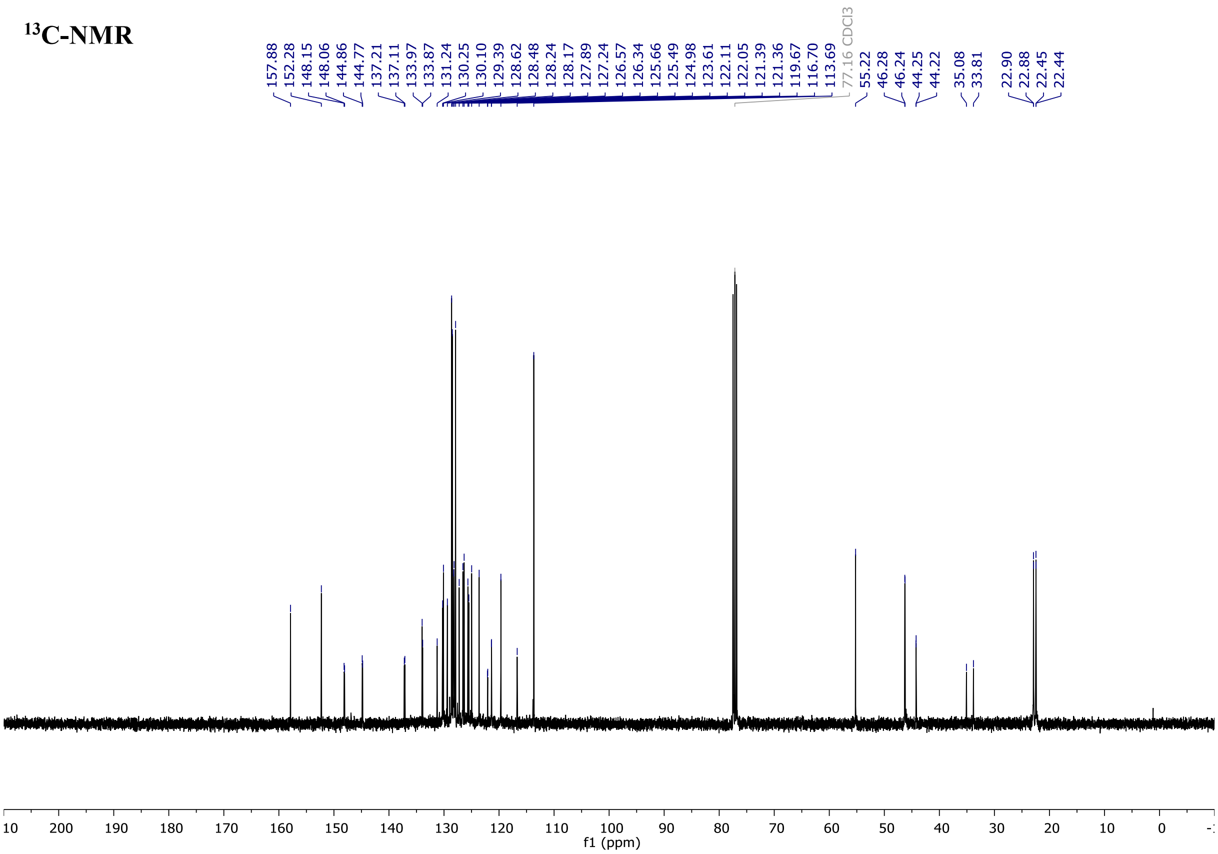

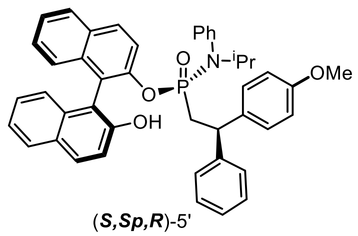
Ph
N–iPr
O
P
OMe
O
OH

(***S*,*Sp*,*R***)-5'

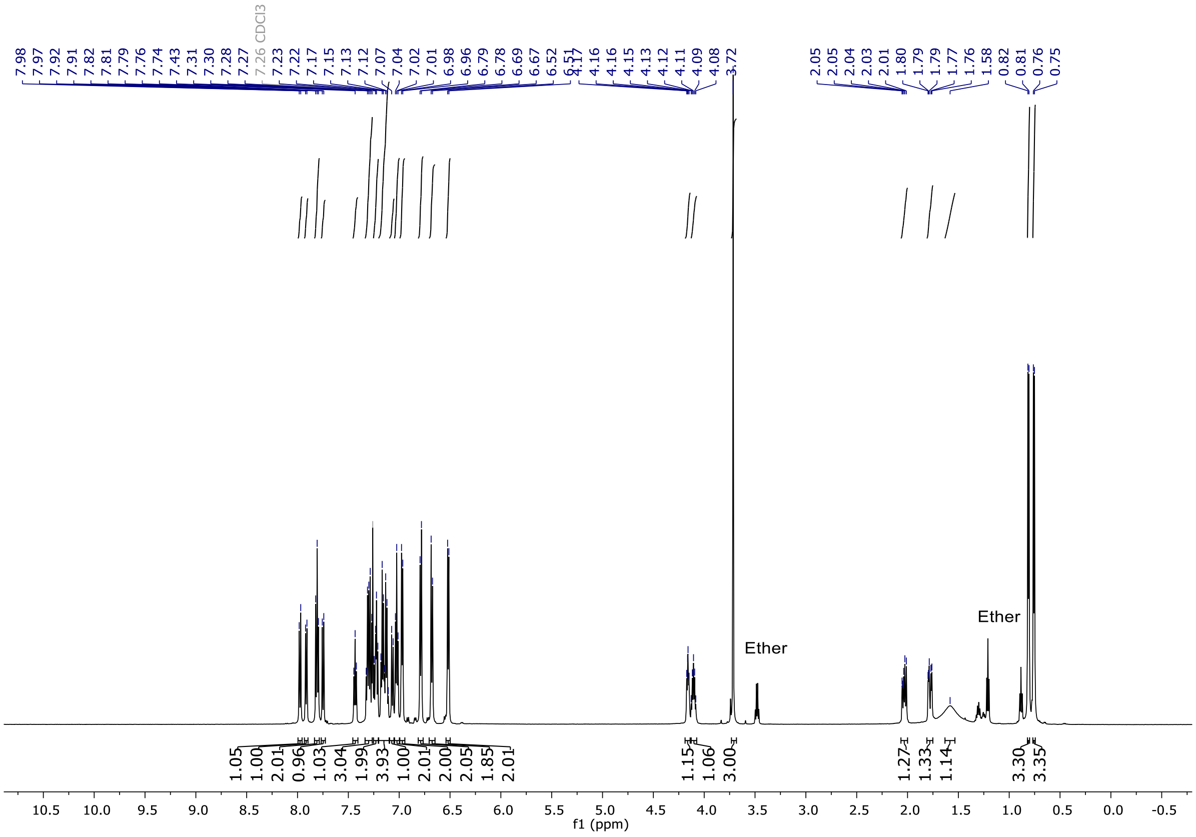
7.98 7.97 7.92 7.91 7.82 7.81 7.79 7.76 7.74 7.43 7.31 7.30 7.28 7.27 7.26 CDCl3 7.23 7.22 7.17 7.15 7.13 7.12 7.07 7.04 7.02 7.01 6.98 6.96 6.79 6.78 6.69 6.67 6.52 6.51 4.17 4.16 4.16 4.15 4.13 4.12 4.11 4.09 4.08 3.72
2.05 2.05 2.04 2.03 2.01 1.80 1.79 1.79 1.77 1.76 1.58 0.82 0.81 0.76 0.75
Ether
Ether
1.05 1.00 2.01 0.96 1.03 3.04 1.99 3.93 1.00 2.01 2.00 2.05 1.85 2.01
1.15 1.06 3.00
1.27 1.33 1.14
3.30 3.35
10.5 10.0 9.5 9.0 8.5 8.0 7.5 7.0 6.5 6.0 5.5 5.0 4.5 4.0 3.5 3.0 2.5 2.0 1.5 1.0 0.5 0.0 -0.5
f1 (ppm)

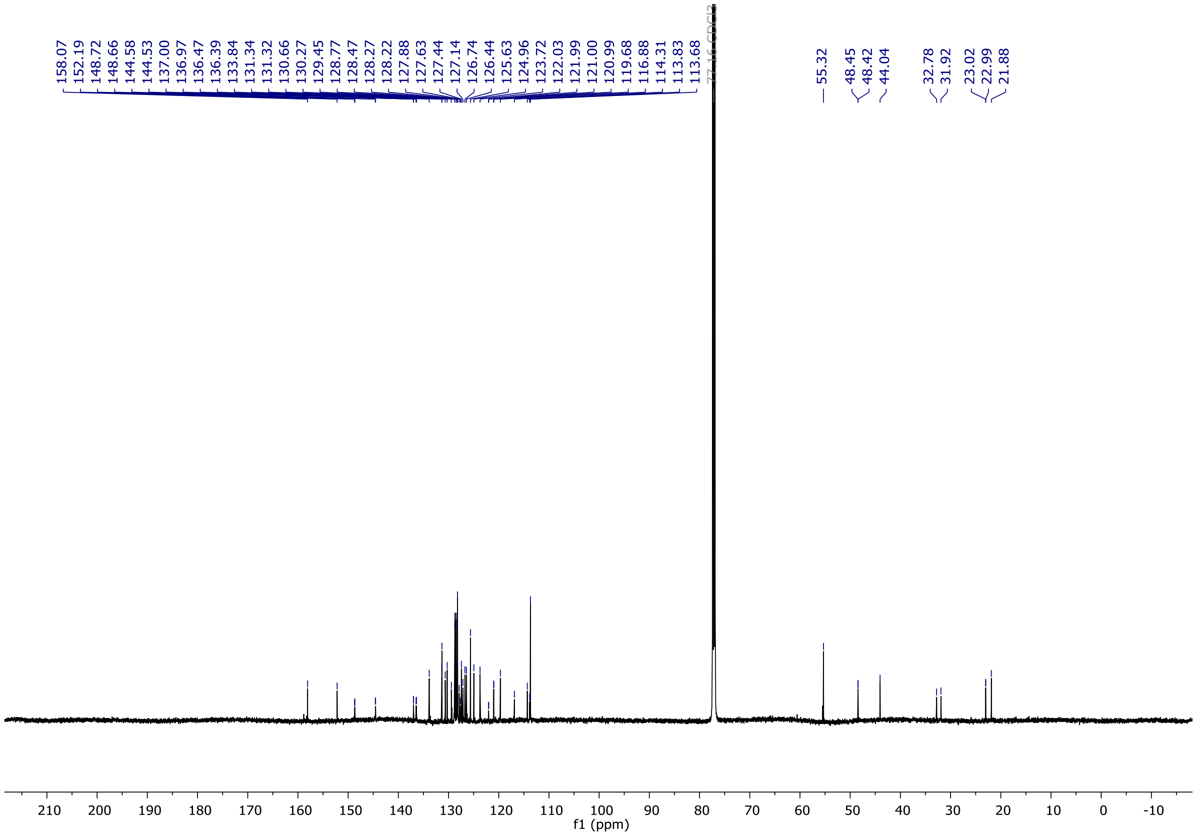
158.07
152.19
148.72
148.66
144.58
144.53
137.00
136.97
136.47
136.39
133.84
131.34
131.32
130.66
130.27
129.45
128.77
128.47
128.27
128.27
128.22
127.88
127.63
127.44
127.14
126.74
126.44
125.63
124.96
123.72
122.03
121.99
121.00
120.99
119.68
116.88
114.31
113.83
113.68
77.16 CDCl3
55.32
48.45
48.42
44.04
32.78
31.92
23.02
22.99
21.88
f1 (ppm)

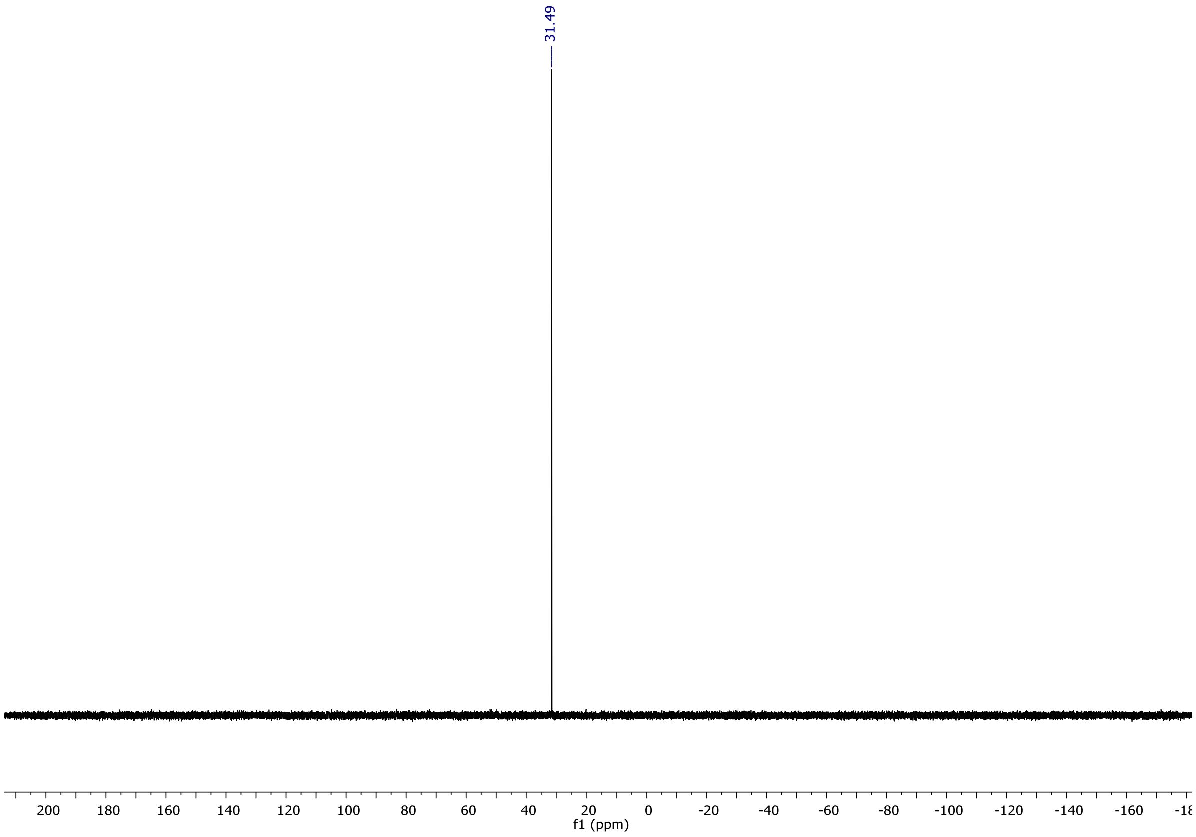
31.49
f1 (ppm)

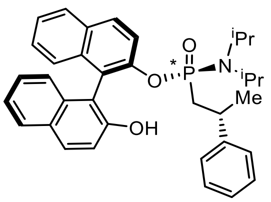

6

^{1}H-NMR

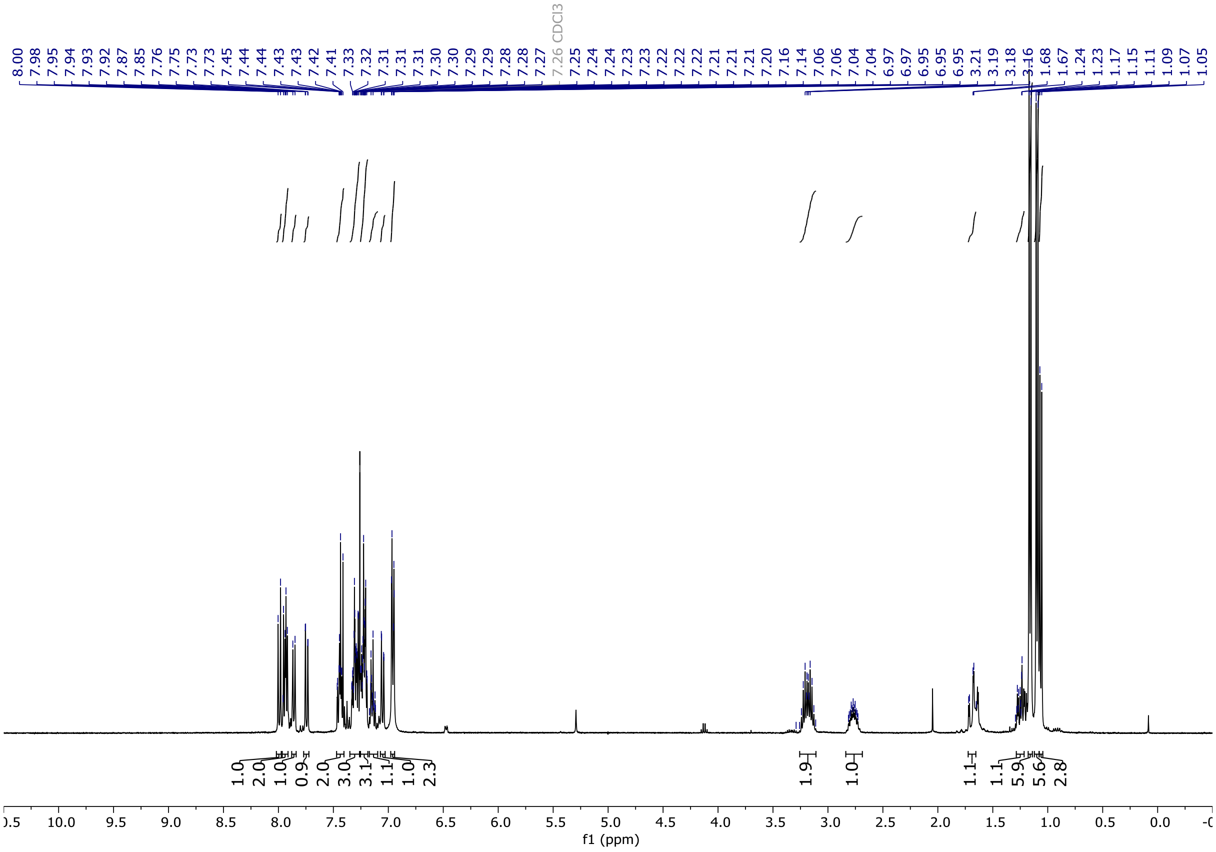

^{31}P-NMR

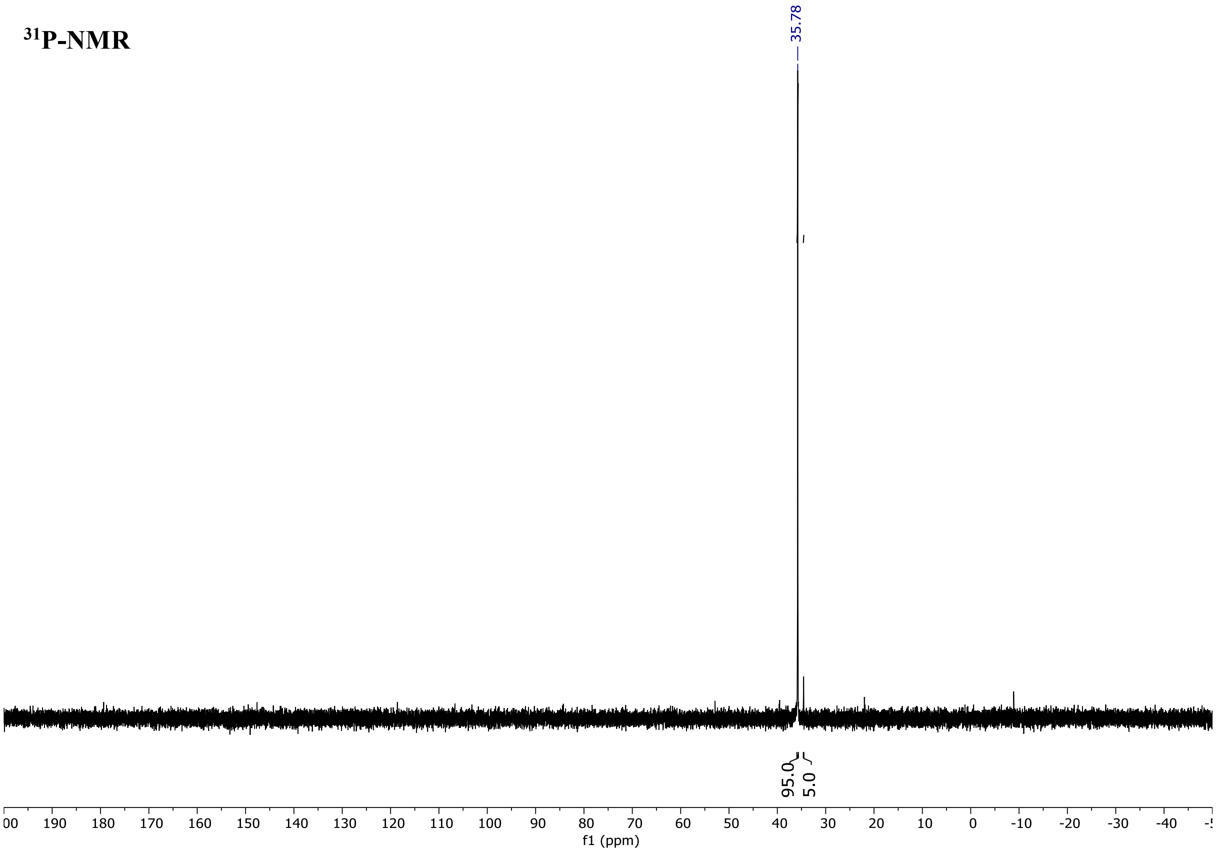

^{13}C-NMR

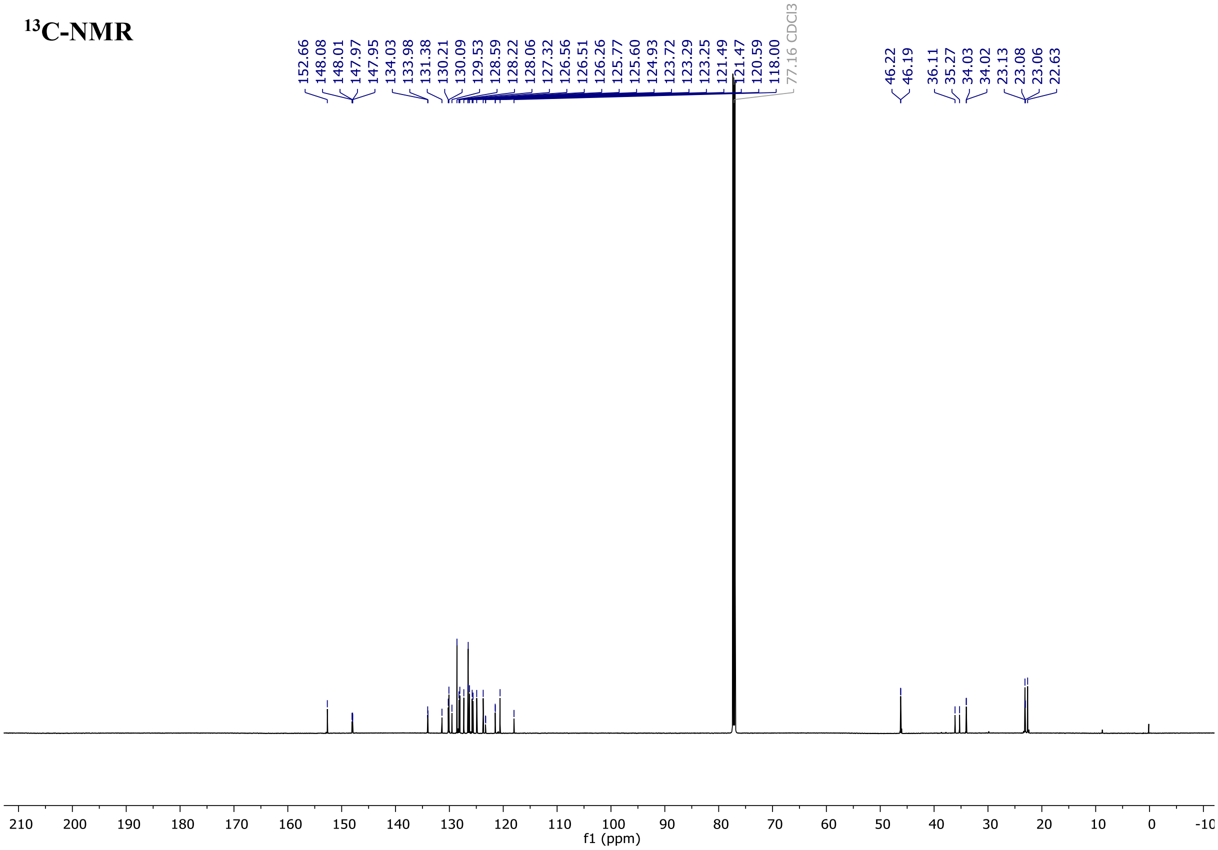

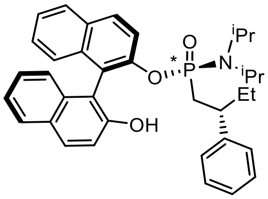

7

^{1}H-NMR

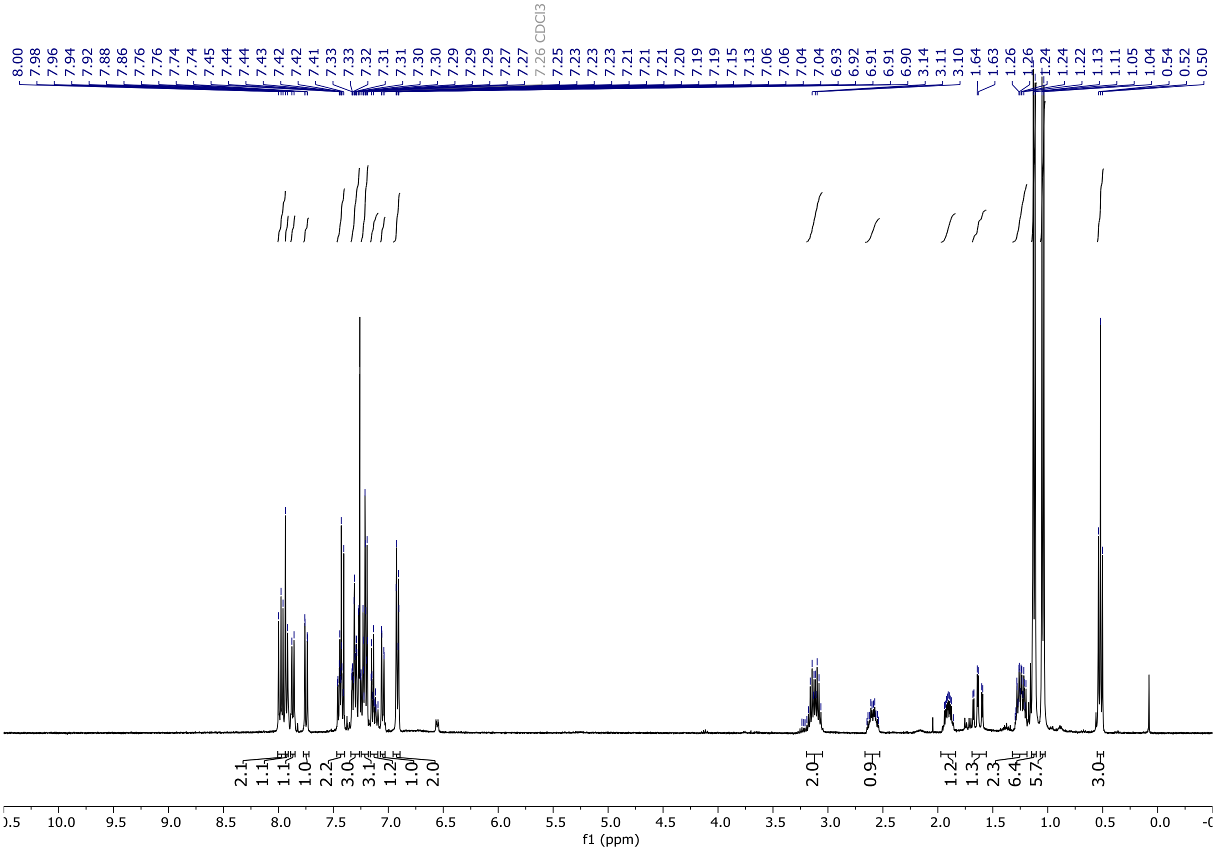

^{31}P-NMR

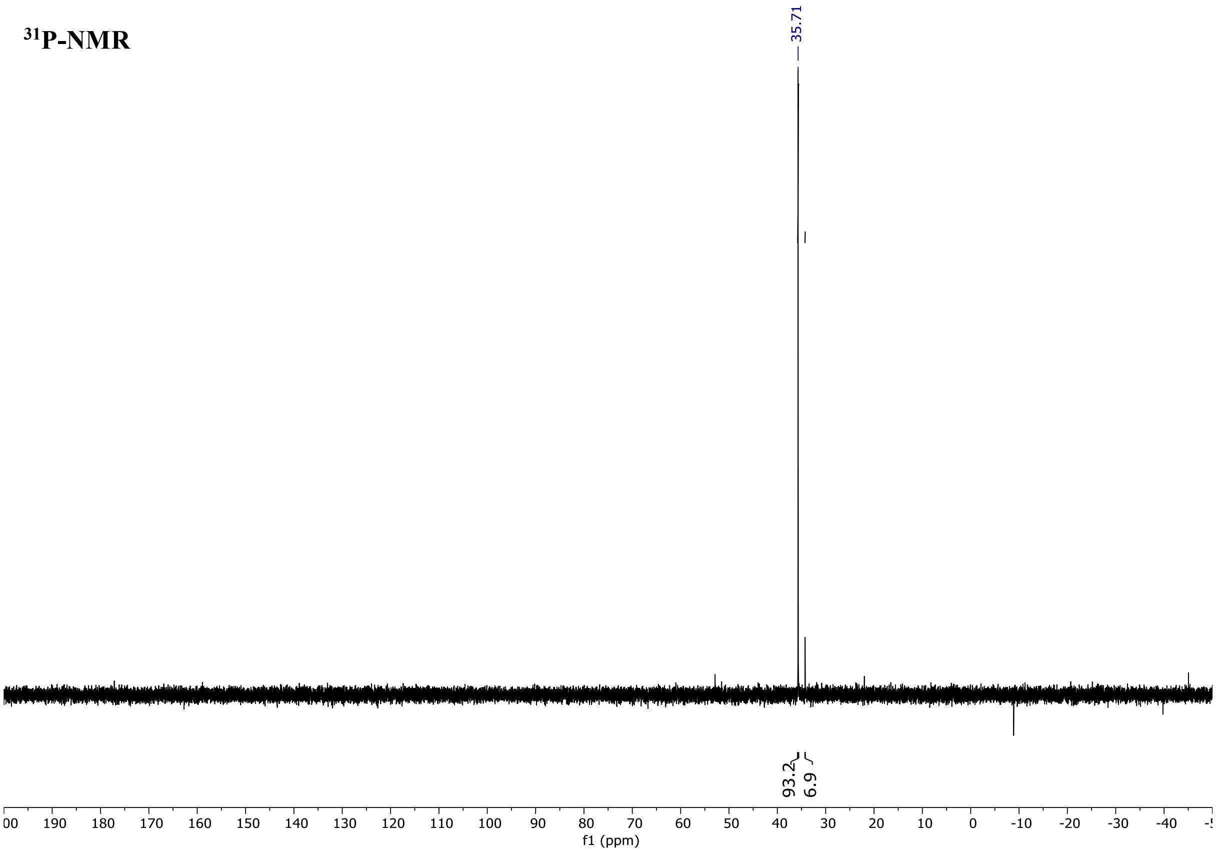

^{13}C-NMR

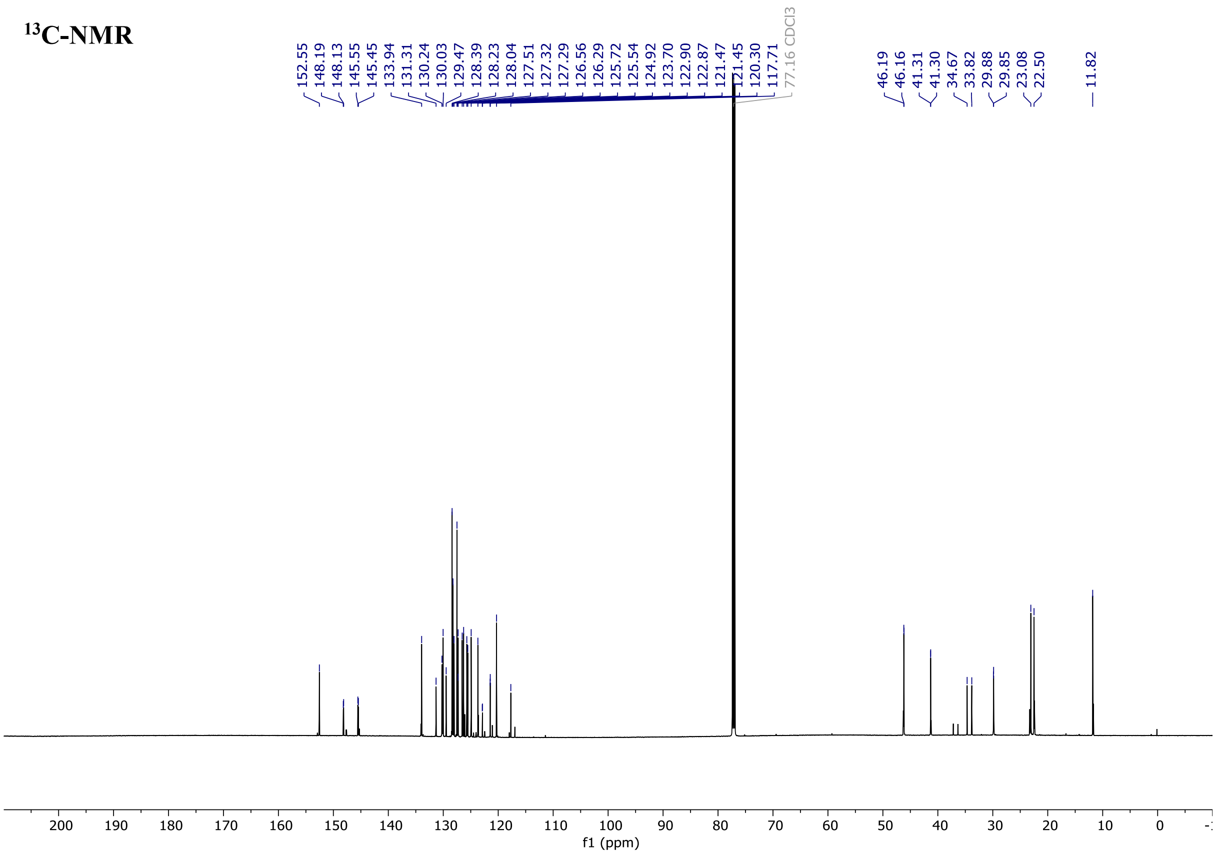

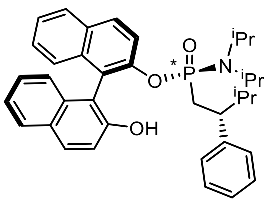

8

^{1}H-NMR

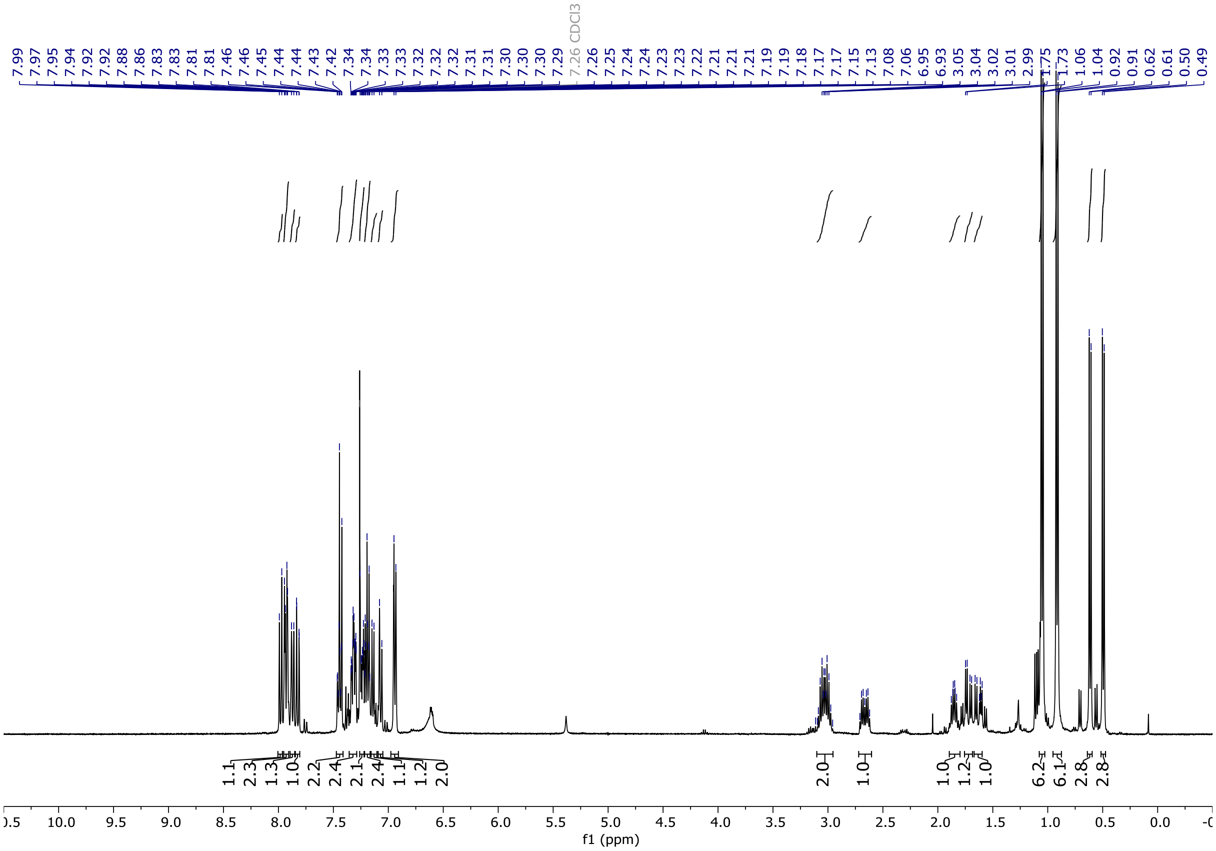

^{31}P-NMR

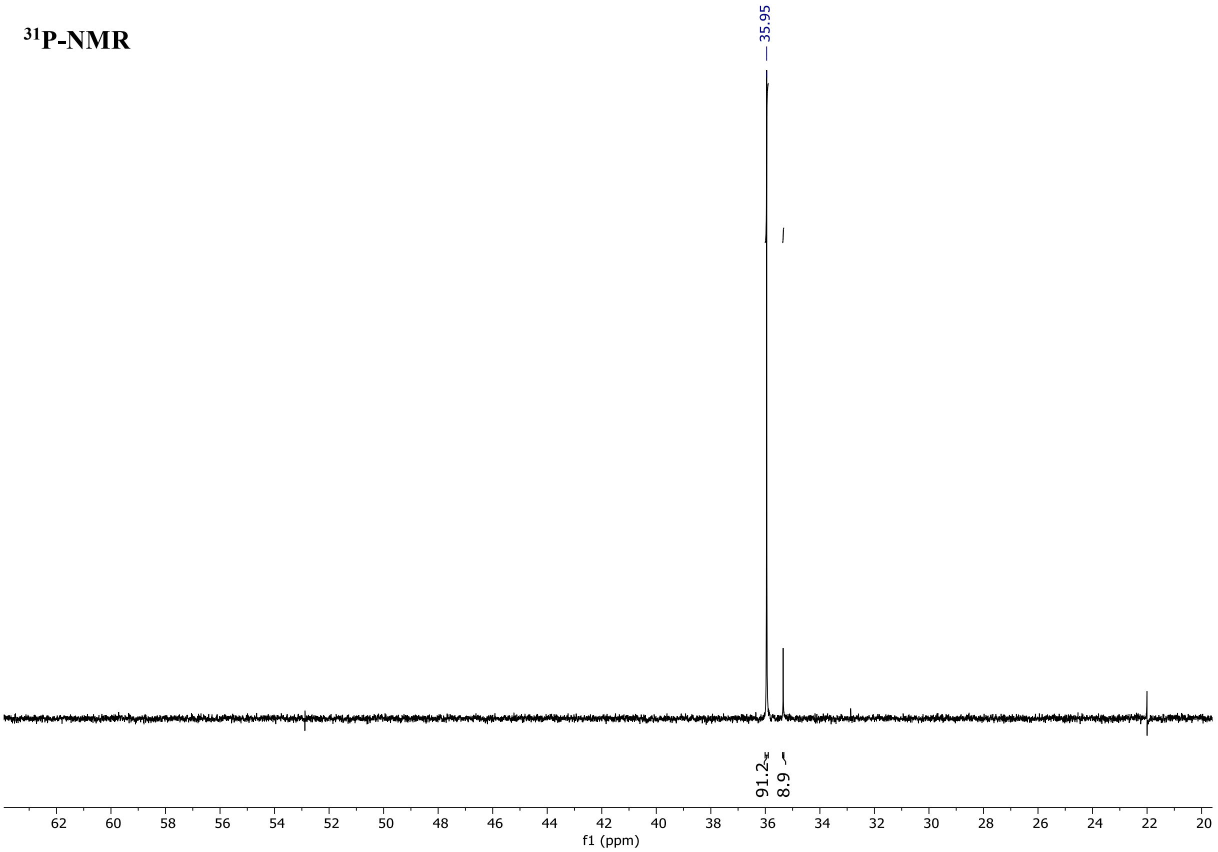

^{13}C-NMR

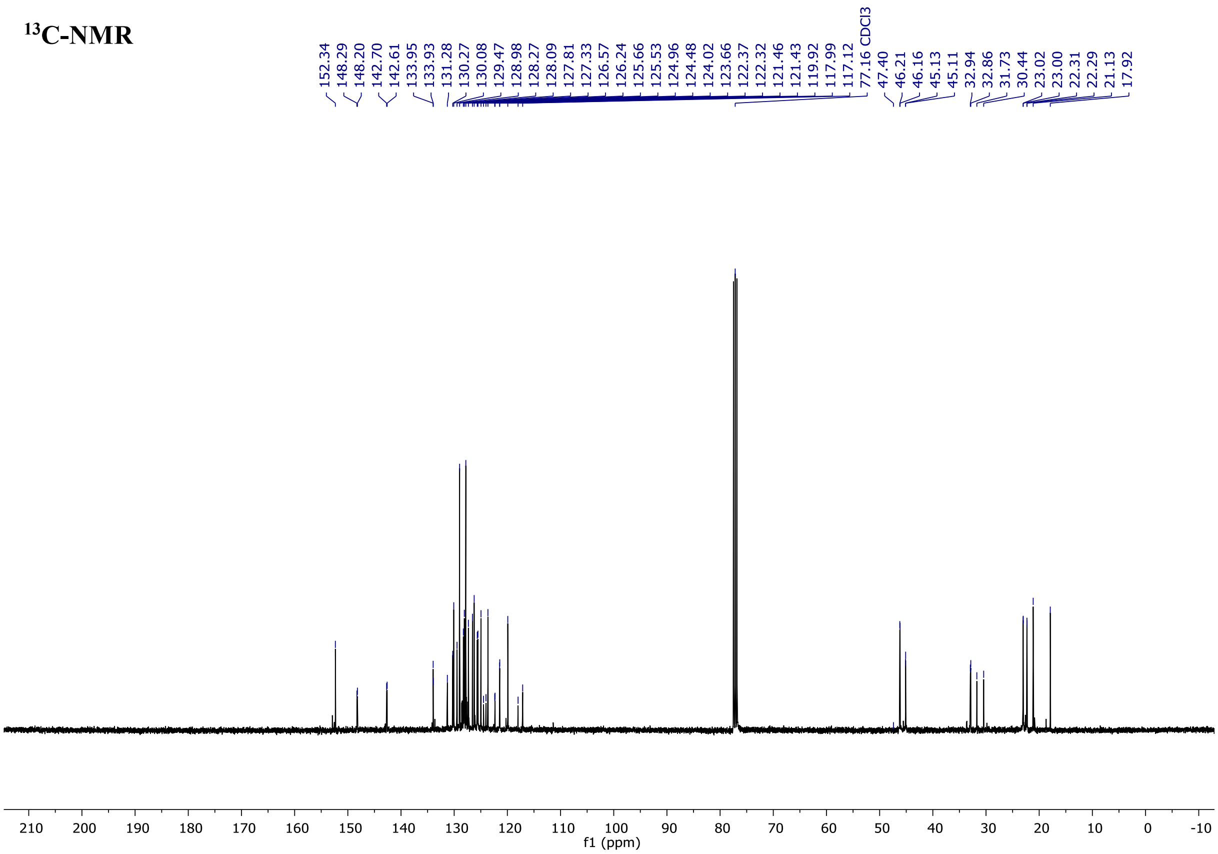

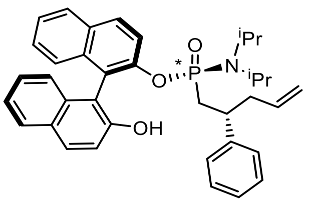

9

^{1}H-NMR

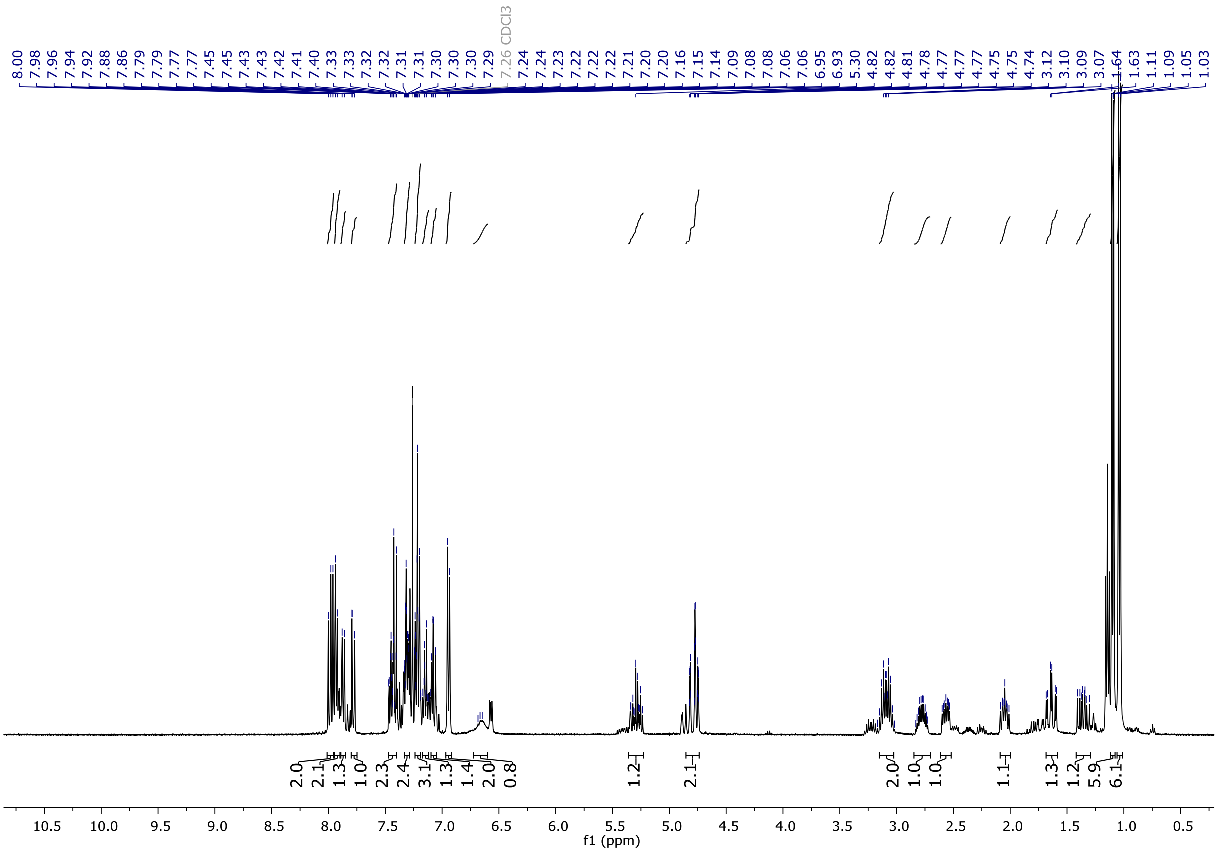

^{31}P-NMR

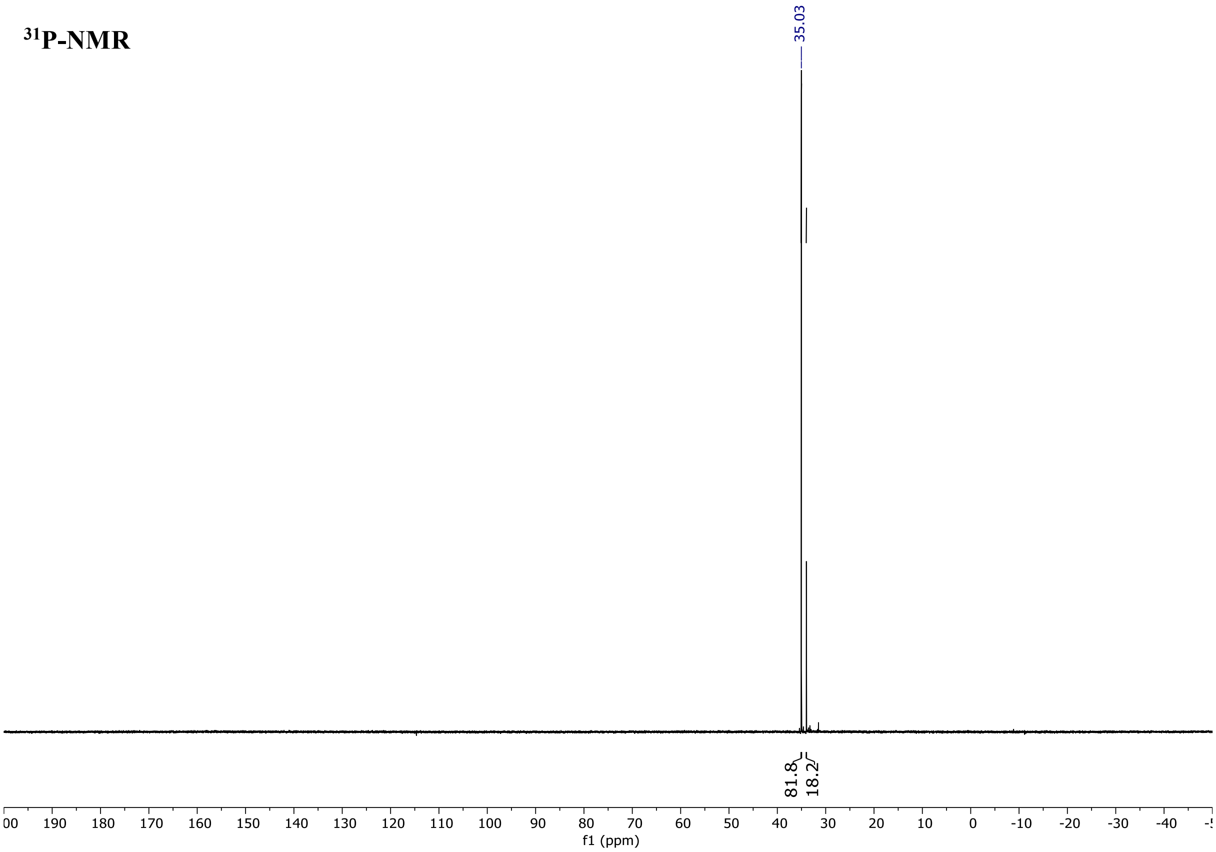

^{13}C-NMR

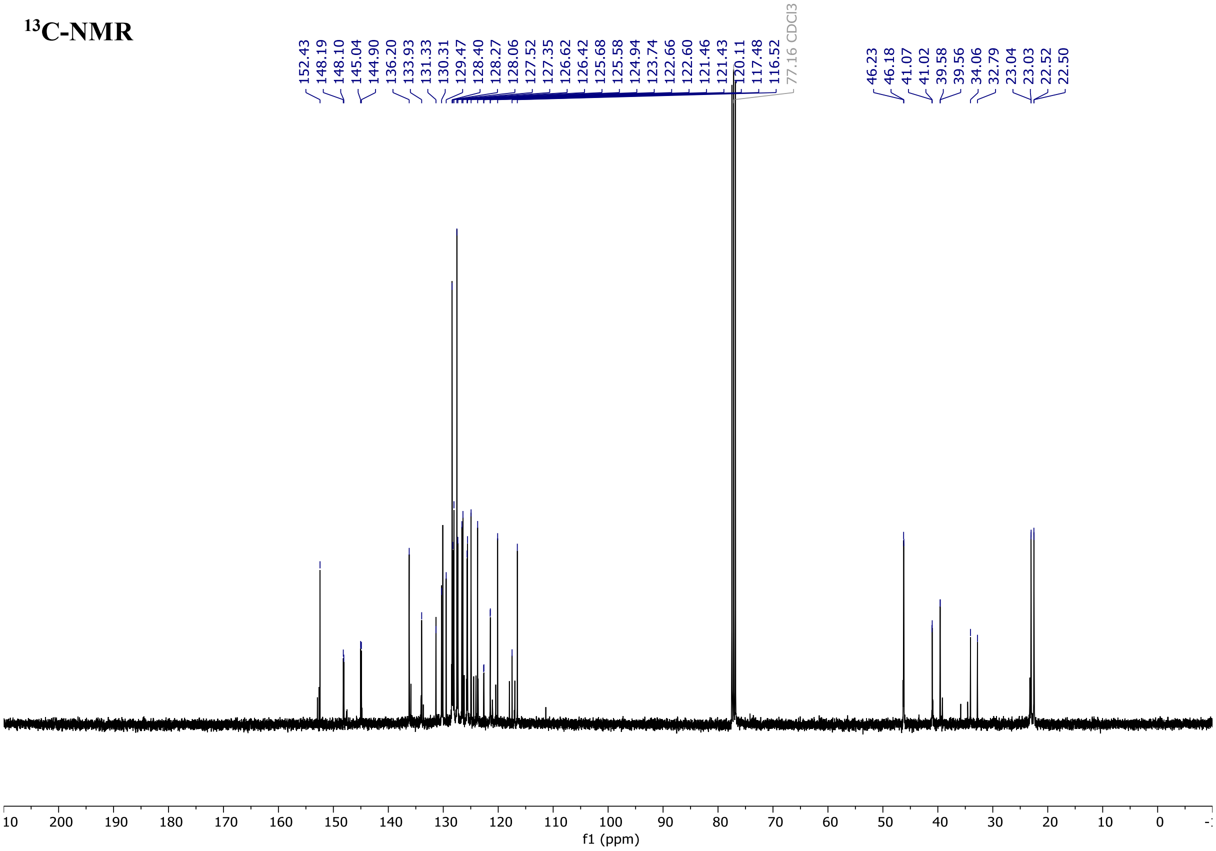

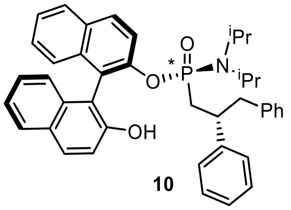

10

^{1}H-NMR

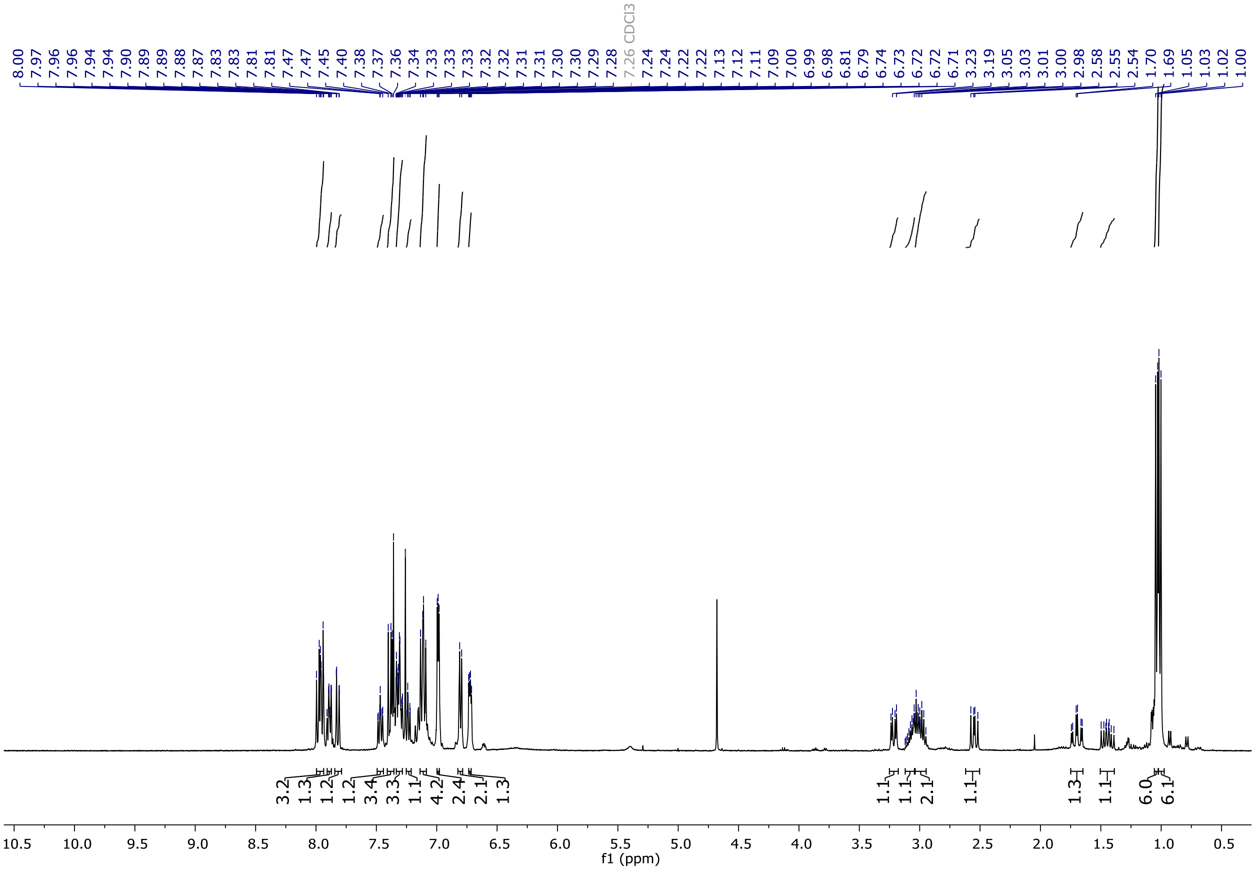

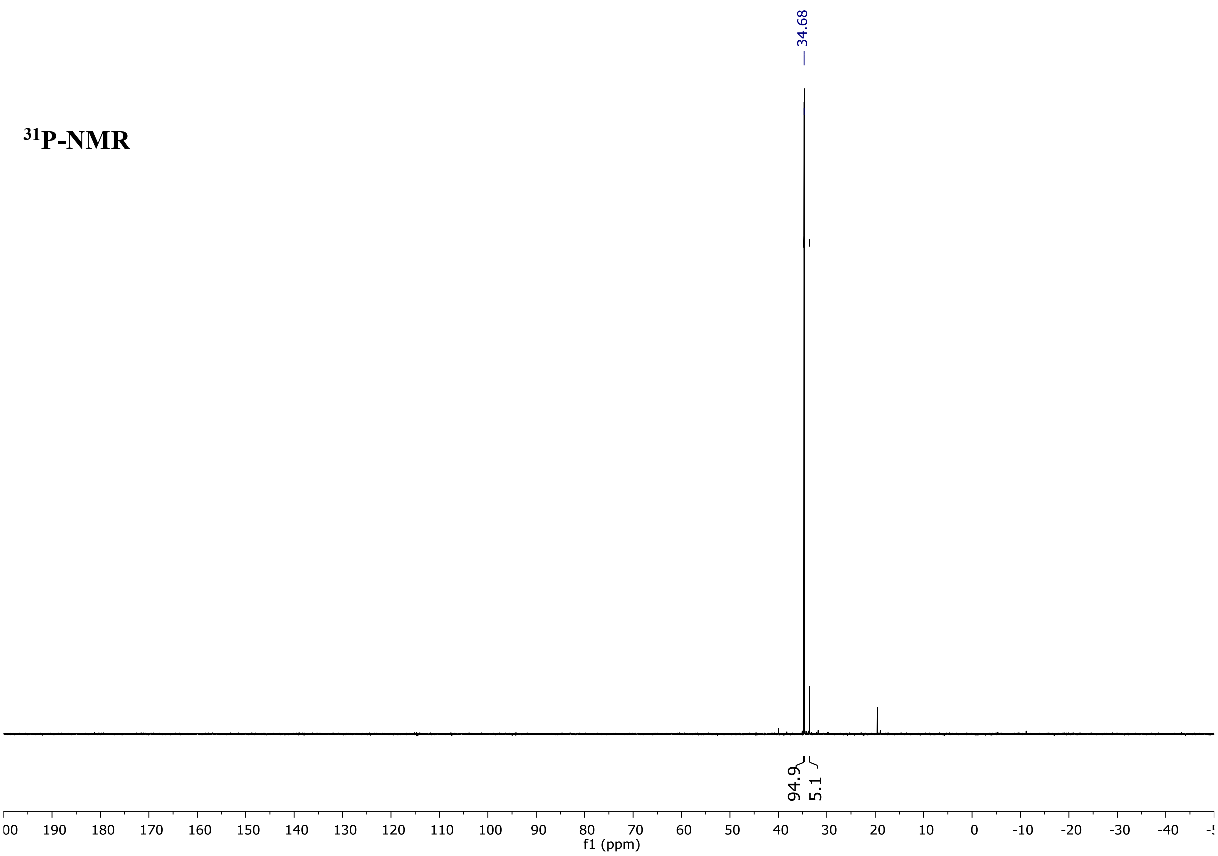

^{31}P-NMR
34.68
94.9
5.1
f1 (ppm)

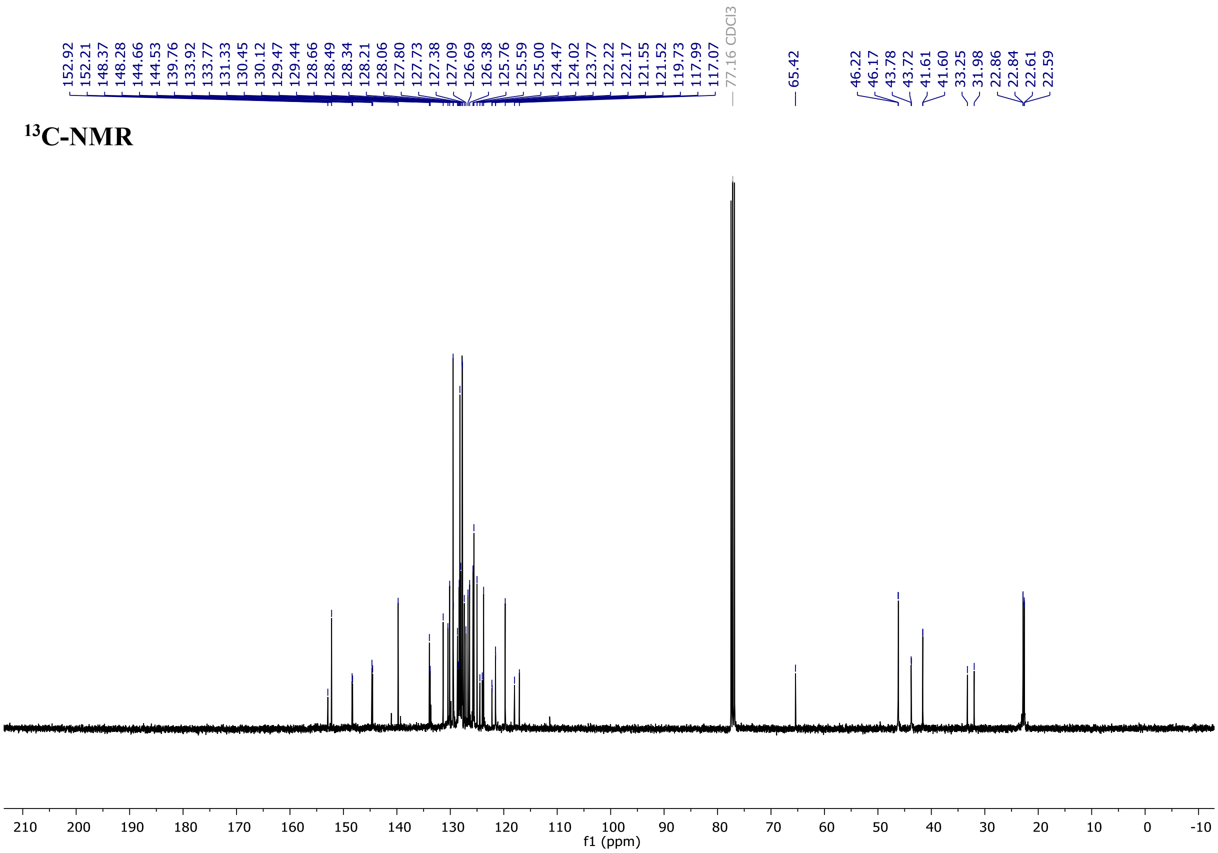

^{13}C-NMR
152.92 152.21 148.37 148.28 144.66 144.53 139.76 133.92 133.77 131.33 130.45 130.12 129.47 129.44 128.66 128.49 128.34 128.21 128.06 127.80 127.73 127.38 127.09 126.69 126.38 125.76 125.59 125.00 124.47 124.02 123.77 122.22 122.17 121.55 121.52 119.73 117.99 117.07
77.16 CDCl3
65.42
46.22 46.17 43.78 43.72 41.61 41.60 33.25 31.98 22.86 22.84 22.61 22.59
f1 (ppm)

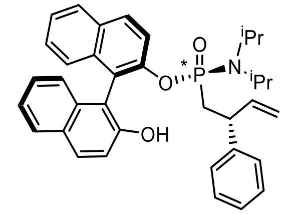

11

^{1}H-NMR

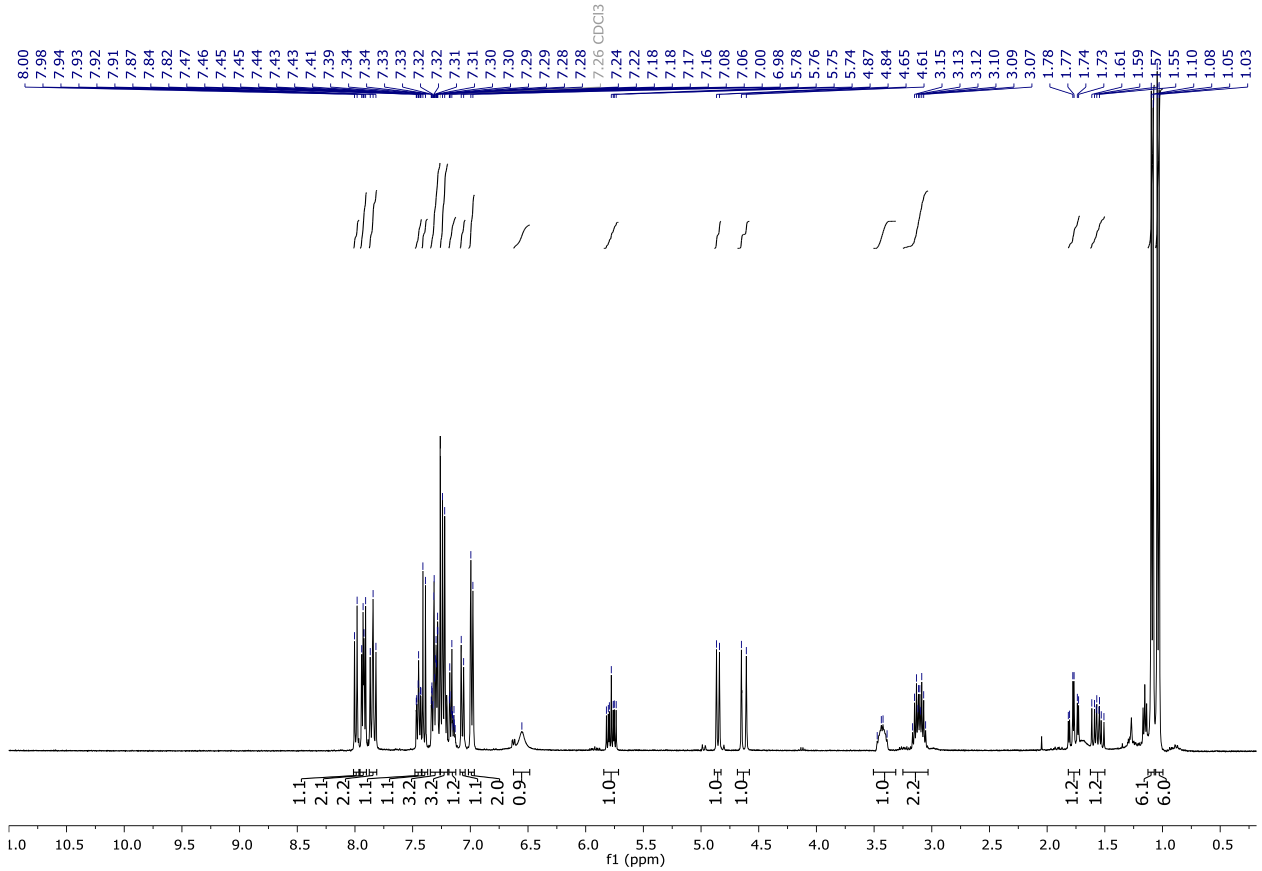

^{31}P-NMR

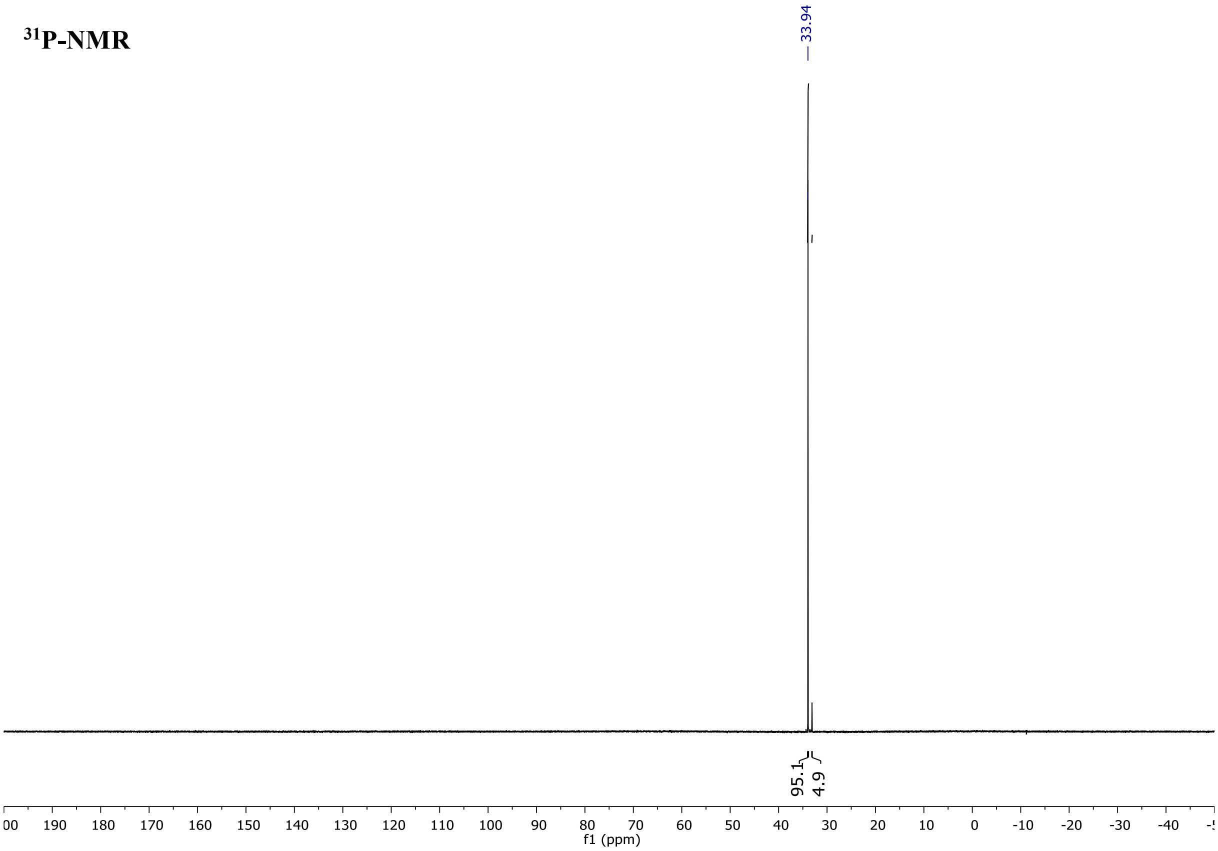

^{13}C-NMR

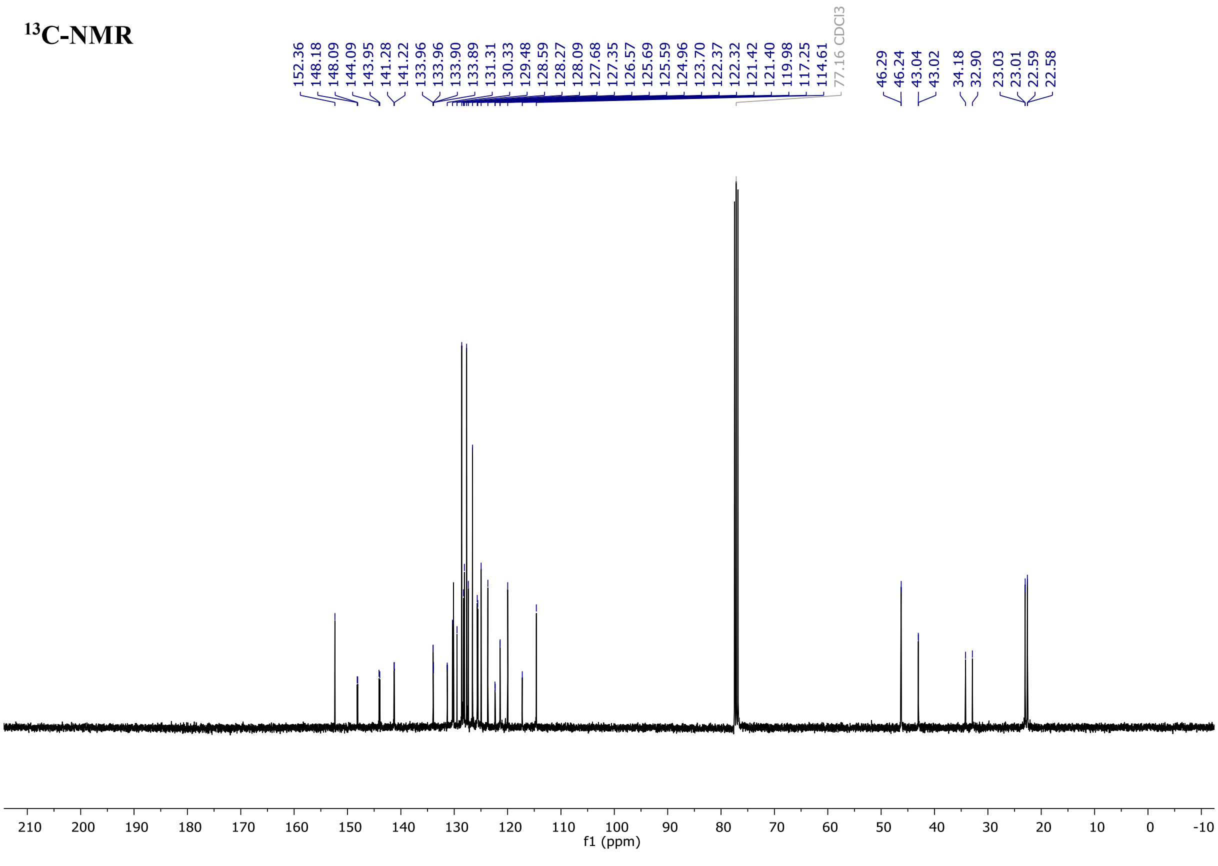

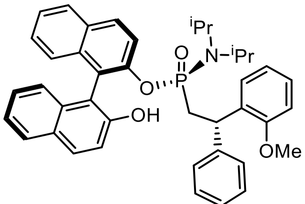

iPr
N–iPr
O
P
O
OH
OMe

12

^{1}H-NMR

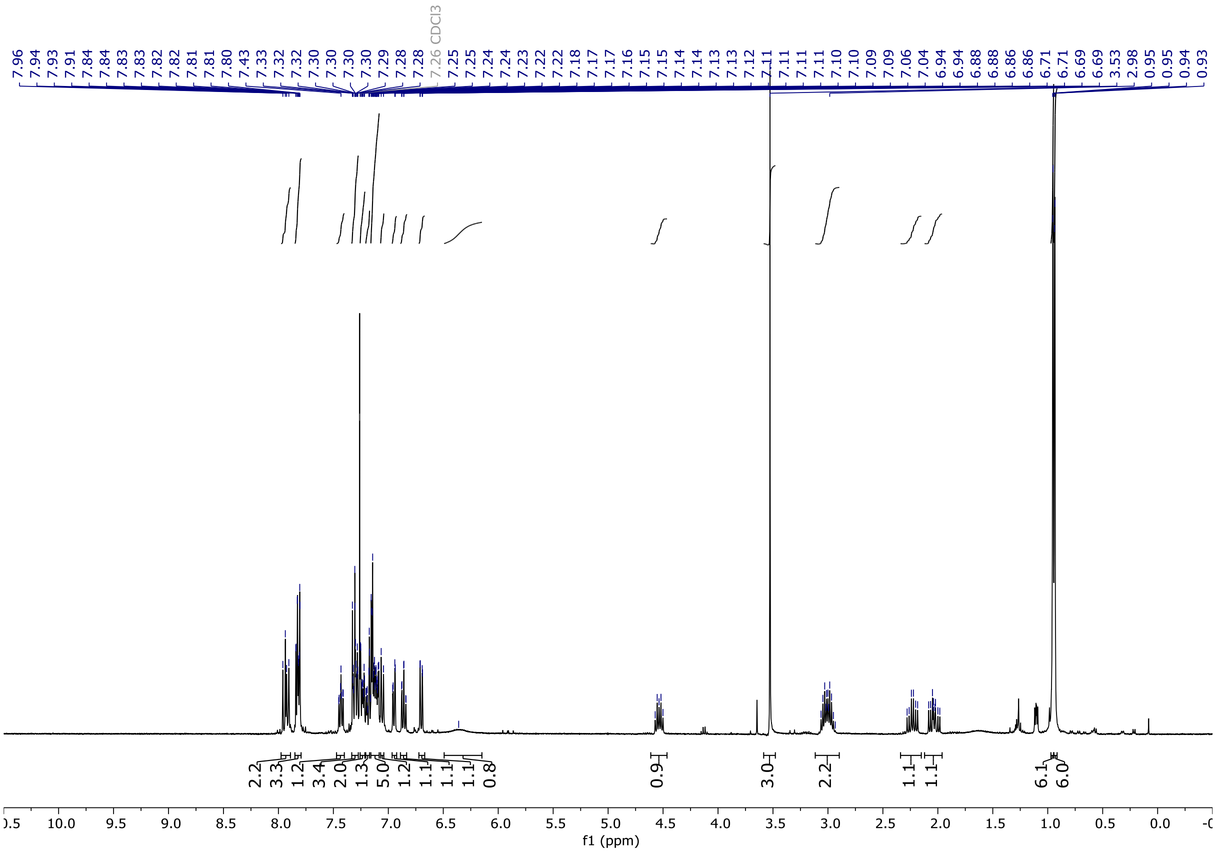

7.26 CDCl3
2.2
3.3
1.2
3.4
2.0
1.3
5.0
1.2
1.1
1.1
1.1
0.8
0.9
3.0
2.2
1.1
1.1
6.1
6.0
f1 (ppm)

^{31}P-NMR

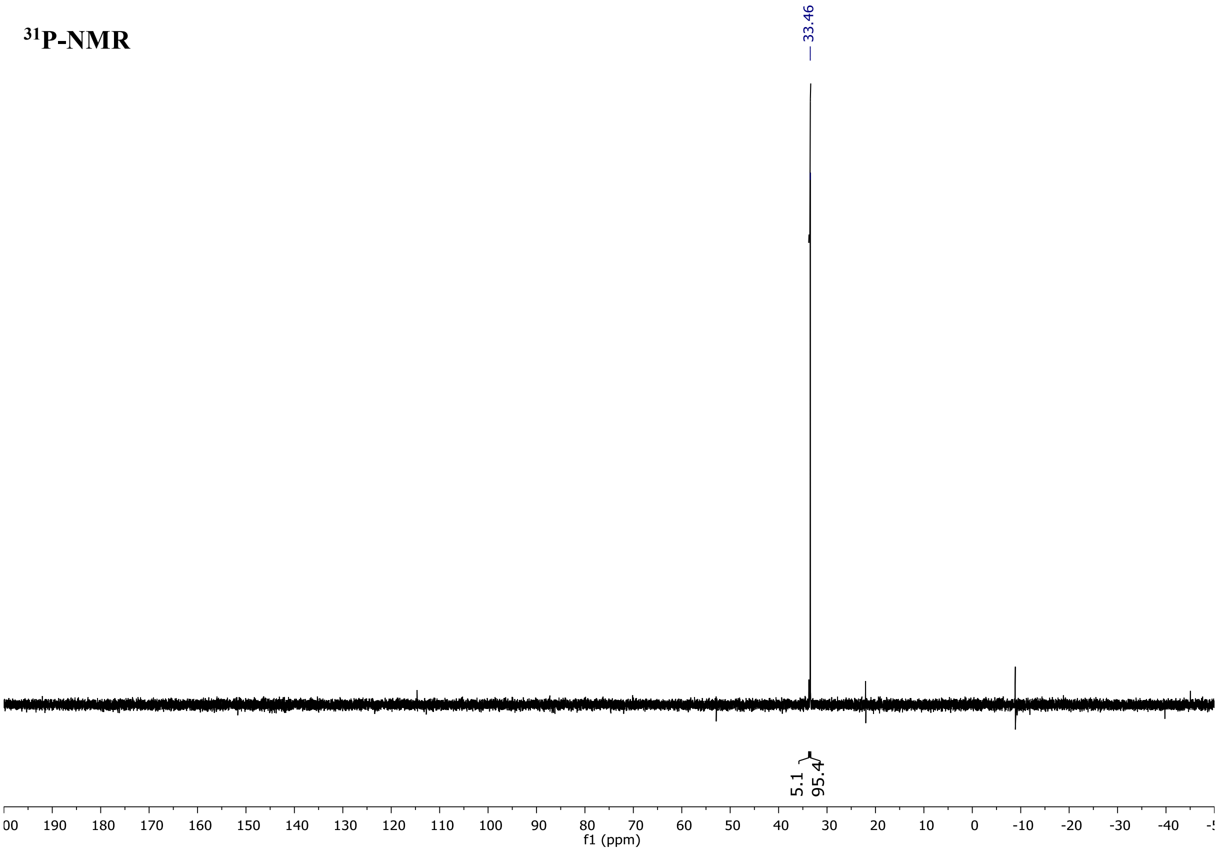

^{13}C-NMR

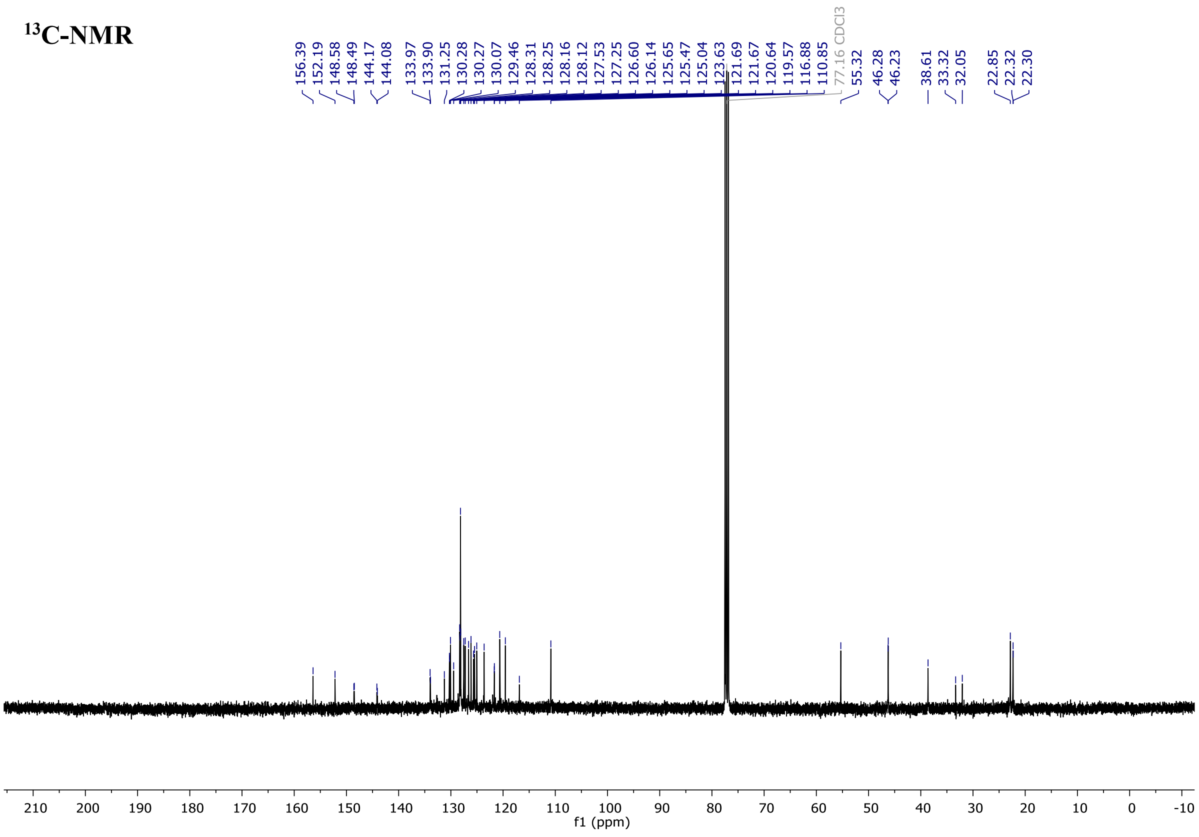

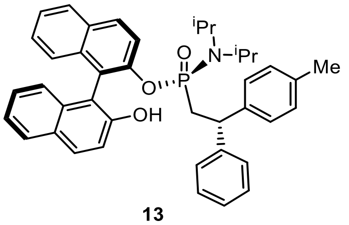

13

^{1}H-NMR

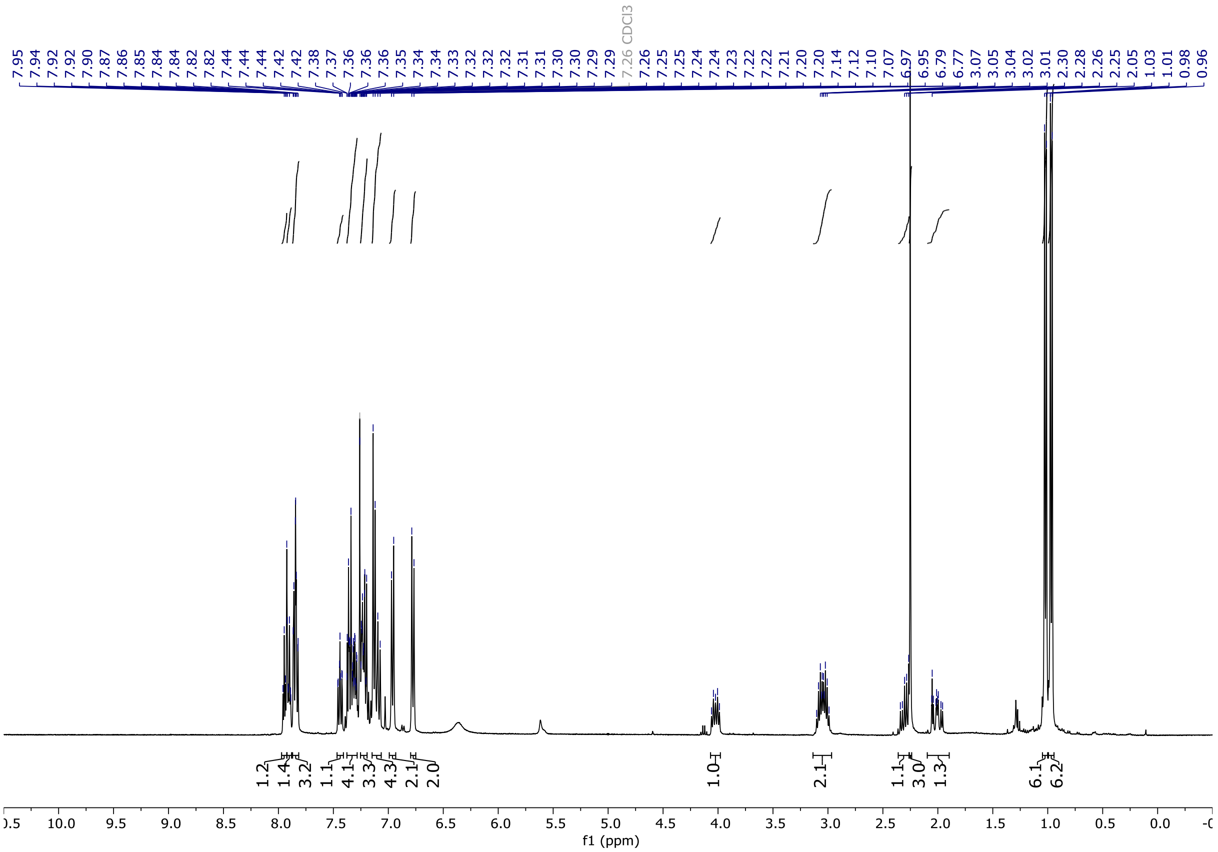

7.95 7.94 7.92 7.92 7.90 7.87 7.86 7.85 7.84 7.84 7.82 7.82 7.44 7.44 7.44 7.42 7.42 7.38 7.37 7.36 7.36 7.36 7.35 7.34 7.34 7.33 7.32 7.32 7.32 7.31 7.31 7.30 7.30 7.29 7.29 7.26 CDCl3 7.25 7.25 7.24 7.24 7.23 7.22 7.22 7.21 7.20 7.20 7.14 7.12 7.10 7.07 6.97 6.95 6.79 6.77 3.07 3.05 3.04 3.02 3.01 2.30 2.28 2.26 2.25 2.05 1.03 1.01 0.98 0.96
1.2 1.4 3.2 1.1 4.1 3.3 4.3 2.1 2.0 1.0 2.1 1.1 3.0 1.3 6.1 6.2
f1 (ppm)

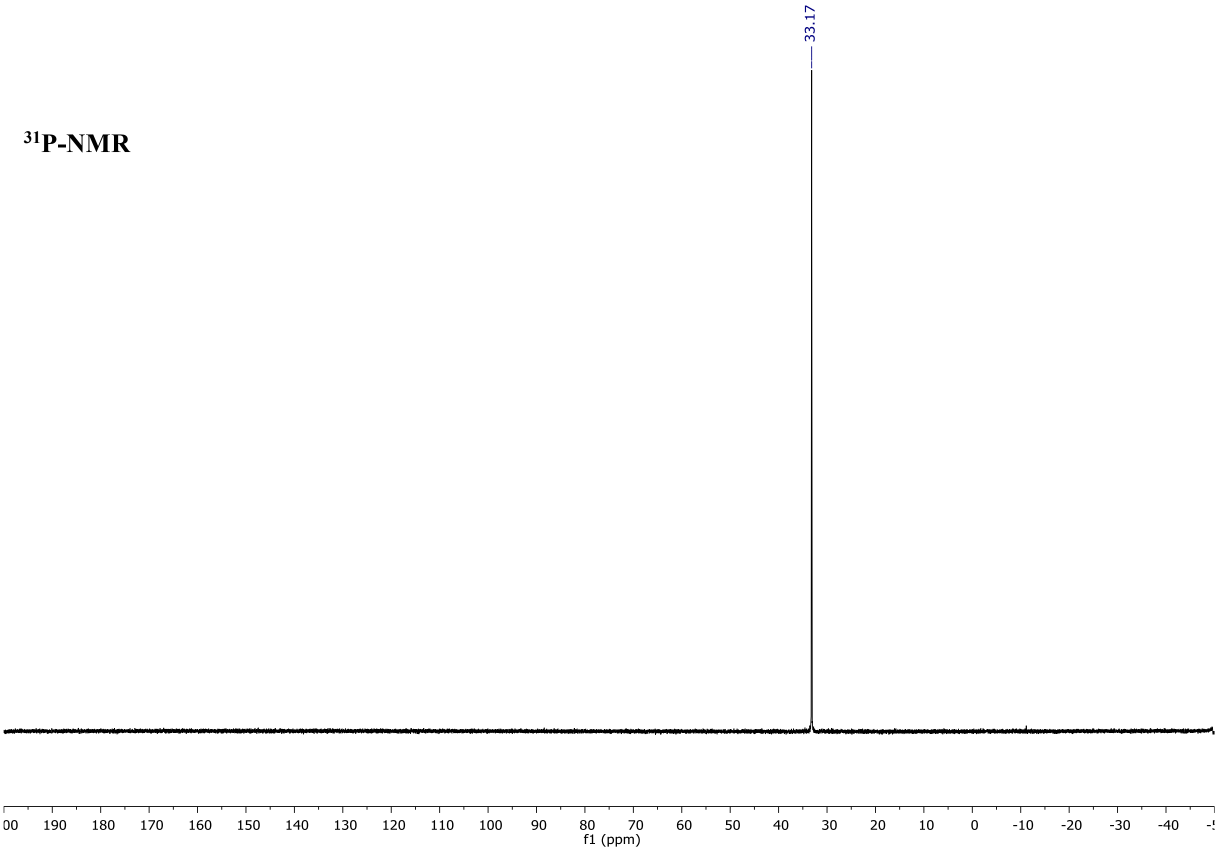

33.17
³¹P-NMR
f1 (ppm)

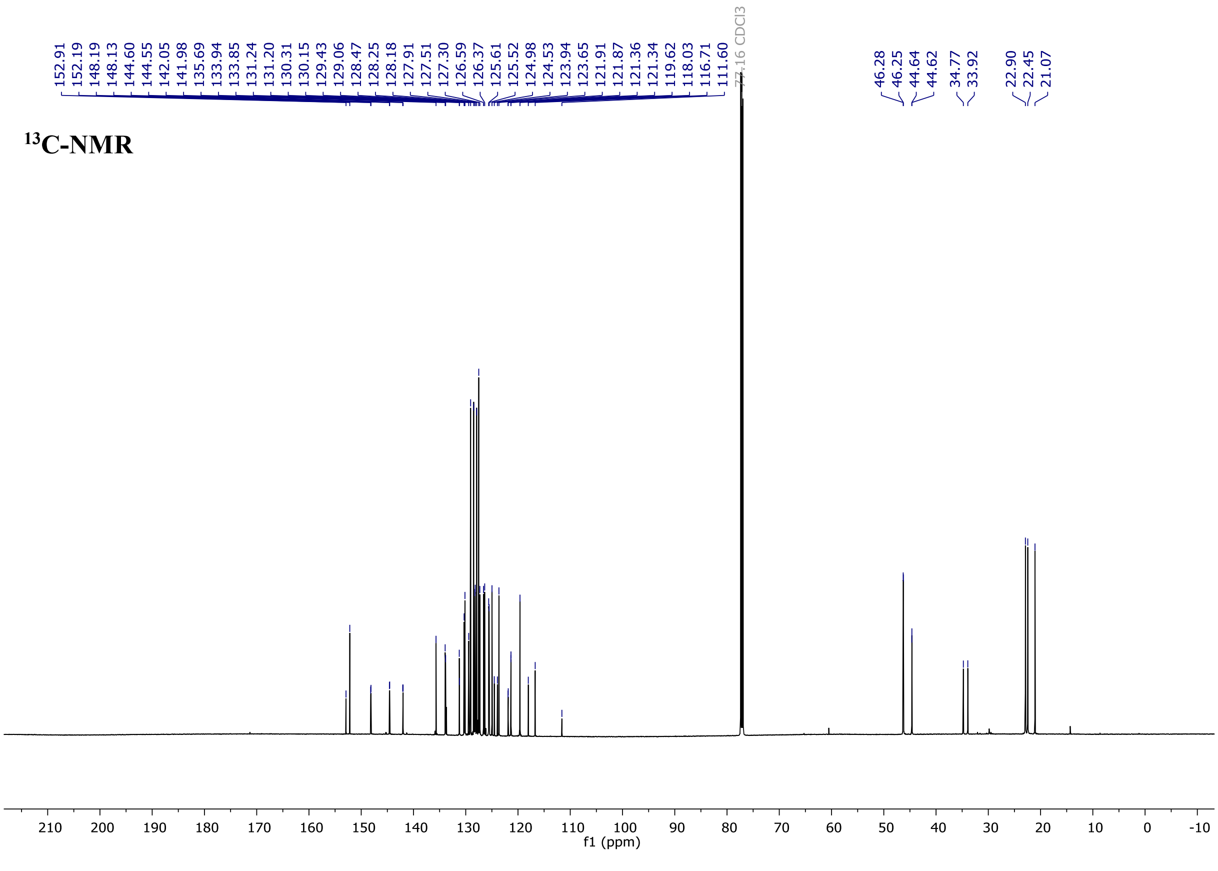

152.91
152.19
148.19
148.13
144.60
144.55
142.05
141.98
135.69
133.94
133.85
131.24
131.20
130.31
130.15
129.43
129.06
128.47
128.25
128.18
127.91
127.51
127.30
126.59
126.37
125.61
125.52
124.98
124.53
123.94
123.65
121.91
121.87
121.36
121.34
119.62
118.03
116.71
111.60
77.16 CDCl3
46.28
46.25
44.64
44.62
34.77
33.92
22.90
22.45
21.07
¹³C-NMR
f1 (ppm)

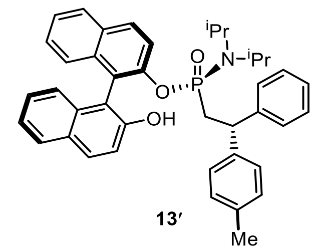

O
iPr
N
iPr
O
P
OH
Me

13′

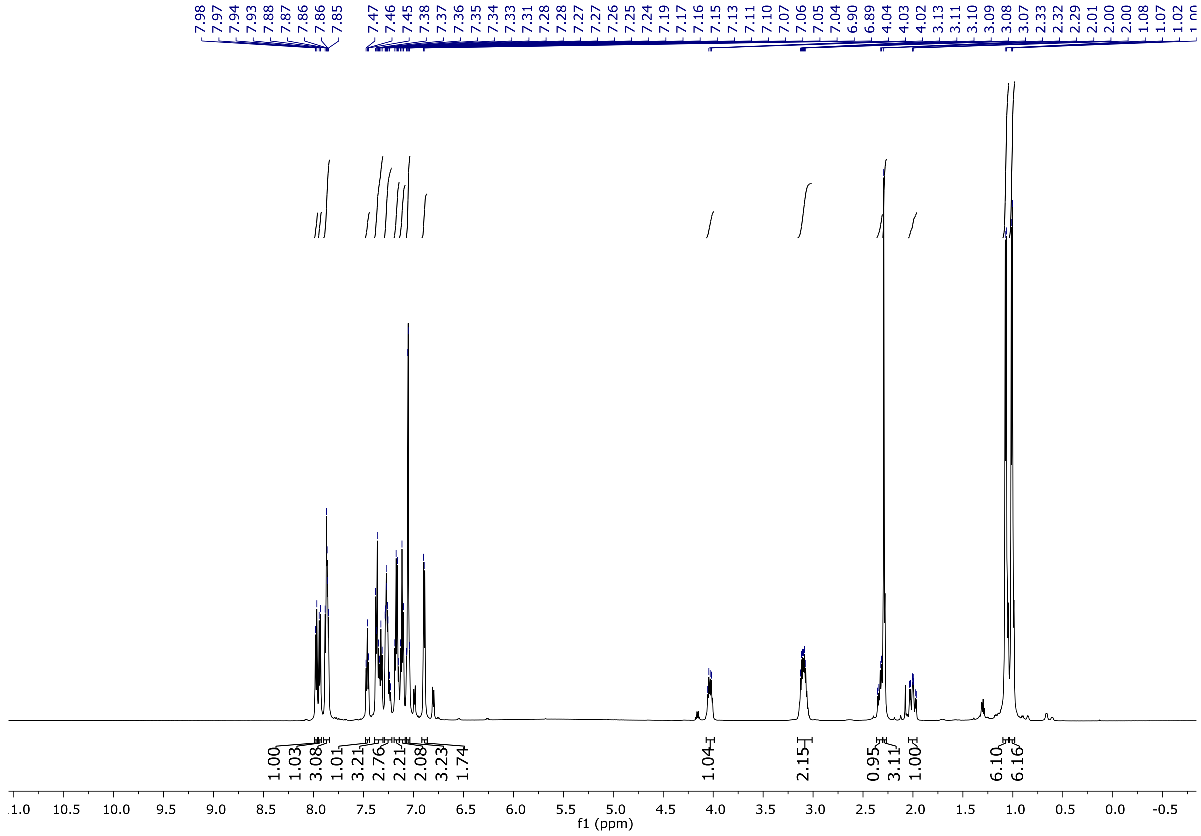

7.98
7.97
7.94
7.93
7.88
7.87
7.86
7.85
7.47
7.46
7.45
7.38
7.37
7.36
7.35
7.34
7.33
7.31
7.28
7.28
7.27
7.27
7.26
7.25
7.24
7.19
7.17
7.16
7.15
7.13
7.11
7.10
7.07
7.06
7.05
7.04
6.90
6.89
4.04
4.03
4.02
3.13
3.11
3.10
3.09
3.08
3.07
2.33
2.32
2.29
2.01
2.00
1.08
1.07
1.02
1.00
1.03
3.08
1.01
3.21
2.76
2.21
2.08
3.23
1.74
1.04
2.15
0.95
3.11
1.00
6.10
6.16
1.0
10.5
10.0
9.5
9.0
8.5
8.0
7.5
7.0
6.5
6.0
5.5
5.0
4.5
4.0
3.5
3.0
2.5
2.0
1.5
1.0
0.5
0.0
-0.5
f1 (ppm)

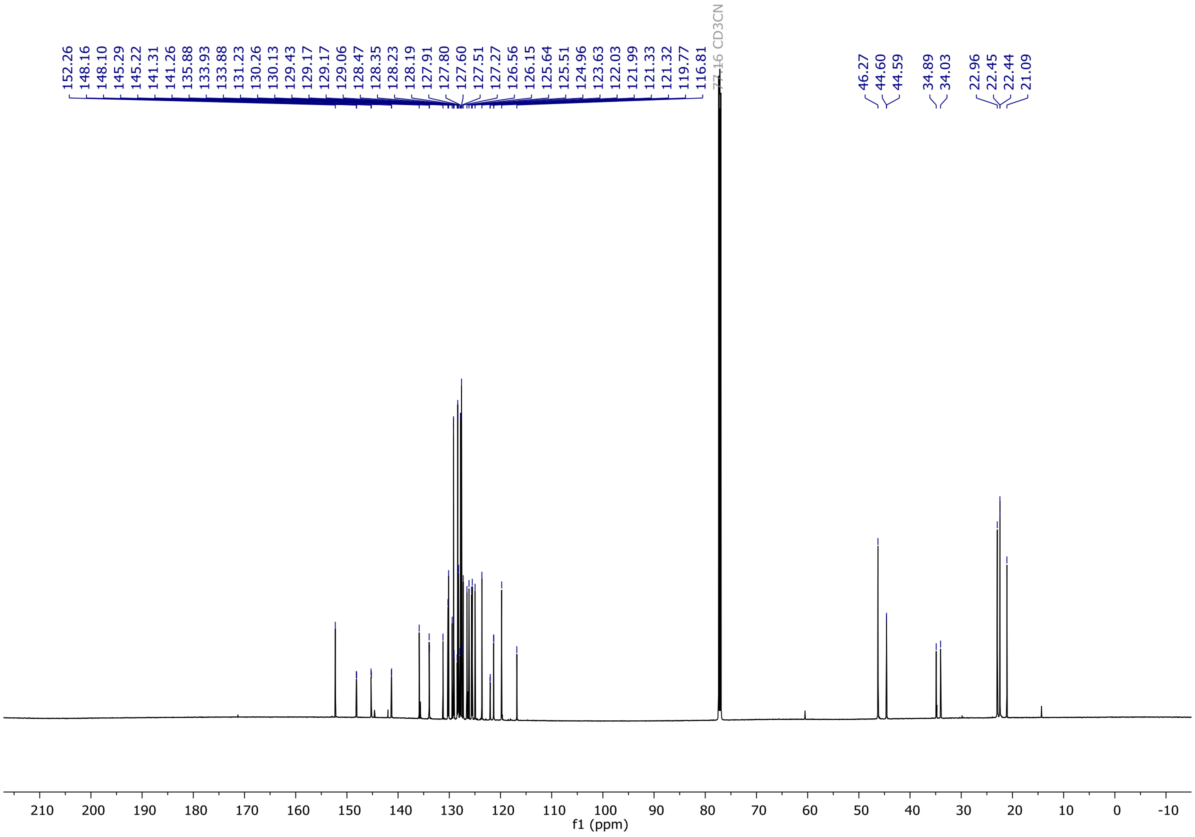
152.26
148.16
148.10
145.29
145.22
141.31
141.26
135.88
133.93
133.88
131.23
130.26
130.13
129.43
129.17
129.17
129.06
128.47
128.35
128.23
128.19
127.91
127.80
127.60
127.51
127.27
126.56
126.15
125.64
125.51
124.96
123.63
122.03
121.99
121.33
121.32
119.77
116.81
77.16 CD3CN
46.27
44.60
44.59
34.89
34.03
22.96
22.45
22.44
21.09
210 200 190 180 170 160 150 140 130 120 110 100 90 80 70 60 50 40 30 20 10 0 -10
f1 (ppm)

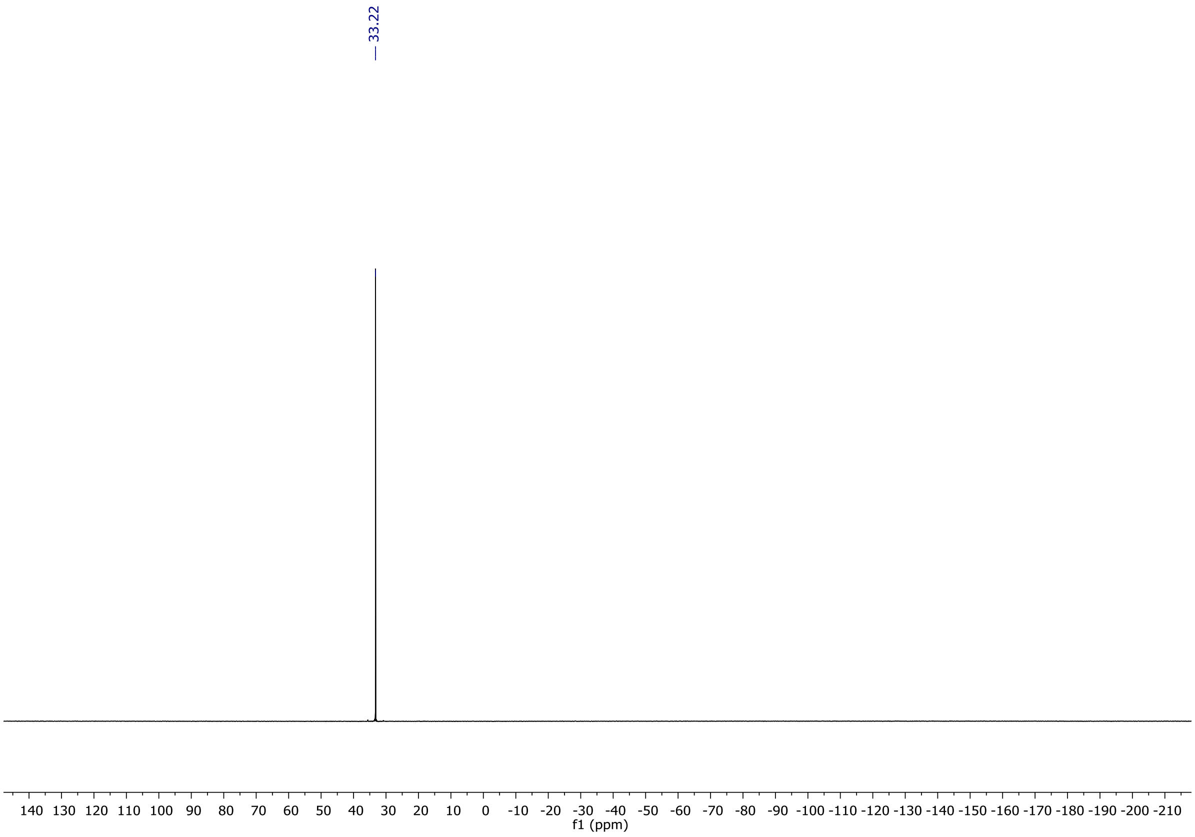
33.22
140 130 120 110 100 90 80 70 60 50 40 30 20 10 0 -10 -20 -30 -40 -50 -60 -70 -80 -90 -100 -110 -120 -130 -140 -150 -160 -170 -180 -190 -200 -210
f1 (ppm)

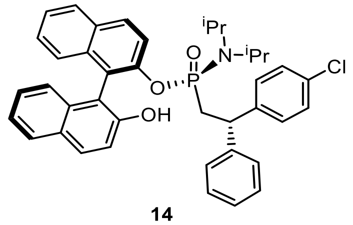
O
P
N
iPr
Cl
OH
O

14

^{1}H-NMR

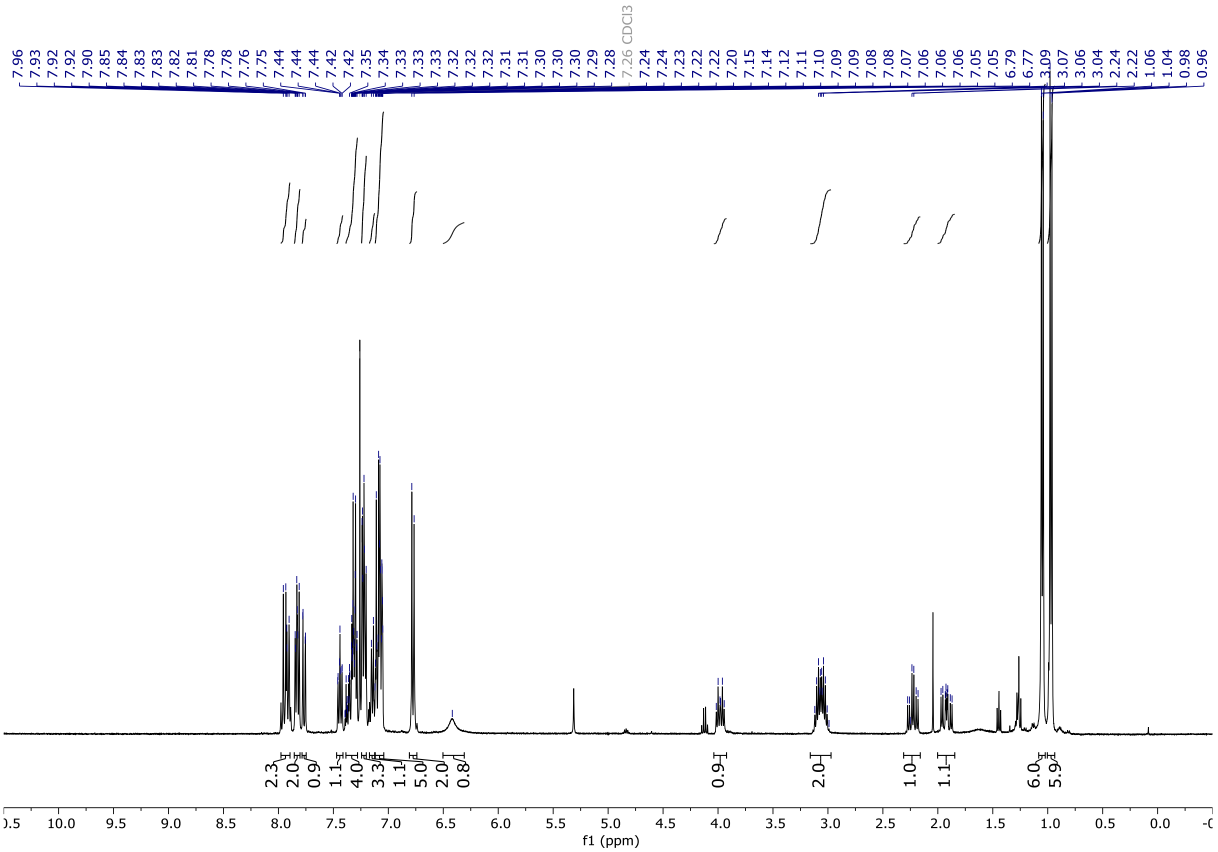
7.96 7.93 7.92 7.92 7.90 7.85 7.84 7.83 7.83 7.82 7.81 7.78 7.76 7.75 7.44 7.44 7.44 7.42 7.42 7.35 7.34 7.33 7.33 7.33 7.32 7.32 7.32 7.31 7.31 7.30 7.30 7.29 7.28 7.26 CDCl3 7.24 7.24 7.23 7.22 7.22 7.20 7.15 7.14 7.12 7.11 7.10 7.09 7.09 7.08 7.08 7.07 7.06 7.06 7.06 7.05 7.05 6.79 6.77 3.09 3.07 3.06 3.04 2.24 2.22 1.06 1.04 0.98 0.96
2.3 2.0 0.9 1.1 4.0 3.3 1.1 5.0 2.0 0.8 0.9 2.0 1.0 1.1 6.0 5.9
10.0 9.5 9.0 8.5 8.0 7.5 7.0 6.5 6.0 5.5 5.0 4.5 4.0 3.5 3.0 2.5 2.0 1.5 1.0 0.5 0.0
f1 (ppm)

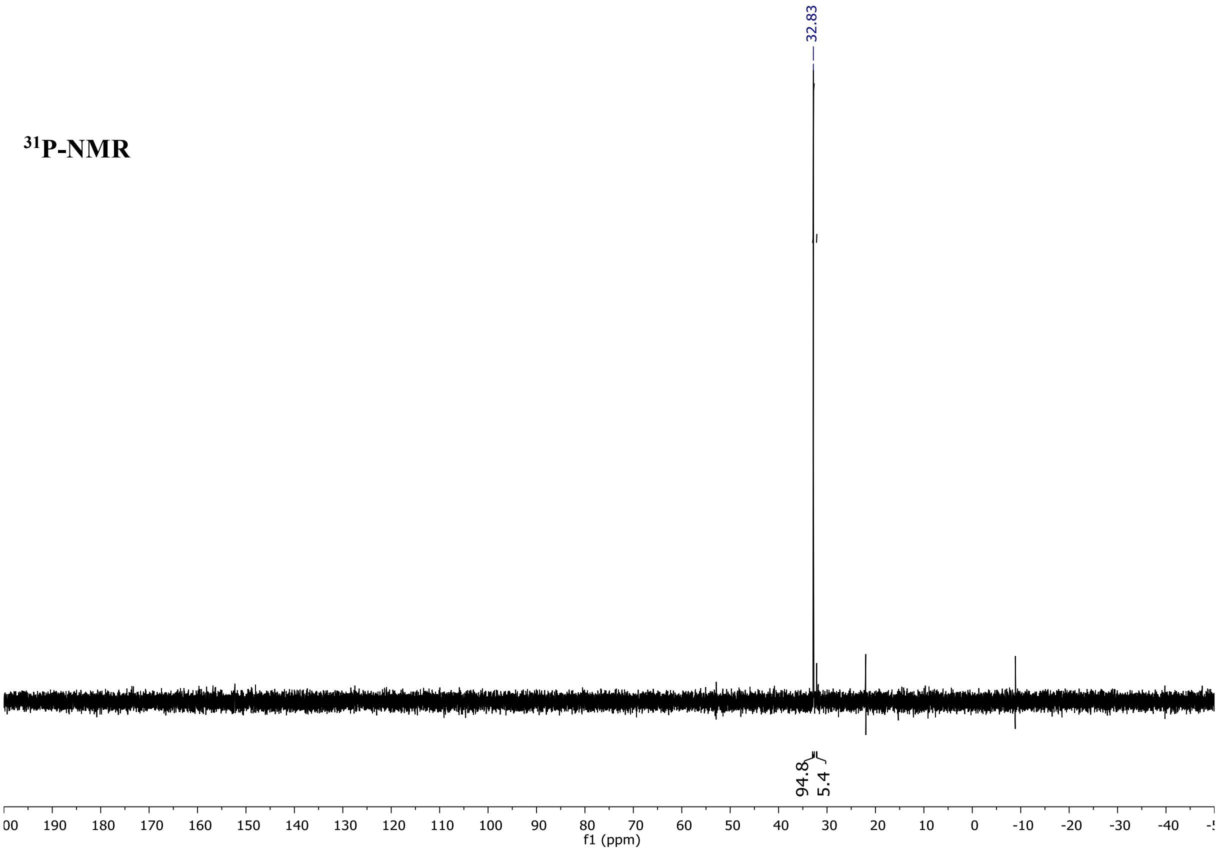
32.83
³¹P-NMR
94.8
5.4
f1 (ppm)

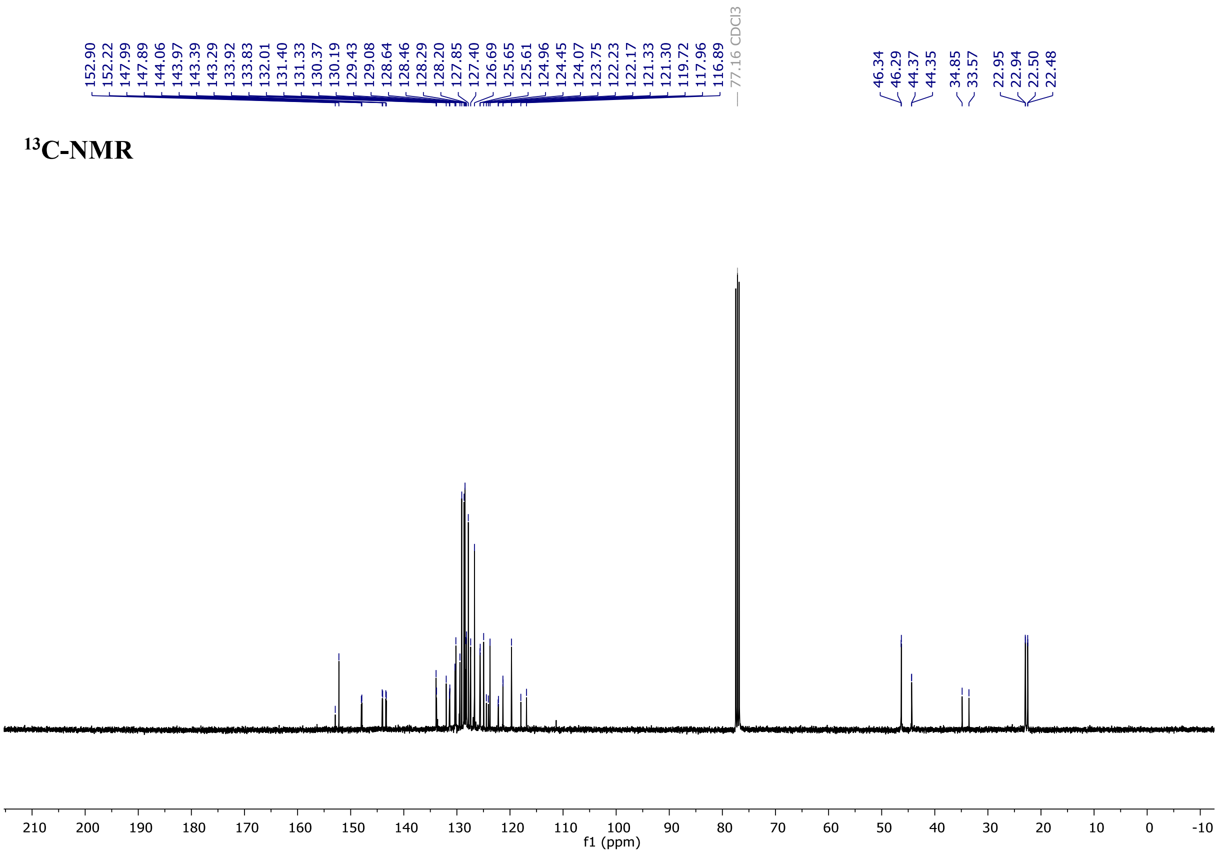
152.90
152.22
147.99
147.89
144.06
143.97
143.39
143.29
133.92
133.83
132.01
131.40
131.33
130.37
130.19
129.43
129.08
128.64
128.46
128.29
128.20
127.85
127.40
126.69
126.65
125.61
124.96
124.45
124.07
123.75
122.23
122.17
121.33
121.30
119.72
117.96
116.89
77.16 CDCl3
46.34
46.29
44.37
44.35
34.85
33.57
22.95
22.94
22.50
22.48
¹³C-NMR
f1 (ppm)

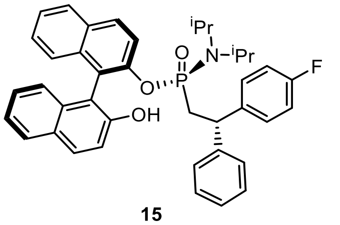

15

^{1}H-NMR

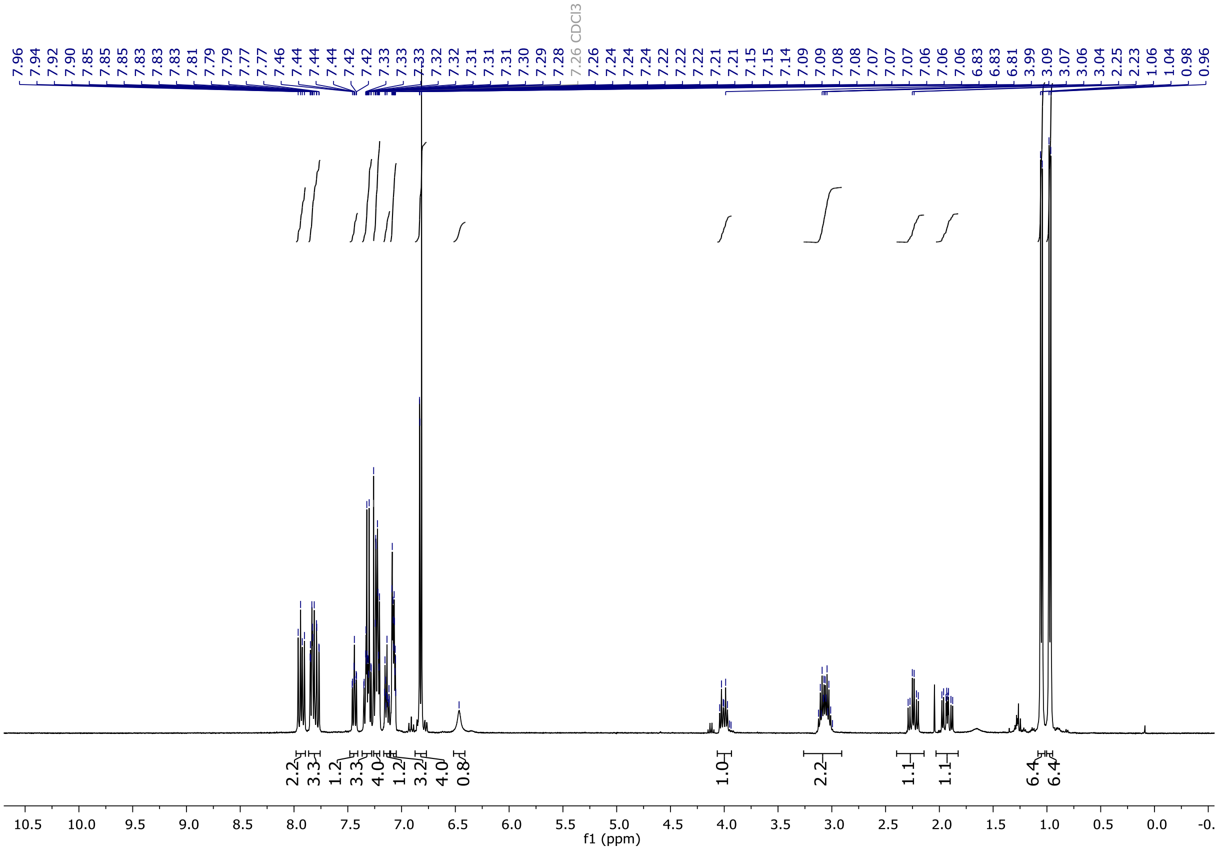

^{31}P-NMR

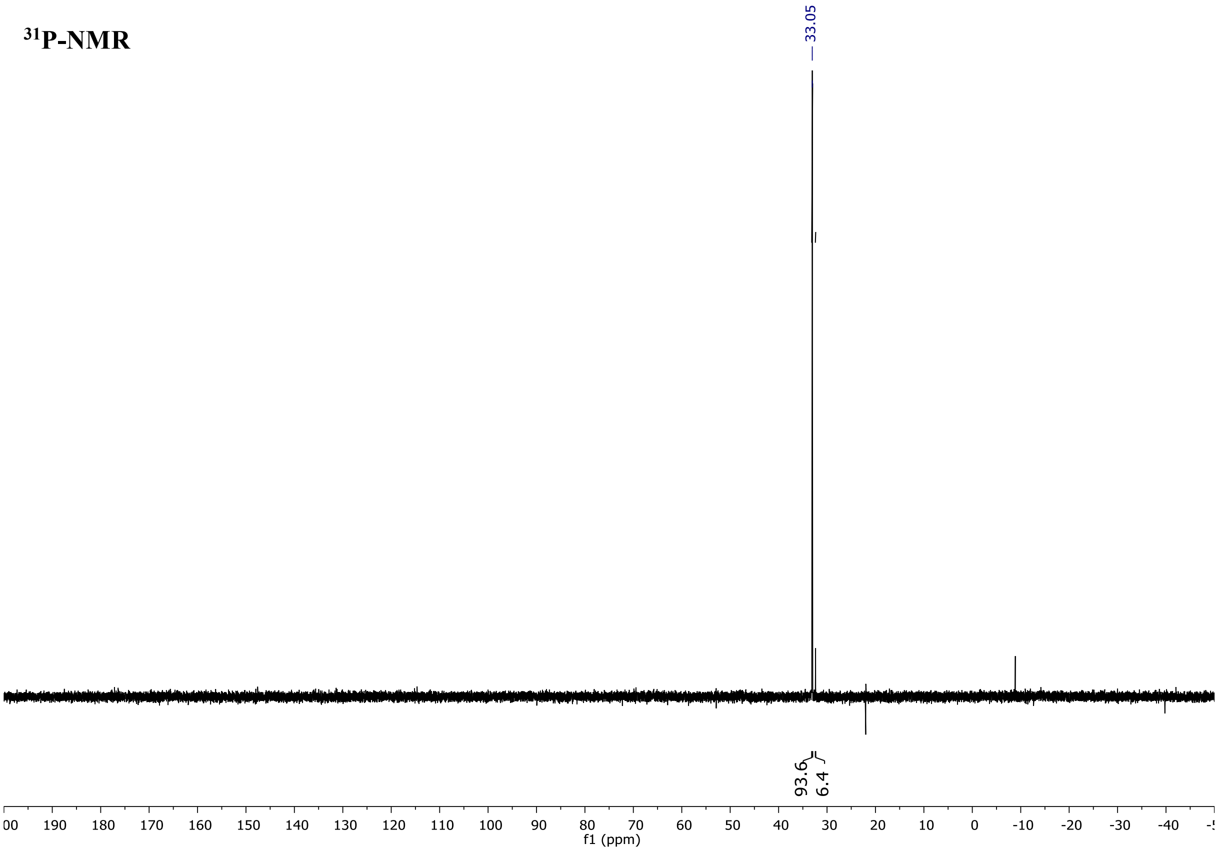

^{19}F-NMR

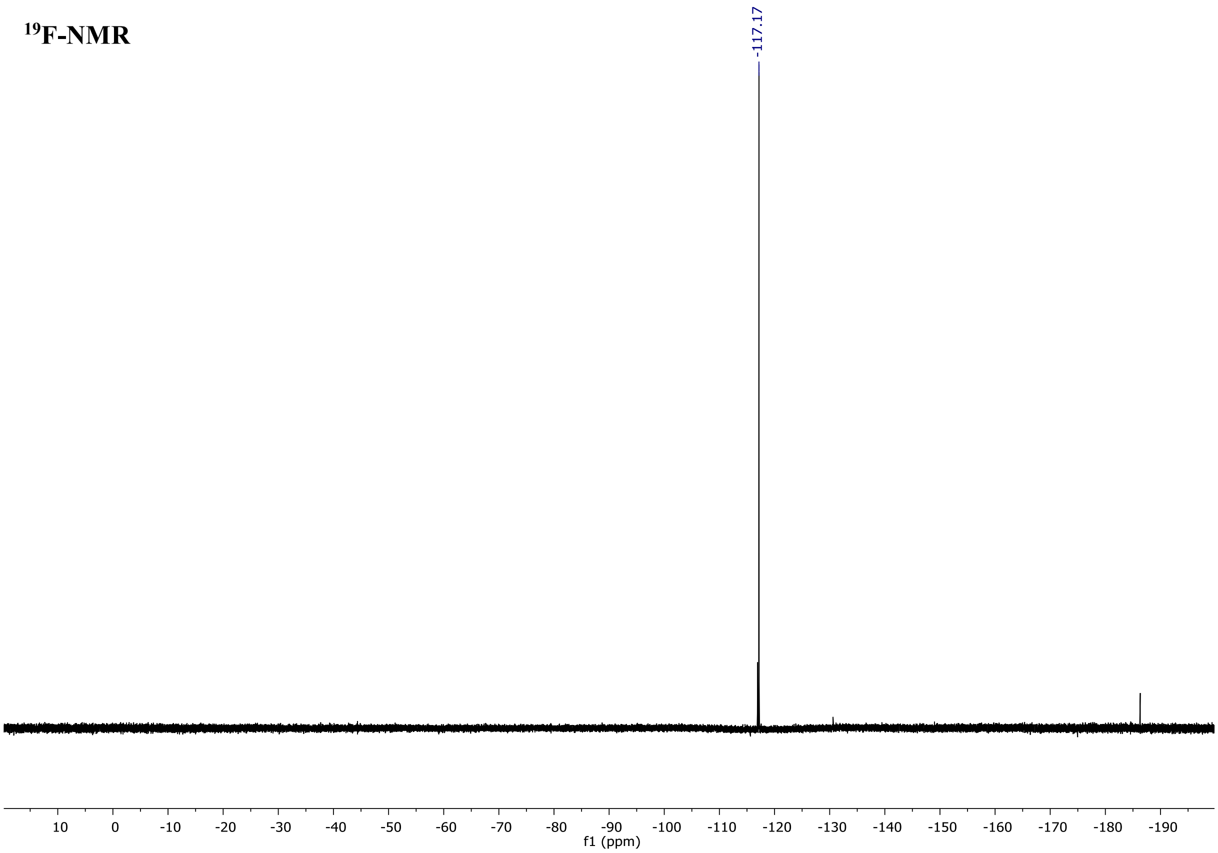

^{13}C-NMR

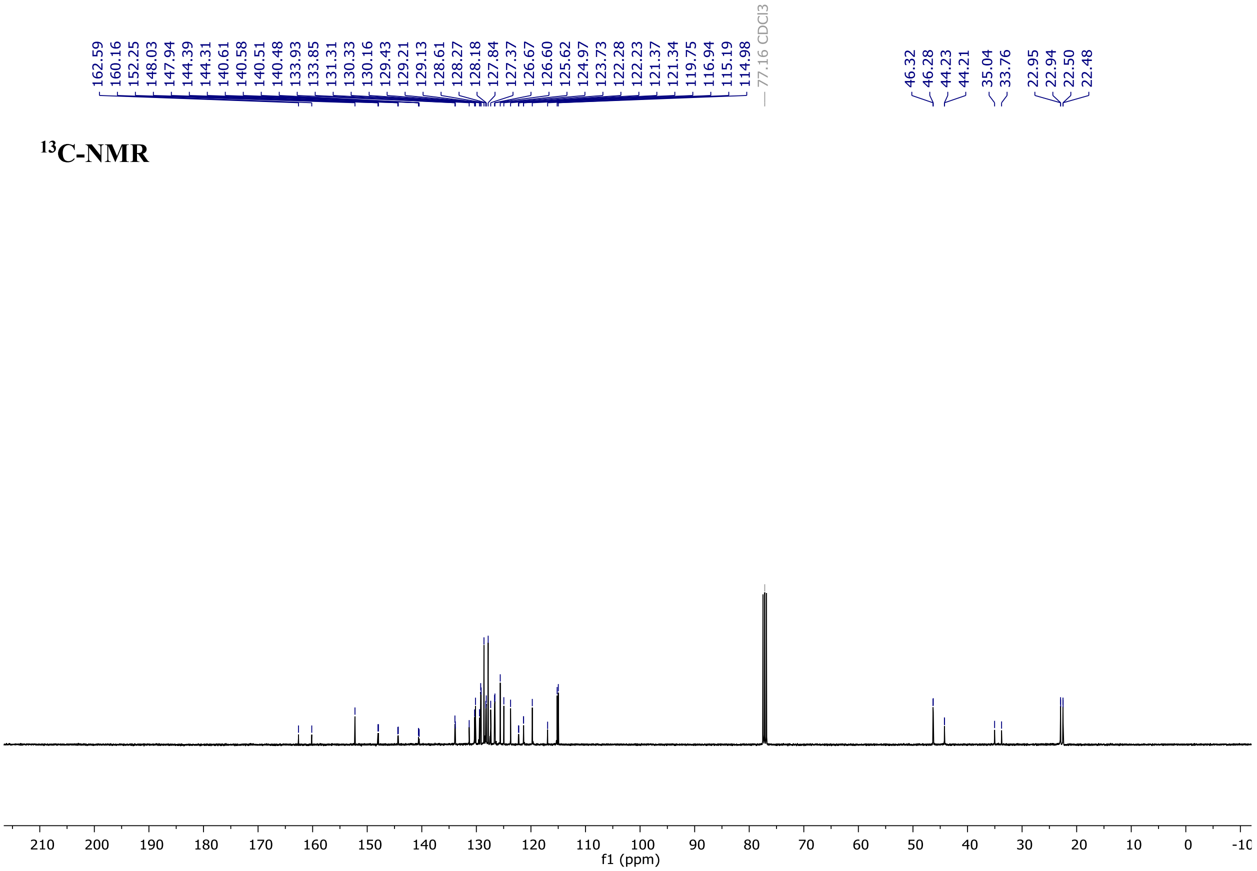

162.59
160.16
152.25
148.03
147.94
144.39
144.31
140.61
140.58
140.51
140.48
133.93
133.85
131.31
130.33
130.16
129.43
129.21
129.13
128.61
128.27
128.18
127.84
127.37
126.67
126.60
125.62
124.97
123.73
122.28
122.23
121.37
121.34
119.75
116.94
115.19
114.98
77.16 CDCl3
46.32
46.28
44.23
44.21
35.04
33.76
22.95
22.94
22.50
22.48
210
200
190
180
170
160
150
140
130
120
110
100
90
80
70
60
50
40
30
20
10
0
-10
f1 (ppm)

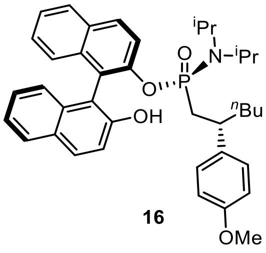

16

^{1}H-NMR

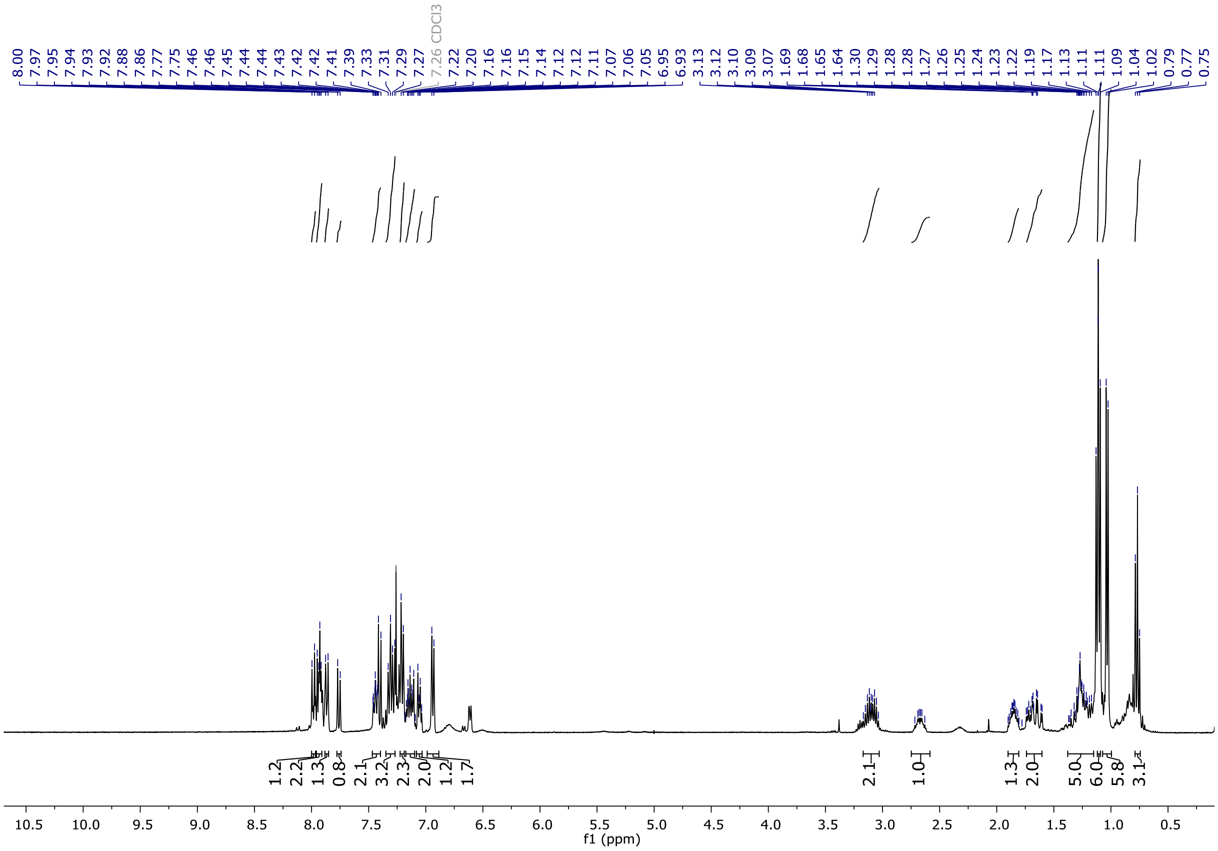

^{31}P-NMR

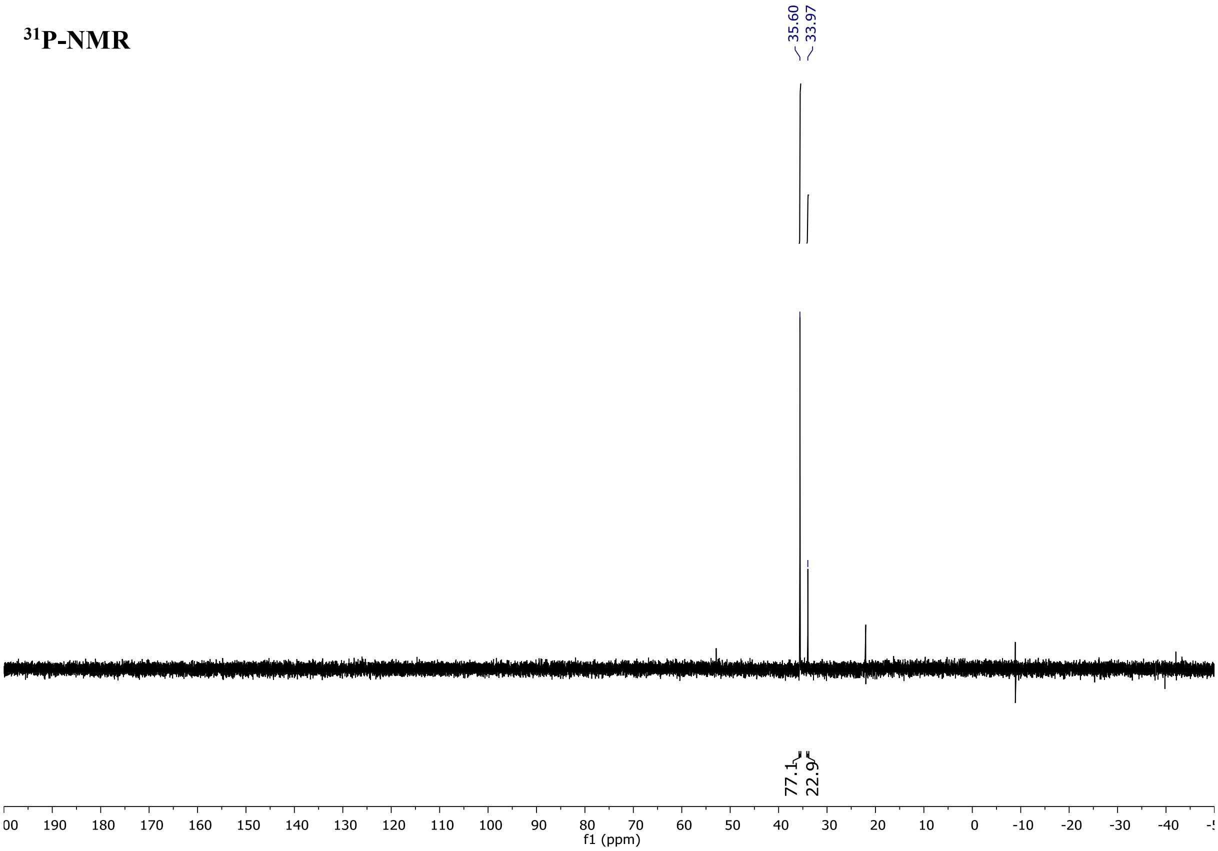

^{13}C-NMR

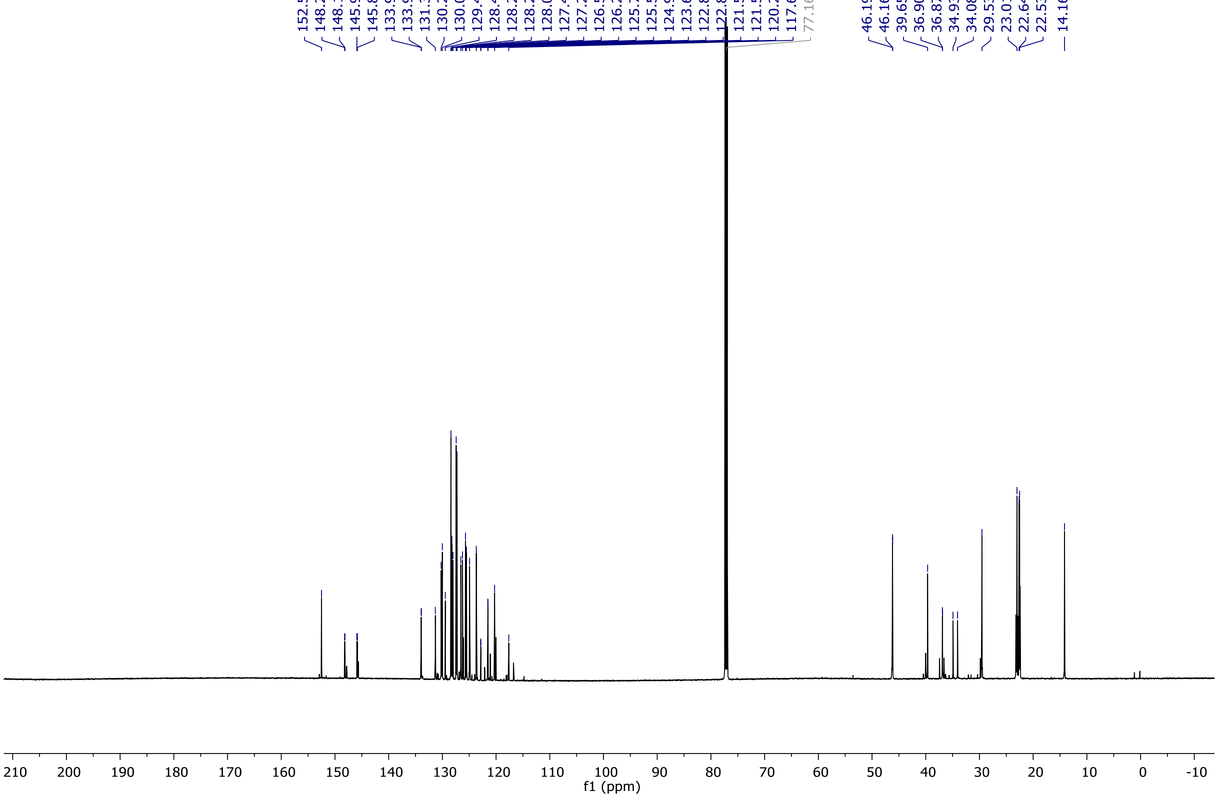

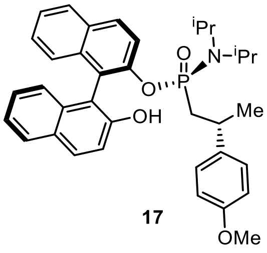

17

^{1}H-NMR

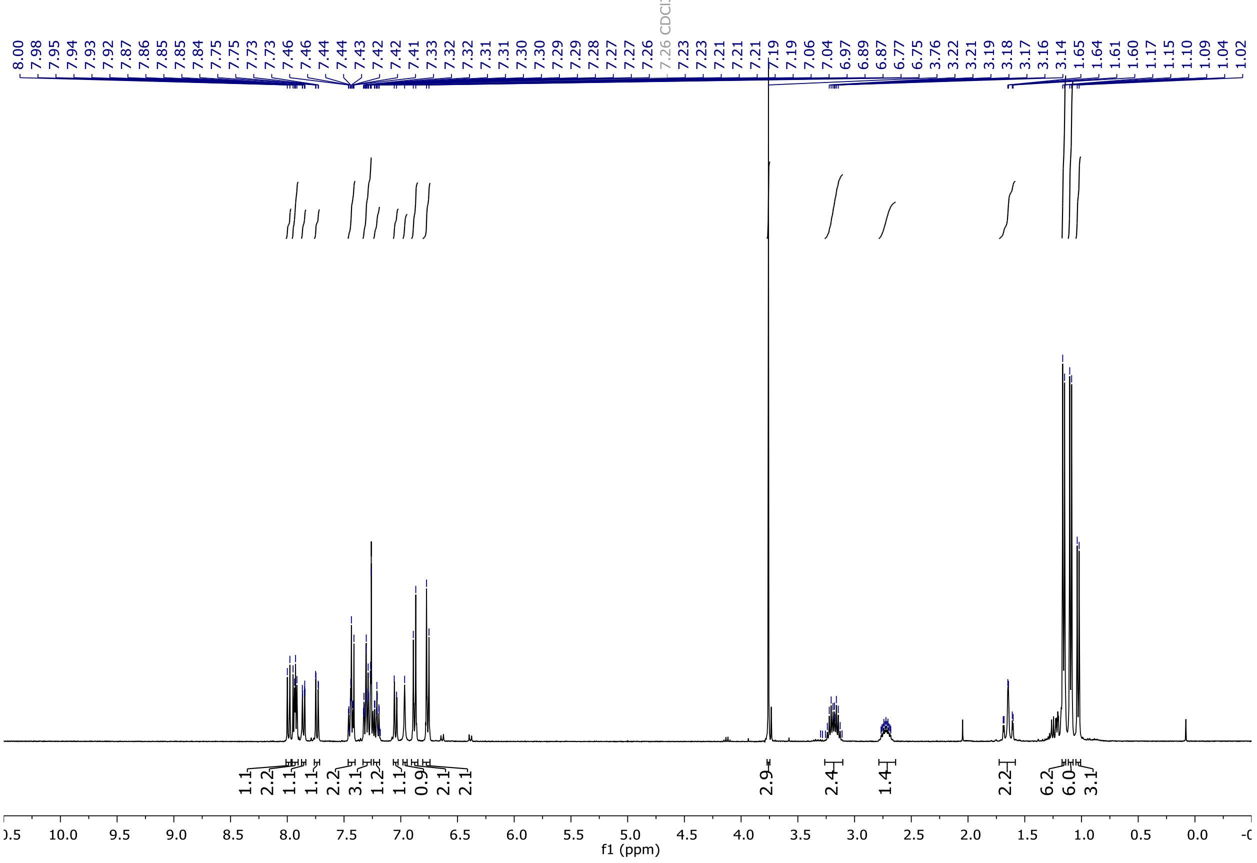

^{31}P-NMR

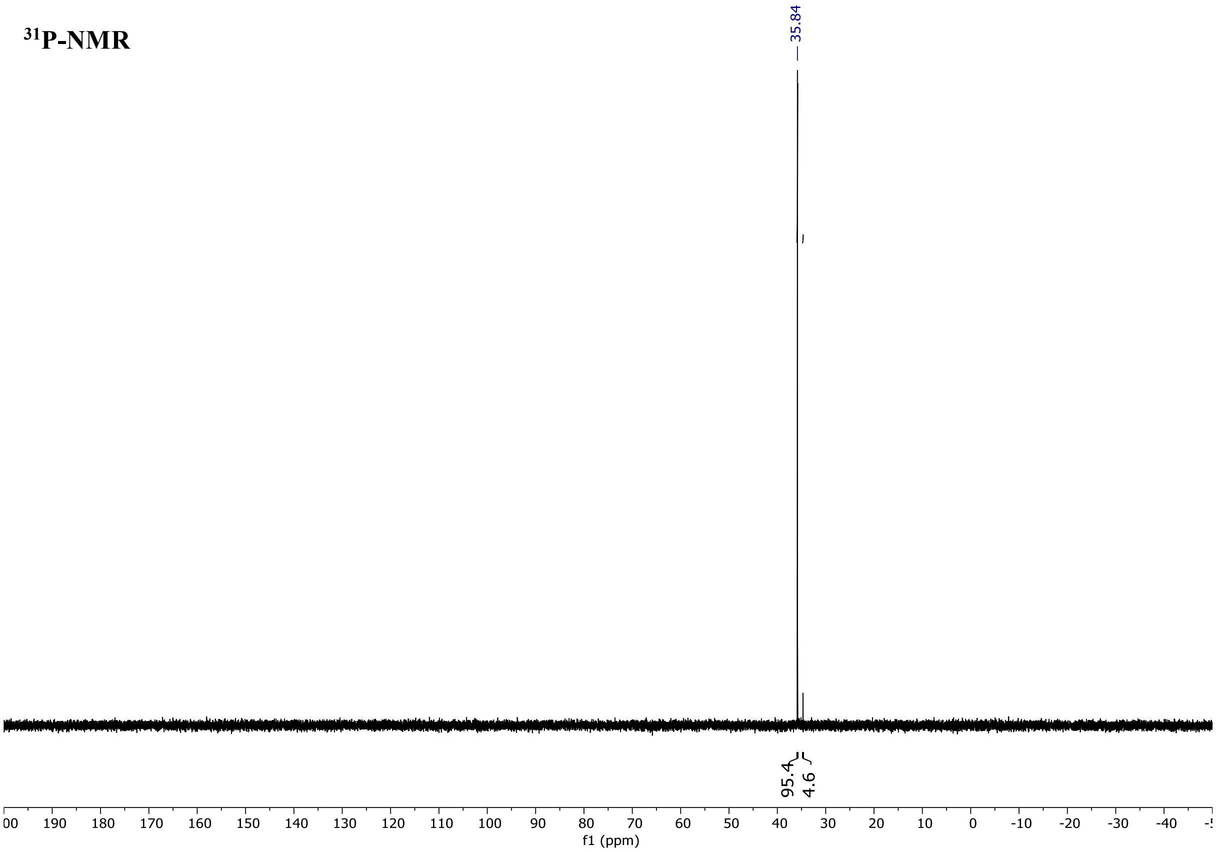

^{13}C-NMR

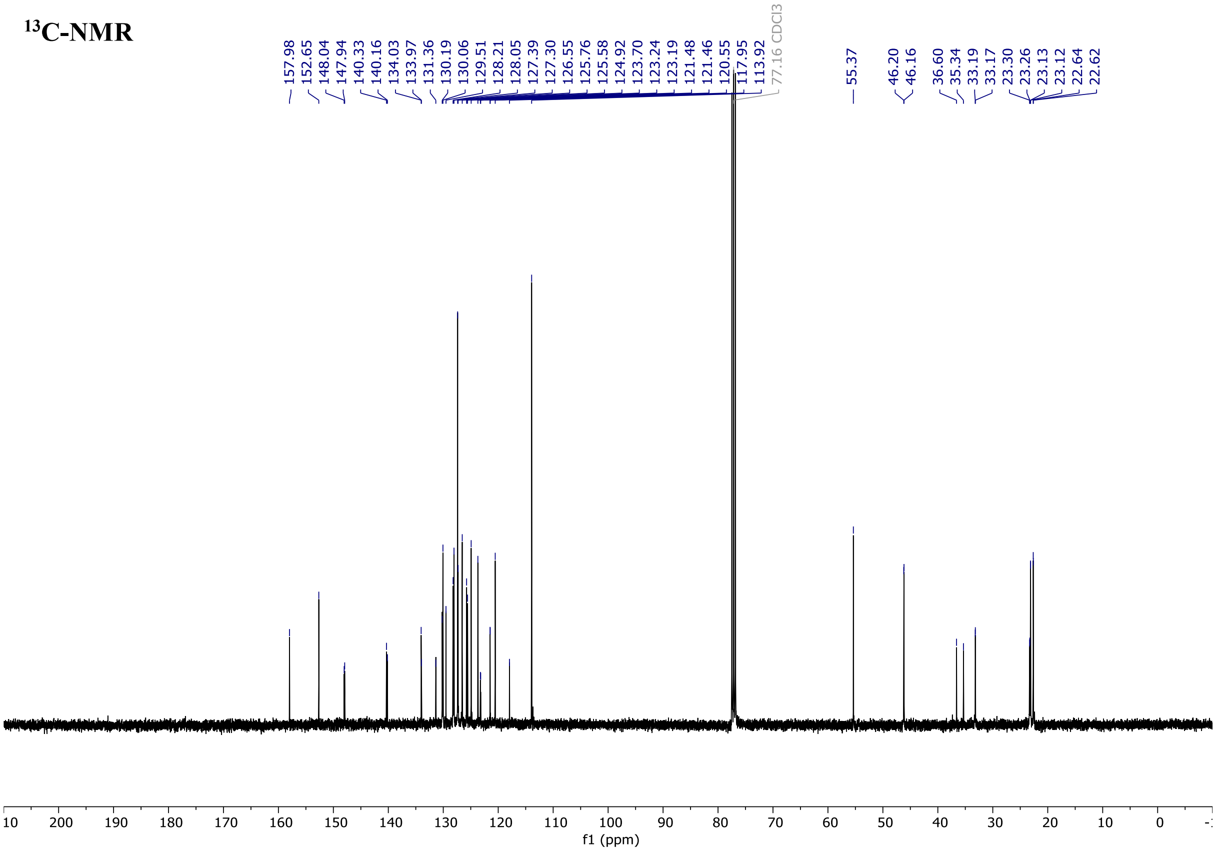

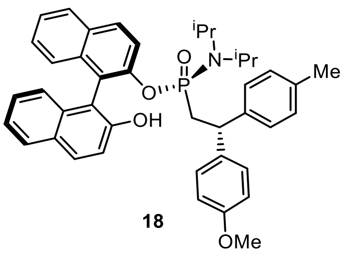

18

^{1}H-NMR

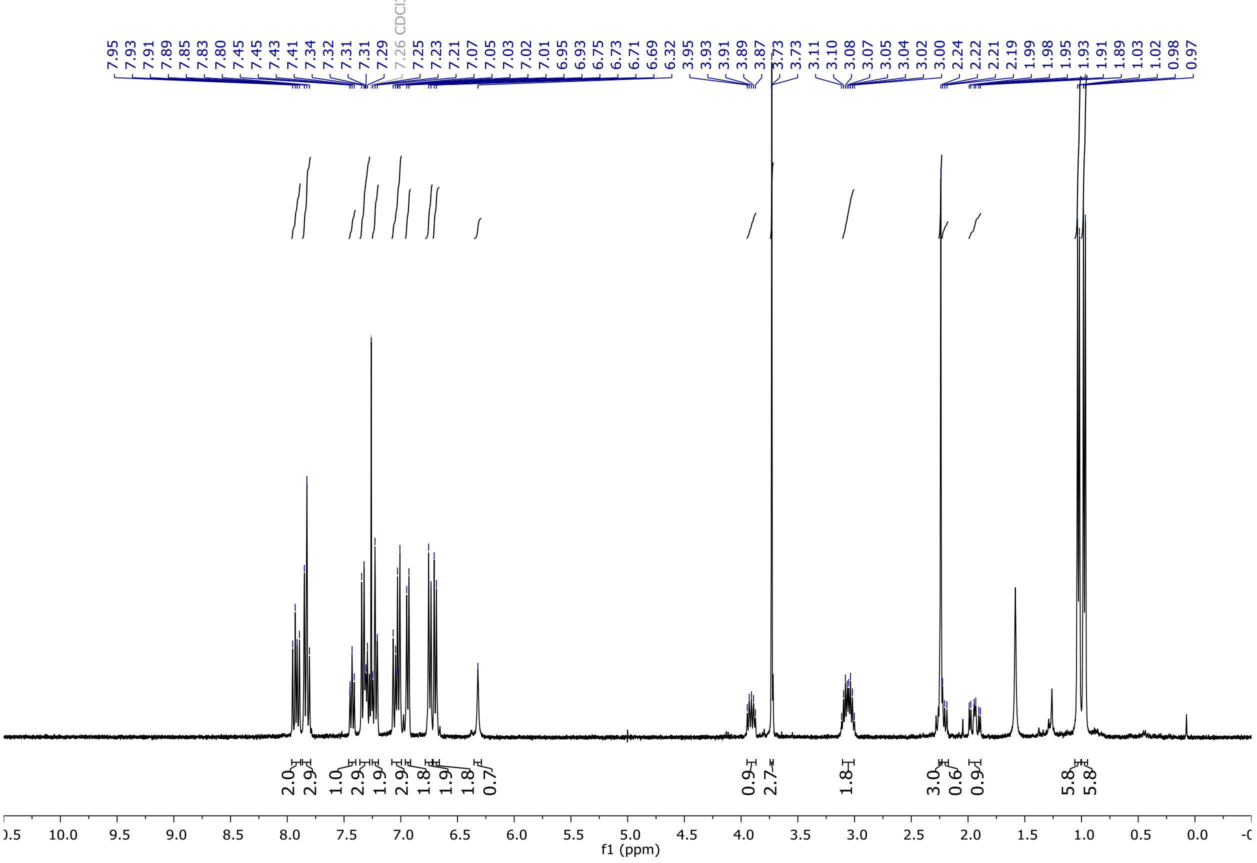

^{31}P-NMR

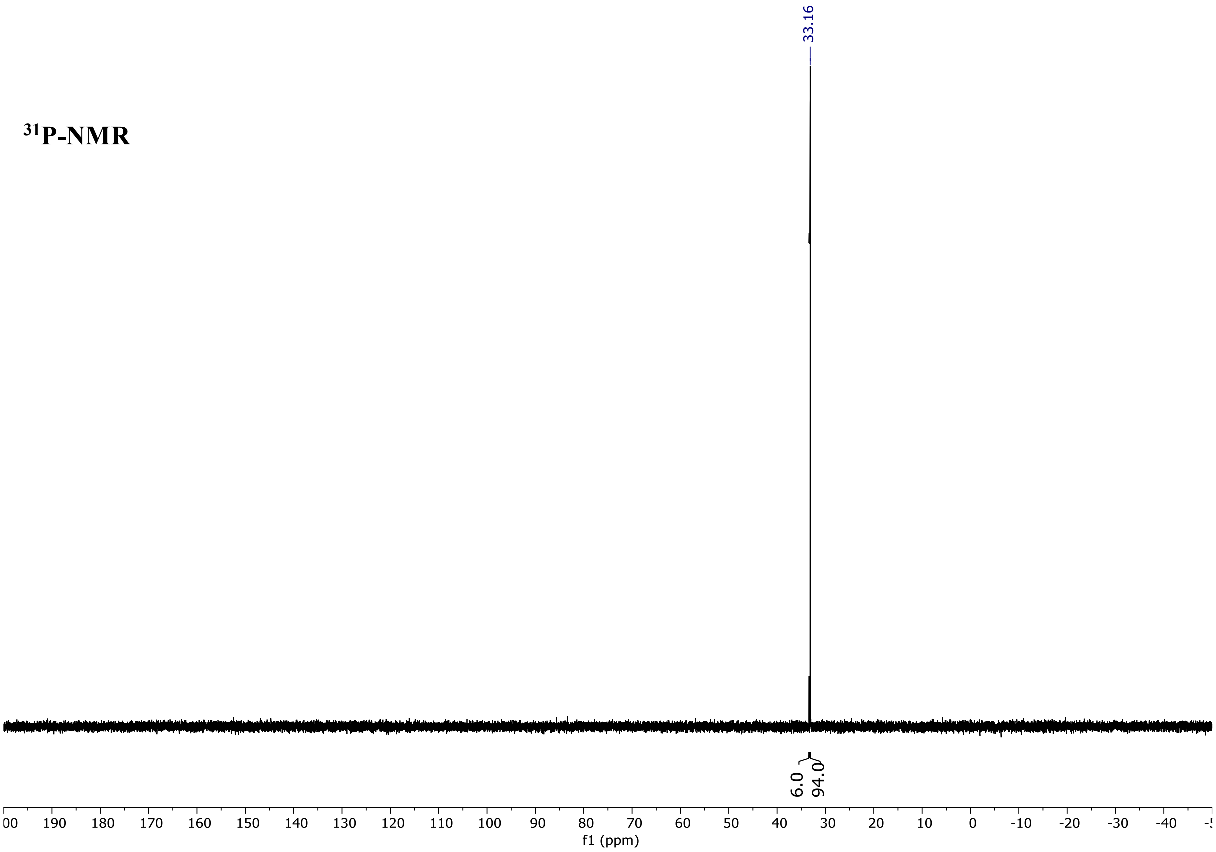

^{13}C-NMR

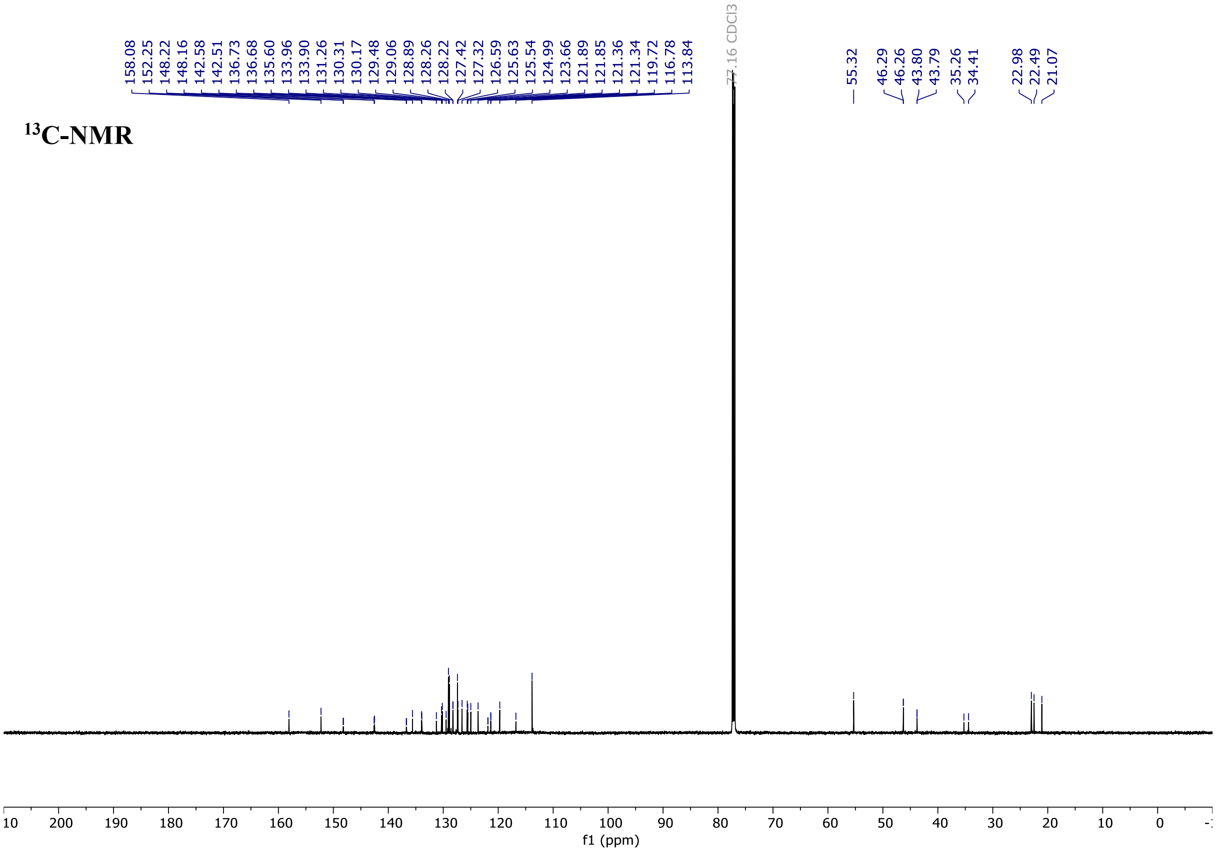

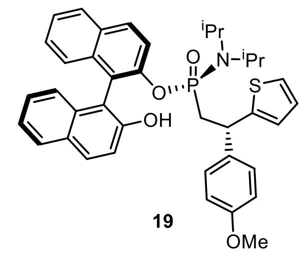

iPr
N–iPr
O
P
S
OH
OMe

19

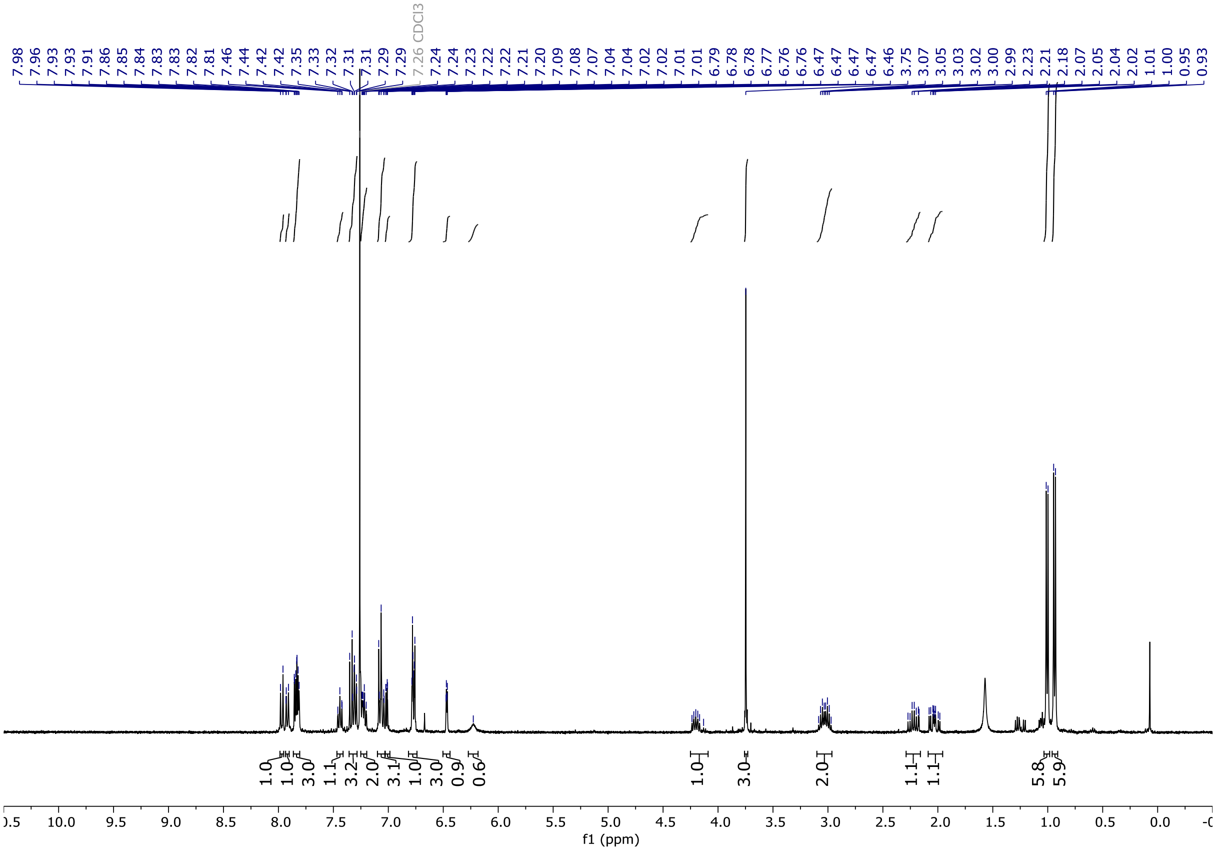

7.98 7.96 7.93 7.93 7.91 7.86 7.85 7.84 7.83 7.83 7.82 7.81 7.46 7.44 7.42 7.42 7.35 7.33 7.32 7.31 7.31 7.29 7.29 7.26 CDCl3 7.24 7.24 7.23 7.22 7.22 7.21 7.20 7.09 7.08 7.07 7.04 7.04 7.02 7.02 7.01 7.01 6.79 6.78 6.78 6.77 6.76 6.76 6.47 6.47 6.47 6.47 6.46 3.75 3.07 3.05 3.03 3.02 3.00 2.99 2.23 2.21 2.18 2.07 2.05 2.04 2.02 1.01 1.00 0.95 0.93
1.0 1.0 3.0 1.1 3.2 2.0 3.1 1.0 3.0 0.9 0.6
1.0 3.0 2.0 1.1 1.1 5.8 5.9
10.5 10.0 9.5 9.0 8.5 8.0 7.5 7.0 6.5 6.0 5.5 5.0 4.5 4.0 3.5 3.0 2.5 2.0 1.5 1.0 0.5 0.0
f1 (ppm)

^{31}P-NMR

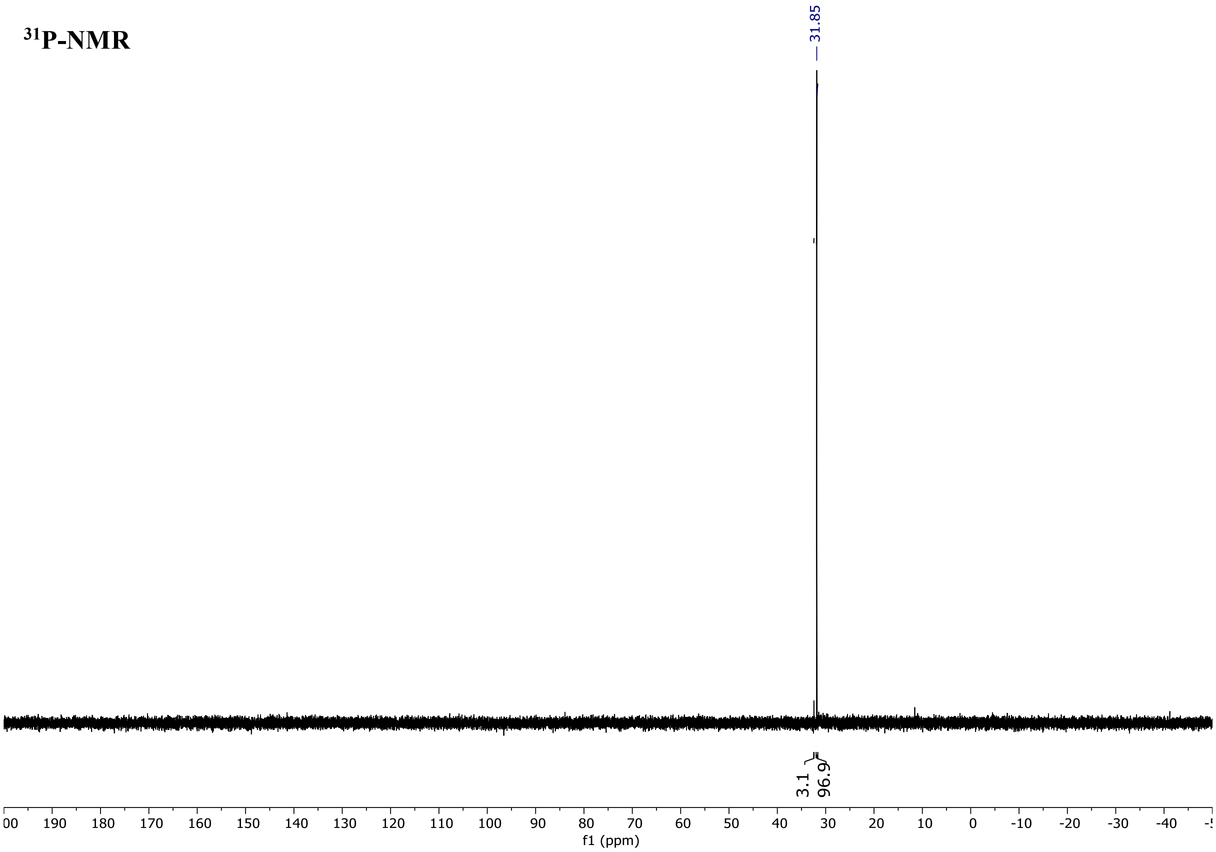

^{13}C-NMR

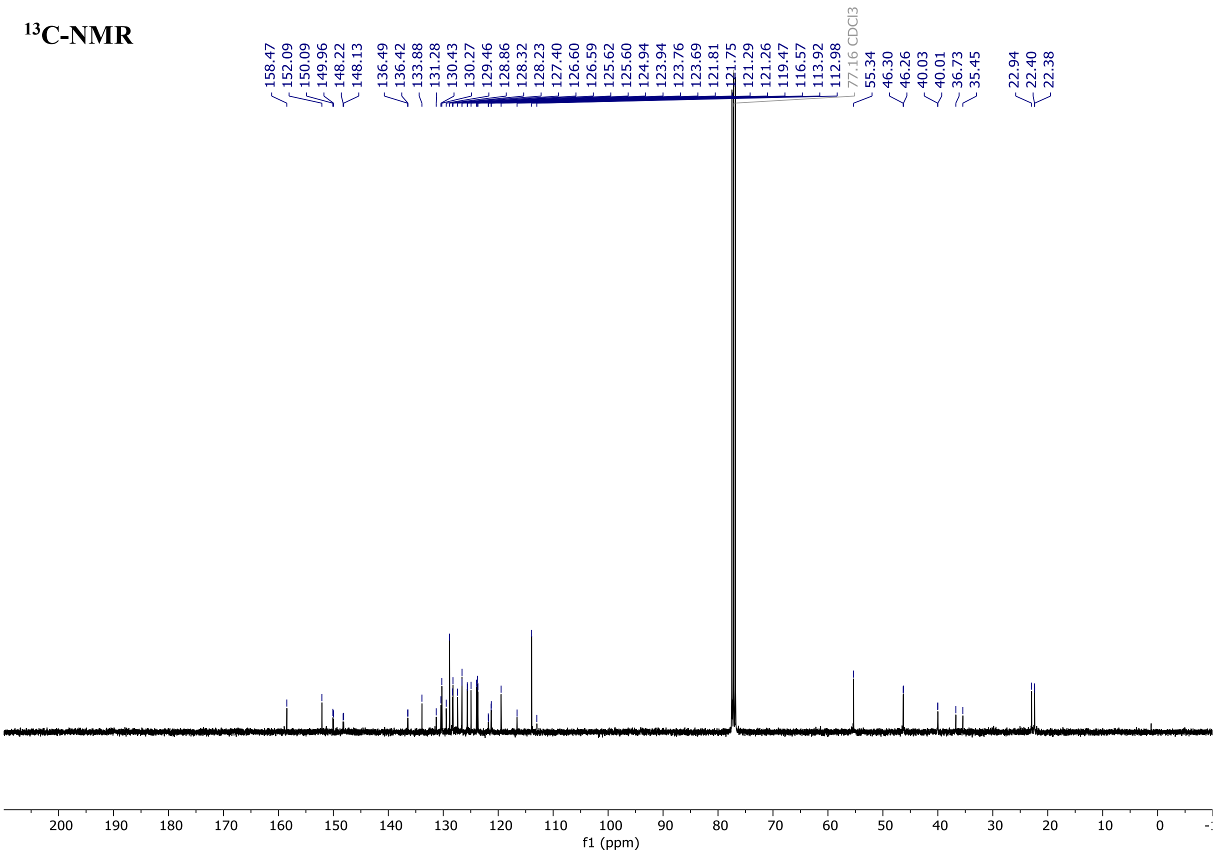

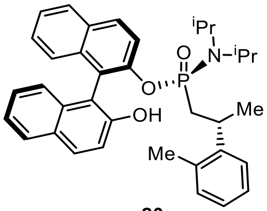

20

^{1}H-NMR

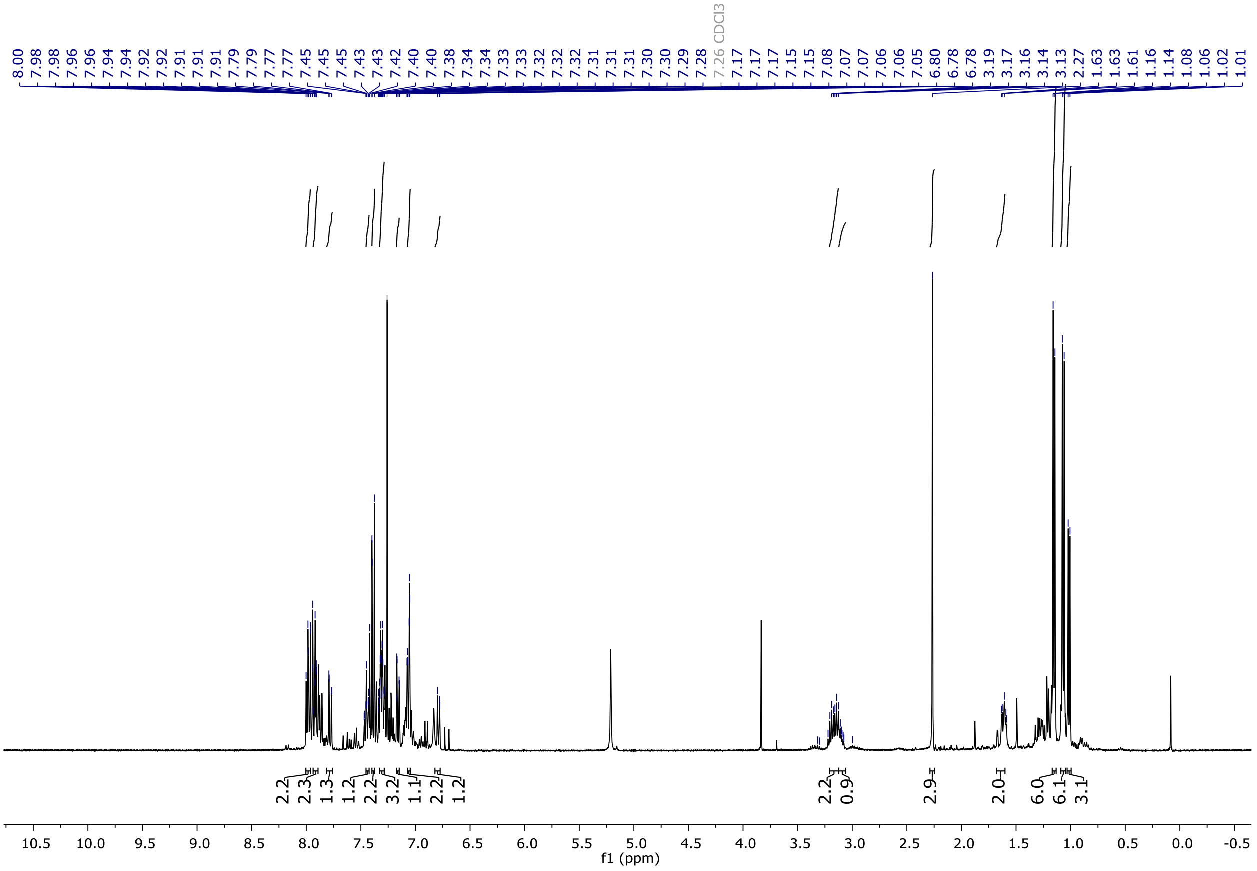

^{31}P-NMR

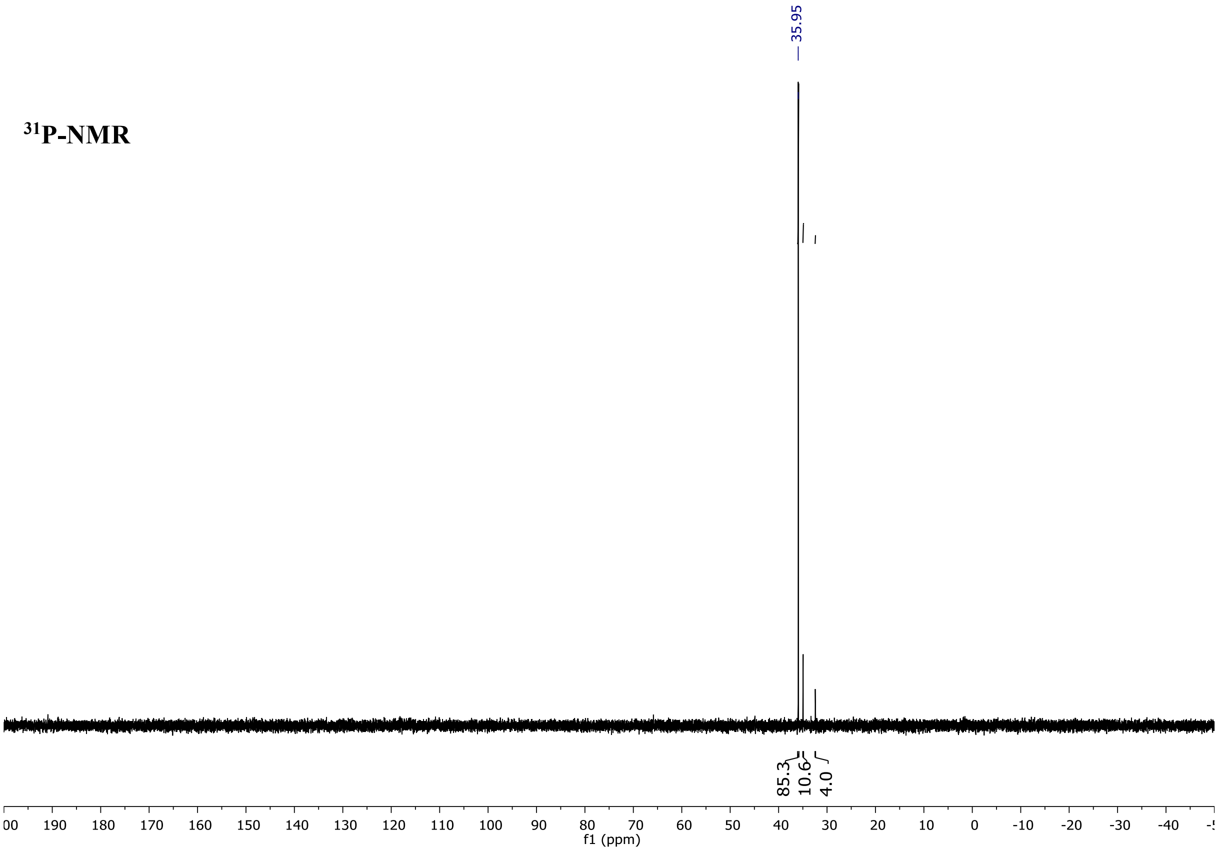

^{13}C-NMR

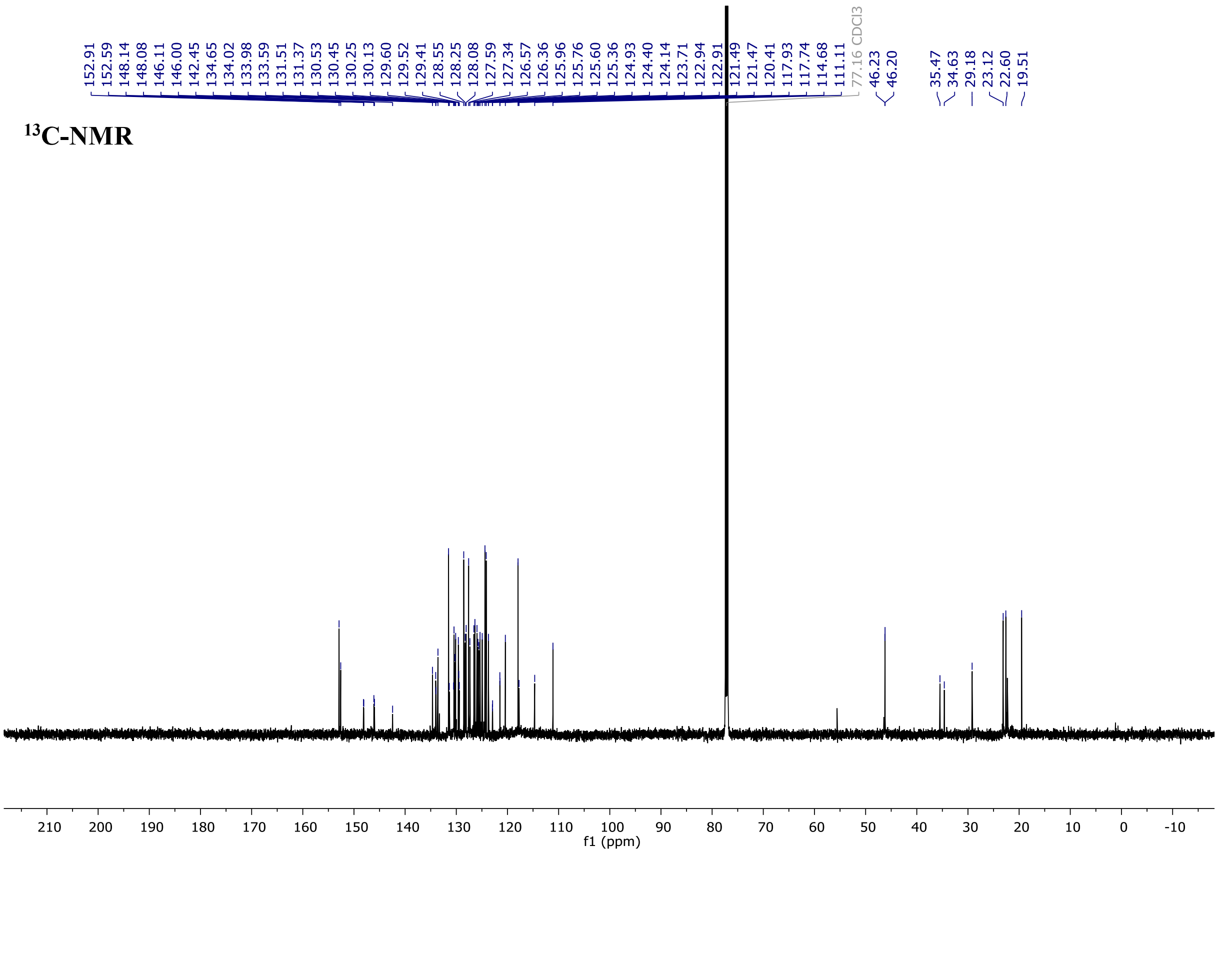

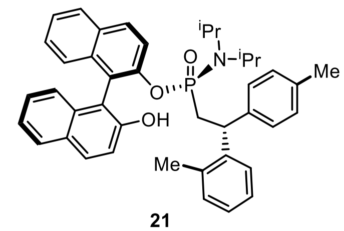

iPr
N
iPr
O
P
O
Me
OH
Me

21

^{1}H-NMR

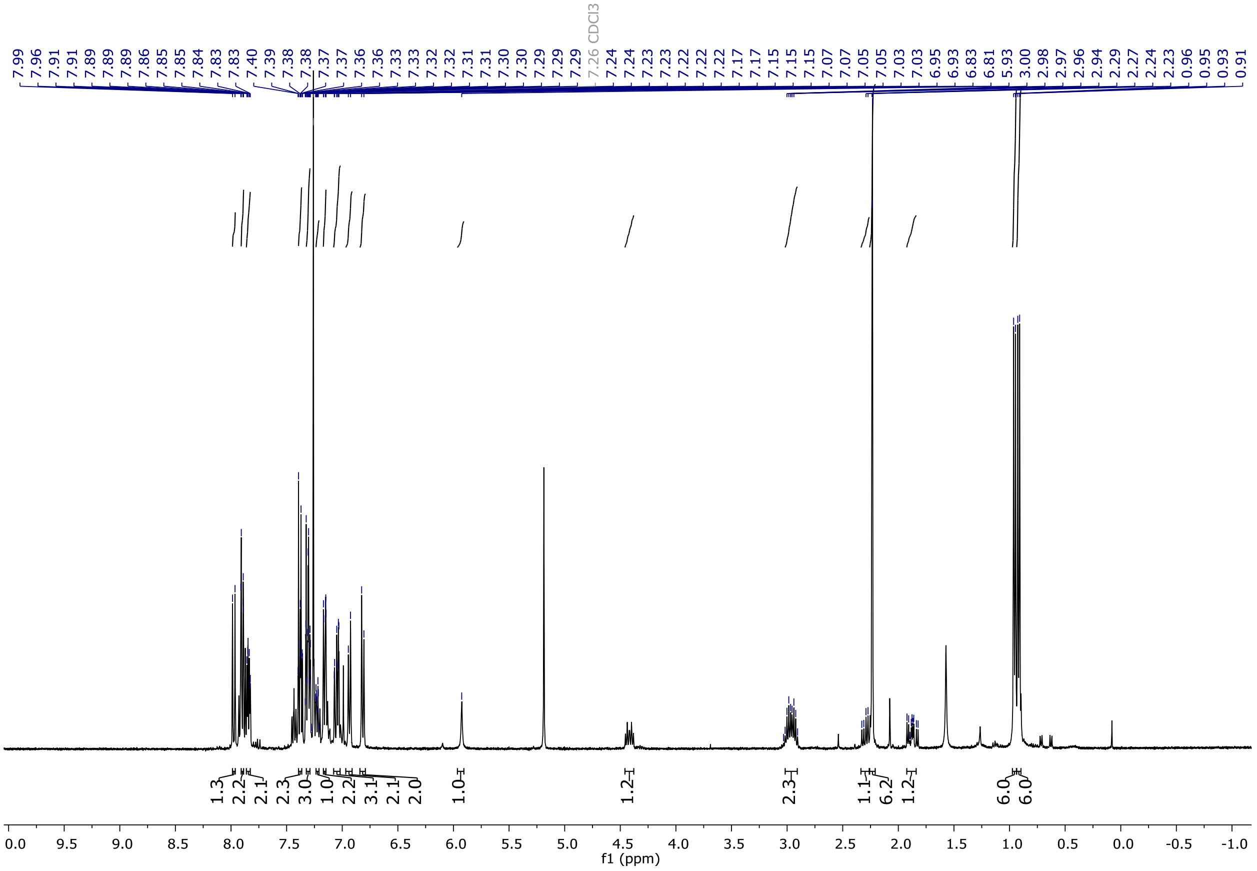

7.99 7.96 7.91 7.89 7.89 7.89 7.86 7.85 7.85 7.84 7.83 7.83 7.40 7.39 7.38 7.38 7.37 7.37 7.36 7.36 7.33 7.33 7.32 7.32 7.31 7.31 7.30 7.30 7.29 7.29 7.29
7.26 CDCl3
7.24 7.24 7.23 7.23 7.22 7.22 7.22 7.17 7.17 7.15 7.15 7.15 7.07 7.07 7.05 7.05 7.05 7.03 7.03 6.95 6.93 6.83 6.81 5.93 3.00 2.98 2.97 2.96 2.94 2.29 2.27 2.24 2.24 2.23 0.96 0.95 0.93 0.91
1.3 2.2 2.1 2.3 3.0 1.0 2.2 3.1 2.1 2.0 1.0 1.2 2.3 1.1 6.2 1.2 6.0 6.0
0.0 9.5 9.0 8.5 8.0 7.5 7.0 6.5 6.0 5.5 5.0 4.5 4.0 3.5 3.0 2.5 2.0 1.5 1.0 0.5 0.0 -0.5 -1.0
f1 (ppm)

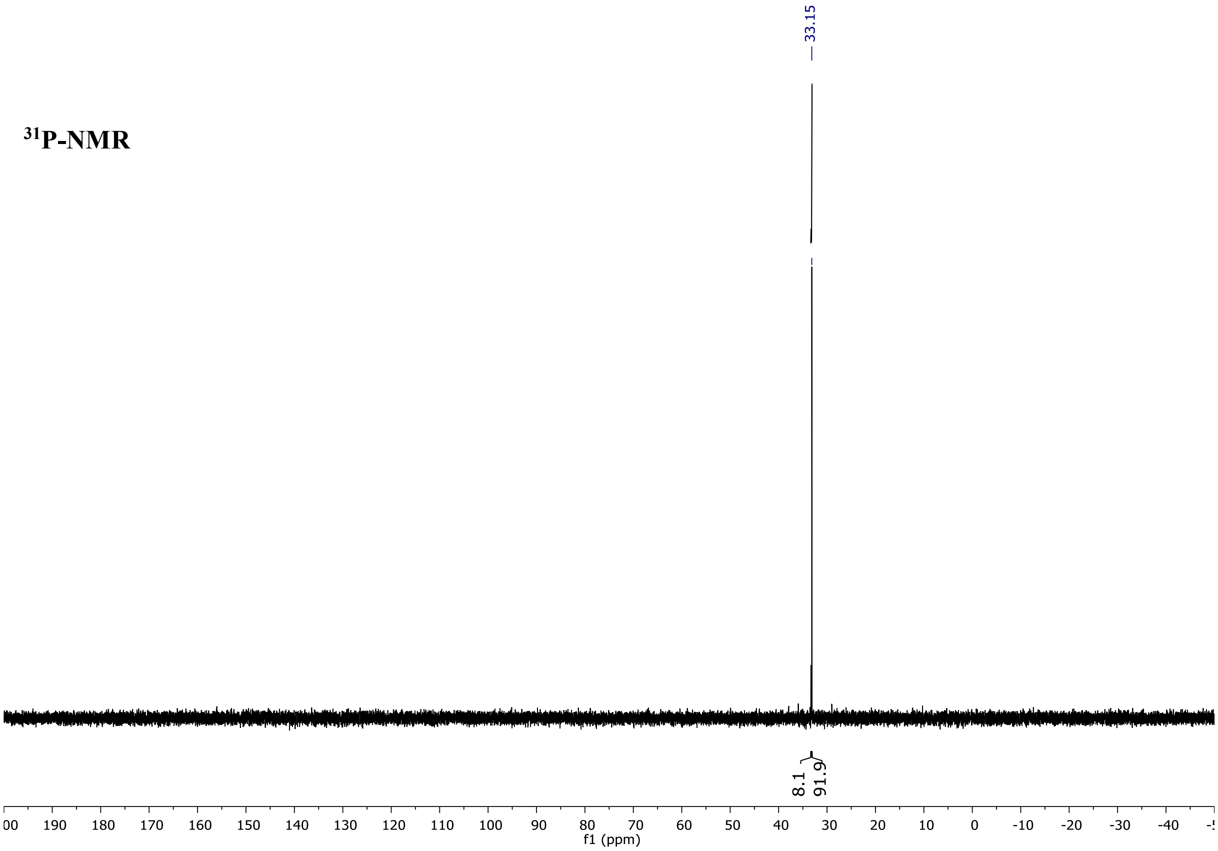

^{31}P-NMR
33.15
8.1
91.9
f1 (ppm)

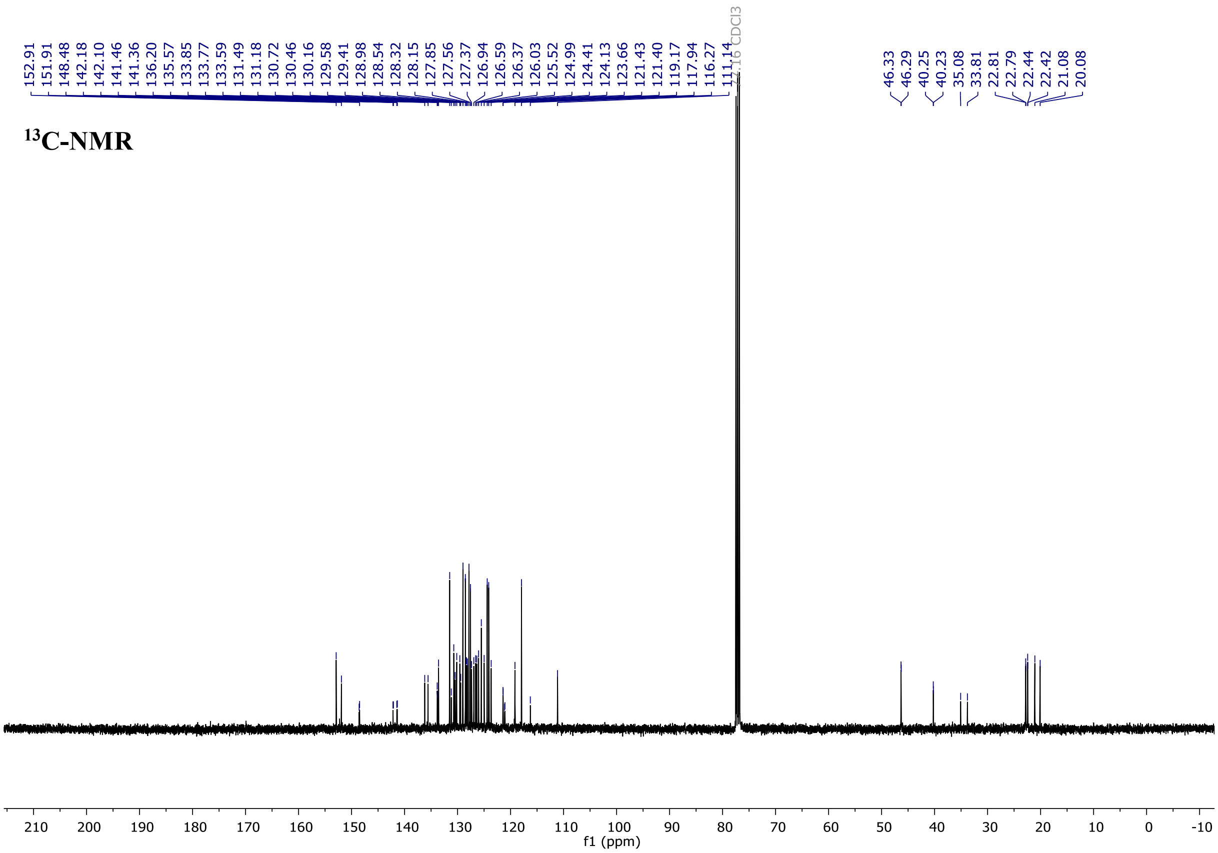

^{13}C-NMR
152.91
151.91
148.48
142.18
142.10
141.46
141.36
136.20
135.57
133.85
133.77
133.59
131.49
131.18
130.72
130.46
130.16
129.58
129.41
128.98
128.54
128.32
128.15
127.85
127.56
127.37
126.94
126.59
126.37
126.03
125.52
124.99
124.41
124.13
123.66
121.43
121.40
119.17
117.94
116.27
111.14
CDCl3
46.33
46.29
40.25
40.23
35.08
33.81
22.81
22.79
22.44
22.42
21.08
20.08
f1 (ppm)

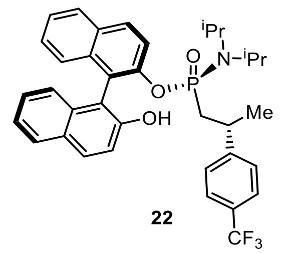

iPr
N
iPr
O
P
O
OH
Me
CF3

22

^{1}H-NMR

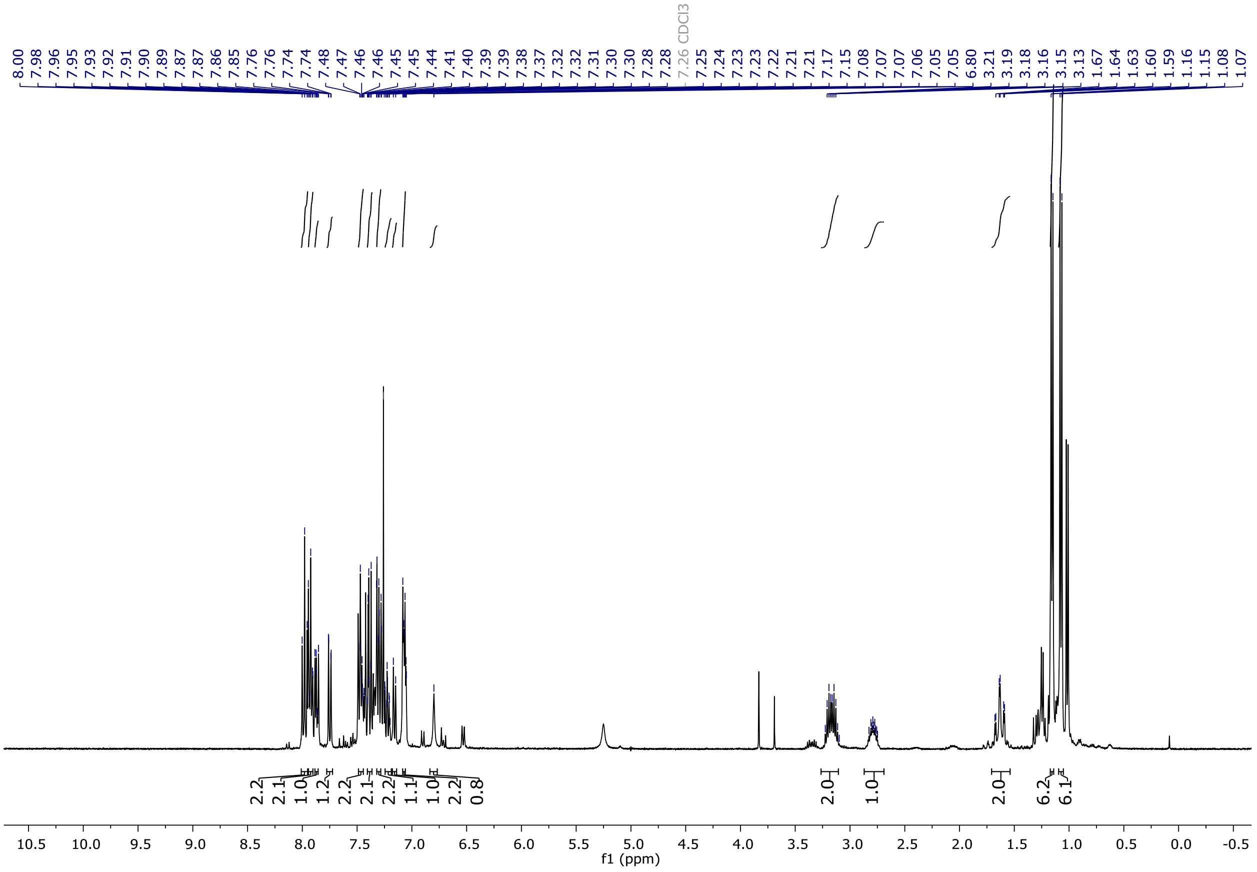

8.00
7.98
7.96
7.95
7.93
7.92
7.91
7.90
7.89
7.87
7.87
7.86
7.85
7.76
7.76
7.74
7.74
7.48
7.47
7.46
7.46
7.45
7.45
7.44
7.41
7.40
7.39
7.39
7.38
7.37
7.32
7.32
7.31
7.30
7.30
7.28
7.28
7.26 CDCl3
7.25
7.24
7.23
7.23
7.22
7.21
7.21
7.17
7.15
7.08
7.07
7.07
7.06
7.05
7.05
6.80
3.21
3.19
3.18
3.16
3.15
3.13
1.67
1.64
1.63
1.60
1.59
1.16
1.15
1.08
1.07
2.2
2.1
1.0
1.2
2.2
2.1
2.2
1.1
1.0
2.2
0.8
2.0
1.0
2.0
6.2
6.1
10.5
10.0
9.5
9.0
8.5
8.0
7.5
7.0
6.5
6.0
5.5
5.0
4.5
4.0
3.5
3.0
2.5
2.0
1.5
1.0
0.5
0.0
-0.5
f1 (ppm)

^{31}P-NMR

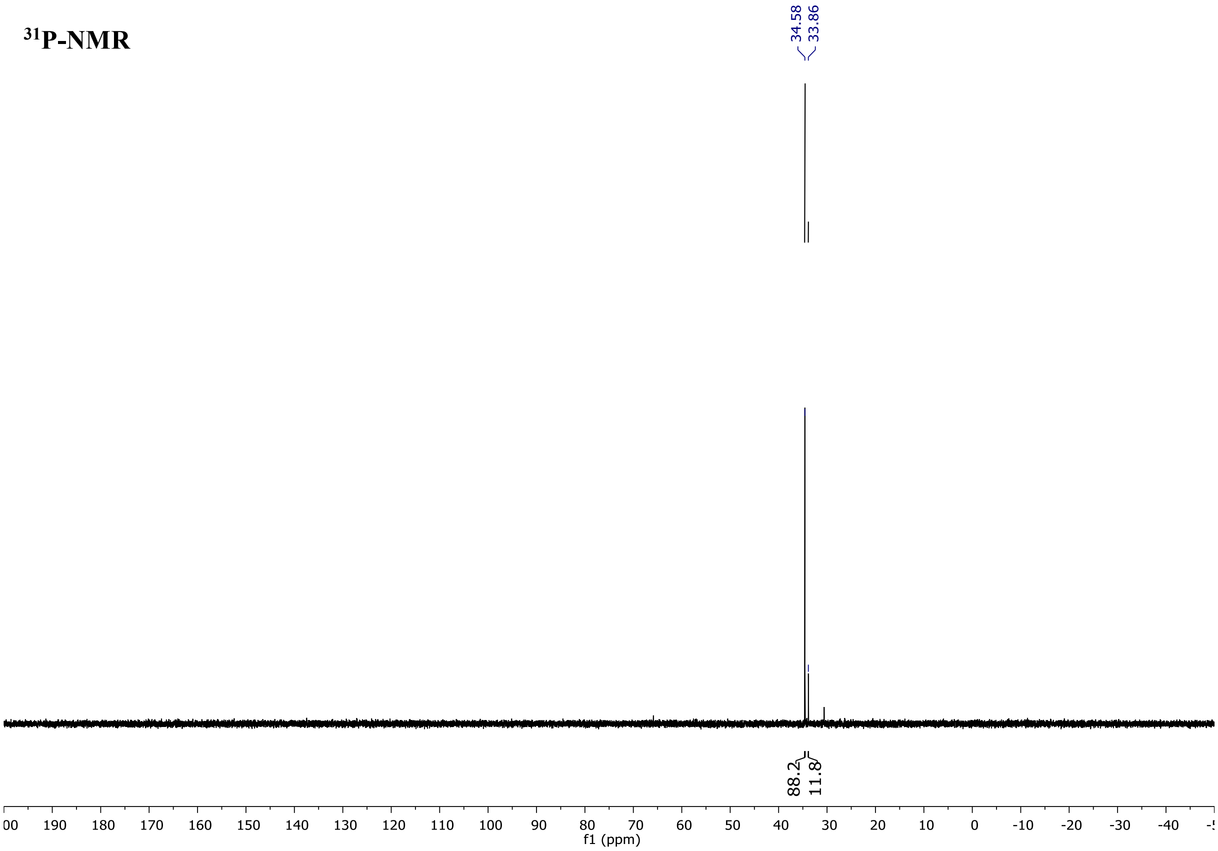

^{19}F-NMR

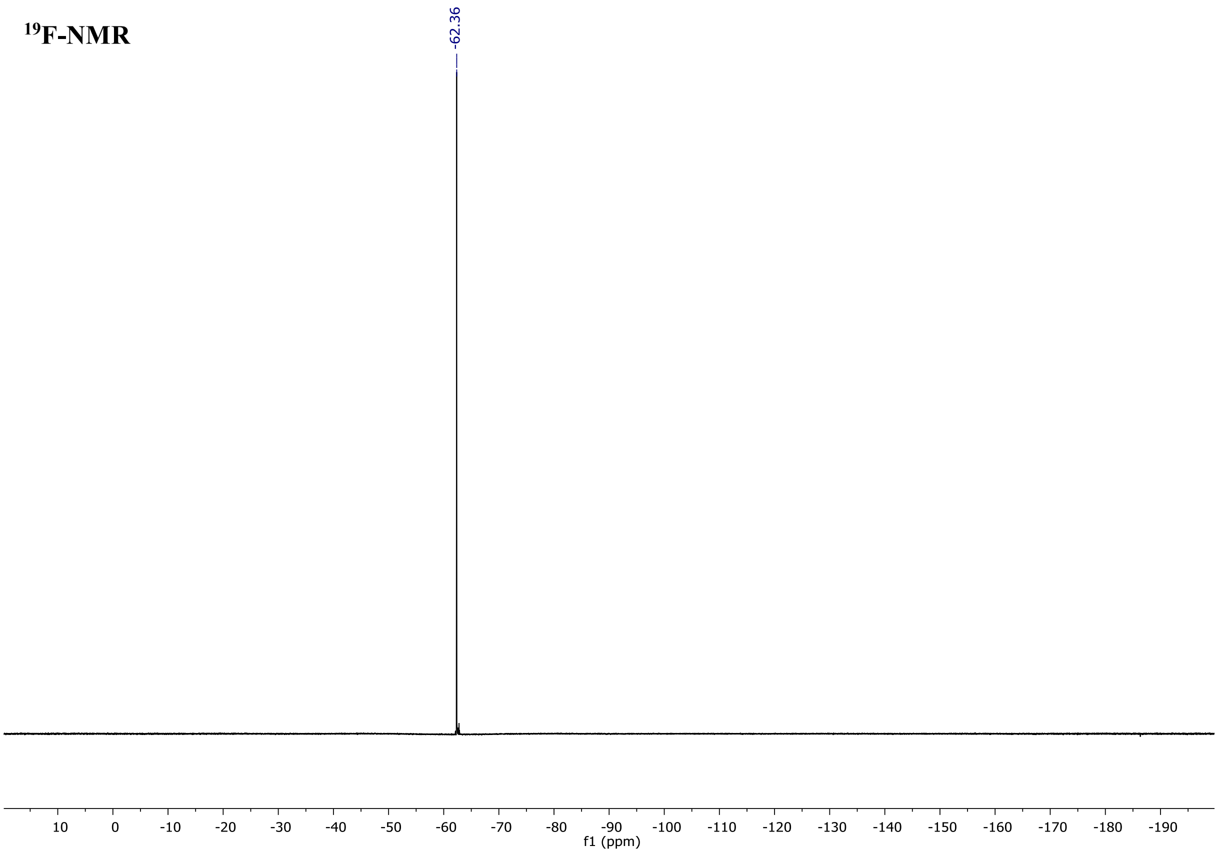

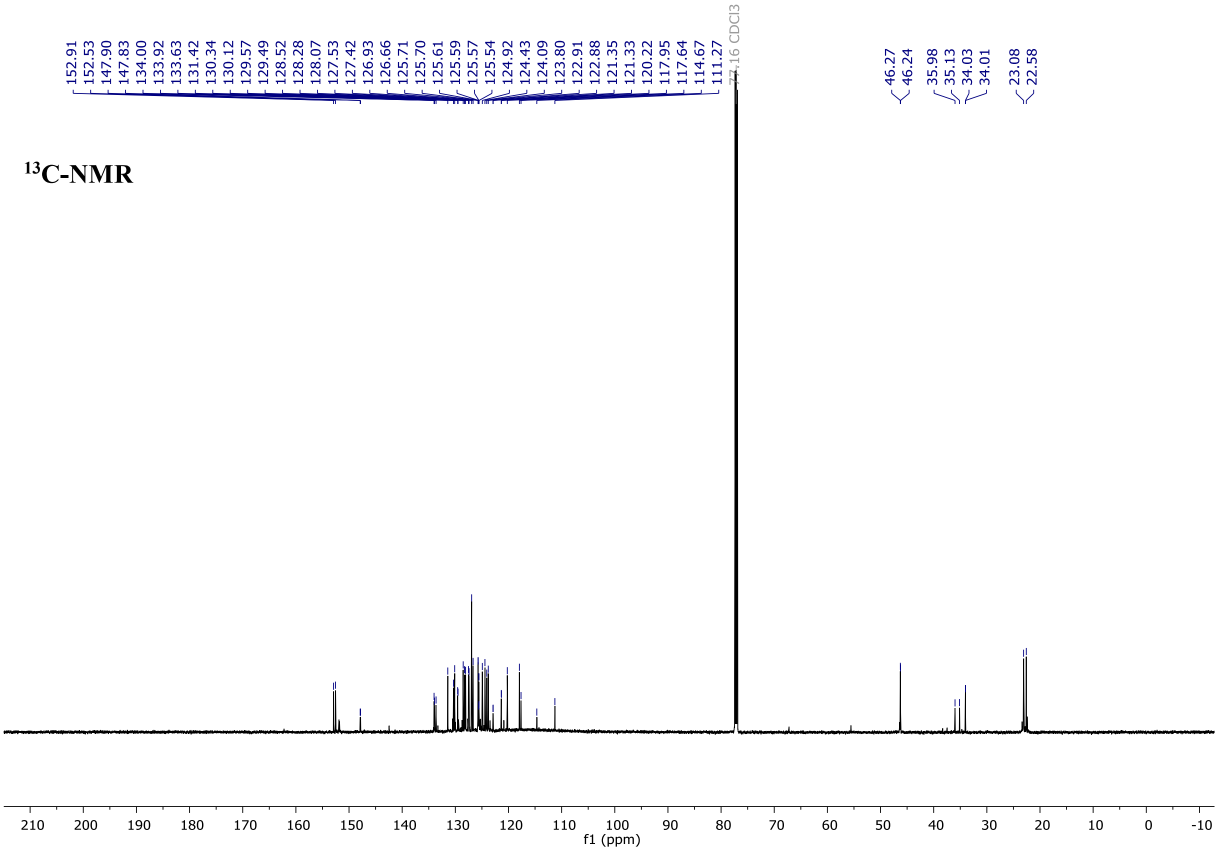

13C-NMR
152.91
152.53
147.90
147.83
134.00
133.92
133.63
131.42
130.34
130.12
129.57
129.49
128.52
128.28
128.07
127.53
127.42
126.93
126.66
125.71
125.70
125.61
125.59
125.57
125.54
124.92
124.43
124.09
123.80
122.91
122.88
121.35
121.33
120.22
117.95
117.64
114.67
111.27
77.16 CDCl3
46.27
46.24
35.98
35.13
34.03
34.01
23.08
22.58
f1 (ppm)

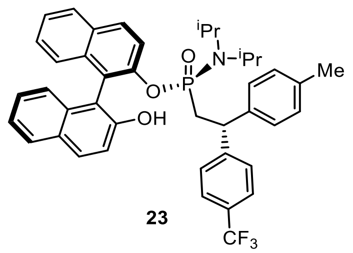

23

^{1}H-NMR

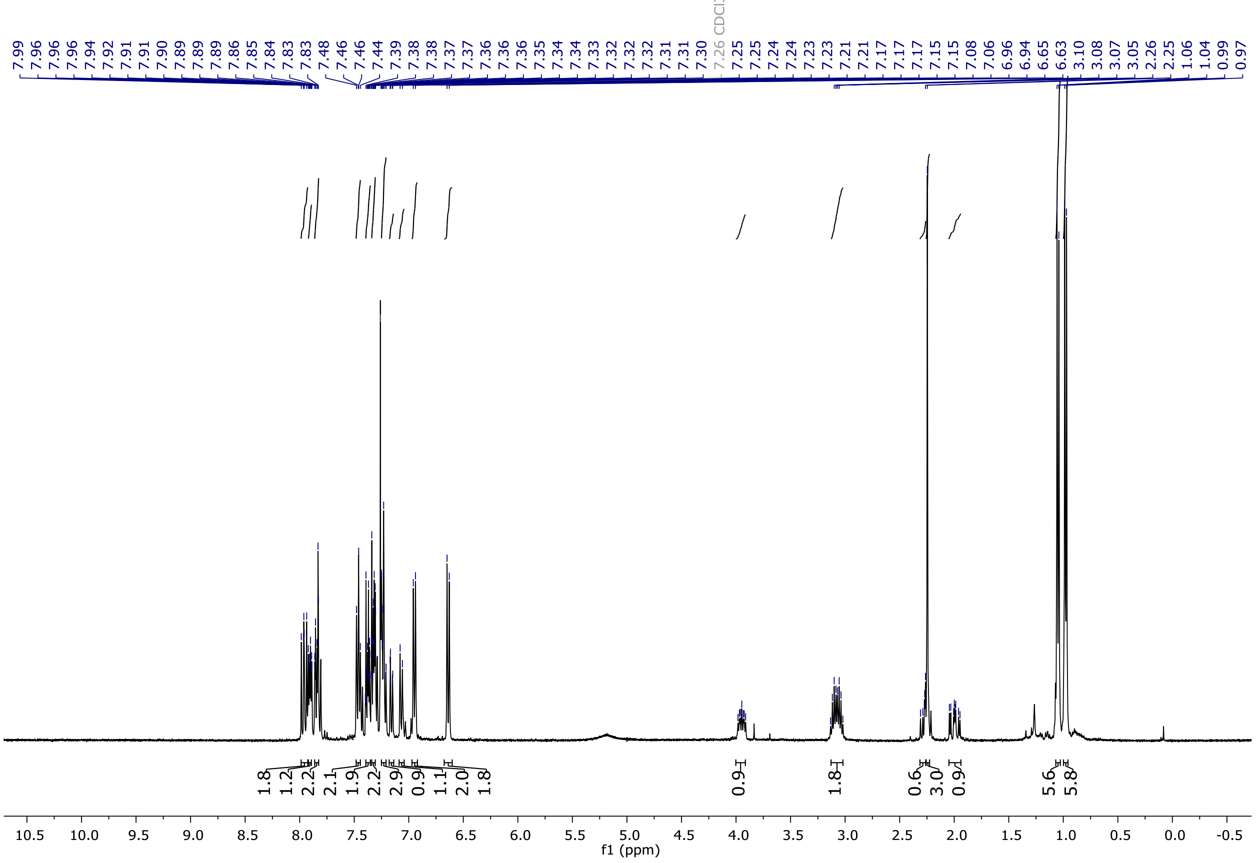

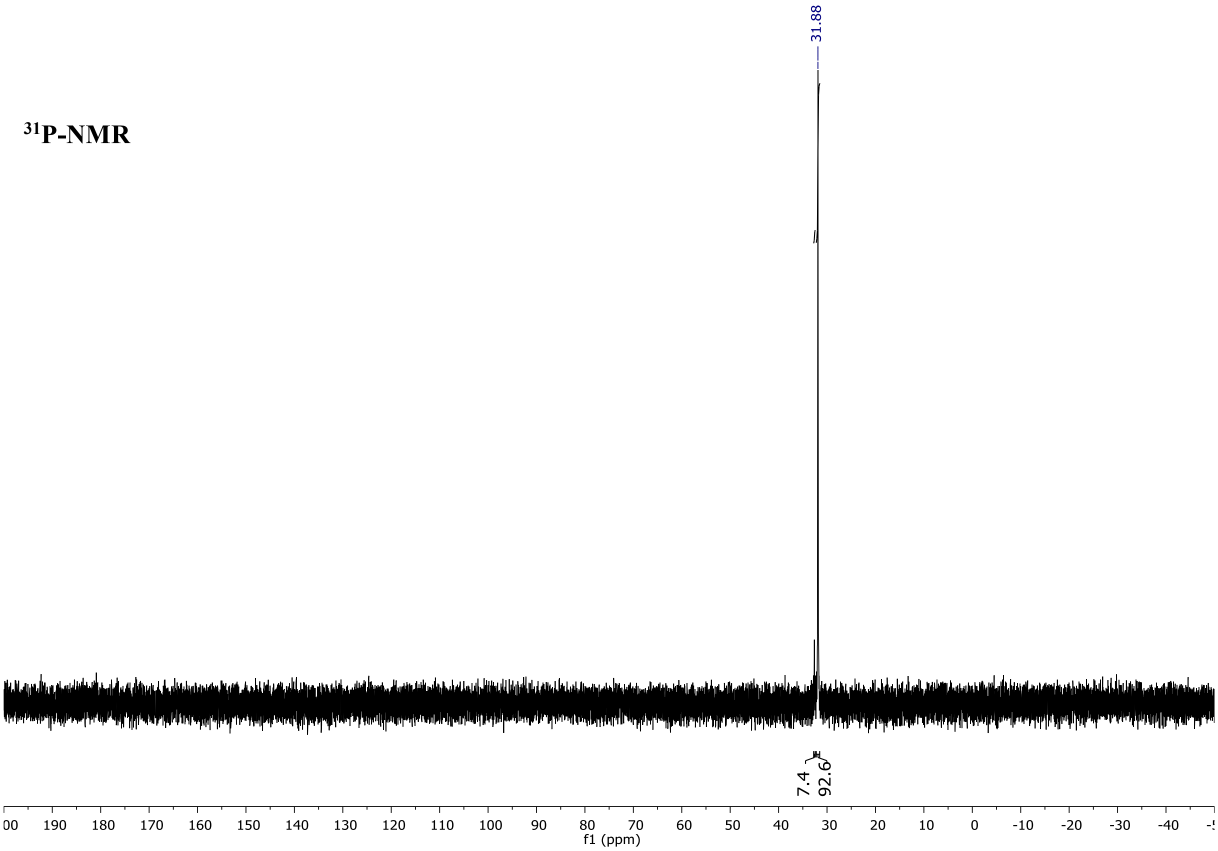
31.88
7.4
92.6
f1 (ppm)

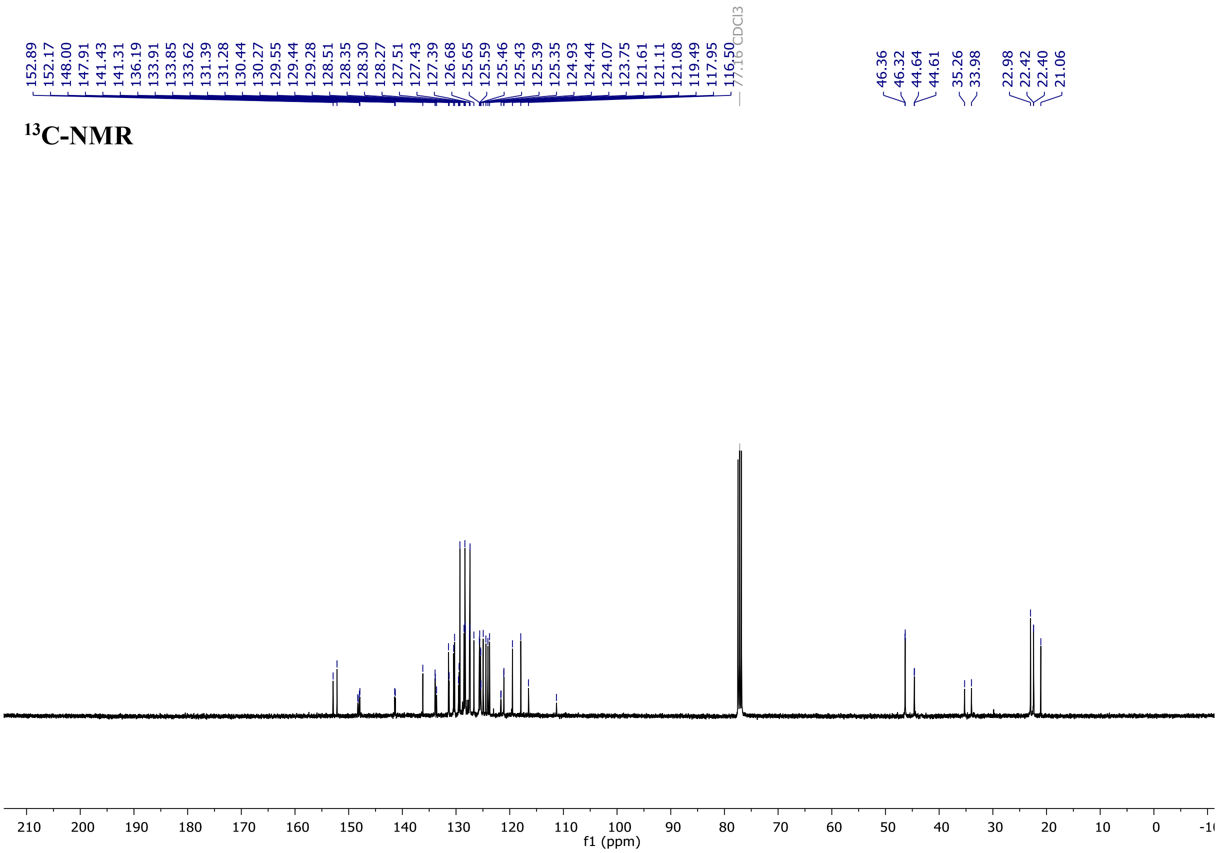
152.89
152.17
148.00
147.91
141.43
141.31
136.19
133.91
133.85
133.62
131.39
131.28
130.44
130.27
129.55
129.44
129.28
128.51
128.35
128.30
128.27
127.51
127.43
127.39
126.68
125.65
125.59
125.46
125.43
125.39
125.35
124.93
124.44
124.07
123.75
121.61
121.11
121.08
119.49
117.95
116.50
77.16 CDCl3
46.36
46.32
44.64
44.61
35.26
33.98
22.98
22.42
22.40
21.06
f1 (ppm)

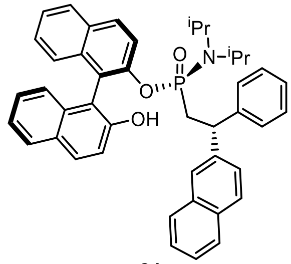

24

^{1}H-NMR

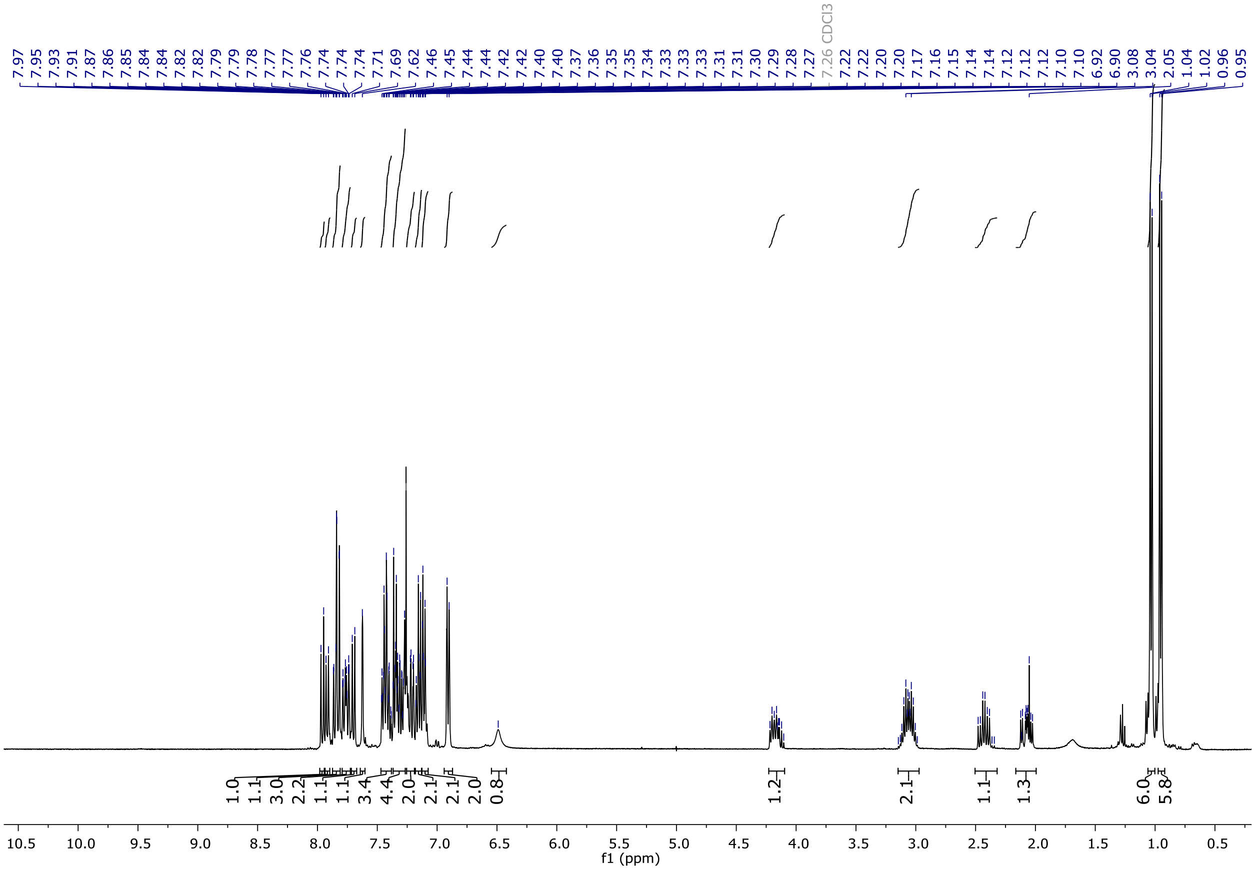

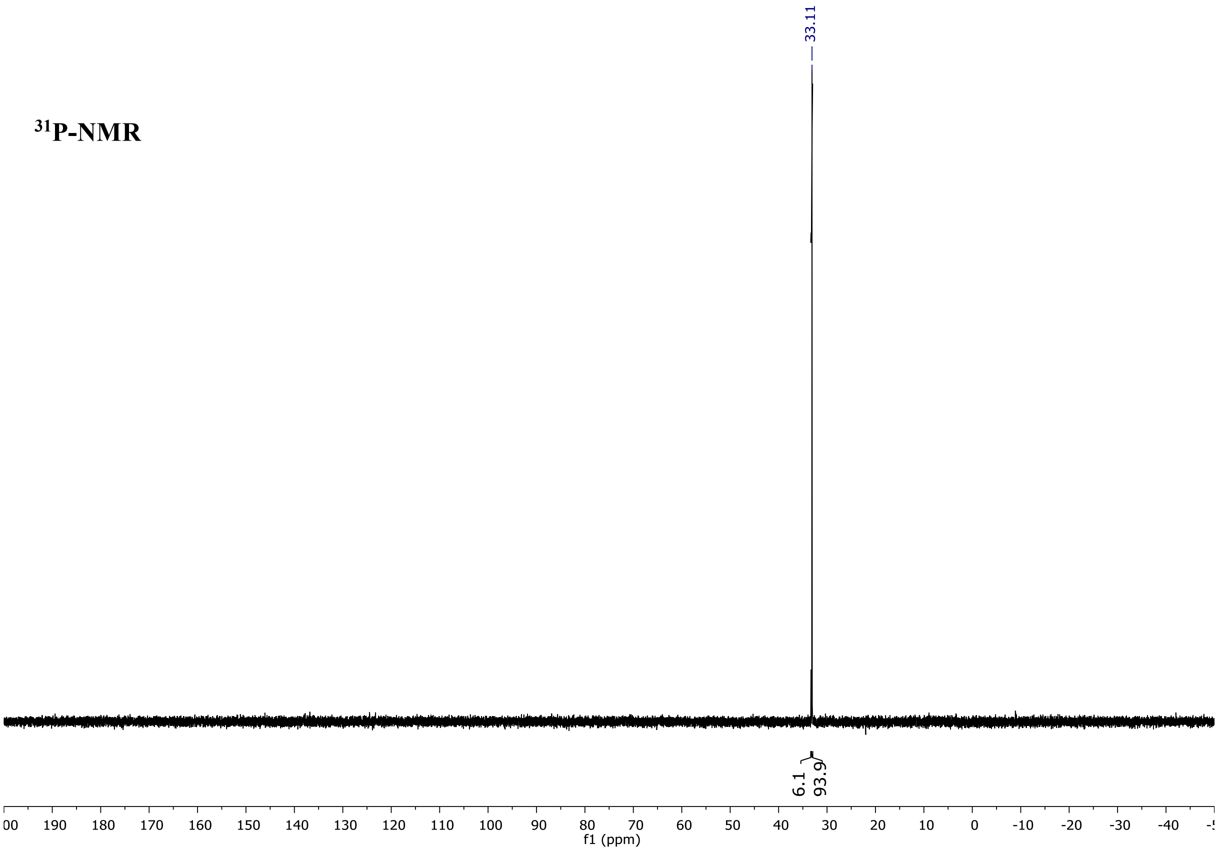

^{31}P-NMR
33.11
6.1
93.9
f1 (ppm)

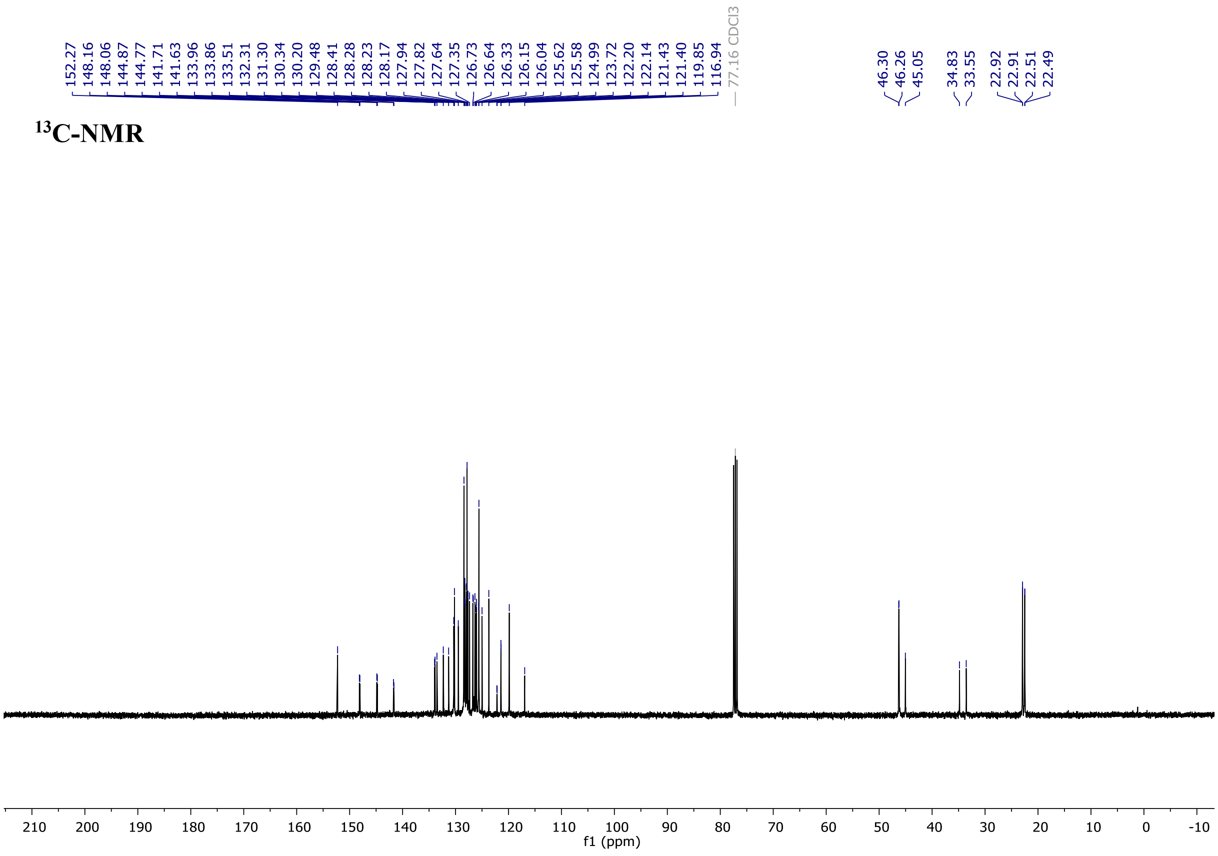

^{13}C-NMR
152.27 148.16 148.06 144.87 144.77 141.71 141.63 133.96 133.86 133.51 132.31 131.30 130.34 130.20 129.48 128.41 128.28 128.23 128.17 127.94 127.82 127.64 127.35 126.73 126.64 126.33 126.15 126.04 125.62 125.58 124.99 123.72 122.20 122.14 121.43 121.40 119.85 116.94
77.16 CDCl3
46.30 46.26 45.05
34.83 33.55
22.92 22.91 22.51 22.49
f1 (ppm)

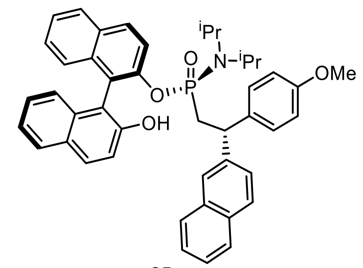

iPr
N
iPr
OMe
O
P
O
OH

25

^{1}H-NMR

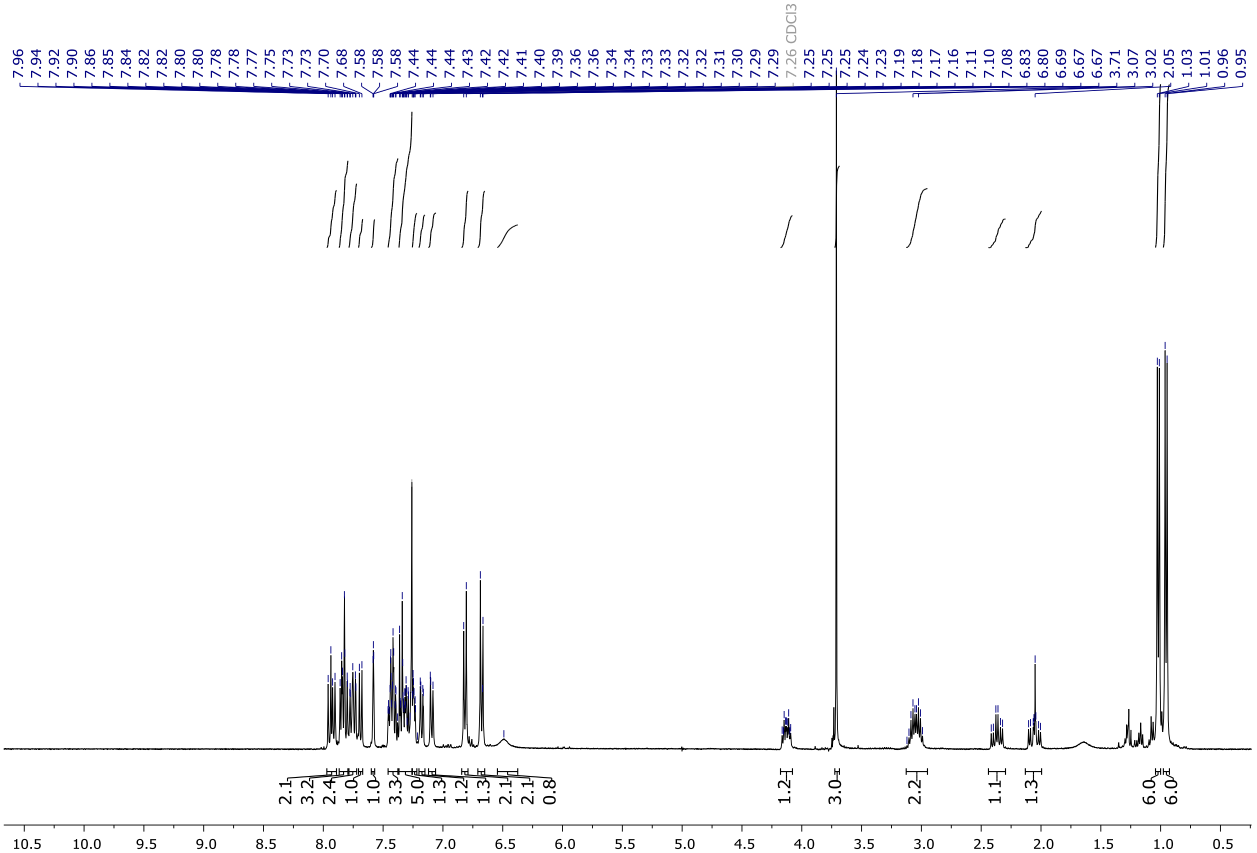

7.96
7.94
7.92
7.90
7.86
7.85
7.84
7.82
7.80
7.80
7.78
7.78
7.77
7.75
7.73
7.73
7.70
7.68
7.58
7.58
7.44
7.44
7.44
7.43
7.42
7.42
7.41
7.40
7.39
7.36
7.36
7.34
7.34
7.33
7.33
7.32
7.32
7.31
7.30
7.29
7.29
7.26 CDCl3
7.25
7.25
7.25
7.24
7.23
7.23
7.19
7.18
7.17
7.16
7.11
7.10
7.08
6.83
6.80
6.69
6.67
6.67
3.71
3.07
3.02
2.05
1.03
1.01
0.96
0.95
2.1
3.2
2.4
1.0
1.0
3.3
5.0
1.3
1.2
1.3
2.1
2.1
0.8
1.2
3.0
2.2
1.1
1.3
6.0
6.0
10.5
10.0
9.5
9.0
8.5
8.0
7.5
7.0
6.5
6.0
5.5
5.0
4.5
4.0
3.5
3.0
2.5
2.0
1.5
1.0
0.5
f1 (ppm)

^{31}P-NMR

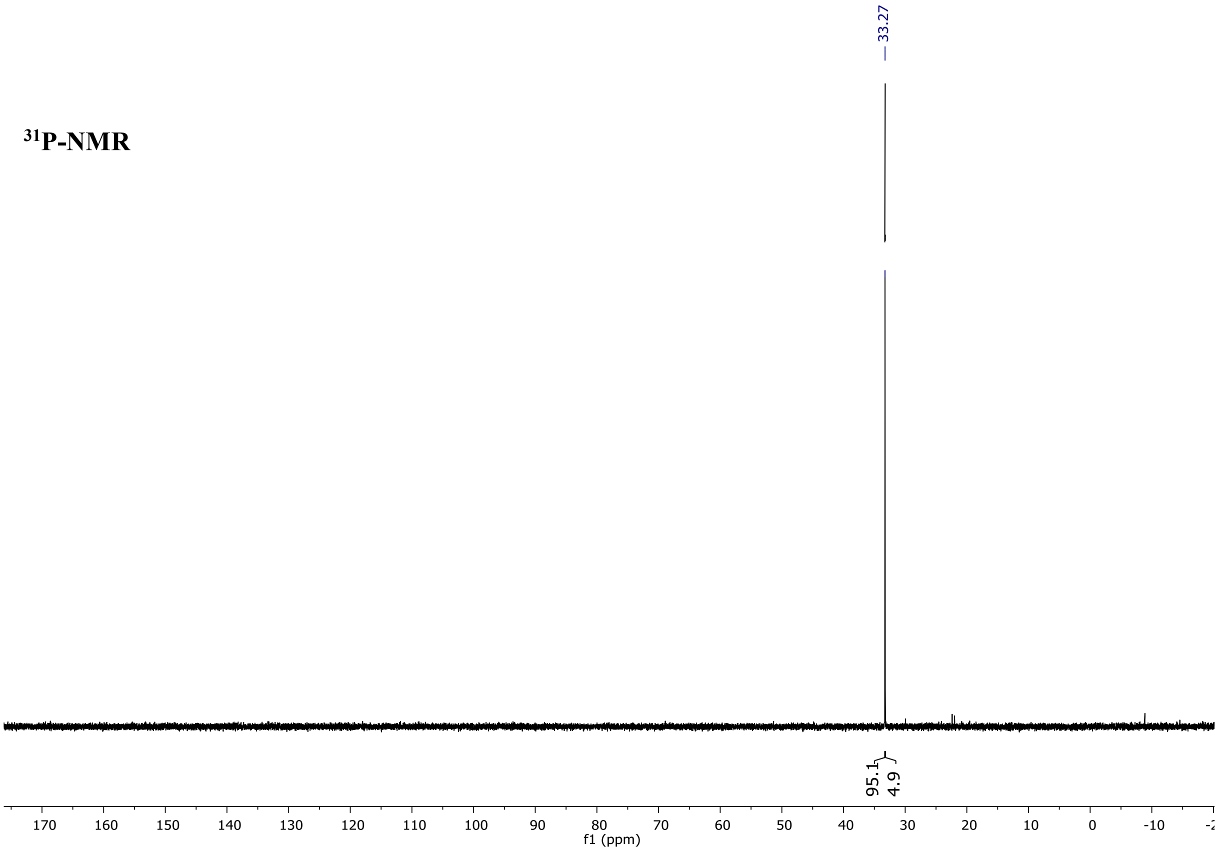

^{13}C-NMR

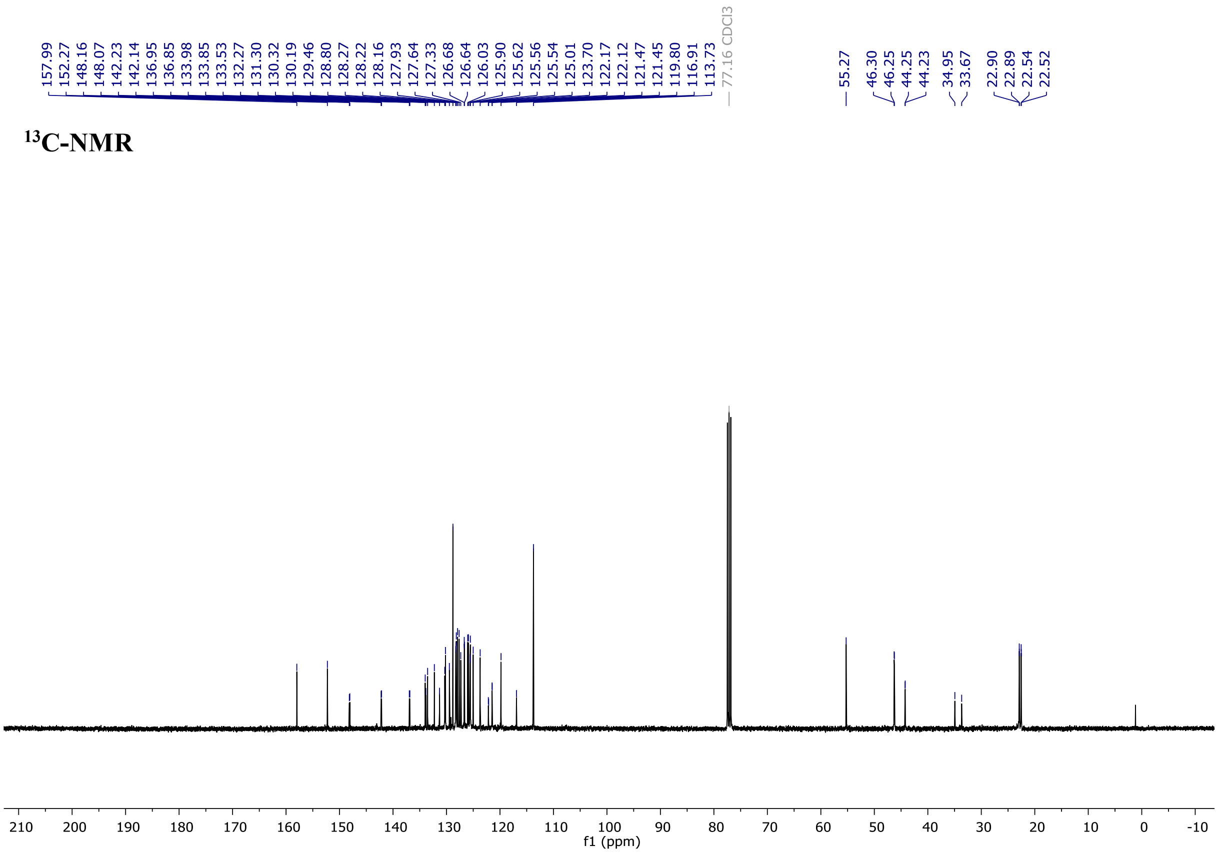

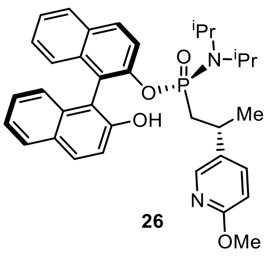

26

^{1}H-NMR

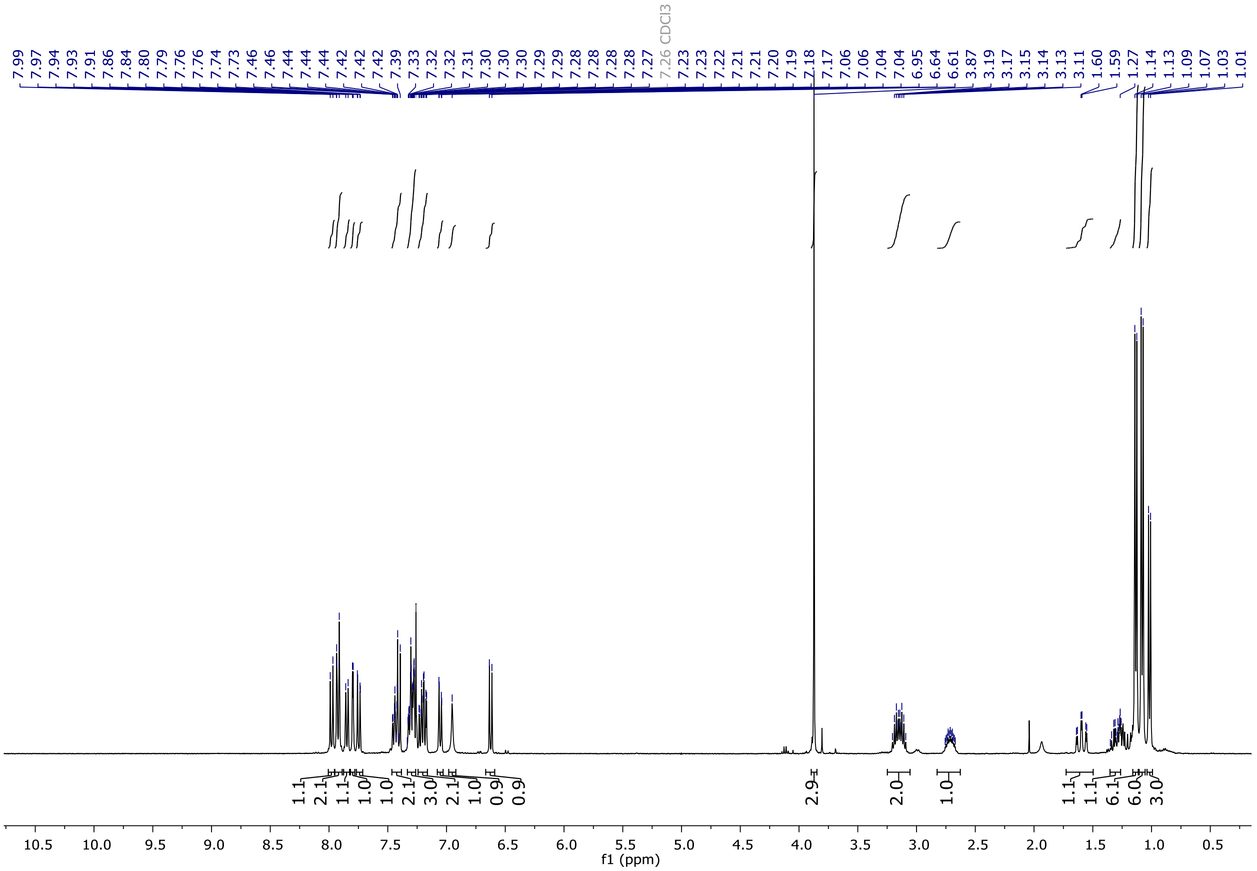

^{31}P-NMR

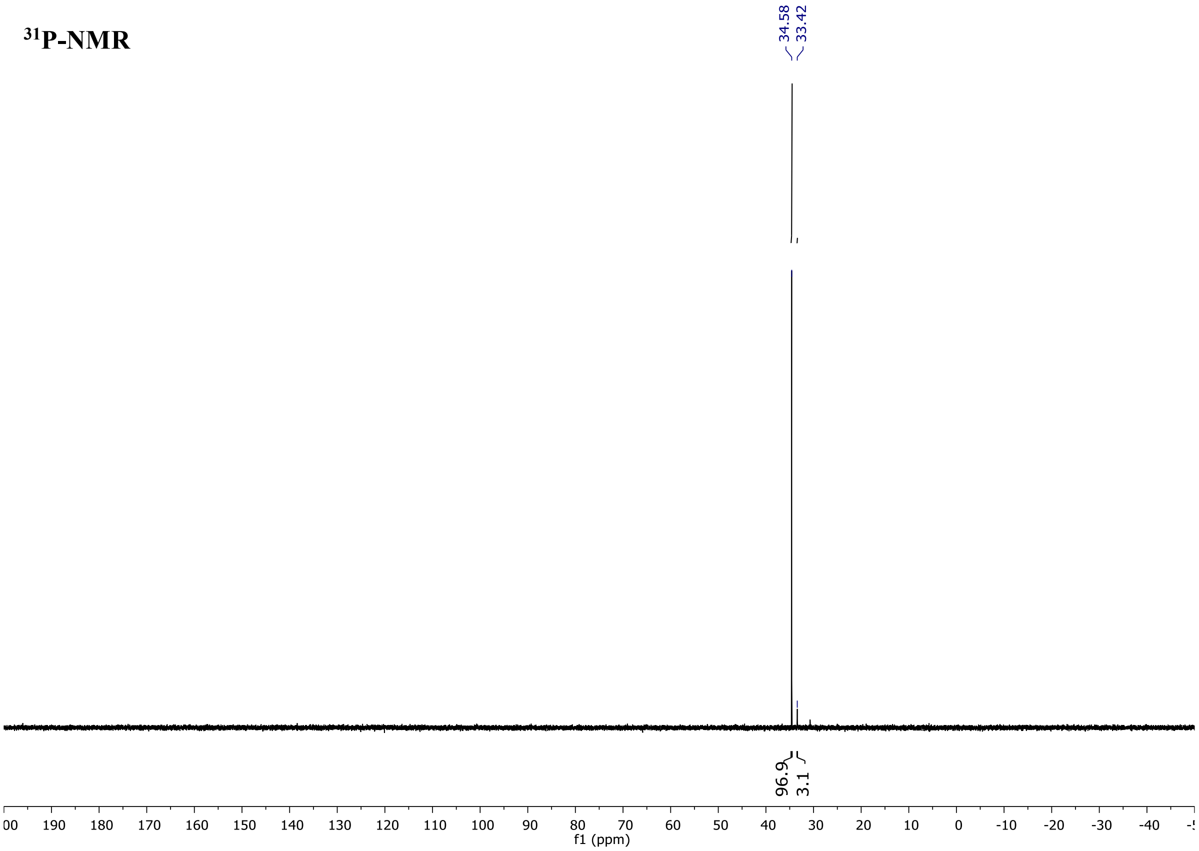

^{13}C-NMR

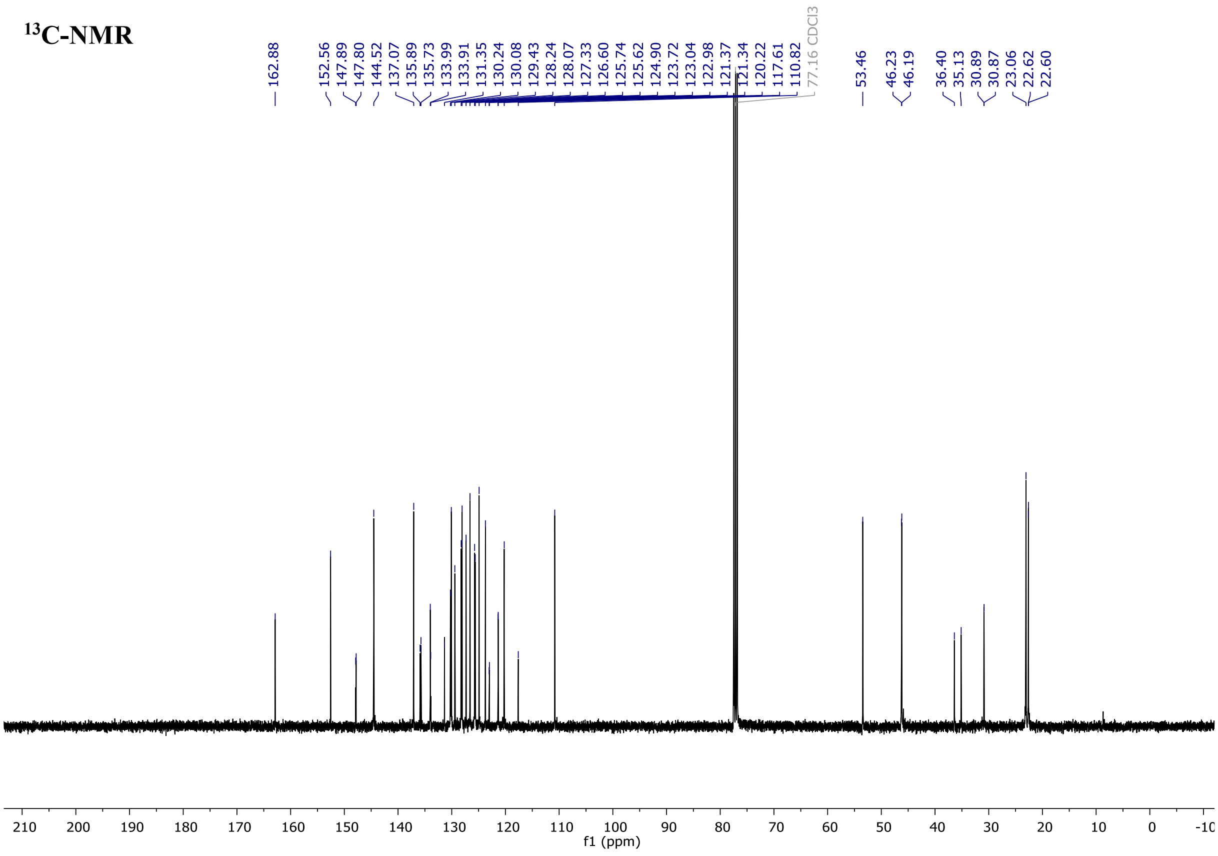

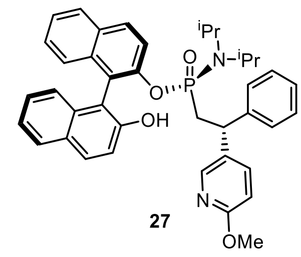

iPr
N
iPr
O
P
O
OH
N
OMe

27

^{1}H-NMR

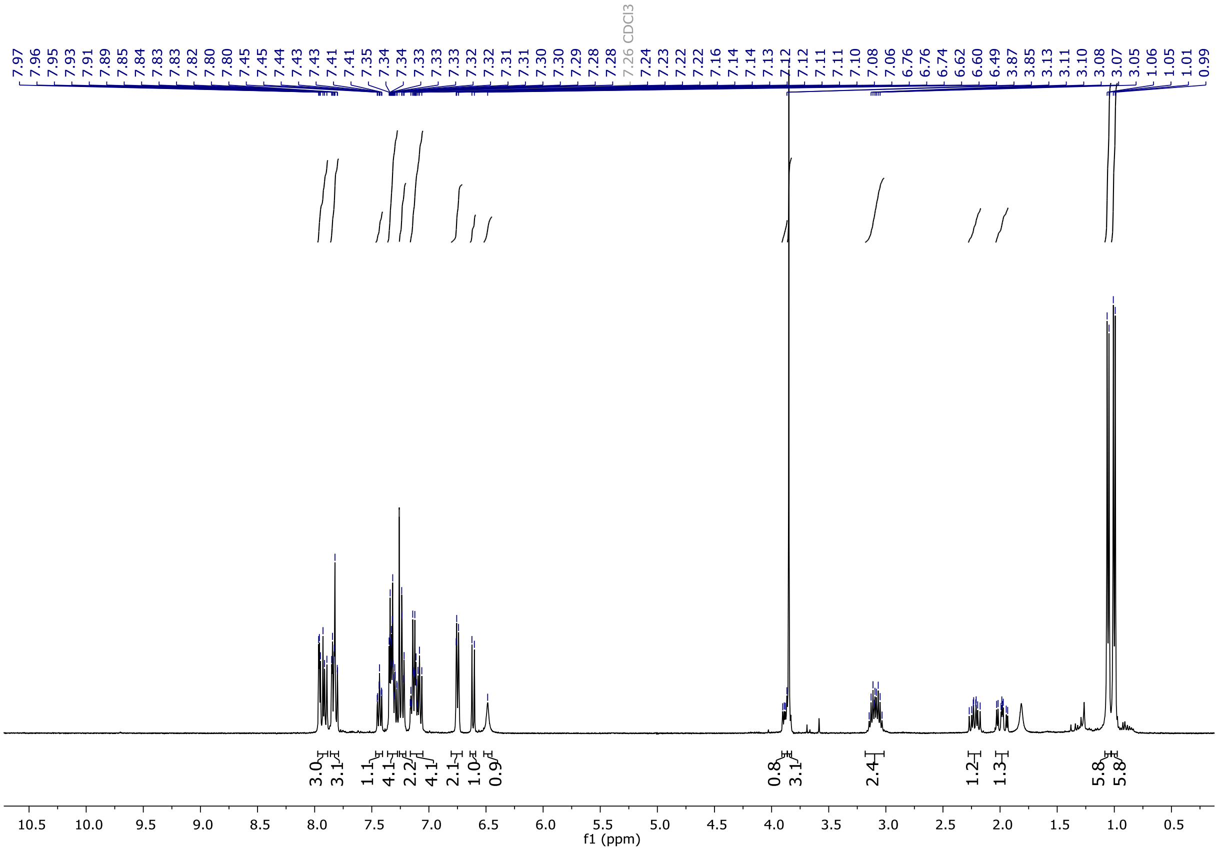

7.97
7.96
7.95
7.93
7.91
7.89
7.85
7.84
7.83
7.83
7.82
7.80
7.80
7.45
7.45
7.44
7.43
7.43
7.41
7.41
7.35
7.34
7.34
7.33
7.33
7.33
7.32
7.32
7.31
7.31
7.30
7.30
7.29
7.28
7.28
7.26 CDCl3
7.24
7.23
7.22
7.22
7.16
7.14
7.14
7.13
7.12
7.12
7.11
7.11
7.10
7.08
7.06
6.76
6.76
6.74
6.62
6.60
6.49
3.87
3.85
3.13
3.11
3.10
3.08
3.07
3.05
1.06
1.05
1.01
0.99
3.0
3.1
1.1
4.1
2.2
4.1
2.1
1.0
0.9
0.8
3.1
2.4
1.2
1.3
5.8
5.8
10.5
10.0
9.5
9.0
8.5
8.0
7.5
7.0
6.5
6.0
5.5
5.0
4.5
4.0
3.5
3.0
2.5
2.0
1.5
1.0
0.5
f1 (ppm)

^{31}P-NMR

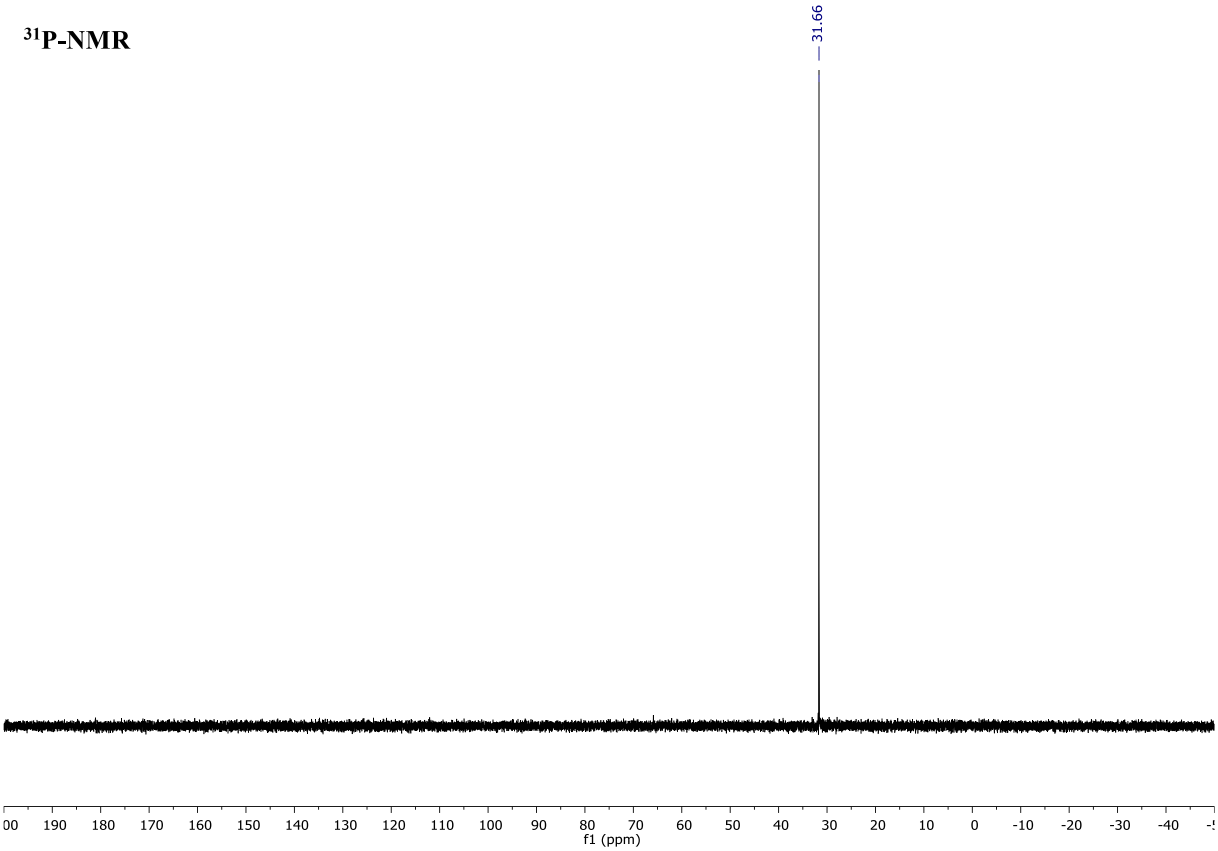

^{13}C-NMR

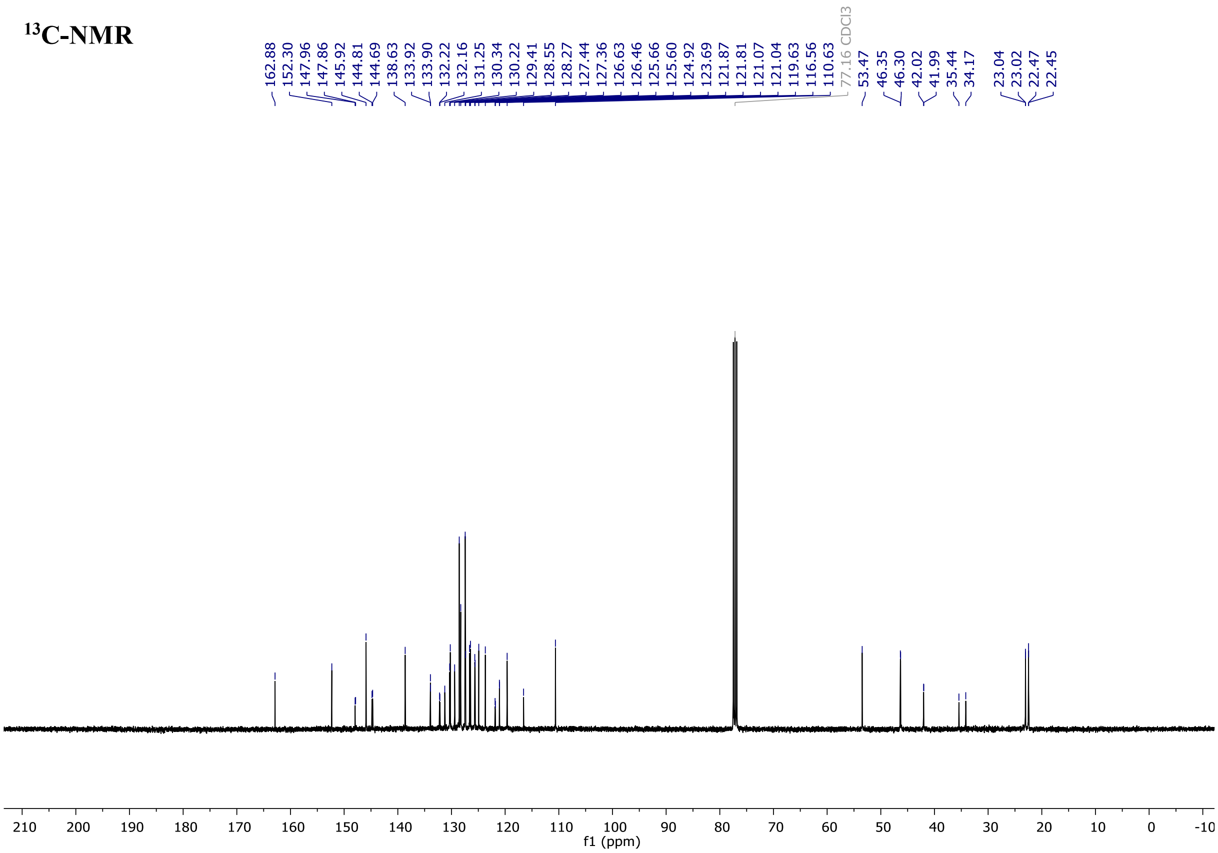

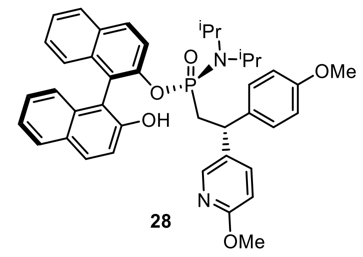

28

^{1}H-NMR

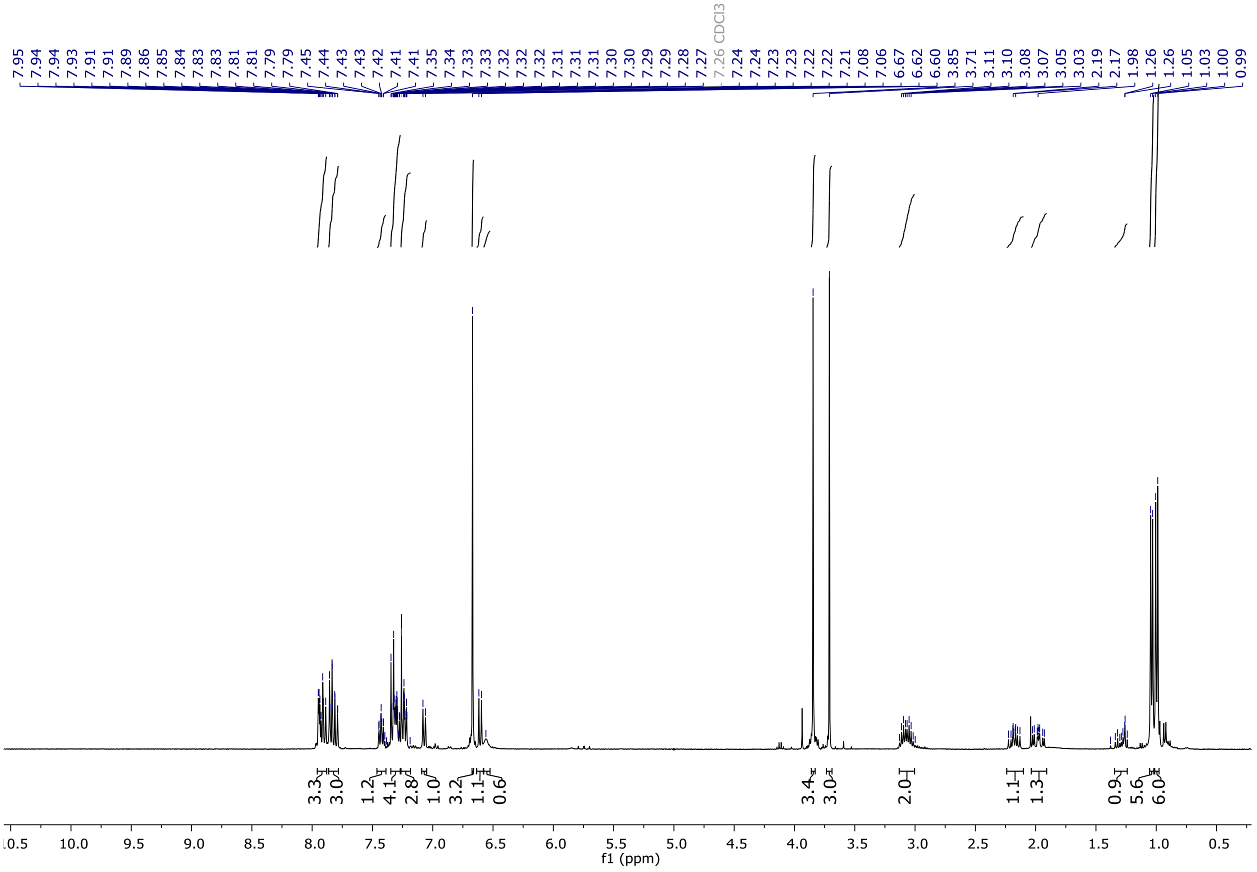

^{31}P-NMR

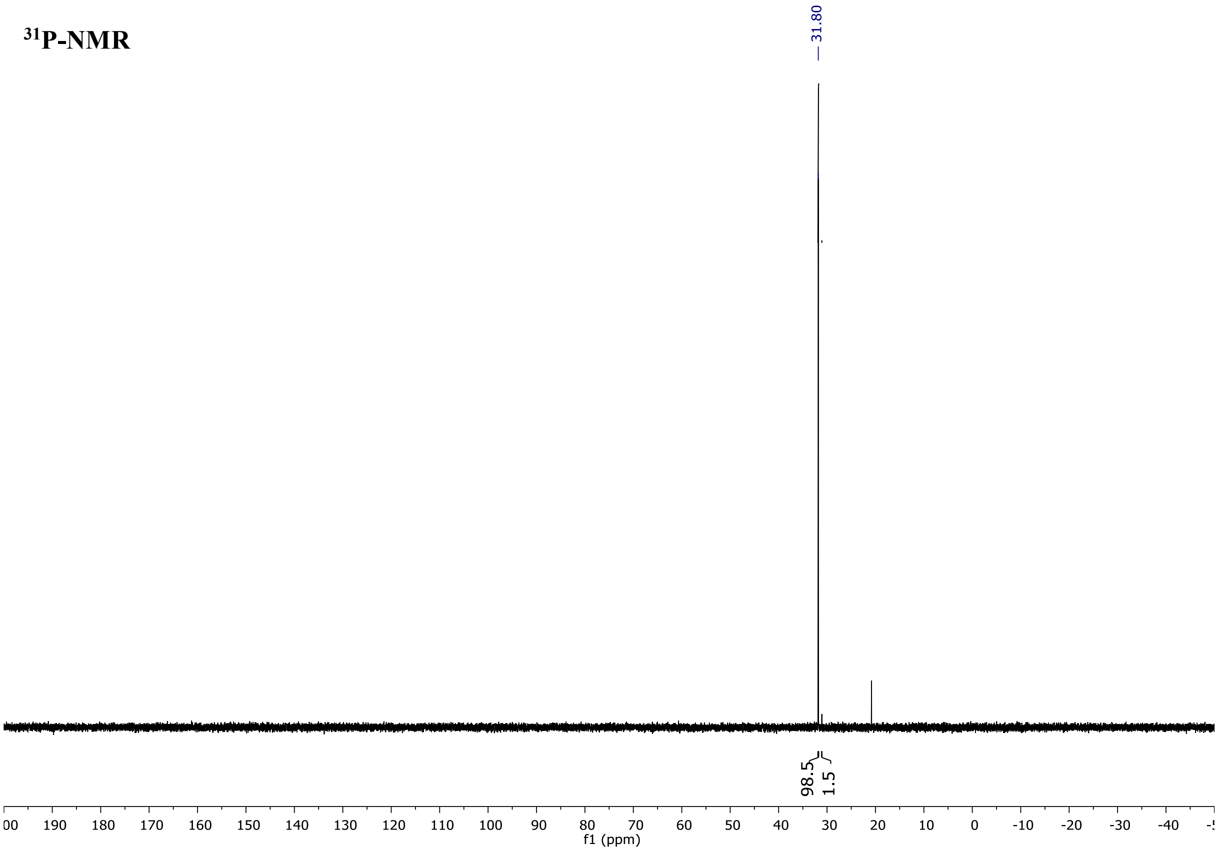

^{13}C-NMR

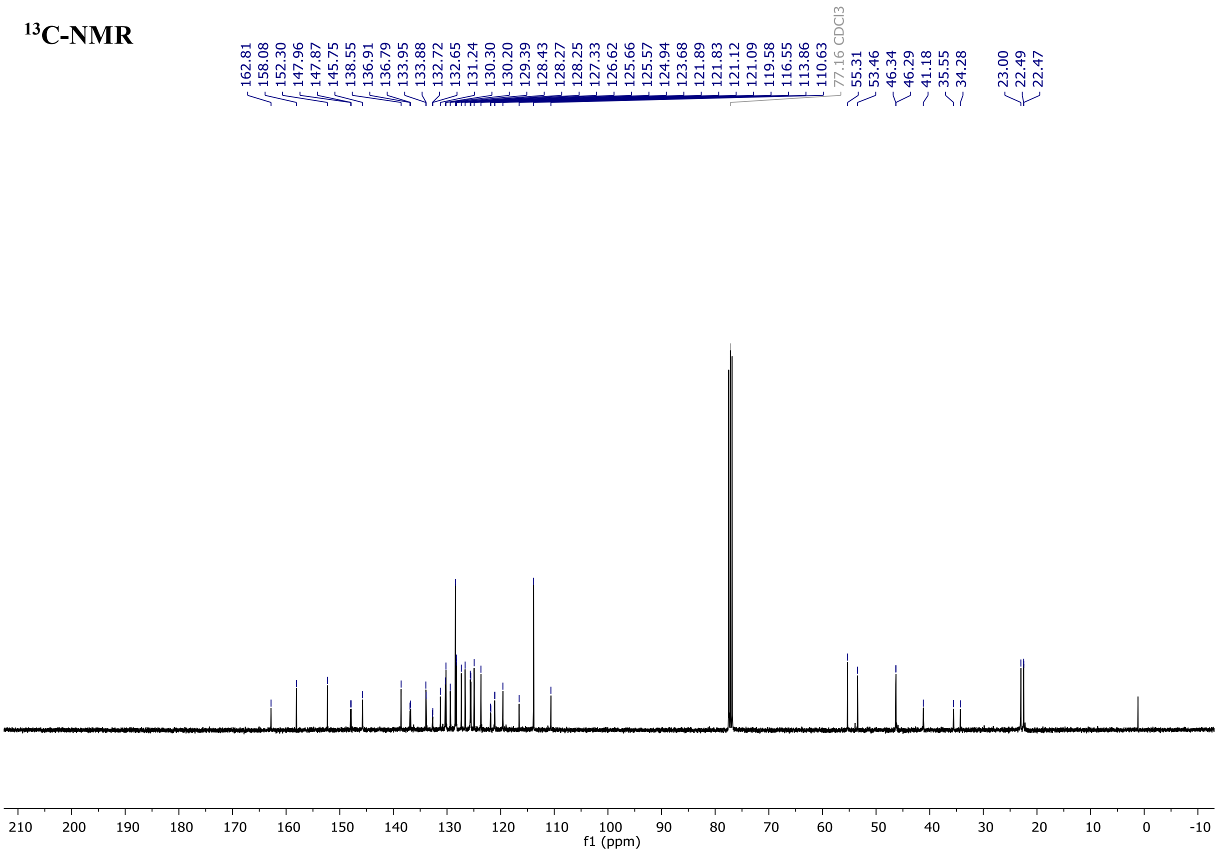

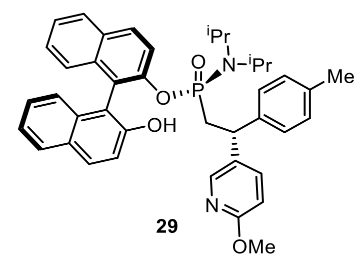

iPr
N
iPr
O
P
O
Me
OH
N
OMe

29

^{1}H-NMR

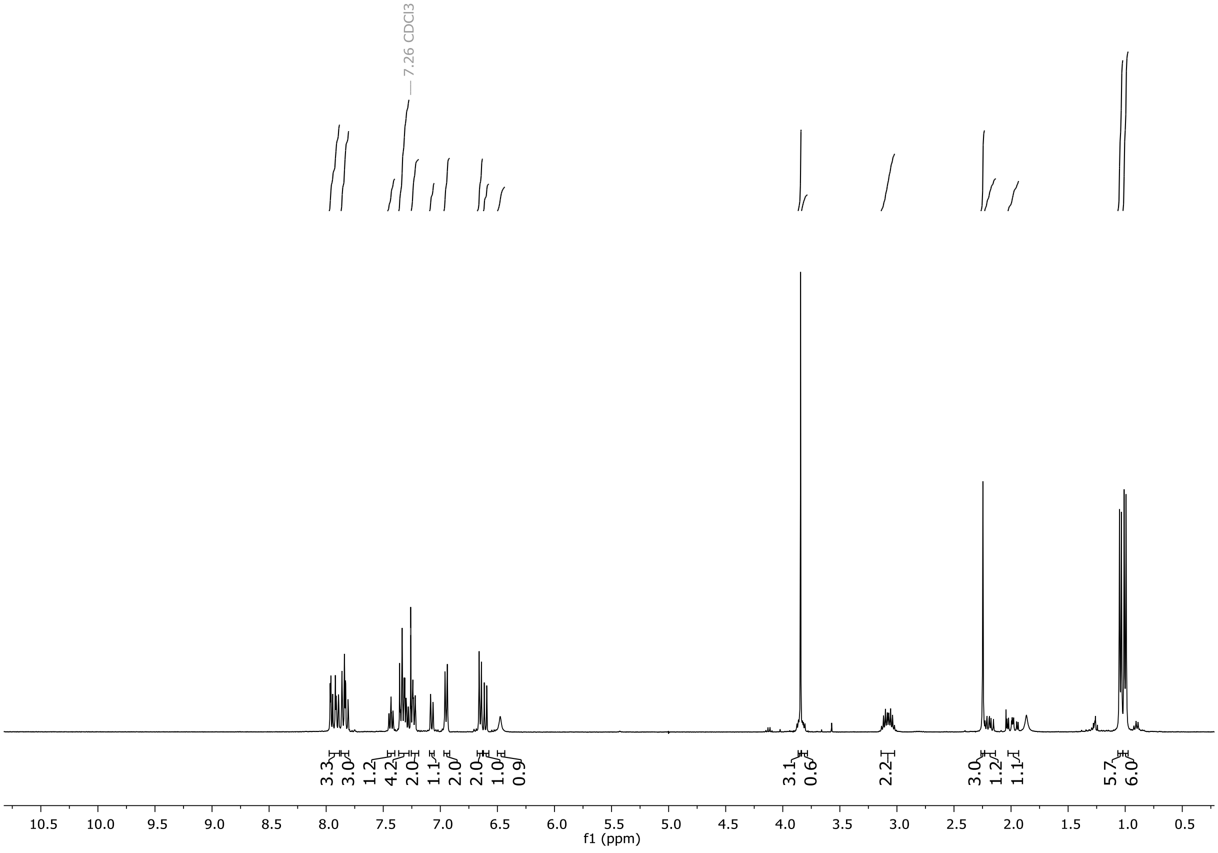

7.26 CDCl3
3.3
3.0
1.2
4.2
2.0
1.1
2.0
2.0
1.0
0.9
3.1
0.6
2.2
3.0
1.2
1.1
5.7
6.0
10.5
10.0
9.5
9.0
8.5
8.0
7.5
7.0
6.5
6.0
5.5
5.0
4.5
4.0
3.5
3.0
2.5
2.0
1.5
1.0
0.5
f1 (ppm)

31P-NMR

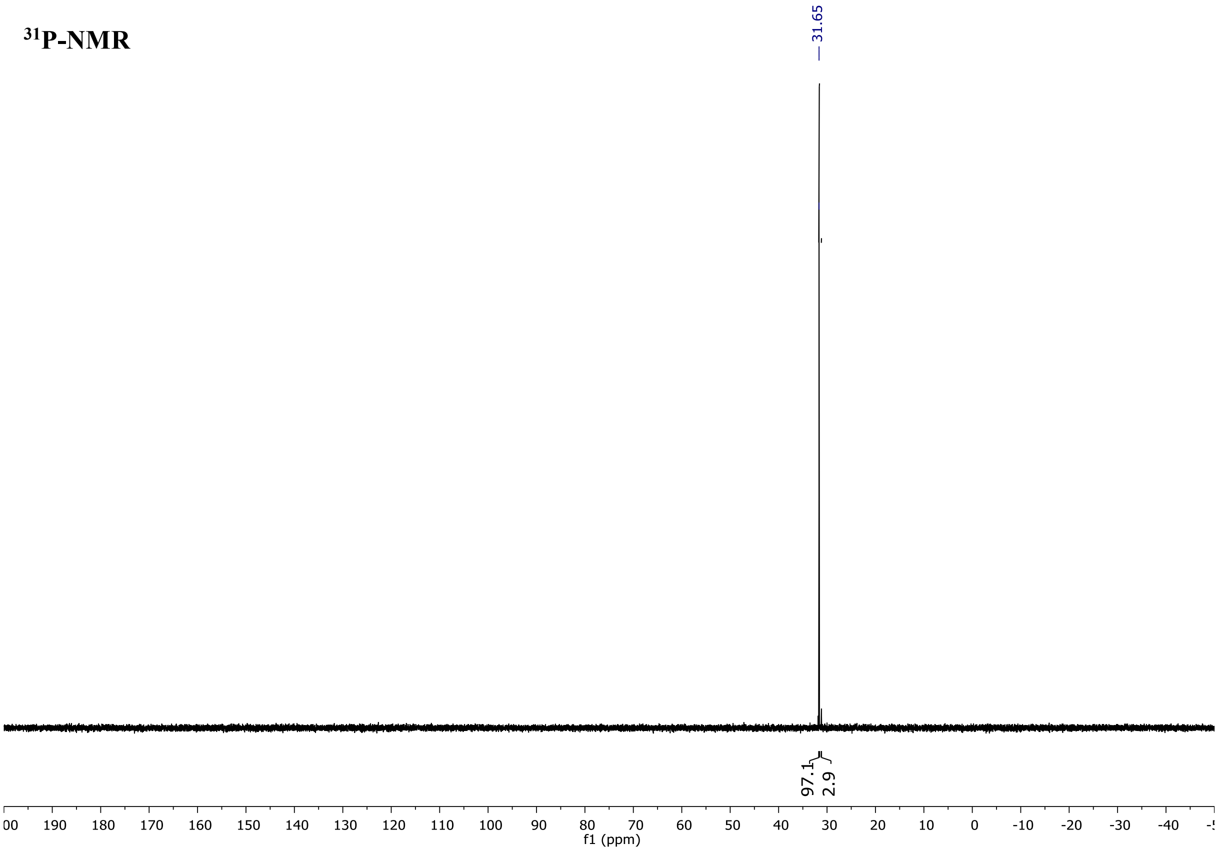

31.65
97.1
2.9
f1 (ppm)

13C-NMR

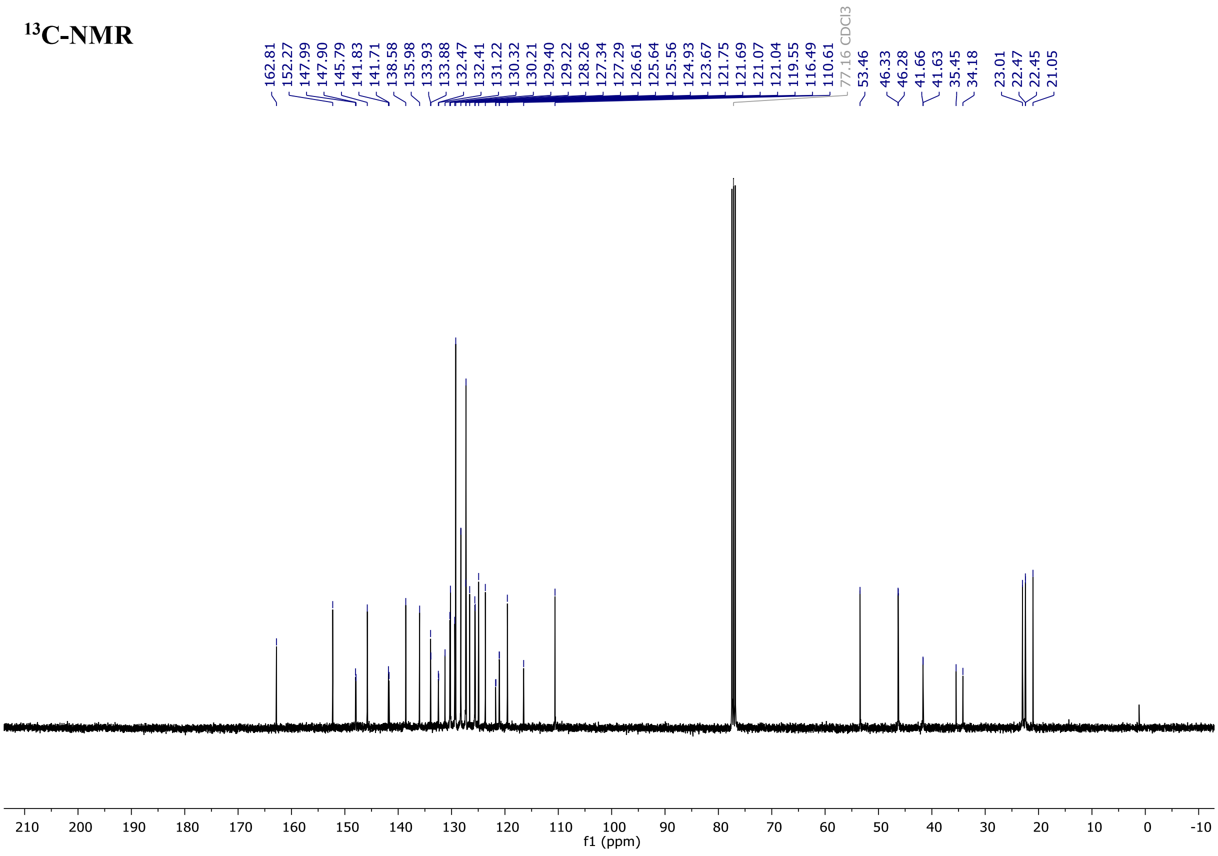

162.81
152.27
147.99
147.90
145.79
141.83
141.71
138.58
135.98
133.93
133.88
132.47
132.41
131.22
130.32
130.21
129.40
129.22
128.26
127.34
127.29
126.61
125.64
125.56
124.93
123.67
121.75
121.69
121.07
121.04
119.55
116.49
110.61
77.16 CDCl3
53.46
46.33
46.28
41.66
41.63
35.45
34.18
23.01
22.47
22.45
21.05
f1 (ppm)

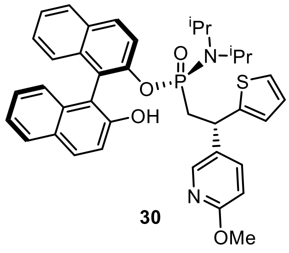

30

¹H-NMR

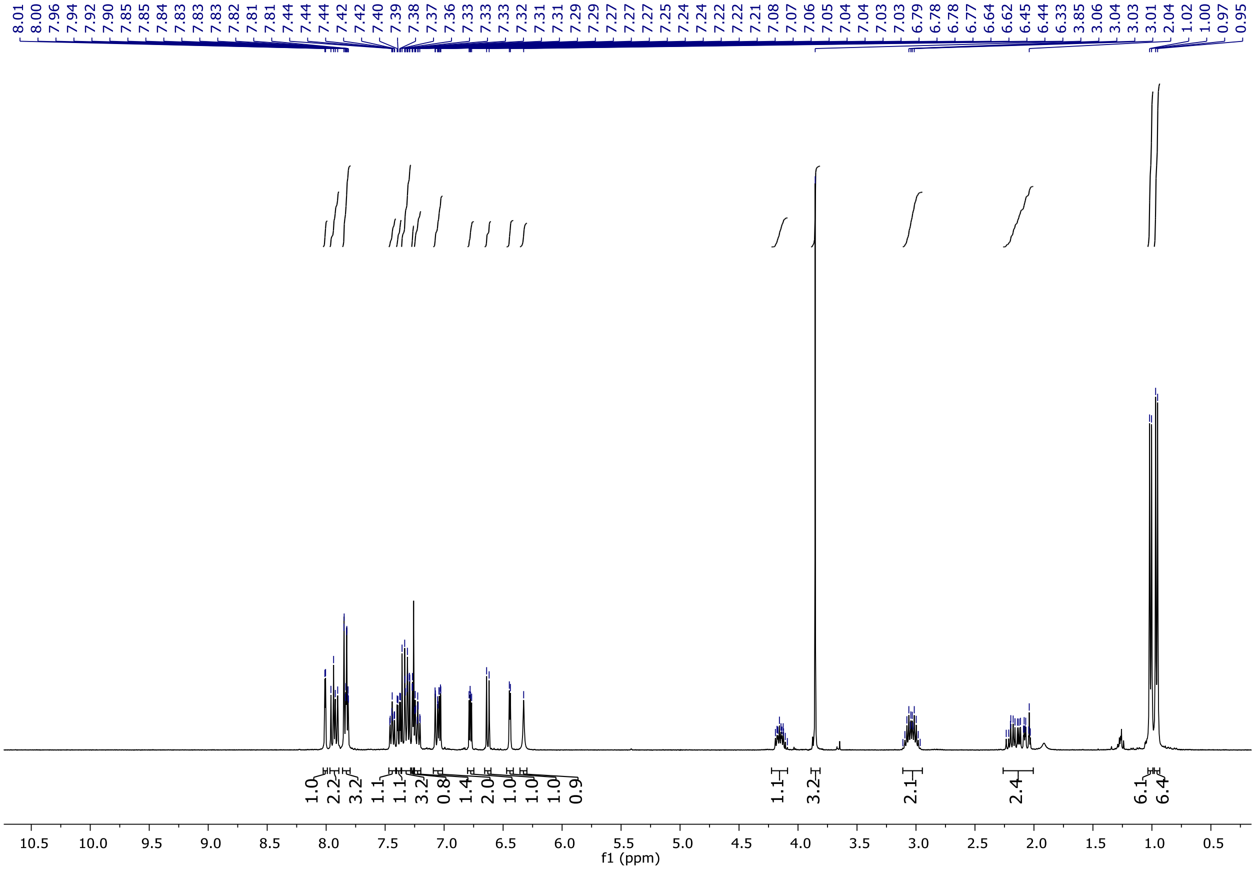

^{31}P-NMR

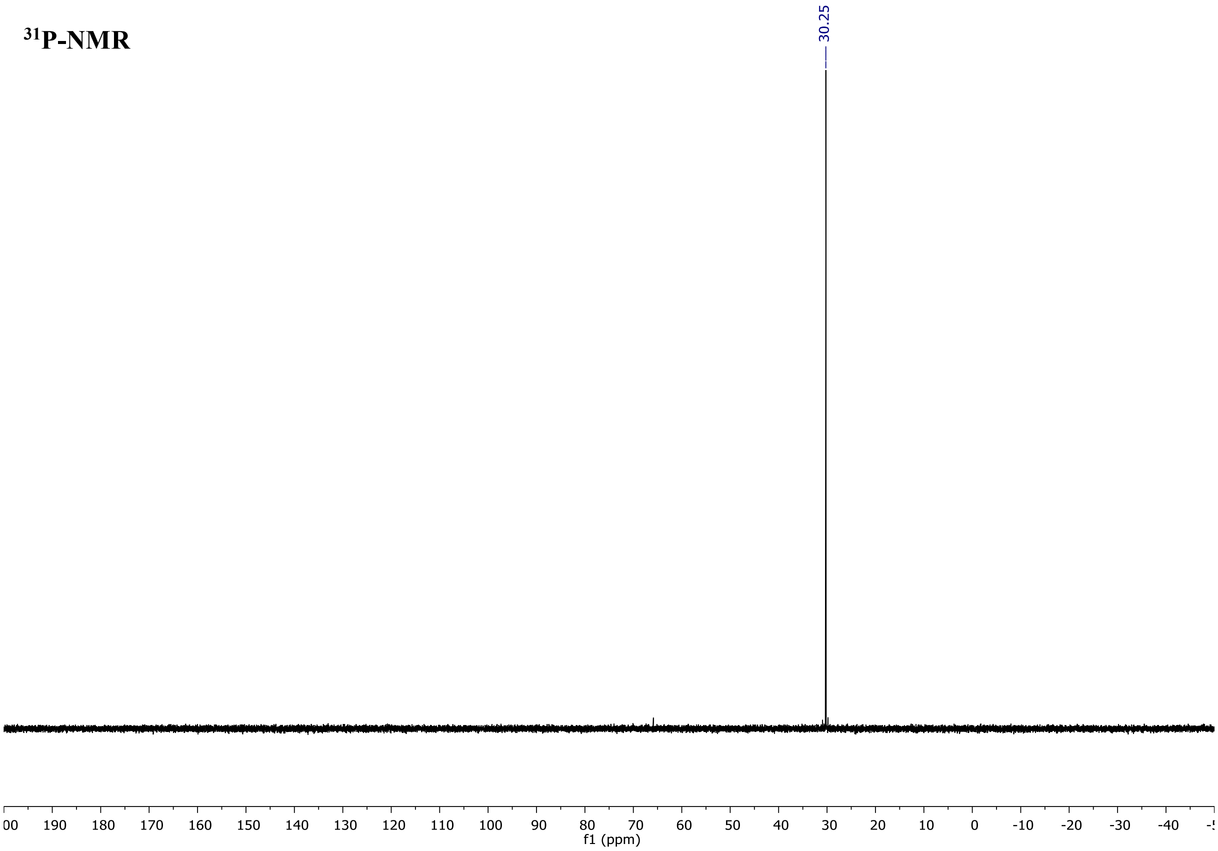

^{13}C-NMR

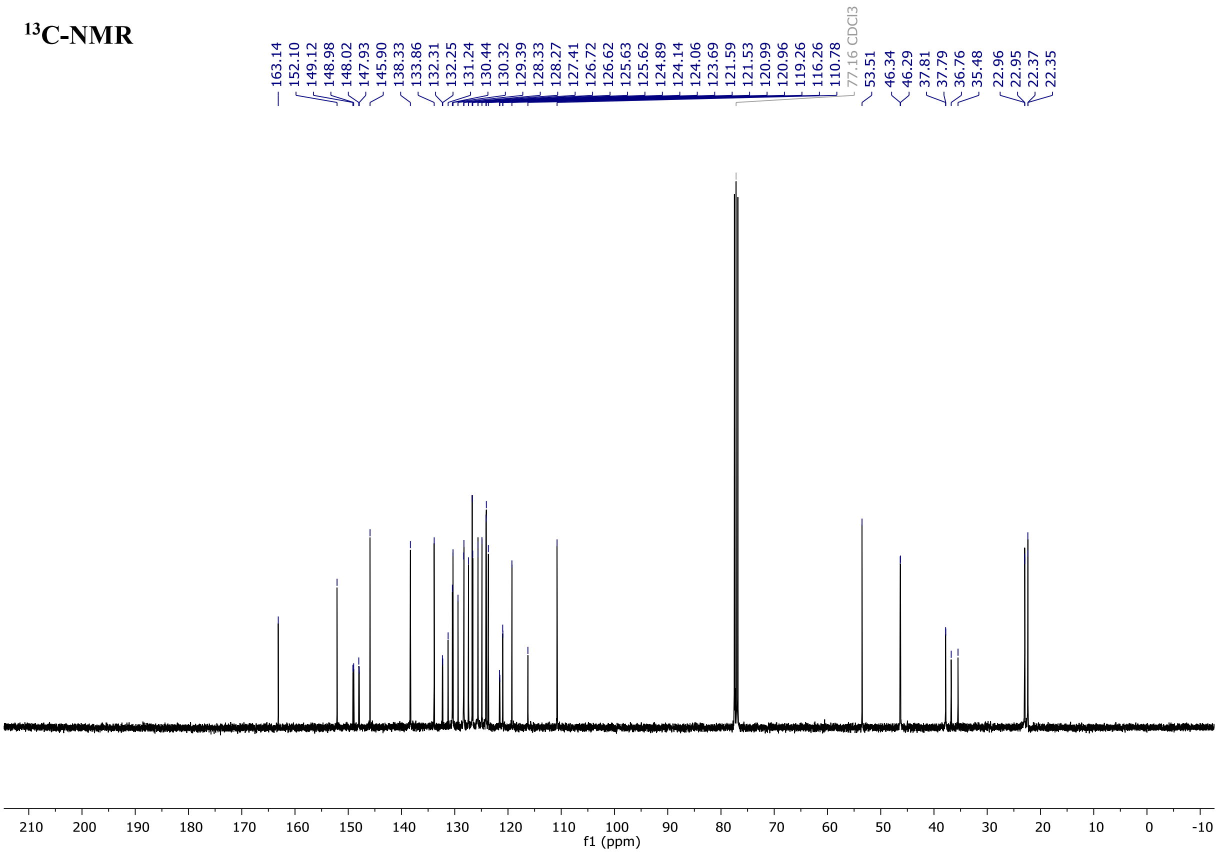

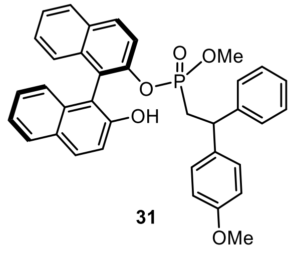
O
OMe
P
O
OH
OMe

31

^{1}H-NMR

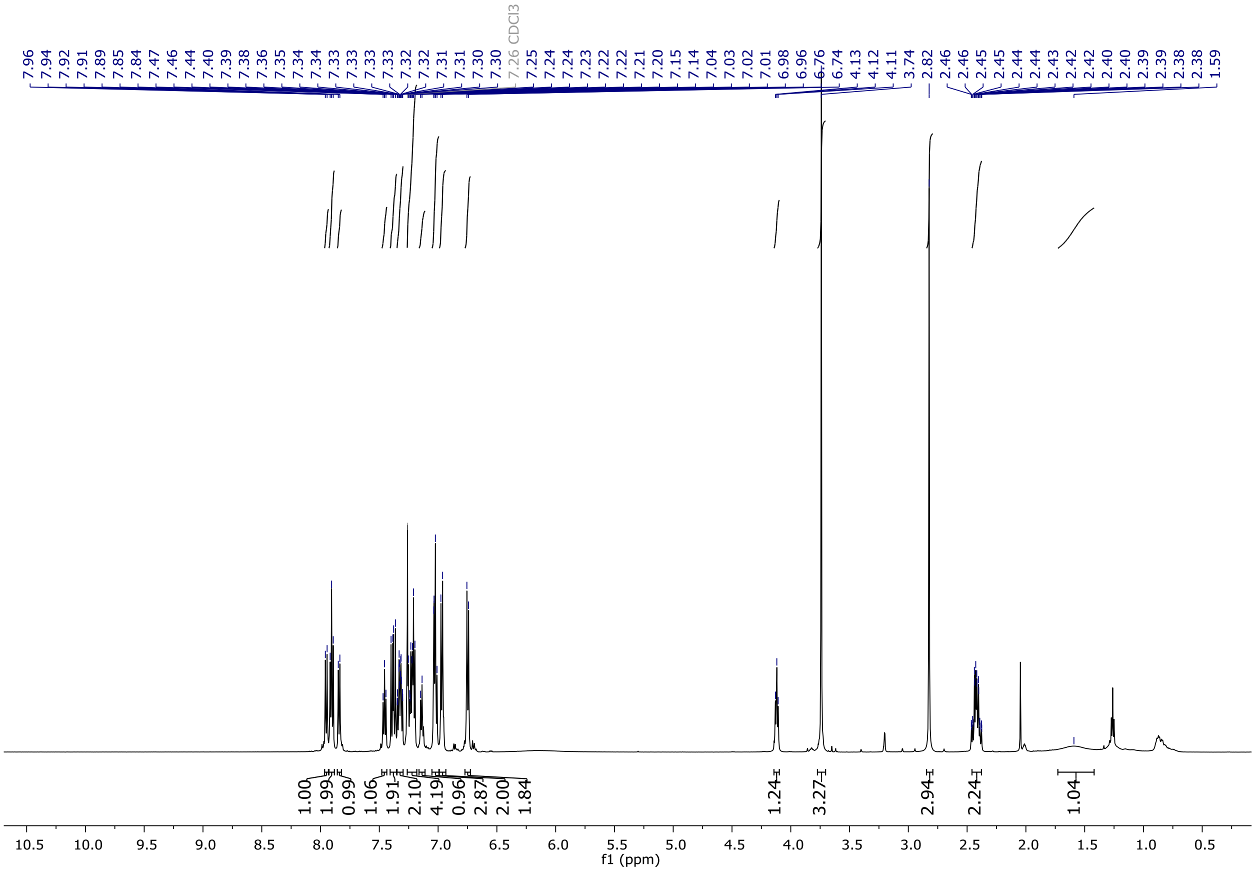
7.96 7.94 7.92 7.91 7.89 7.85 7.84 7.47 7.46 7.44 7.40 7.39 7.38 7.36 7.35 7.34 7.34 7.33 7.33 7.33 7.33 7.32 7.32 7.31 7.31 7.30 7.30 7.26 CDCl3 7.25 7.24 7.24 7.23 7.22 7.22 7.21 7.20 7.15 7.14 7.04 7.03 7.02 7.01 6.98 6.96 6.76 6.74 4.13 4.12 4.11 3.74 2.82 2.46 2.45 2.45 2.44 2.44 2.43 2.42 2.42 2.40 2.39 2.39 2.38 2.38 1.59
1.00 1.99 0.99 1.06 1.91 2.10 4.19 0.96 2.87 2.00 1.84
1.24
3.27
2.94
2.24
1.04
10.5 10.0 9.5 9.0 8.5 8.0 7.5 7.0 6.5 6.0 5.5 5.0 4.5 4.0 3.5 3.0 2.5 2.0 1.5 1.0 0.5
f1 (ppm)

^{31}P-NMR

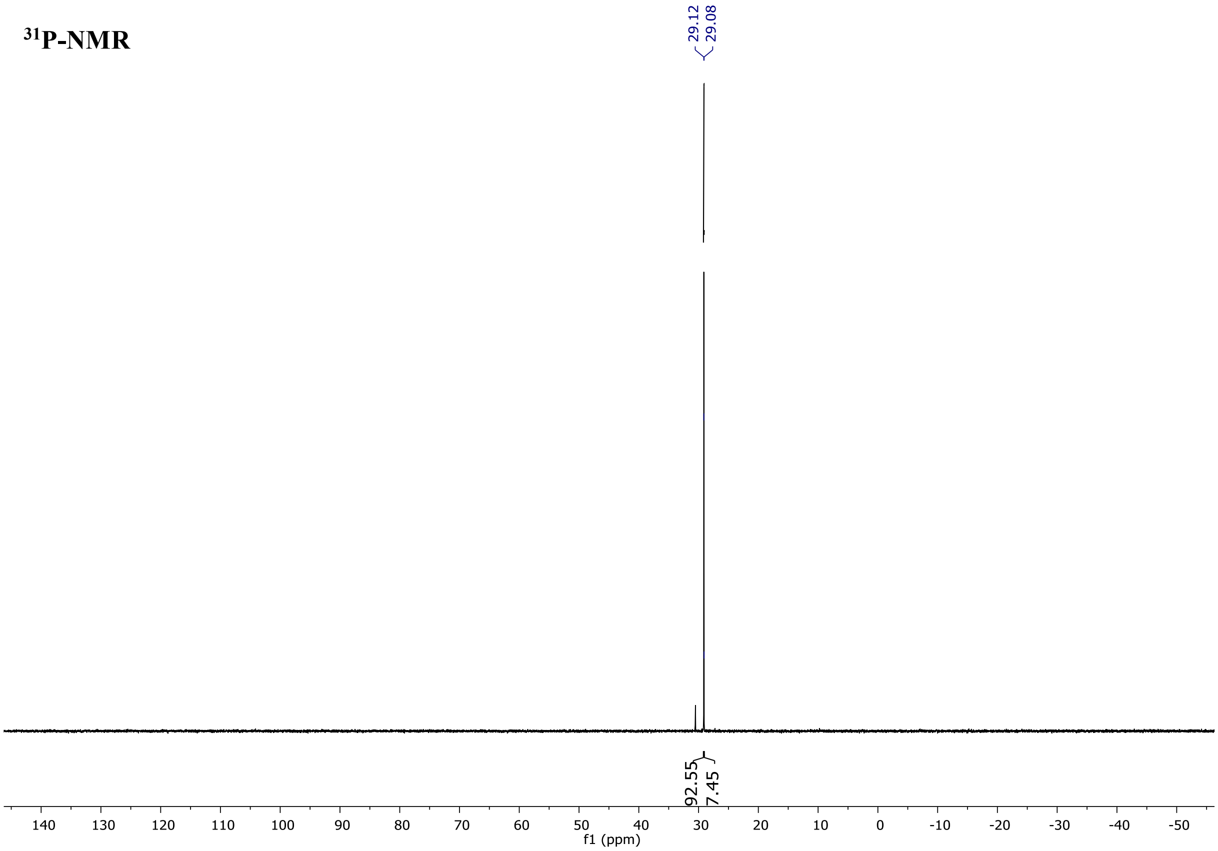

^{13}C-NMR

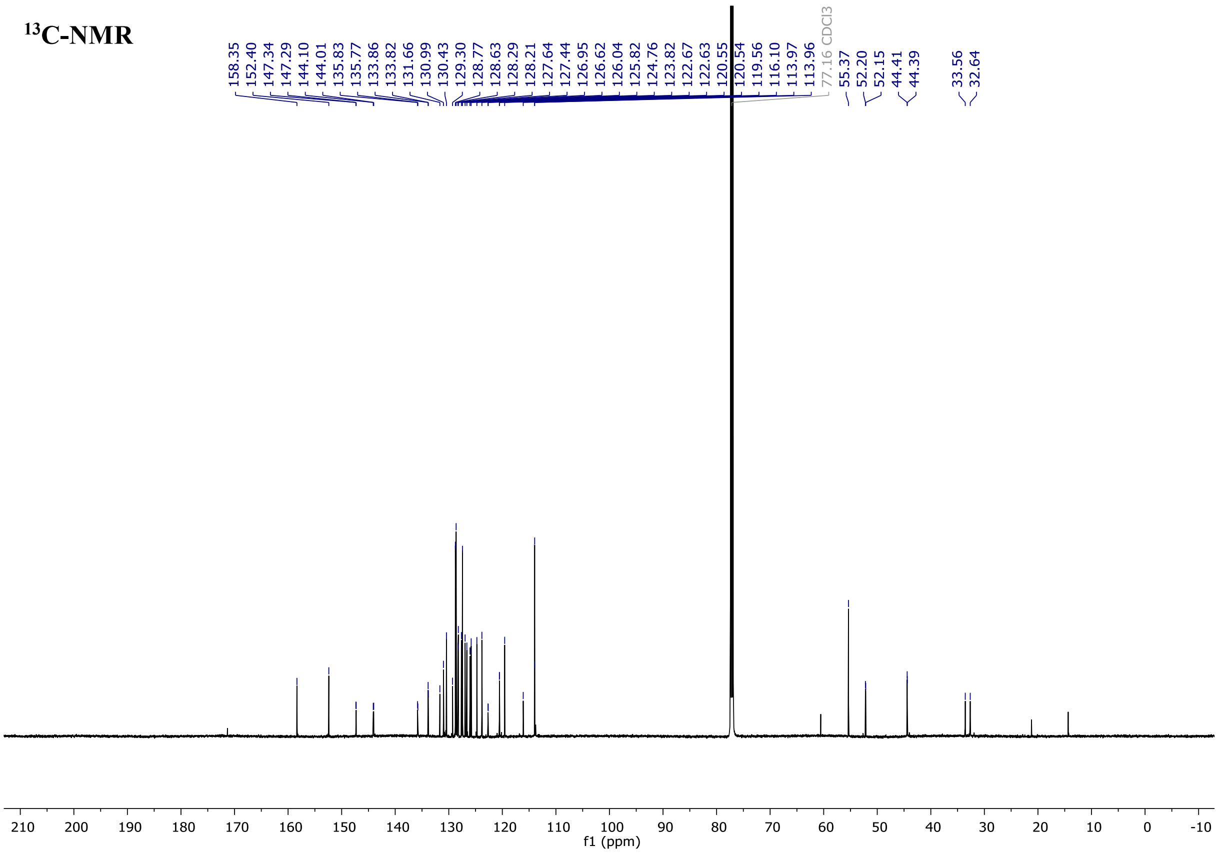

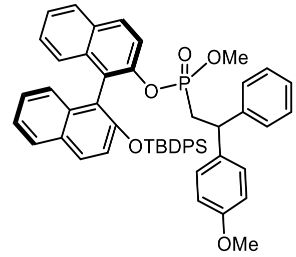
OMe
OTBDPS
OMe

31’

^{1}H-NMR

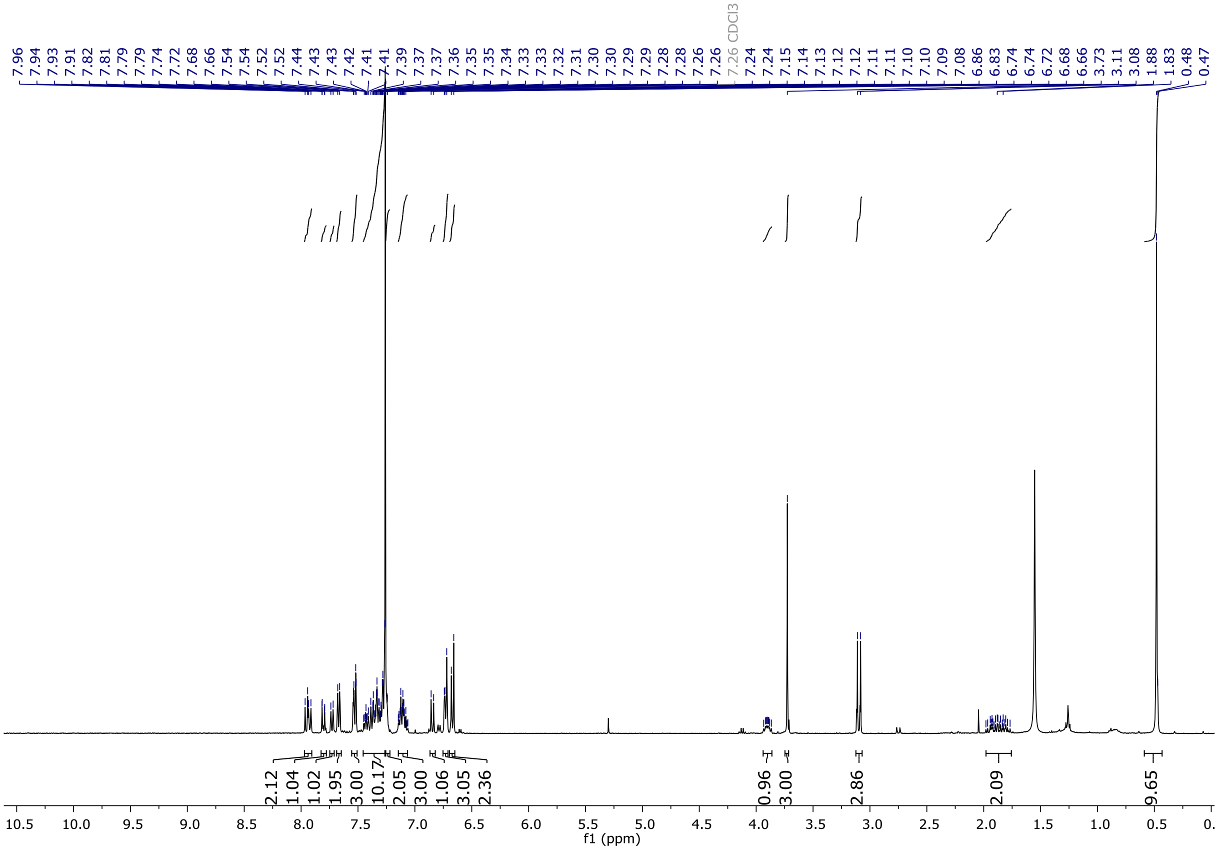
7.96 7.94 7.93 7.91 7.82 7.81 7.79 7.79 7.74 7.72 7.68 7.66 7.54 7.54 7.52 7.52 7.44 7.43 7.43 7.42 7.41 7.41 7.39 7.37 7.37 7.36 7.35 7.35 7.34 7.33 7.33 7.32 7.31 7.30 7.30 7.29 7.29 7.28 7.28 7.26 7.26 7.26 CDCl3 7.24 7.24 7.15 7.14 7.13 7.12 7.12 7.11 7.11 7.10 7.10 7.09 7.08 6.86 6.83 6.74 6.74 6.72 6.68 6.66 3.73 3.11 3.08 1.88 1.83 0.48 0.47
2.12 1.04 1.02 1.95 3.00 10.17 2.05 3.00 1.06 3.05 2.36 0.96 3.00 2.86 2.09 9.65
10.5 10.0 9.5 9.0 8.5 8.0 7.5 7.0 6.5 6.0 5.5 5.0 4.5 4.0 3.5 3.0 2.5 2.0 1.5 1.0 0.5 0.
f1 (ppm)

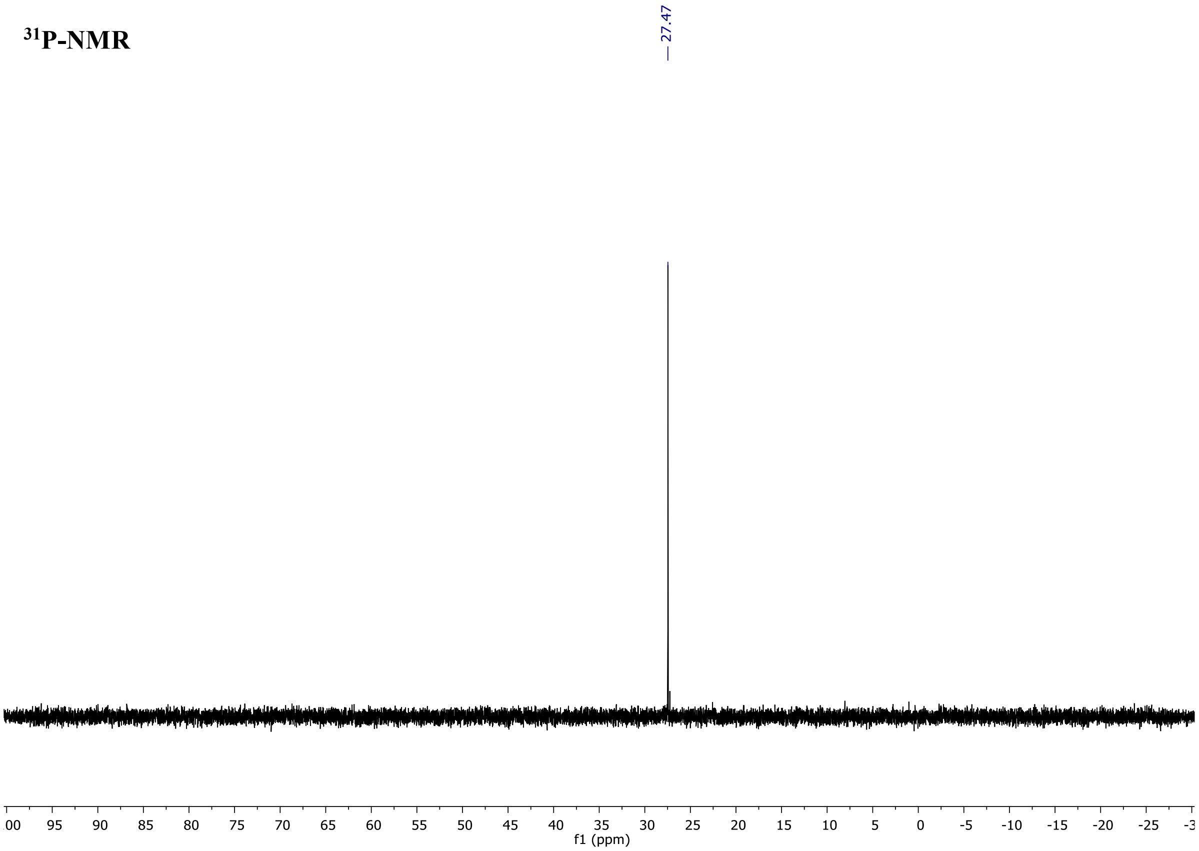
31P-NMR
27.47
f1 (ppm)

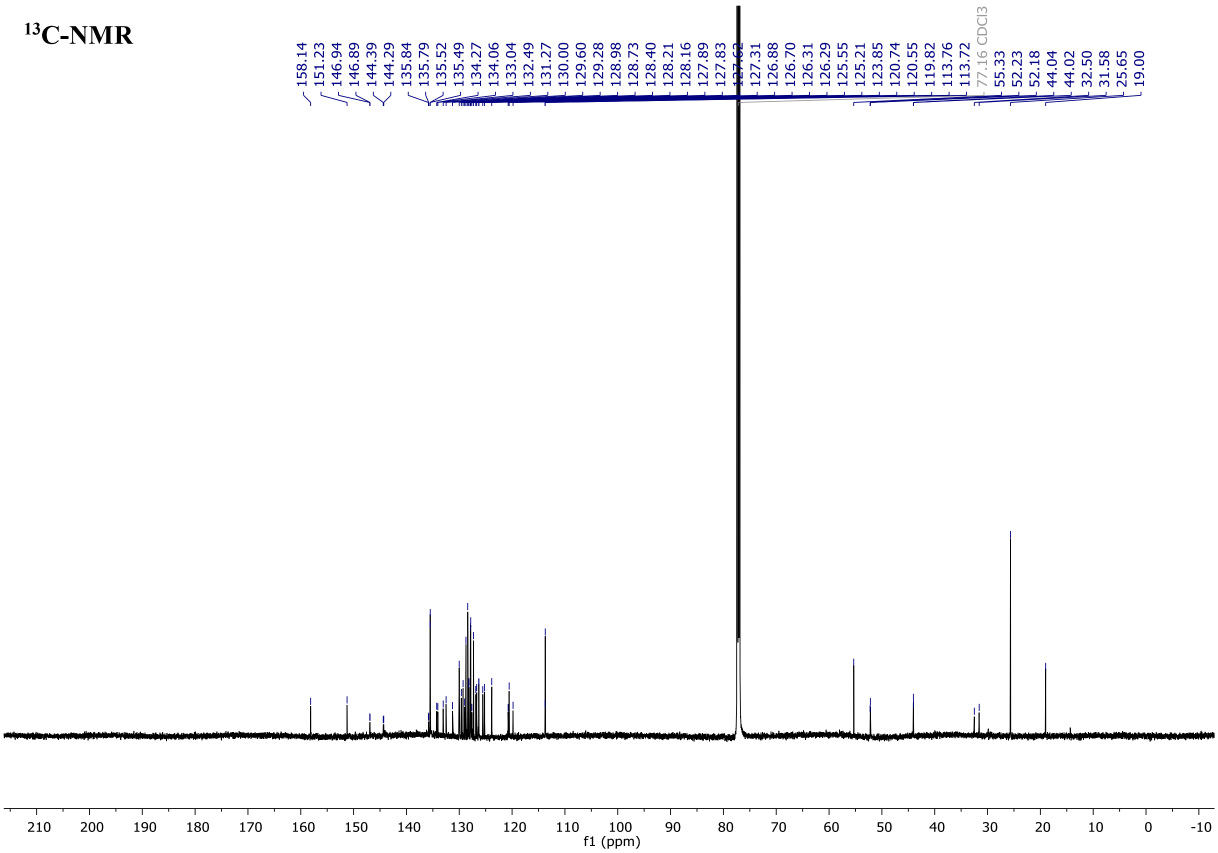
13C-NMR
158.14
151.23
146.94
146.89
144.39
144.29
135.84
135.79
135.52
135.49
134.27
134.06
133.04
132.49
131.27
130.00
129.60
129.28
128.98
128.73
128.40
128.21
128.16
127.89
127.83
127.62
127.31
126.88
126.70
126.31
126.29
125.55
125.21
123.85
120.74
120.55
119.82
113.76
113.72
77.16 CDCl3
55.33
52.23
52.18
44.04
44.02
32.50
31.58
25.65
19.00
f1 (ppm)

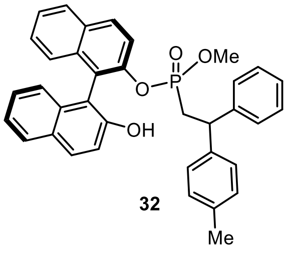

32

^{1}H-NMR

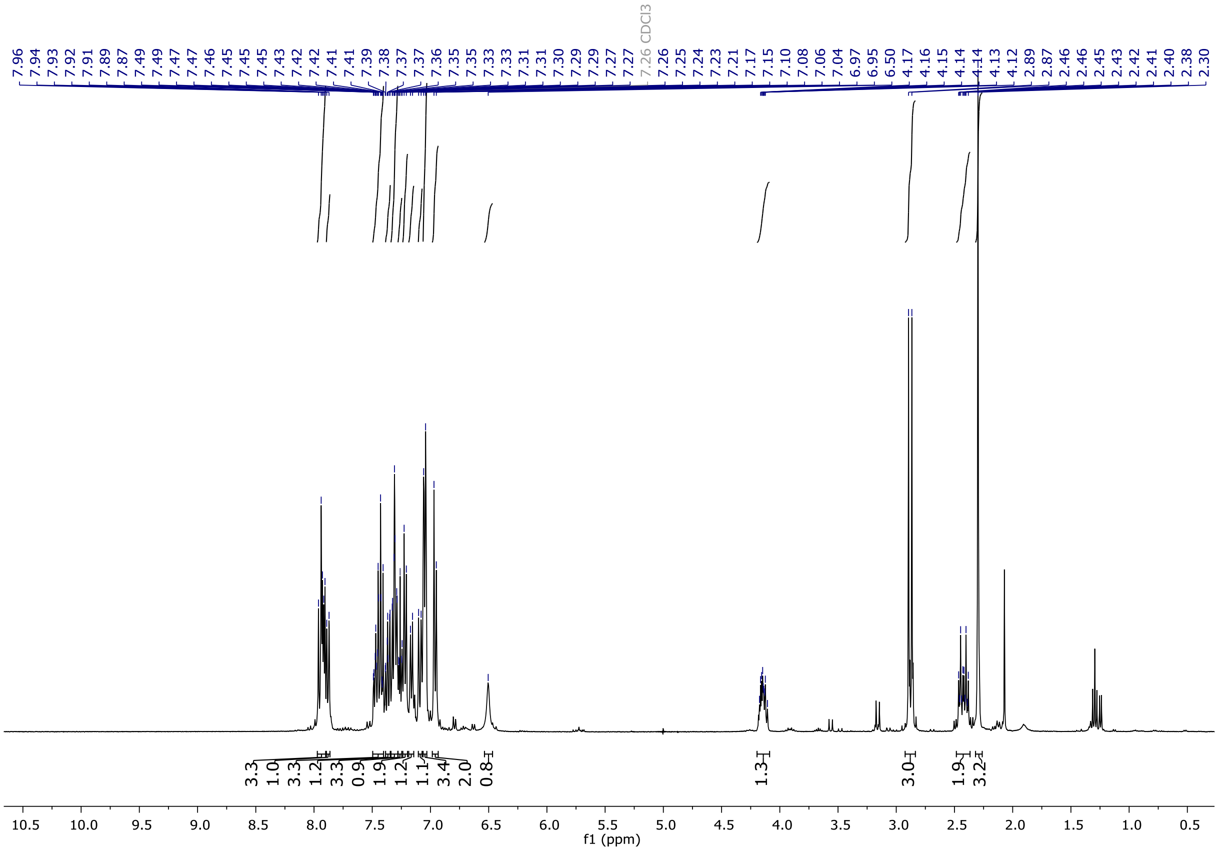

^{31}P-NMR

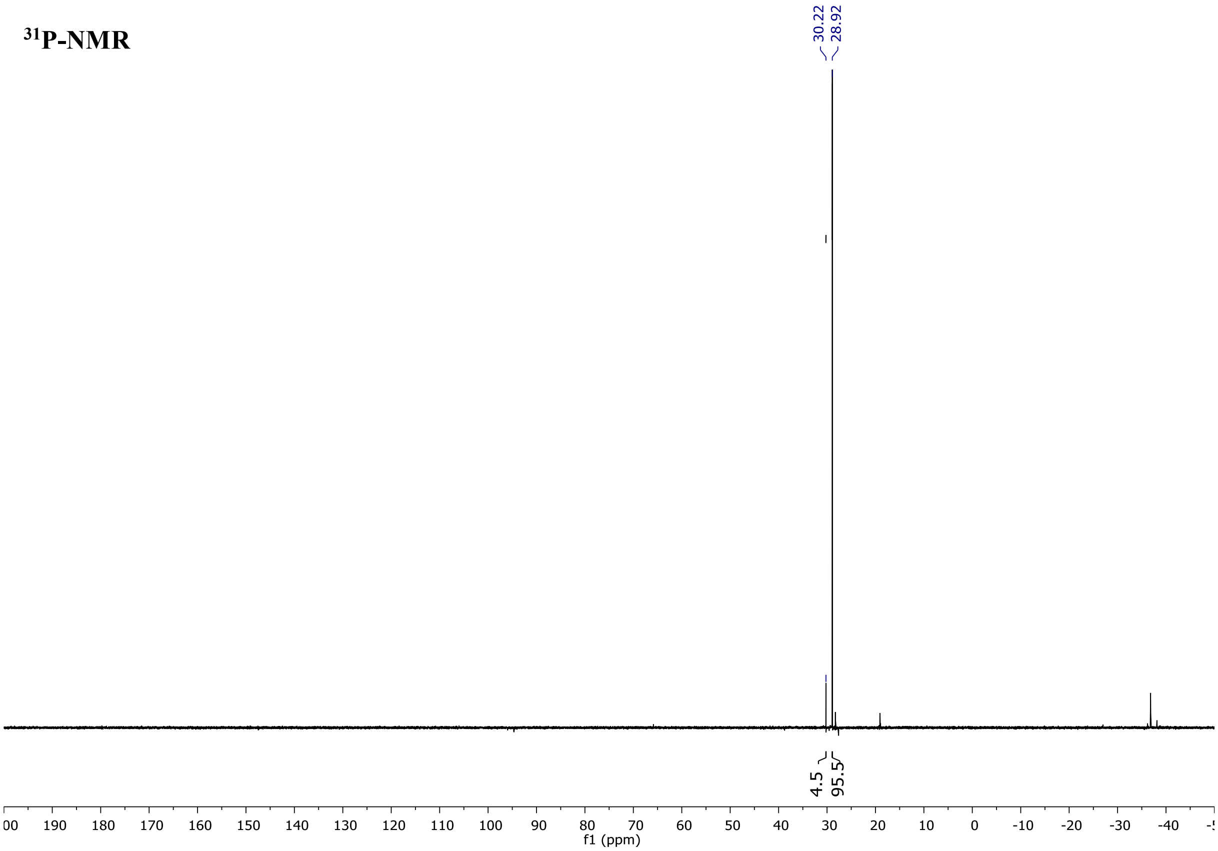

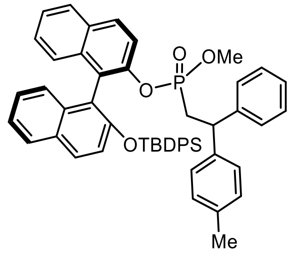

32'

^{1}H-NMR

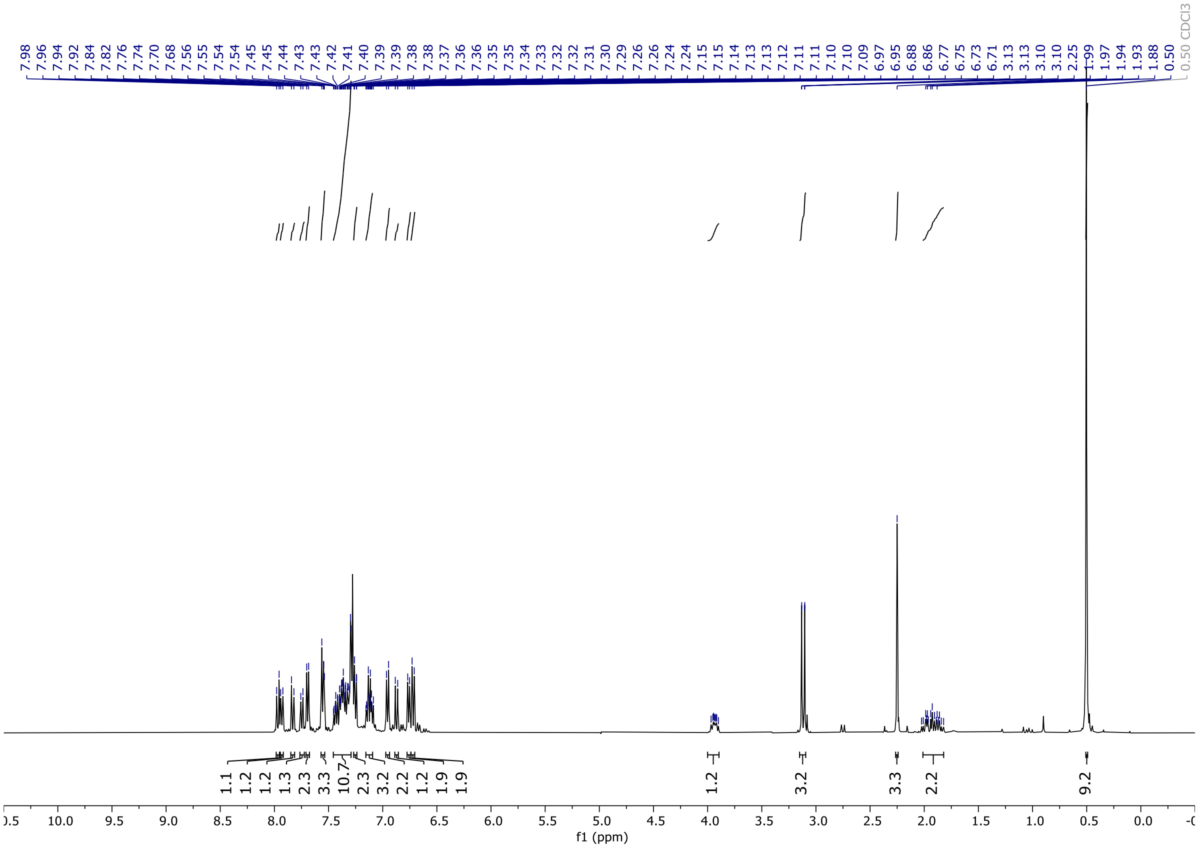

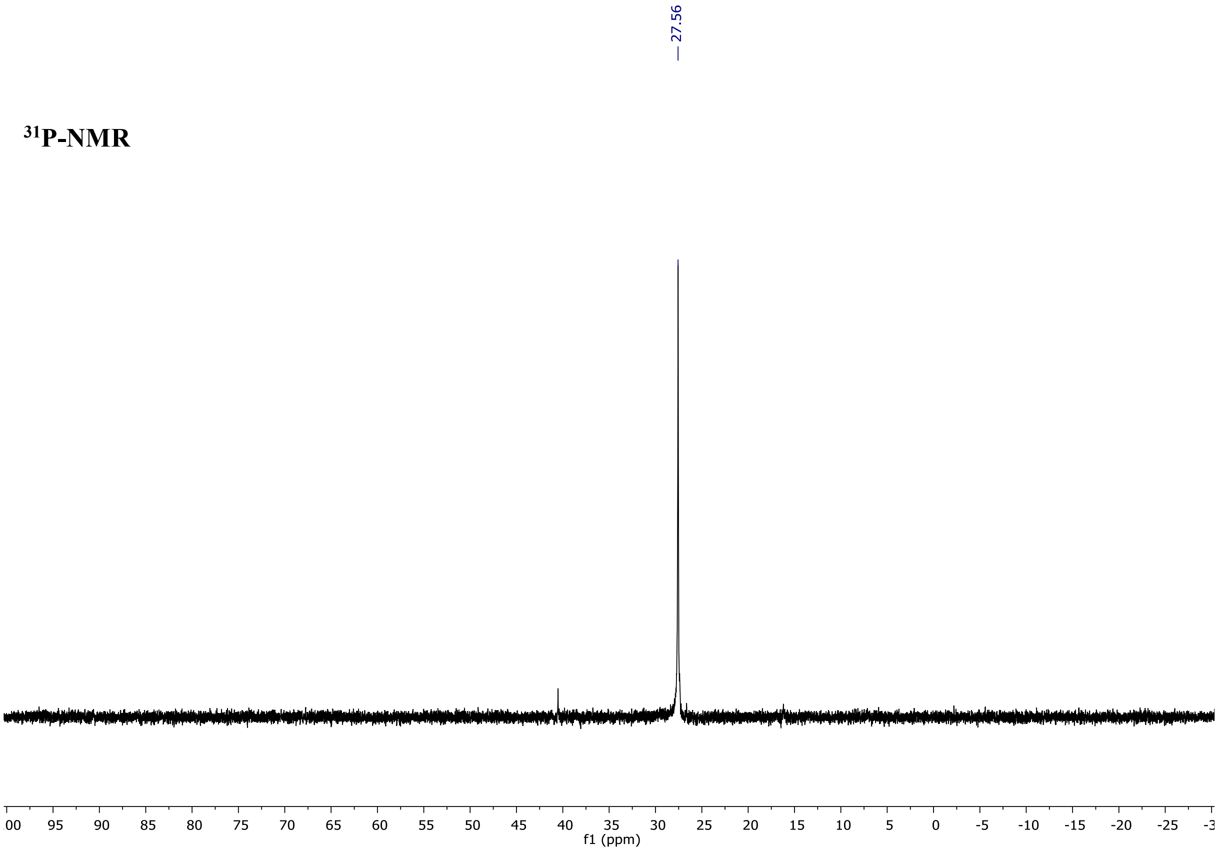
31P-NMR
27.56
f1 (ppm)

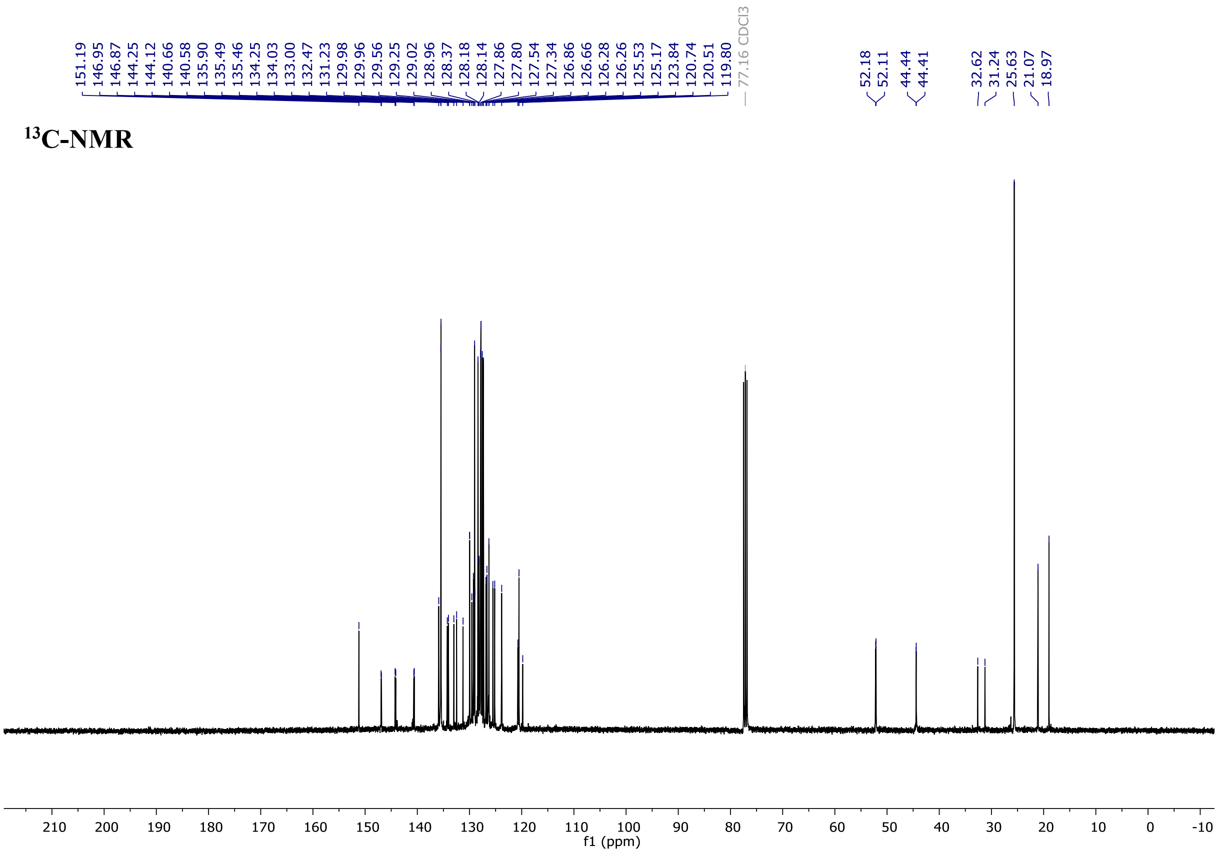
13C-NMR
151.19
146.95
146.87
144.25
144.12
140.66
140.58
135.90
135.49
135.46
134.25
134.03
133.00
132.47
131.23
129.98
129.96
129.56
129.25
129.02
128.96
128.37
128.18
128.14
127.86
127.80
127.54
127.34
126.86
126.66
126.28
126.26
125.53
125.17
123.84
120.74
120.51
119.80
77.16 CDCl3
52.18
52.11
44.44
44.41
32.62
31.24
25.63
21.07
18.97
f1 (ppm)

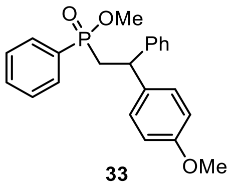

33

^{1}H-NMR

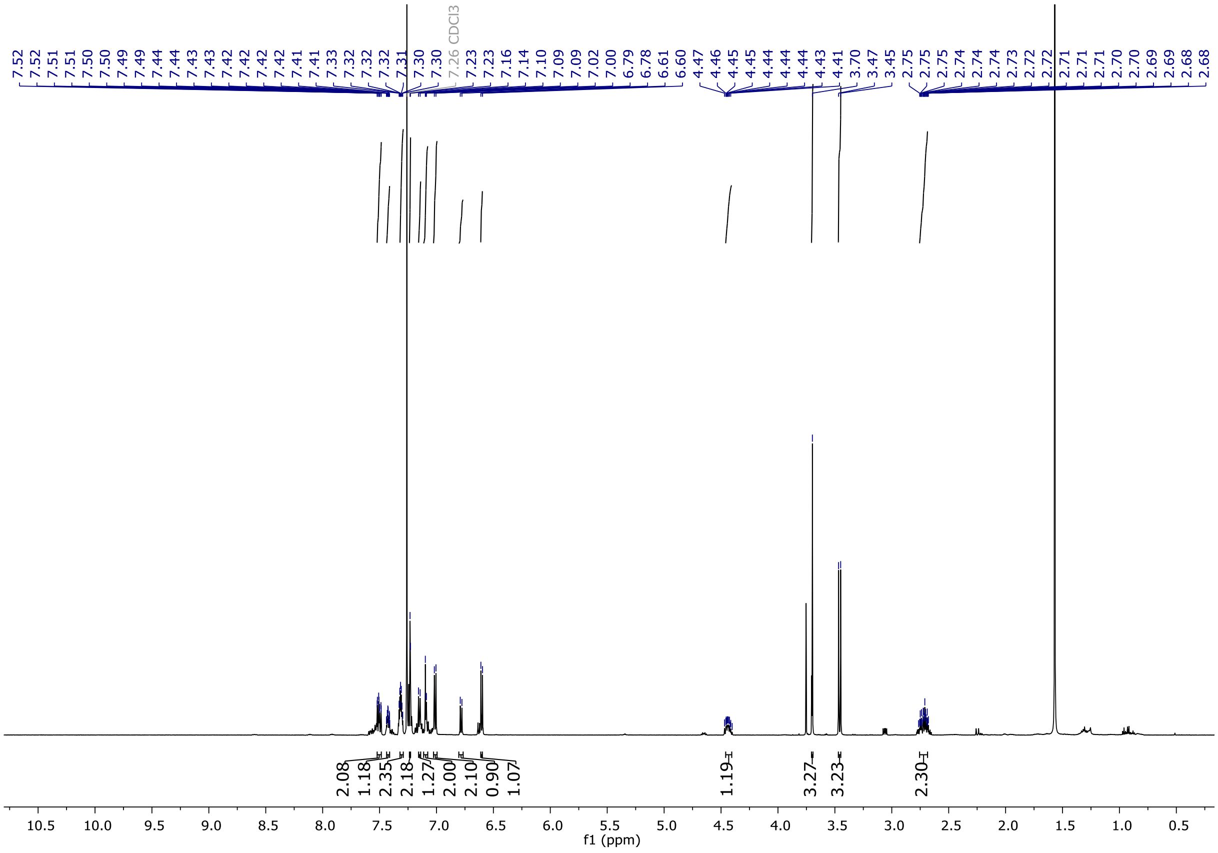

^{31}P-NMR

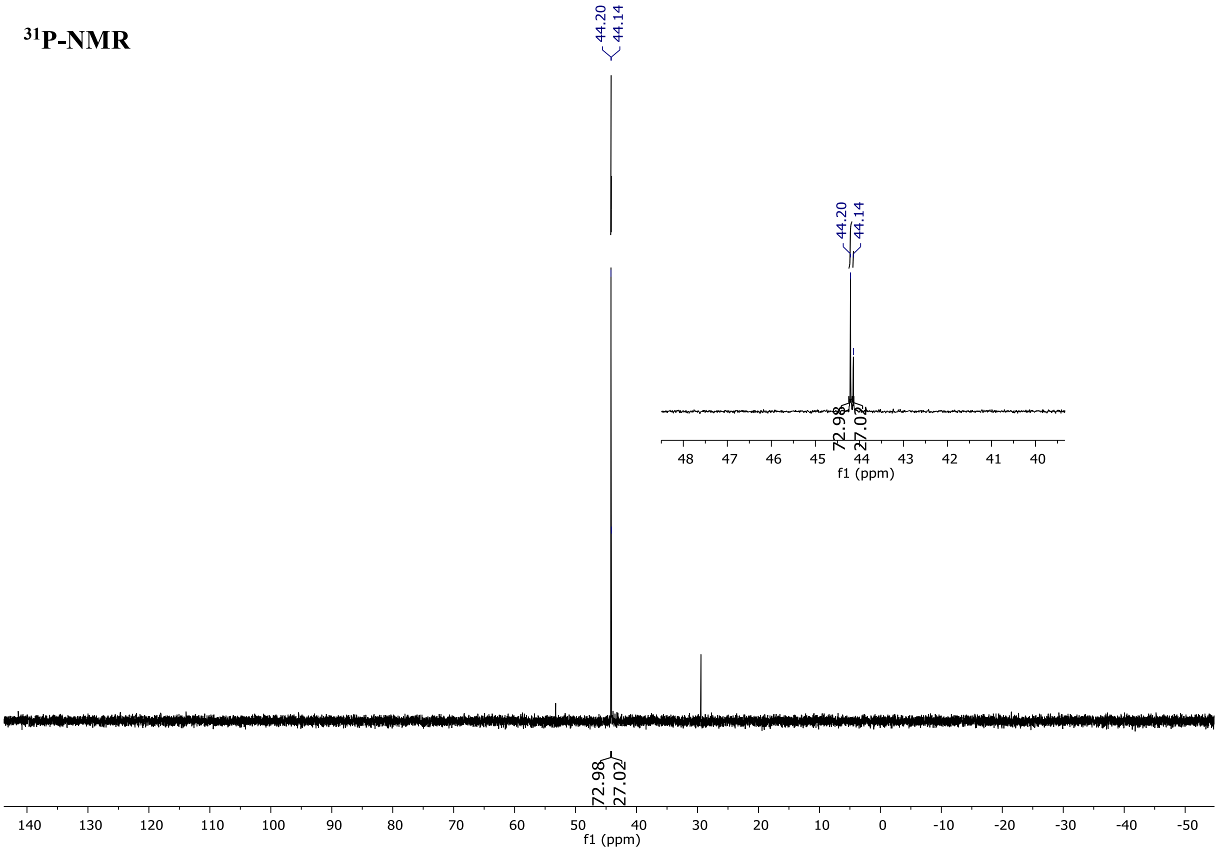

^{13}C-NMR

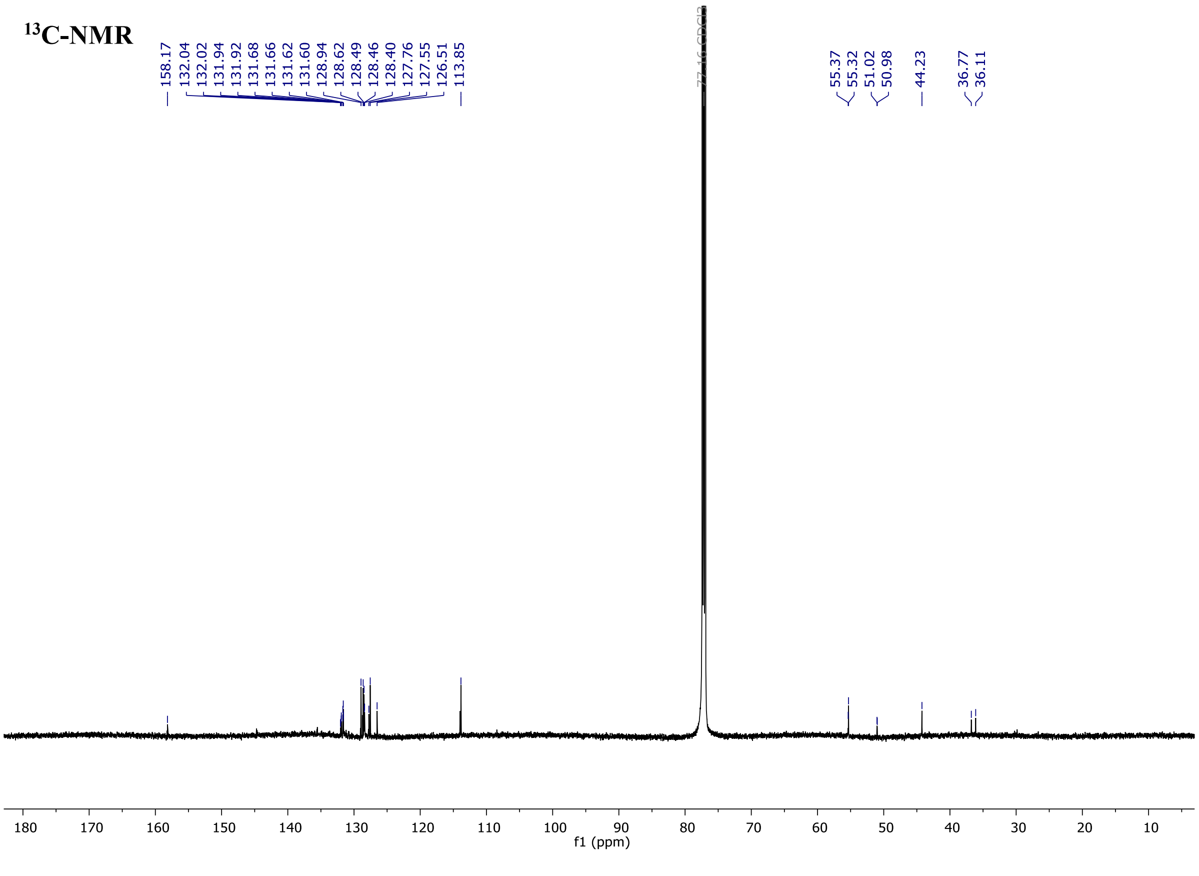

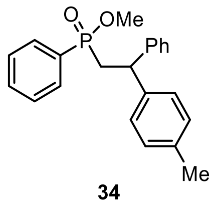

34

^{1}H-NMR

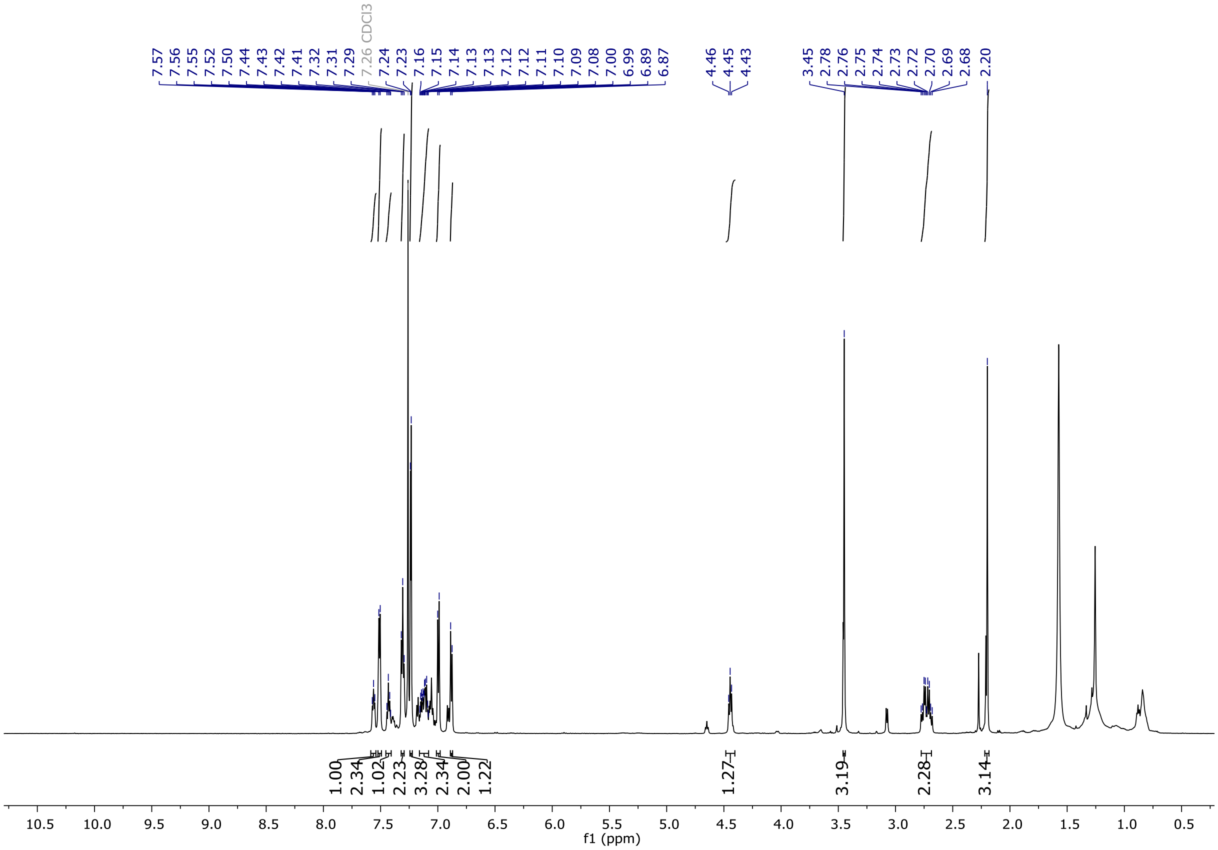

^{31}P-NMR

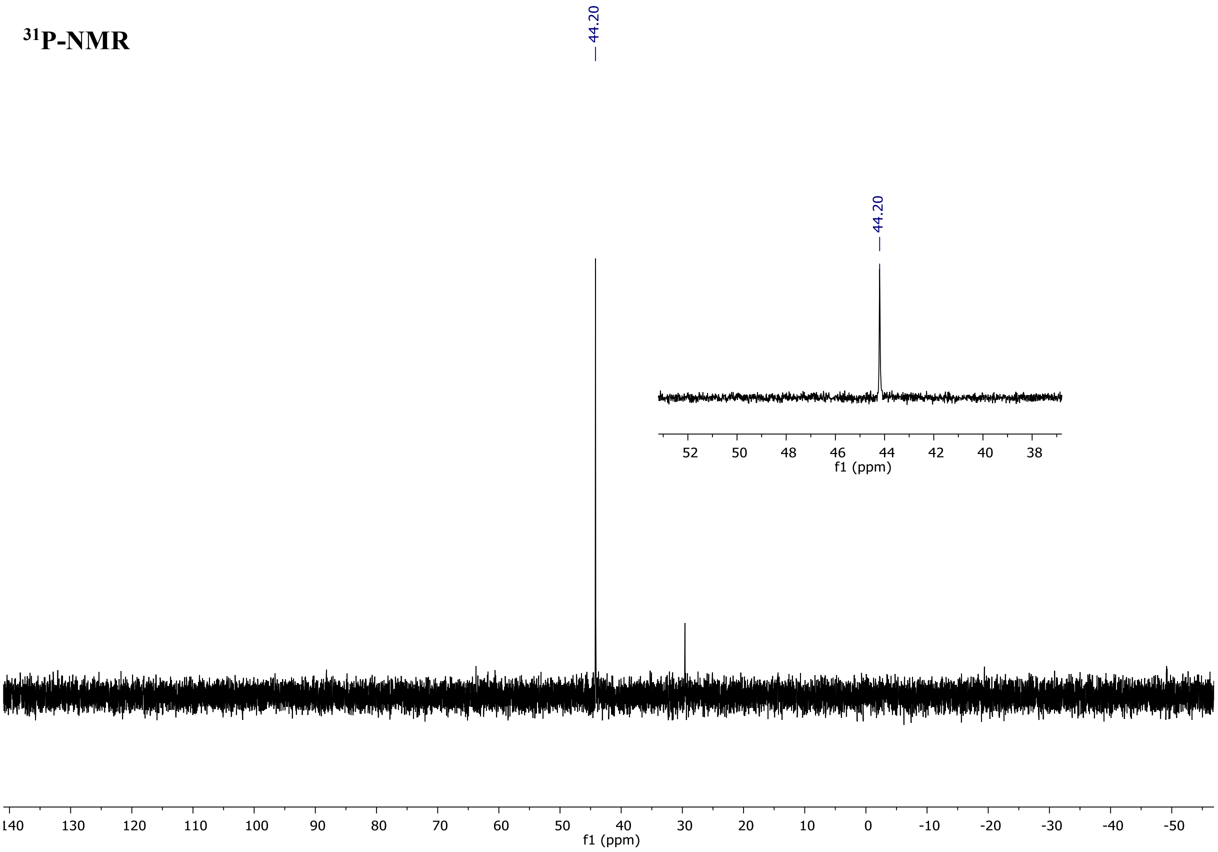

^{13}C-NMR

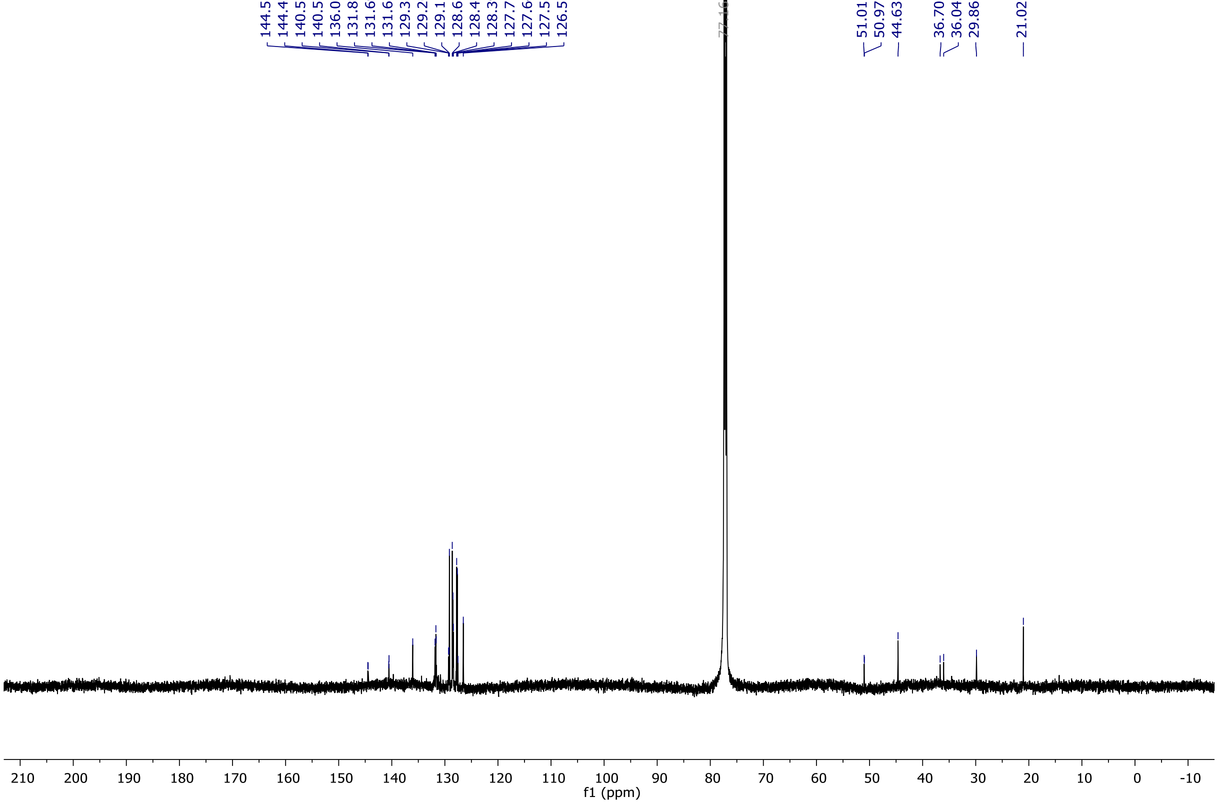

Supplement: SC-017-D5SC09946C-s001 [file SC-017-D5SC09946C-s001.pdf]
